# Redox-Active Ligands Permit Multielectron O_2_ Homolysis and O-Atom Transfer at Exceptionally High-Valent Vanadyl Complexes

**DOI:** 10.1021/jacs.4c18305

**Published:** 2025-04-09

**Authors:** Andrew G. Hill, Mariah C. Castillo, John Bacsa, Kaitlyn S. Otte, Jake D. Soper

**Affiliations:** † School of Chemistry and Biochemistry, Georgia Institute of Technology, Atlanta, Georgia 30332-0400, United States; ‡ X-ray Crystallography Center, Department of Chemistry, Emory University, 1515 Dickey Drive, Atlanta, Georgia 30322, United States

## Abstract

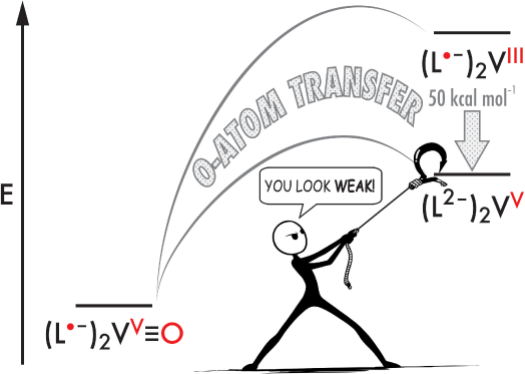

A five-coordinate chlorovanadium species supported by two redox-active *N*-phenyl aminophenol ligands was prepared. Experimental and computational data support formulation of this complex as [(^Ph^ap)(^Ph^isq)V^IV^Cl], containing one dianionic [^Ph^ap]^2–^ amidophenolate and one monoanionic [^Ph^isq]^•–^ iminosemiquinonate radical. Exposure of [(^Ph^ap)(^Ph^isq)V^IV^Cl] to O_2_ readily cleaves the O=O bond to generate [(^Ph^isq)(^Ph^ibq)V^IV^(O)Cl], containing an [^Ph^ibq] iminobenzoquinone, so the 2e^–^ oxidation is entirely ligand centered. [(^Ph^isq)(^Ph^ibq)V^IV^(O)Cl] is reduced by net H_2_ abstraction from 9,10-dihydroanthracene, or in reactions with main-group nucleophiles, such as PPh_3_ and Me_2_S, which form a new bond to oxygen and regenerate [(^Ph^ap)(^Ph^isq)V^IV^Cl]. Accordingly, the dioxygenase-type O_2_ activation and O-atom transfer cycling are a direct consequence of ligand redox noninnocence and covalency in the vanadium—aminophenol bonding. The reactions with O–atom donor and acceptor substrates establish a V≡O BDE of 73 ± 14 kcal mol^–1^ in [(^Ph^isq)(^Ph^ibq)V^IV^(O)Cl]. Reported V≡O BDEs in redox-innocent vanadyl complexes typically fall in the range of 120–170 kcal mol^–1^. Unlike later 3d metals, where M=O species are typically high energy and activated by, for instance, occupancy of M–O π* antibonding MOs, the exceptionally weak V≡O bond in [(^Ph^isq)(^Ph^ibq)V^IV^-(O)Cl] reflects stabilization of the reduced product. Thus, this research highlights an alternative pathway to generating strong oxidants that are not strong outer-sphere electron acceptors, with implications for the design of early metal catalysts for aerobic oxidations of weak O–atom acceptors or strong X–H bonds.

## Introduction

The use of dioxygen as the active oxidant in preparatory reactions is a tantalizing prospect.^1,2^ It is cheap, abundant, and green. Free radical autoxidations have a long and distinguished history in commodity chemicals production, but their use is largely limited to substrates that can undergo radical propagation, such as those with weak benzylic or allylic C–H bonds, and selectivity is limited to the thermodynamically weakest bond.^3−10^ Additionally, O-atom transfer between many main-group molecules does not proceed at appreciable rates despite large thermodynamic driving forces. Accordingly, selective targeting of stronger bonds or kinetically less accessible products motivates ongoing efforts to elaborate transition metal catalysts for selective aerobic oxidations.^4,11−13^

**Scheme 1:**
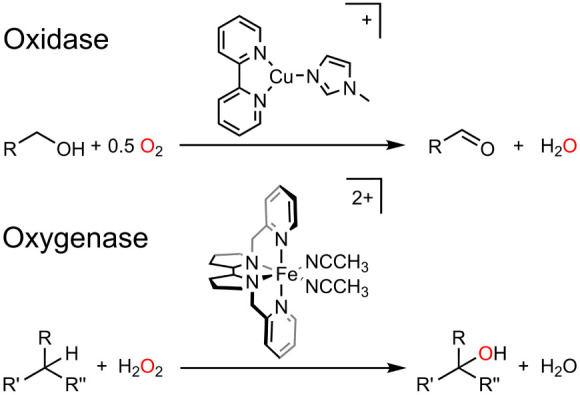
Examples of a Synthetic Oxidase Catalyst Reported by Stahl and Coworkers^20^ and a Synthetic Oxygenase Catalyst from White and Coworkers^26^

Enzymes for aerobic oxidations are conveniently classified as oxidases or oxygenases, and these terms have been extended to small molecule catalysts (Scheme 1).^14−18^ In oxidase-type reactions, O_2_ is an electron acceptor, and addition of four electrons and four protons generates two H_2_O equivalents.^3,4,14,18−20^ Synthetic oxidases featuring copper,^4,19,20^ palladium,^3,21^ and vanadium^22^ have been extensively studied, primarily for alcohol oxidation or dehydrogenation of C–C bonds. Oxygenases, in contrast, incorporate one or both oxygen atoms from O_2_ into the oxidation product, and metal–oxido intermediates predominate.^4,18,23−30^

The structures and reactivity of oxometal complexes have been a subject of intense focus for over 60 years,^31,32^ primarily because of their capacity to mediate selective oxidations in biological, industrial, and benchtop reactions, ranging from activation and functionalization of strong C–H bonds,^4,11,12,22,27,33,34^ to water oxidation,^35,36^ to alkene epoxidation.^11,12,25,37^ Absent ligand or secondary sphere efforts, the redox reactivity of an oxo complex generally tracks with the metal to which it is bound. For instance, mid-to-late transition metal–oxo (M=O) complexes are typically strongly oxidizing and electrophilic at O,^38^ reflecting the thermodynamic bias for lower oxidation states at more electronegative metals. Accordingly, their reactivity is dominated by O-atom transfer to a nucleophile such as a phosphine or alkene, or net H atom abstraction via protonation of the oxido ligand with concomitant reduction of the metal.^27^ This is exemplified by mononuclear oxo compounds of Groups 6–8 that function as strong stoichiometric oxidants^27,38−41^ (e.g., MnO_4_^–^, CrO_2_Cl_2_) and biomimetic oxygenase-type catalysts which operate via high-valent iron and manganese oxido intermediates.^11,12,18,24−27,42,43^

O-atom transfer at early metal–oxo complexes is disfavored because of the thermodynamic bias against 2e^–^ reduction of the metal center.^33,36,44,45^ For example, oxovanadium complexes have demonstrated utility in a variety of oxidation reactions,^22,46^ but examples of reductive O-atom transfer from an oxovanadium complex are exceedingly rare,^40,47^ reflecting the strong V≡O bond^40,48^ and the tendency of electropositive vanadium to retain high +4 and +5 oxidation states.^49^ For instance, the capacity of polyoxovanadates to accept multiple e^–^/H^+^ pairs via sequential PCET is ascribed to distribution of the reducing equivalents across the vanadium centers.^50,51^ These same properties preclude oxygenase-type catalysis at early metals. Highly reduced vanadium species react rapidly with dioxygen, sometimes forming terminal oxos, but these reactions are usually low yielding and lead to olation or μ-oxo polymerization.^39^ Accordingly, the known aerobic oxidation catalysis at oxovanadium compounds is dominated by oxidase-type reactions which are redox-neutral at vanadium or involve vanadium(V/IV) cycling.^22,52,53^ Vanadium(III) intermediates have been proposed, but evidence for their involvement is scant,^46,53^ and 2e^–^ redox cycling has not been conclusively demonstrated in a vanadium aerobic oxidation cycle.

The capacity of redox-active ligands to function as electron reservoirs for organometallic-type small molecule multielectron redox reactions at d^0^ metals was first demonstrated by Heyduk and coworkers in oxidative additions of dihalides at zirconium(IV) complexes supported by amidophenolate ligands.^54^ This strategy was subsequently extended to other redox processes at d^0^ early metals,^55−60^ as well as stoichiometric and catalytic bond-making and -breaking reactions that span the d- and f-blocks and extend into the main group,^61−72^ including catalytic O_2_ activation and ″nonclassical″ O atom transfer at Mo and high-valent Re.^73−78^

We speculated that redox-active ligands might similarly engender O_2_ homolysis and O atom transfer at early metals, thereby extending dioxygenase-type aerobic catalysis to Groups 3–6. The viability and power of this approach are established herein. A vanadium complex in the formal +5 oxidation state supported by redox-active aminophenol ligands is described and shown to cleanly homolyze O_2_ to form oxovanadium products two redox levels above the parent material. These oxovanadium complexes exhibit reactivity typical of late metal–oxos. They reductively transfer an O atom to phosphine or alkene nucleophiles and are reduced by net H_2_ transfer in sequential 1e^–^ + 1H^+^ proton-coupled electron transfer (PCET) steps. Ligand-centered redox is shown by spectroscopic and computational methods to be an essential component of both net 2e^–^ redox processes, establishing design principles for the development of dioxygenase-type aerobic oxidation catalysis at ″redox inert″ early metals.

## Results

### Synthesis and Characterization of [(^Ph^ap)(^Ph^isq)V^IV^Cl] (I)

**Figure 1:**
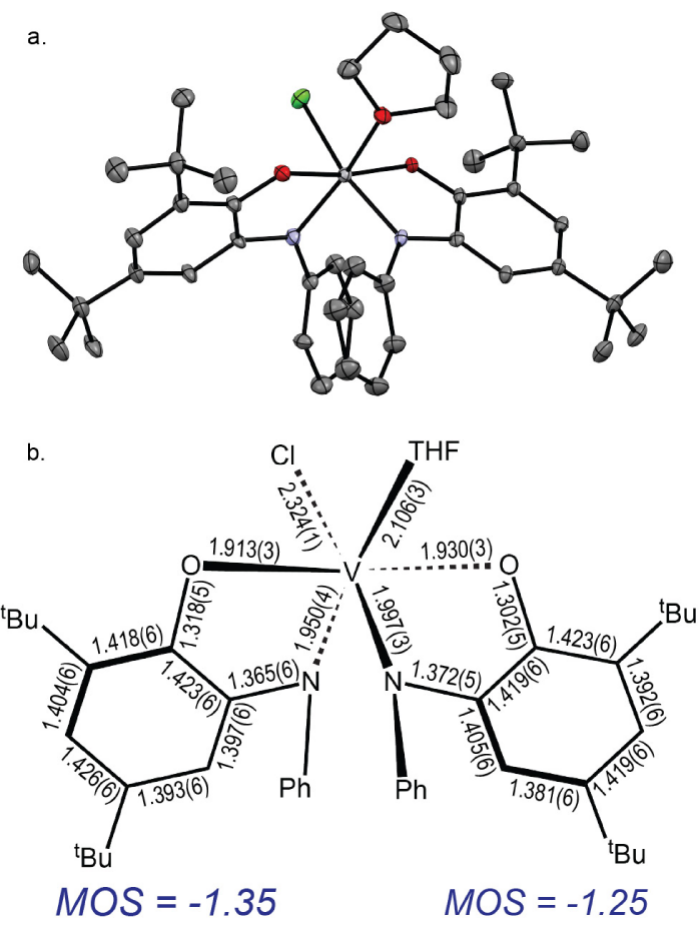
**a.** ORTEP plot of the THF adduct of [(^Ph^ap)(^Ph^isq)V^IV^Cl] (**I**). Ellipsoids drawn at 50% probability. **b.** Schematic of selected bond lengths (Å) for **I** and calculated metrical oxidation states^79^ for each ligand.

Addition of a yellow THF solution of K_2_[^Ph^ap] ([^Ph^ap]^2–^ = 2,4-di*tert*-butyl-6-(phenylamido)phenolate) to a dark red THF solution of VOCl_3_ resulted in an immediate color change to blue, from which a dark blue solid (**I**) was obtained in 85% yield (eq 1). The ^1^H NMR spectrum of **I** in C_6_D_6_ shows a forest of peaks in the regions expected for aryl and *tert*-butyl resonances (Figure S8), but no signals are observed by ^51^V NMR spectroscopy. Vapor diffusion of pentane into a concentrated THF solution of **I** afforded crystals suitable for analysis by X-ray diffraction. As shown in Figure 1, **I** is pseudo-octahedral with the amido N-donors occupying *cis* sites (θ_N–V–N_ = 89.2(1)°), and the phenoxide O-donors in an approximately *trans* disposition (θ_O–V–O_ = 171.1(1)°), giving the [(^Ph^ap)_2_V] core quasi-*C*_*2*_ symmetry. The remaining sites are occupied by a chloride and a solvent-derived THF, making the two aminophenol derived ligands inequivalent. The V–N bond *trans* to the chloride ligand is 0.047(5) Å longer than the V–N bond *trans* to the THF ligand. The average C–C bond distances around both aminophenol rings are 1.41 ± 0.25 Å, but show a slight quinonoid-type deviation from aromaticity (Figure 1b), which is manifested in the computed metrical oxidation states (MOS)^79^ of –1.35 for the ligand *trans* to the THF ligand and –1.25 for the ligand *trans* to the chloride. The MOS values for two fully reduced [^Ph^ap]^2–^ ligands should sum to –4.0. The sum of –2.6 in **I** implies significant but incomplete oxidation with removal of more than one electron equivalent across the ligand pair. Such noninteger MOS values for amidophenolate ligands are indicators of strong π interactions with the metal center or facile electron delocalization in the ground state.^79−81^
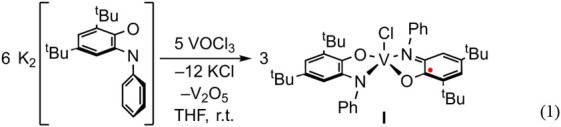
1

Evans’ method solution magnetic moment^82^ measurements of **I** at 25 °C returned values of μ_eff_ = 1.0–2.5 μ_B_ at 25 °C in CDCl_3_, toluene-d_8_, THF-*d*_8_, and C_6_D_6_ (Figures S9–S13), with samples more consistently returning moments on the lower end of the reported range, which is well below that expected for an S = 1 ground state. A VT Evans’ method study showed only a linear dependence of the magnetic moment on T^1/2^ in CDCl_3_ from –50 °C to +50 °C (Figure S14). The absorption spectrum of **I** is dominated by two charge transfer features ca. 600–700 nm; no CT bands are observed at wavelengths >1000 nm (Figure S1). Low-energy LLCT transitions are commonly observed in complexes containing electronically inequivalent redox active ligands, but the absence of these features does not preclude such an assignment.^83^

In total, the experimental data are most consistent with formulation of **I** as [(^Ph^ap)(^Ph^isq)V^IV^Cl] ([^Ph^isq]^•–^ = 2,4-di*tert*-butyl-6-(phenylimino)semiquinonate) in the solid state, containing a d^1^ vanadium(IV) and a second unpaired electron shared across the two aminophenol ligands, antiferromagnetically coupled to the vanadium d electron. However, the noninteger ligand oxidation state implies strong electron delocalization and covalency in the vanadium–aminophenol bonding,^79,84^ and the observed paramagnetism might reflect geometric isomerism in solution or even multiple contributors to the electronic ground state, which are not well captured by the oxidation states indicated in the formula.

The electronic structure of **I** was examined with unrestricted DFT calculations (PBE0, def2-TZVPP, D3BJ, RIJCOSX) in the singlet, triplet, and quintet states. In each spin state, a constrained hydrogens-only optimization was performed starting from the coordinates of the X-ray crystal structure to benchmark and validate the inferences drawn from the solid state-data, above. The S = 0 solution was found to be the lowest in energy by 9.4 kcal/mol, however, it shows high levels of spin contamination (⟨S^2^⟩ = 1.28) from higher multiplicity states. The S = 1 (⟨S^2^⟩ = 2.56) solution also shows significant contamination. The involvement of an S = 2 (⟨S^2^⟩ = 6.03) state is highly unlikely as it was calculated to lie at +23.0 kcal/mol relative to S = 0 and does not agree with the experimentally measured solution magnetic moment.

**Figure 2:**
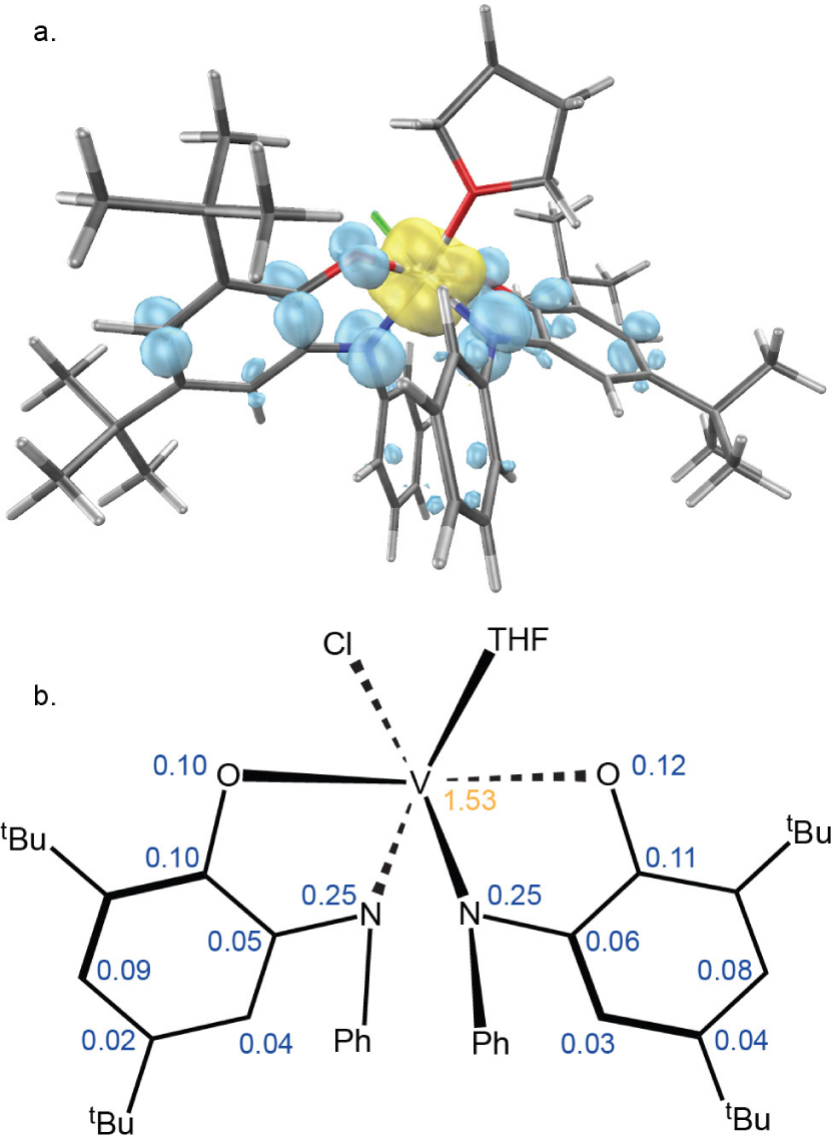
**a.** Spin density plot of THF adduct of [(^Ph^ap)(^Ph^isq)V^IV^Cl] (**I**) generated in IQmol (isosurface value 0.006). **b.** Löwdin spin population per atom (*n* > 0.02). Spin down density is shown in yellow.

Despite being a closed-shell compound, the singlet calculation shows a high degree of spin polarization. The vanadium center has a spin population of *n* = 1.53 (Figure 2), in excellent agreement with the intermediate oxidation state assigned based on the metrical oxidation states of the redox-active ligands (*vide supra*). Each aminophenol ligand has significant spin delocalized in its π system, which is antiferromagnetically coupled with the spatially separated spin on vanadium.

The S = 1 calculation (Figure S39) also shows an intermediate spin population on the metal *n* = 1.68, and one ligand with parallel and one with antiparallel spin relative to the vanadium electron spin. These singlet and triplet calculations qualitatively agree in having one frontier spin–orbital that is mostly a metal d orbital and three spin–orbitals localized in the ligand π systems with some ligand–metal π covalency (cf. Figures S35–S38, S40–S43). The simplest formulation for **I** based on these results, therefore, agrees with the [(^Ph^ap)(^Ph^isq)V^IV^Cl] oxidation state derived from the crystallography.

Full optimizations of **I** performed at the same level of theory in implicit benzene solvent (CPCM) suggest a compression of the energetically accessible spin states, with the S = 1 state in **I** at +0.1 kcal/mol above the S = 0 ground state, and the S = 2 state at +5.2 kcal/mol, consistent with the observed intermediate solution magnetic moment measurements. The simulated solution-phase electronic structures have 1.75 and 1.90 unpaired electrons on the vanadium center in the S = 0 and S = 1 states, respectively, and MOS calculations on the computationally optimized structures give total charges of –2.15 (S = 0) and –2.00 (S = 1) across the two redox-active ligands (Table S26). The sum of these data are therefore most consistent with a solution vanadium(III) [(^Ph^isq)_2_V^III^Cl] formulation. However, the computational results highlight (1) the limitations of oxidation state formalisms for such species with highly covalent metal–ligand bonding; (2) the sensitivity of these species to isomerization or bond fluctuations which are probable in solution and challenging to assess experimentally. We caution against overinterpretation in extrapolating the solid-state assignments to homogeneous solution or overweighting any of the oxidation state assignments.

**Figure 3:**
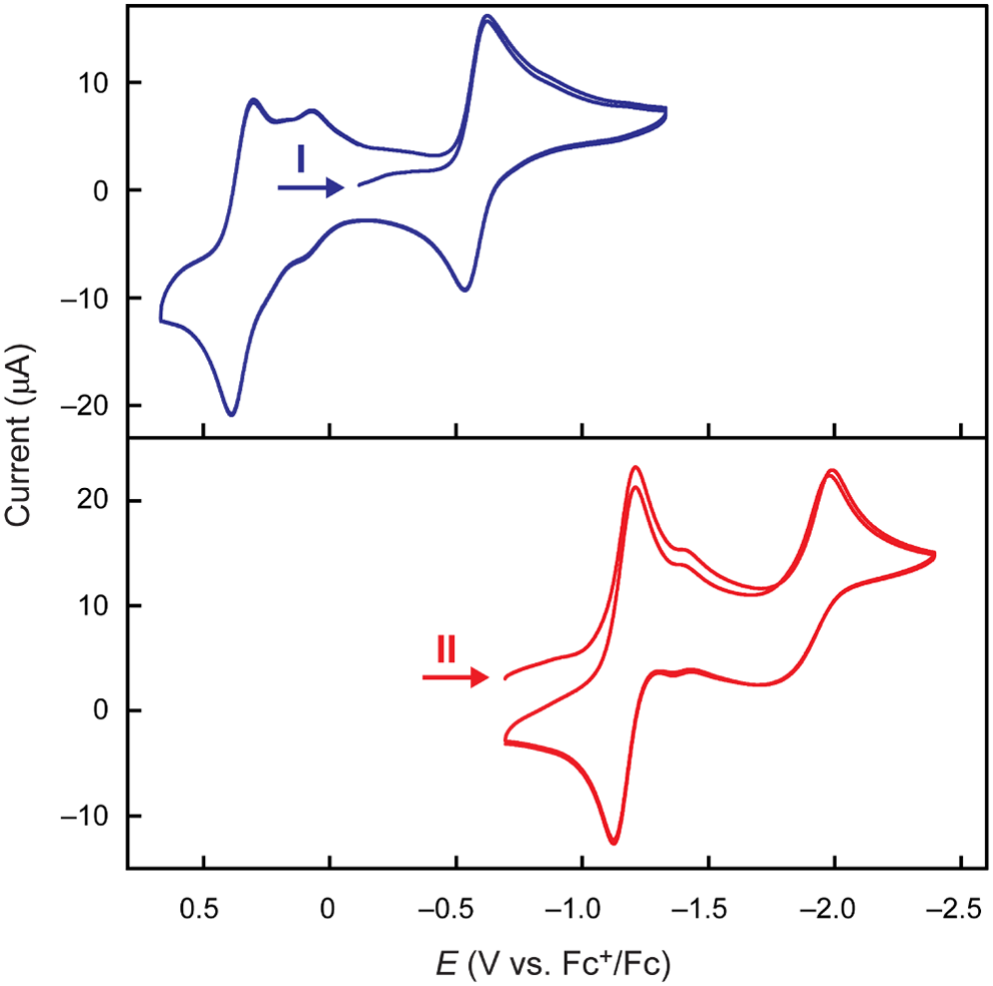
Cyclic voltammograms of **I** (blue) and **II** (red) in CH_3_CN. Conditions: 1.5 mM **I**-**II**, 0.1 M [^n^Bu_4_N][PF_6_], 2.5 mm glassy carbon working electrode, platinum wire counter electrode, silver wire reference electrode. 100 mV s^–1^ scan. Potentials are reported relative to the Fc/Fc^+^ couple.

Cyclic voltammetry measurements of **I** show a quasi-reversible reduction at E = –0.58 V relative to Fc^+^/Fc and a quasi-reversible oxidation at E = +0.35 V (Figure 3, top). A second, smaller cathodic wave is observed at E_pc_ = +0.07 V, which increases in current intensity upon scanning in the positive direction beyond an irreversible anodic feature at E_pa_ = +0.91 V (Figure S33). Further scanning in the reducing direction shows one additional irreversible cathodic wave at E_pc_ = –1.72 V. Accordingly, the feature at E_pc_ = +0.07 V is attributable to a minor species in solution, which is electrochemically generated in the cell by oxidation at E > +0.91 V. The isolation of multiple isomers of **II** (*vide infra*) suggests that five-coordinate **I** is fluxional and the energy landscape for the oxidized materials is relatively flat, so these additional features are likely explained by isomerism or ligand loss on oxidation.

### Synthesis and Characterization of [(^Ph^isq)(^Ph^ibq)V^IV^(O)Cl] (II)

**Figure 4:**
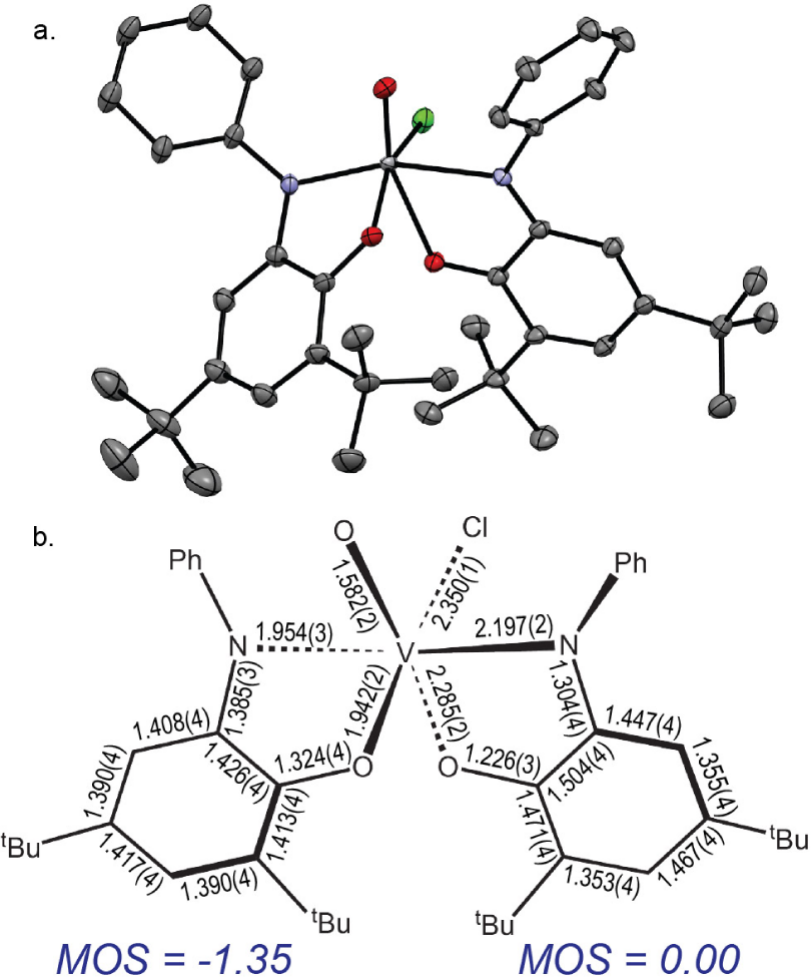
**a.** ORTEP plot of *trans*-[(^Ph^isq)(^Ph^ibq)V^IV^(O)Cl] (**IIa**). Ellipsoids drawn at 50% probability. **b.** Schematic of selected bond lengths (Å) for **IIa** and calculated metrical oxidation states^79^ for each ligand.

**Figure 5:**
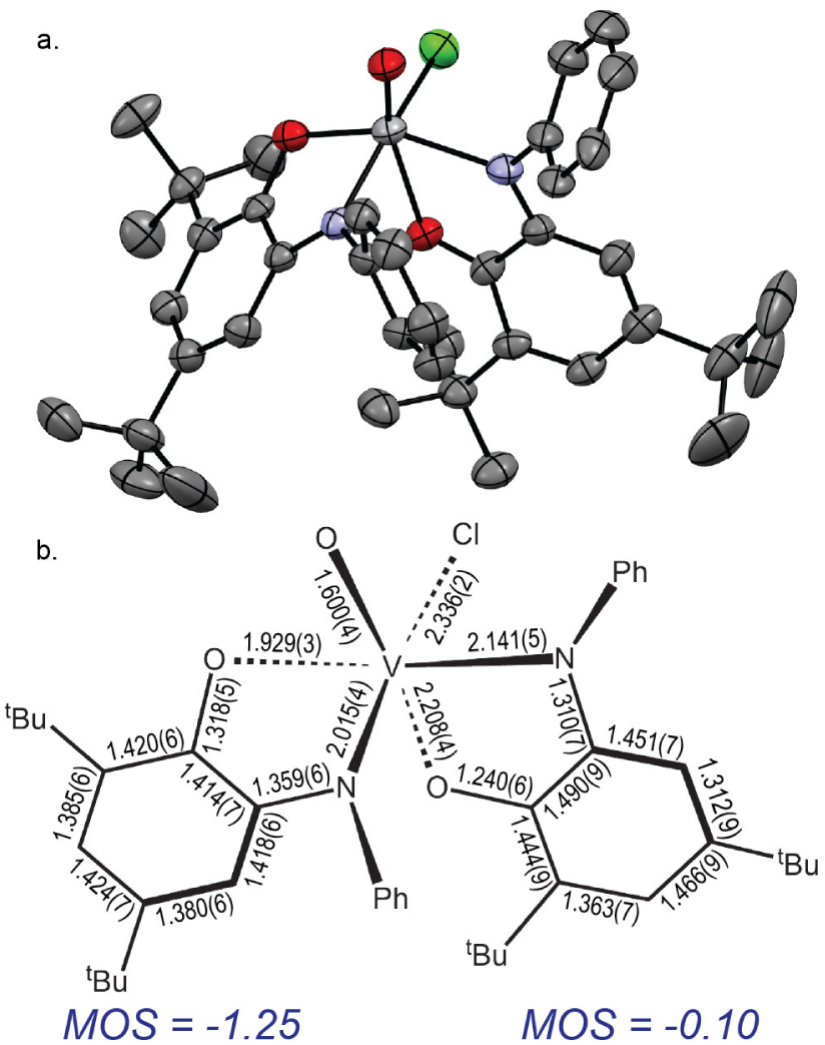
**a.** ORTEP plot of *cis*-[(^Ph^isq)(^Ph^ibq)V^IV^(O)Cl] (**IIb**). Ellipsoids drawn at 50% probability. **b.** Schematic of selected bond lengths (Å) for **IIb** and calculated metrical oxidation states^79^ for each ligand.

**Figure 6:**
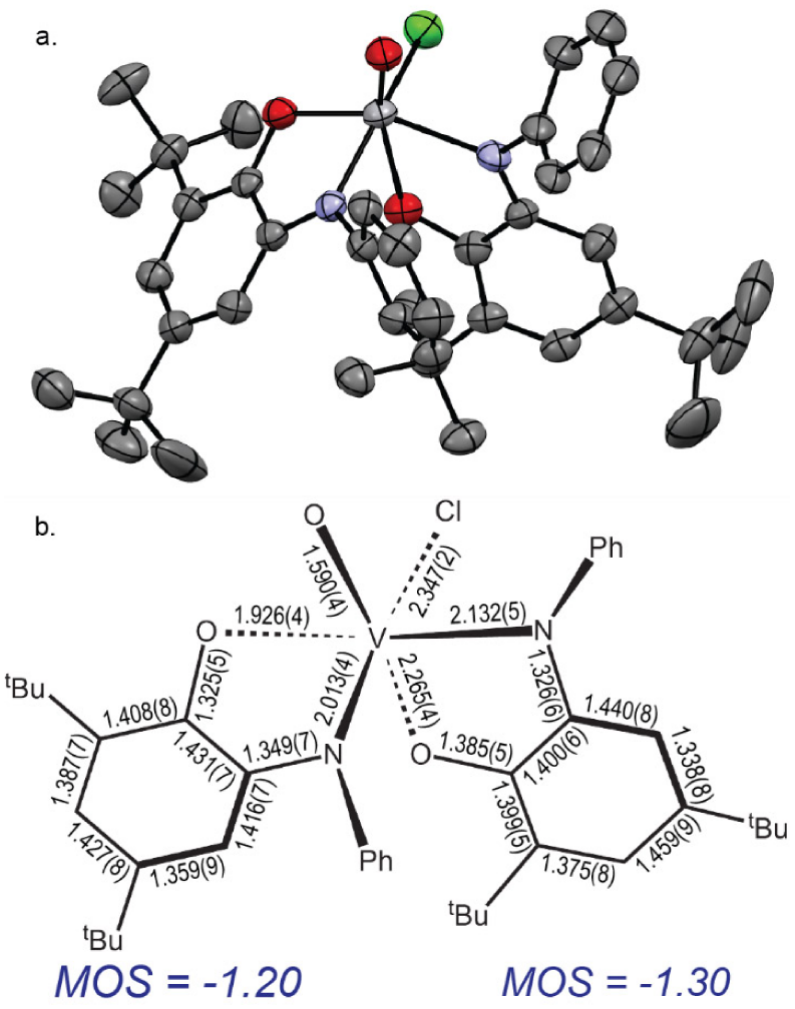
**a.** ORTEP plot of [(^Ph^isq)_2_V^V^(O)Cl] (**IIb’**). Ellipsoids drawn at 50% probability. **b.** Schematic of selected bond lengths (Å) for **IIb’** and calculated metrical oxidation states^79^ for each ligand.

Exposure of **I** to air gives a color change to red-brown **II** over 12 h at ambient temperature, with loss of the characteristic absorption band at 598 nm for **I** and concomitant growth of two new CT features at 326 and 464 nm (Figure S1). As in **I**, no CT bands are observed at wavelengths >1000 nm. Like **I**, **II** shows a complicated peak pattern in the aryl and *tert*-butyl regions of the ^1^H NMR spectrum indicative of a mixture of products (Figure S10). Slow evaporation of a concentrated benzene solution of **II** afforded X-ray quality single crystals of three structurally independent molecules, **IIa, IIb,** and **IIb’** (Figures 4–6). All are pseudo-octahedral; **IIa** and **IIb** differ in the relative orientations of the aminophenol ligands. In **IIa**, the N donors are in a *trans* disposition (θ_N–V–N_ = 161.7(1)°). The phenoxide O atoms occupy *cis* cites (θ_O–V–O_ = 79.35(8)°) *trans* to chloride (θ_O–V–Cl_ = 155.19(8)°) and oxido (θ_O–V≡O_ = 161.1(1)°) ligands, which complete the coordination sphere. The vanadyl V≡O distance of 1.582(2) Å is within the range of previously reported V^IV^(O) and V^V^(O) bonds (1.55–1.71 Å), which are valence-independent for the +4 and +5 oxidation states.^85,86^ The bond lengths within the aminophenol ligand *trans* to Cl are effectively unchanged, relative to **I**, but the V–O and V–N bond lengths to the aminophenol ligand *trans* to the oxo in **IIa** are lengthened by 0.372 (4) Å and 0.247(4) Å, respectively, and the C–C bond distances in the chelate ring exhibit a quinonoid-type, 4-long-2-short pattern characteristic of ligand oxidation (Figure 4b). Additionally, the V–O_phenoxide_ bond *trans* to the oxido ligand is 0.343(3) Å longer than the V–O_phenoxide_ bond *trans* to the chloride ligand, reflecting the stronger π donation from the oxido and/or weaker donation from the oxidized ligand. These differences are manifested in the computed MOS values^79^ of –1.35 and 0.00 for the ligands *trans* to Cl and O, respectively, implying a V oxidation state of +4.35. Accordingly, the simplest formulation for **IIa** in the solid state is [(^Ph^ibq)(^Ph^isq)V^IV^(O)Cl] ([^Ph^ibq] = 2,4-di*tert*-butyl-6-(phenylimino)benzoquinone), where the computed nonintegral oxidation state reflects V → ^Ph^ibq π backdonation from the d^1^ V(IV) center.^79,84^

Structures **IIb** and **IIb’** are crystallographically inequivalent structural isomers of **IIa** collocated in the same unit cell (Figures 5 and 6). The connectivity in **IIb** and **IIb’** is identical; both have the aminophenol N and O donors occupying mutual *cis* sites on the V center (θ_N–V–N_ = 91.8(2)°; θ_O–V–O_ = 82.9(1)°). The remaining sites are filled by a chloride *trans* to N (θ_N–V–Cl_ = 160.0(1)°), and an oxido *trans* to a phenoxide O (θ_O–V≡O_ = 165.1(2)°). The indicated bond angles for **IIb** are representative and statistically indistinguishable from those in **IIb’**. The vanadyl V–O bond lengths of 1.600(4) and 1.590(4) Å in **IIb** and **IIb’**, respectively, are also statistically identical, as are the bond lengths in the aminophenol ligand *trans* to Cl. However, statistically significant differences in the bond distances in the ligand *trans* to the oxo in **IIb** vs **IIb’** suggest electronic inequivalence. As in **IIa**, the V–O and V–N bond lengths of the aminophenol ligand *trans* to the oxido ligand in **IIb** are lengthened by 0.295(4) Å and 0.191(6) Å, respectively, relative to **I**, and the C–C bond distances show a quinonoid-type distortion. The computed MOS values^79^ for the ligands *trans* to Cl and O in **IIb** are –1.20 and –0.20, respectively.

In **IIb’**, the V–O_phenoxide_ bond *trans* to the oxo moiety is contracted by 0.057(6) Å, relative to **IIb**. The C–O_phenoxide_ bond in the ligand *trans* to the oxo in **IIb’** is 0.145(8) Å longer than in **IIb**. Collectively, these changes suggest a reduction in the ligand oxidation state in **IIb’**, which is manifested in the computed MOS^79^ value of –1.30 for the ligand *trans* to O and –1.20 for the ligand *trans* to Cl, giving an implied experimental oxidation state of +5.5 for V center in **IIb’**. Inspection of the extended crystal phase reveals that the phenoxide ring of **IIb’** is close to the chloride of **IIb** (d_Cl_..._C_ = 3.729(6) Å), within the range typical for solid-state halogen bonding,^87−90^ which apparently drives intramolecular charge transfer in the ground state via stabilization of an intervalence CT isomer. Accordingly, **IIb** and **IIb’** are redox isomers or valence tautomers, where the structural data for **IIb** are most consistent with a [(^Ph^ibq)(^Ph^isq)V^IV^(O)Cl] formulation, analogous to **IIa**, but the data for **IIb’** imply a [(^Ph^isq)_2_V^V^(O)Cl] formulation.

Cyclic voltammograms of **II** show a single quasi-reversible reduction centered at E = –1.17 V vs Fc^+^/Fc, and an irreversible reduction at E_pc_ = –2.00 V (Figure 3, bottom). Two small features centered at E = –1.39 V are attributed to a minor isomer. Three irreversible anodic features are observed between E_pa_ = +210 and +670 mV, prior to the onset of a large irreversible anodic feature at E_pa_ = +1.36 V (Figure S34). Chemical oxidation with tris(4-bromophenyl)ammoniumyl hexachloroantimonate (″magic blue″, E°’ = +0.67 V) yields purple crystals of 6,8-di*tert*-butyl-3H-phenoxazine-3-phenylazanylidinium hexachloroantimonate, which presumably results from the well-known demetalation and rearrangement of the oxidized ligand^91^ (see Figure S2 and crystal structure report).

Evans’ method solution magnetic moment^82^ measurements ranged from μ_eff_ = 1.1–2.3 μ_B_ (CDCl_3_, toluene-d_8_ C_6_D_6_, 25 °C) for independently prepared samples of **II** (Figures S17–S21), which are all substantially lower than the expected spin-only value for two unpaired electrons. A VT Evans’ method experiment showed a linear dependence of the magnetic moment on T^1/2^ from –50 °C to +50 °C (Figure S22).

A ^51^V NMR signal was observed for **II** at δ = –361 ppm in C_6_D_6_ solution (Figure S16). The broad peak (fwhm = 250 Hz) is typical of bulky low-symmetry complexes.^92,93^ Typical oxovanadium(V) complexes with exclusively *N*- and O- donors have chemical shifts between δ = –400 and –700 ppm,^93−95^ however coordination of redox-active ligands has been demonstrated to shift resonances downfield by up to hundreds of ppm.^96−98^ Additionally, substitution of an O-donor ligand for a chloride may produce a smaller such effect.^93,99^ Spin-coupled dinuclear V^IV^_2_ complexes show the same trends in chemical shift as d^0^ complexes.^92^ In summary, the ^51^V NMR data reflect only that some closed-shell complex is part of a potential mixture of triplet and singlet V(IV) or V(V) complexes in solution.

These possibilities were evaluated using unrestricted DFT calculations (PBE0, def2-TZVPP, D3BJ, RIJCOSX). Hydrogens-only optimizations of the crystal structures **IIa, IIb, and IIb’** were carried out in the singlet and triplet spin states. The results are summarized in Table 1. The lowest energy geometry and spin state was found to be the triplet state of **IIb**. The next-lowest calculated PBE0 energy is structure **IIa** in the singlet state, at +0.6 kcal/mol above triplet **IIb**. The singlet state of **IIb** lies +1.6 kcal/mol higher in energy than the triplet. The other three states, S = 1 **IIa** (+6.2 kcal/mol), S = 0 **IIb’** (+12.4 kcal/mol), and S = 1 **IIb’** (+14.8 kcal/mol), are significantly higher in energy. The gas phase energies of singlet **IIa**, singlet **IIb**, and triplet **IIb** are within the margin of error of DFT energetics.^100^ It is likely that all three are present in solution at room temperature, leading to the observed intermediate solution magnetic moment (*vide supra*). Both electron configurations of the valence tautomer **IIb’** are prohibitively high in energy in the gas phase, suggesting that the observation of the redox isomer in the solid state reflects crystal packing forces including halogen bonding, as discussed below.

**Table 1:** DFT Energies of Structures **IIa**, **IIb**, and **IIb’** in the Singlet and Triplet State Relative to the Lowest Energy Calculated State

Compound	E_S=0_ (kcal/mol)	E_S=1_ (kcal/mol)
**IIa**	+0.6	+6.2
**IIb**	+1.5	0.0
**IIb’**	+12.4	+14.8

**Figure 7:**
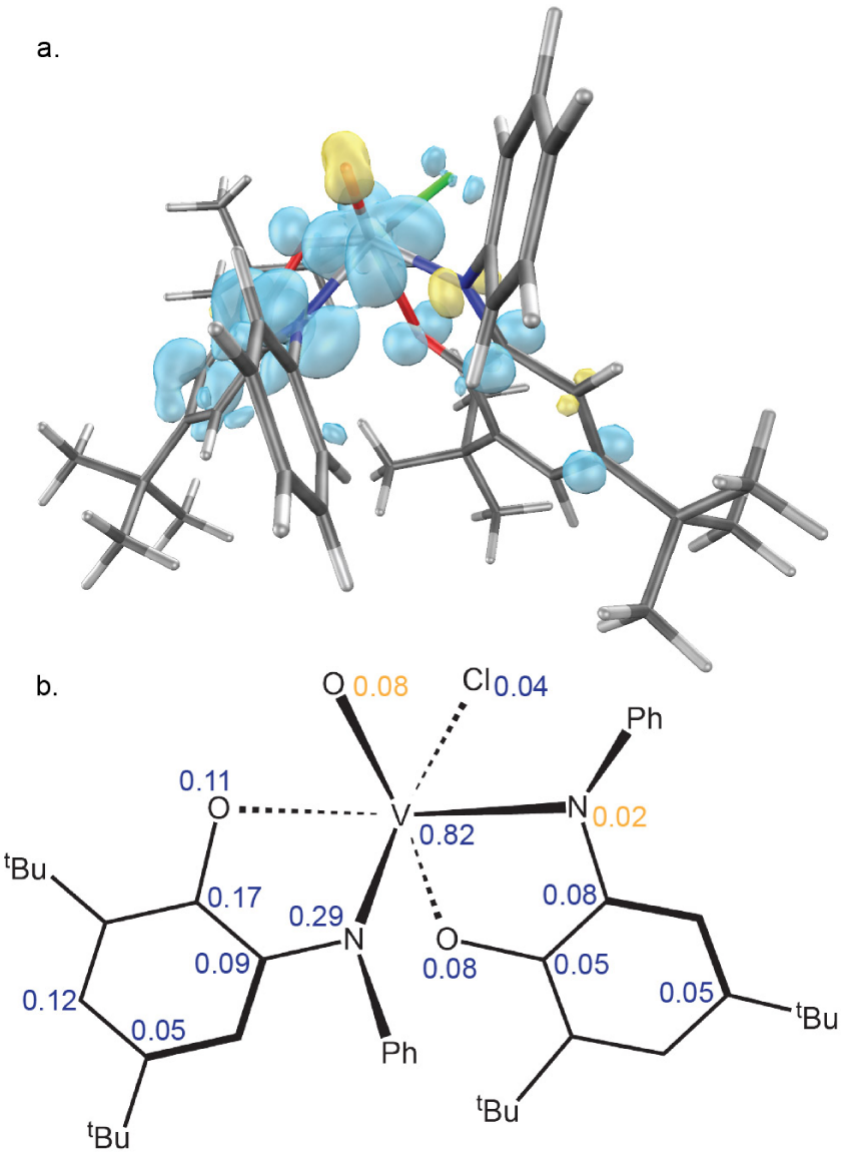
**a.** Spin density plot of S = 1 *cis*-[(^Ph^isq)(^Ph^ibq)V^IV^(O)Cl] (**IIb**) generated in IQmol (isosurface value 0.005). Spin down density is shown in yellow. **b.** Löwdin spin population per atom (*n* > 0.02).

**Figure 8:**
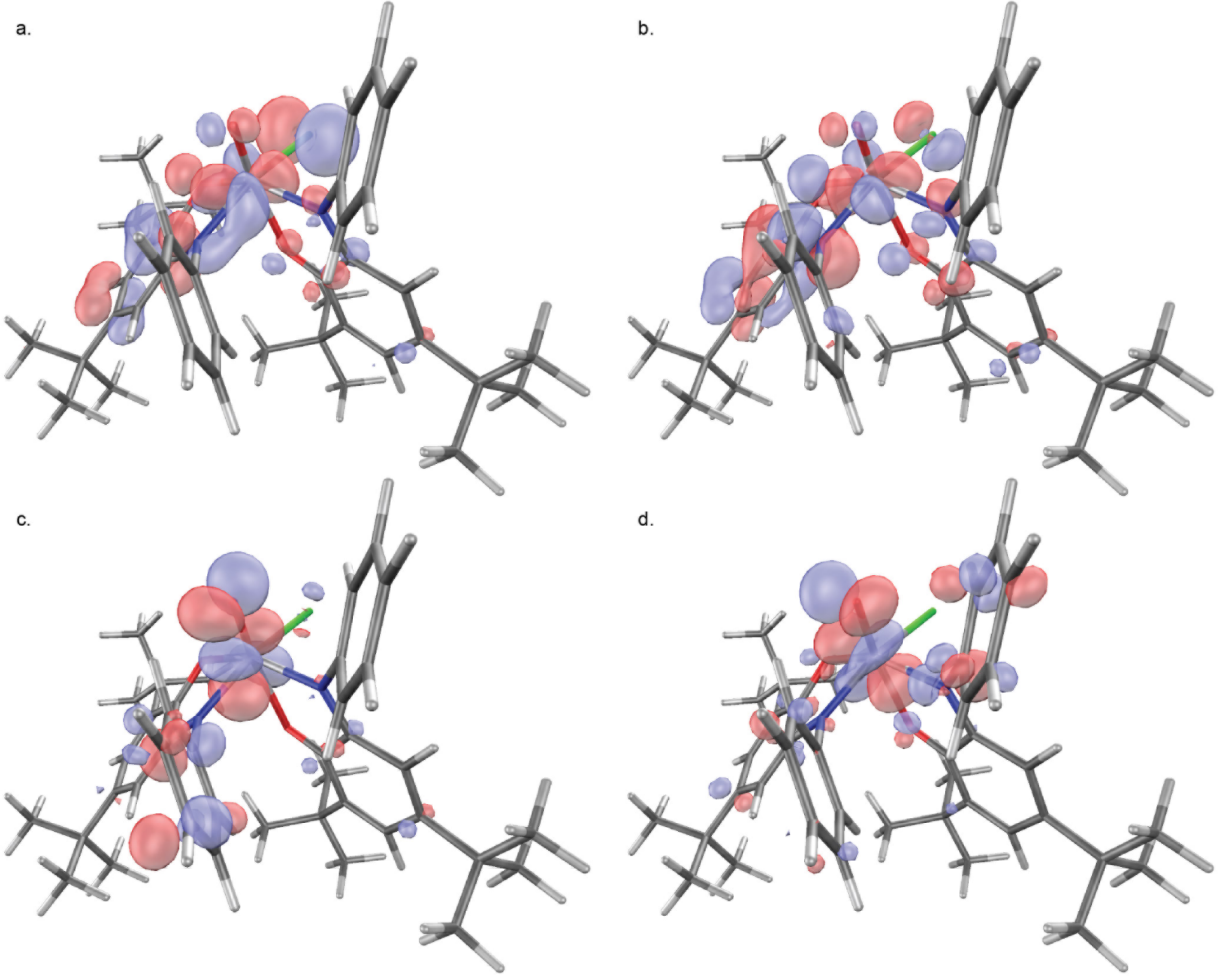
Selected DFT molecular orbitals of S = 1 *cis*-[(^Ph^isq)(^Ph^ibq)V^IV^(O)Cl] (**IIb**) generated in IQmol, isovalue 0.04. **a.** SOMO 1. **b.** SOMO 2 (HOMO). **c.** LUMO+1 **d.** LUMO+2.

The ground state triplet solution of **IIb** (⟨S^2^⟩ = 2.05) has more spin on the ligand *trans* to chloride than the ligand *trans* to the oxo (Figure 7), in agreement with their crystallographic assignment as [isq]^•–^ and [ibq]^0^, respectively. Significant spin down density (−0.08) is localized on the terminal oxo ligand due to spin polarization. Spin down density is also found on one of the nitrogen donors and two of the aromatic carbons. The calculated spin density on vanadium of 0.81, while less than in **I**, is most consistent with a d^1^ electron configuration. The lower energy SOMO has a contribution from the vanadium d_*xy*_ orbital (Figure 8a), which is π antibonding with respect to the V–Cl bond, π bonding with respect to an iminosemiquinonate π orbital *via* the V–N bond, and essentially nonbonding with respect to the vanadium-oxo bond. The second SOMO has a smaller contribution from the vanadium d_*xy*_ orbital and exhibits a π bonding interaction with an iminobenzoquinone π orbital via the nitrogen donor (Figure 8b). This observation of π backdonation from the occupied vanadium d_*xy*_ orbital to the [ibq]^0^ ligand corroborates the crystallographic assignment as d^1^ V(IV). The LUMO is primarily vanadium d_*xy*_-centered and is π antibonding with respect to both nitrogen donors (Figure S44). The LUMO+1 and LUMO+2 are the canonical vanadium oxo π* interactions in the xz and yz planes (Figure 8c,d).^32^

**IIa** converged as a singlet (⟨S^2^⟩ = 0.00). The HOMO qualitatively resembles the lower-energy SOMO in S = 1 **IIb** and has a contribution from the vanadium d_*xy*_ orbital (Figure S44). It is again π* with respect to the V–Cl bond, π bonding to a nitrogen p orbital, and nonbonding to the oxo. This orbital likewise shows the vanadium–iminosemiquinonate covalency, where two electrons occupy a bonding orbital with significant density on the metal and ligand, agreeing with the crystallography-based assignment as d^1^ vanadium(IV). The vanadyl π* orbitals appear with the same composition and ordering as S = 1 **IIb** (Figures S48 and S49). The singlet solution of **IIb** (⟨S^2^⟩ = 0.00) qualitatively resembles S = 1 **IIb**. The shapes and ordering of the orbitals are unchanged, the only difference being that the lower energy SOMO is now doubly occupied and the higher energy SOMO is the LUMO (Figures S50–S54).

Full optimizations of **IIa** in implicit benzene solvent (CPCM) show the same compression of the singlet/triplet gap, as observed in **I**, to 1.0 kcal/mol. However, the calculated solution electronic structure of **IIa** is unambiguously vanadium(IV), with 0.97 and 0.98 unpaired electrons on the metal in the S = 0 and S = 1 states, compared to 0.95 in the solid state (Table S26).

Finite-temperature DFT calculations (PBE0, def2-TZVPP, D3BJ, RIJCOSX, 10000K) of **IIa** and **IIb** each returned a large N_FOD_,^101^ 2.62 and 2.74 respectively, and visualization of the FODs revealed that they are distributed across both redox-active ligands as well as the vanadium center (Figures S55 and S56). **IIa** returned 13 orbitals with occupations between 0.02 and 0.98, and **IIb** returned 11 such orbitals. These data indicate that a multiconfigurational ground state is likely in both isomers of **II**. Moreover, the sensitivity of the electronic structures to subtle perturbations in bond lengths again highlights the need for caution in extrapolating the solid state electronic structural assignments to homogeneous solution.

### Oxygen-Atom Transfer Reactions

**Scheme 2:**
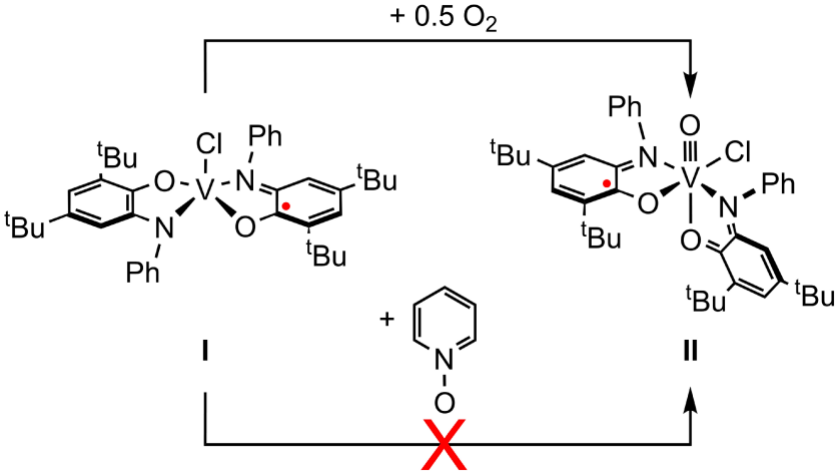
O-Atom Transfer to **I** to Generate **II**

Conversion of **I** to **II** is balanced by addition of one oxygen atom. As detailed above, exposure of **I** to O_2_ results in conversion to **II** in 87% yield (Table 2, entry 1). However, treatment of **I** with the pyridine-N-oxide or trimethylamine-N-oxide gave no evidence for **II** up to its decomposition temperature of 120 °C (Table 2, entries 2–3; Scheme 2).

**Table 2:** Thermodynamics of X–O Bonds Broken or Formed in Reactions with **I** or O-Atom Transfer to I to Generate II in Benzene

entry	II + X		I + X–O	ΔH_f_X–O (kcal/mol)
1	0.5 O^a^	←	0.5 O2	–59.6^102^
2	Me_3_N^b^		Me_3_NO	–61.6^41^
3	C_5_H_5_N^b^^c^		C_5_H_5_NO	–72.2^102^
4	Me_2_S^c^	→	Me_2_SO	–86.7^102^
5	9,10-DHA^c^	→	H_2_O + C_14_H_10_	–100.1^103^
6	AsPh_3_^a^	→	AsOPh_3_	–102.7^102^
7	Me_2_SO^c^	→	Me_2_SO_2_	–112.3^102^
8	PPh_3_^a^	→	OPPh_3_	–133.4^102^

a 25 °C.

b No reaction was observed in either direction.

c 50 °C.

**Scheme 3:**
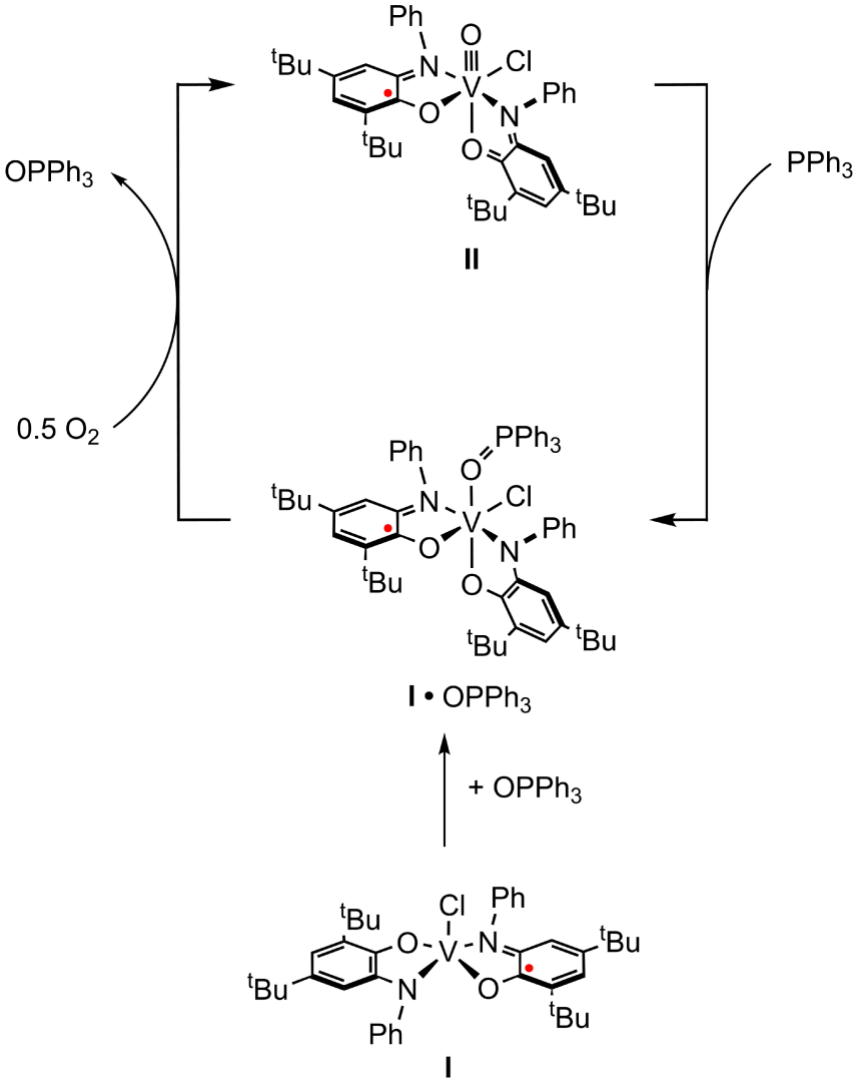
Reaction of **II** with PPh_3_ Gives **I**•OPPh_3_, Which is Independently Generated by the Reaction of **I** with OPPh_3_

Addition of 1 equiv of PPh_3_ to a C_6_D_6_ solution of **II** gave a solution with a single ^31^P NMR resonance at 42.7 ppm, which does not match either PPh_3_ or OPPh_3_ (Scheme 3, Figure S23), and is consistent with an OPPh_3_–Lewis acid adduct.^104^ Exposure of the solution to air gave clean conversion to a solution with a single resonance at 25.3 ppm, which matches the literature value for free OPPh_3_ (Figure S24). The UV–vis spectrum of the reaction of **II** + PPh_3_ matches the spectrum of **I**, with an additional low-intensity absorbance at 460 nm and a shoulder at 750 nm (Figure S3). These features are ascribed to binding a sixth ligand to **I**. Addition of 1 equiv OPPh_3_ to isolated **I** reproduces the ^31^P NMR spectrum with the resonance at 42.7 ppm and generates a UV–vis spectrum that matches that observed in the reaction of **II** with PPh_3_ (Figures S25, S3). These results suggest that the initial product of the reaction of **II** with PPh_3_ is [(^Ph^ap)(^Ph^isq)V(OPPh_3_)Cl], containing a triphenylphosphine oxide in the primary coordination sphere, which is displaced upon reoxidation with O_2_ (Table 2, entry 8; Scheme 3).

**Scheme 4:**
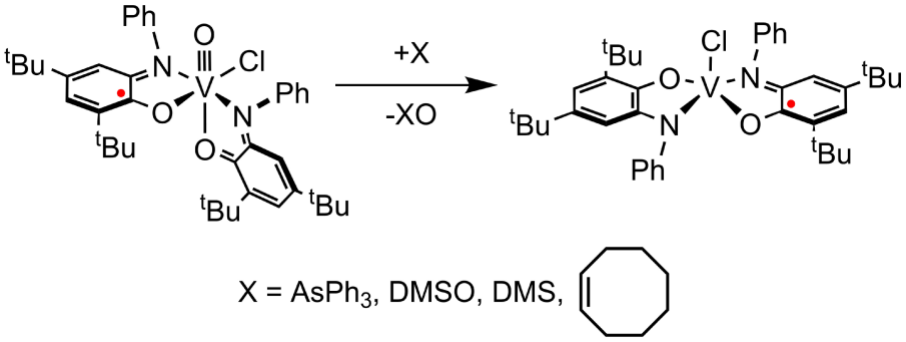
Oxidation of Main-Group Nucleophiles by **II**

Analogous reactions of **II** with 1 equiv of AsPh_3_, dimethyl sulfoxide (DMSO), or dimethylsulfide (DMS) were performed in C_6_D_6_ (Table 2, entries 4, 6, 7; Scheme 4). In all cases, the UV–vis spectrum matched that expected for the adducts **I**•OX, as described above (Figures S4–S6). The ^1^H NMR spectrum of the DMSO reaction shows consumption of DMSO and contains a broad singlet at 1.76 ppm, which does not match DMS, DMSO, or DMSO_2_ (Figures S26–S28), and aliphatic signals associated with the vanadium complex appear at 1.67 and 1.15 ppm, which do not match the precise shifts of **I** or **II** (Figure S28). Two aliphatic signals associated with **II** are retained at 1.02 and 0.82 ppm and are attributed to unreacted **II**. The sum of these data indicate formation of **I**•DMSO_2_ with some unreacted **II** potentially reflecting an unreactive minor isomer. Attempts to observe DMS oxidation products by ^1^H NMR showed the same singlet at 1.76 ppm seen in the products of the DMSO reaction (Figure S27), implying that **II** was fully consumed to oxidize half an equivalent of DMS to DMSO_2_ because of the relatively stronger driving force for the second oxidation.

Adding 8 equiv of *cis-*cyclooctene to a CDCl_3_ solution of **II** resulted in conversion to *cis*-cyclooctene oxide as shown by ^1^H and ^13^C NMR (Scheme 4, Figures S29 and S30). An epoxide yield of 90% based on limiting **II** was estimated by integration of the ^1^H NMR spectrum.

**Scheme 5:**
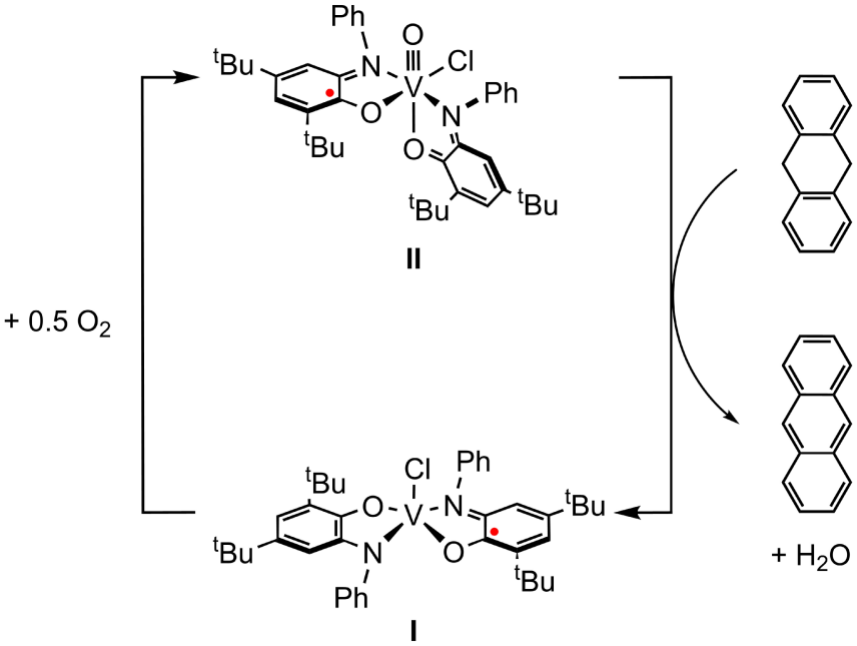
Oxidase-type DHA Catalysis via Aerobic Cycling of **I** and **II**

Reaction of **II** in C_6_D_6_ with 1 equiv 9,10-dihydroanthracene (DHA) at room temperature resulted in partial conversion to anthracene over 16 h, as evidenced by ^1^H NMR (Table 2, entry 5). Heating to 50 °C overnight effected a color change to blue, and the resulting UV–vis spectrum matched **I** and the diagnostic peaks for anthracene (Figure S7). As in the reaction of **II** with PPh_3_ (vide supra), a low-intensity feature at 460 nm and a shoulder at 750 nm in the UV–vis indicate coordination of a sixth ligand, which in this case is the H_2_O generated by net H_2_ transfer to the oxo from DHA. ^1^H NMR demonstrated the consumption of DHA and production of anthracene, and a broad singlet at 0.5 ppm is attributed to the bound aquo ligand (Figure S31). Heating a C_6_D_6_ solution of **II** and 10 equiv of DHA to 50 °C overnight while open to air also effected complete consumption of DHA and produced ^1^H NMR signals for anthracene and a distinct H_2_O peak (Scheme 5, Figure S32).

The sum of the thermodynamic data collected in Table 2 permit an estimation of the V–O BDE in **II** at 73 ± 14 kcal mol^–1^,^102^ which implies a diminution of at least 50 kcal mol^–1^ relative to the previously reported V≡O bonds in classic, redox-innocent ligand vanadyl complexes.^48^ The origins of this remarkable weakening and implications for aerobic oxidations are discussed below.

## Discussion

### Electronic Structures of Exceptionally High-Valent Vanadyl Complexes

The electronic structures of the complexes reported herein are dominated by covalency in the vanadium–ligand bonding and the accessibility of low energy spin states, including multiconfigurational ground states, which defy simple models and oxidation state formulations. These challenges are exacerbated by the capacity of the [ap]^2–^/[isq]^•–^/[ibq]^0^ ligand series to function as both π donors and π acceptors, switching from primarily π donor to acceptor with increasing ligand oxidation state.^79^

This ambiguity is evinced in **I**, which is best formulated in the solid state as a d^1^ V(IV) center with a second unpaired spin delocalized across the π systems of the two aminophenol ligands. The ligand spin couples antiferromagnetically with the spatially separated spin on V, leading to an admixture of singlet and triplet ground states. The sum of the computed MOS values^79^ for the two aminophenol ligands of –2.60 is significantly less than the –3.0 expected for [(^Ph^ap)(^Ph^isq)V^IV^Cl] but is inconsistent with alternative V(III) or V(V) formulations. The implied noninteger V oxidation state of +3.6 is therefore rationalized by π donation from the [^Ph^ap]^2–^ ligand, which reduces the net charge on V in **I** to below that indicated by the formula. In this context, computations suggesting an accessible [(^Ph^isq)_2_V^III^Cl] valence tautomer in solution are intriguing but challenging to assess or benchmark experimentally, particularly given the accessibility of multiple spin states at ambient temperatures.

The observation of three distinct structural and electronic isomers of **II** in the solid-state data is atypical and suggests the energy landscape in this region is relatively flat. **IIa** and **IIb** are structural isomers that vary in the relative (*trans* vs *cis*) disposition of the aminophenol ligands. Structural and computational data suggest both are best formulated as [(^Ph^ibq)(^Ph^isq)V^IV^(O)Cl], but this is an oversimplification. The iminobenzoquinone ligand is a π acceptor, leading to a computed metrical oxidation state for V of slightly more than +4 in both **IIa** and **IIb**, and the bonding in the complexes is highly covalent with significant delocalization of the spin across the metal and ligands. Computations suggest that **IIa** and **IIb** have different ground spin states—the former is a singlet; the latter is a triplet—but the gas phase energies of singlet **IIa**, singlet **IIb**, and triplet **IIb** are all within 1.5 kcal mol^–1^ and functionally indistinguishable by DFT. Accordingly, the ground states are most likely multiconfigurational with significant singlet/triplet mixing, and we caution against overinterpretation of oxidation state formalisms that likely have little bearing on the true electronic structures of **II**.

Against this backdrop, the observation of a valence tautomer of **IIb** in the solid state is surprising and merits further discussion. **IIb** and **IIb’** are not bond stretch isomers, of the type that have historically beguiled coordination chemists.^105,106^ The V≡O bond distances in **IIb** and **IIb’** are statistically indistinguishable, and the X-ray data refinement is not improved by solution of **IIb’** as a dichloro complex. Rather, **IIb** and **IIb’** are valence tautomers, which vary in the distribution of spin across the vanadium–ligand framework, with **IIa** exhibiting structural features more consistent with [(^Ph^ibq)(^Ph^isq)V^IV^(O)Cl] and **IIb’** being better described as [(^Ph^isq)_2_V^V^(O)Cl]. The capacity of such redox-active ligand complexes to undergo low energy intravalence CT in response to subtle environmental perturbations, including changes in temperature, pressure, solvent dielectric, or visible light irradiation have been a subject of study since at least 1980.^107−110^ To our knowledge, there are no published examples of two redox isomers cocrystallizing, but the accessibility of two minimum energy isomers is well precedented. It strikes us as entirely plausible that such a transition could be similarly driven by crystal packing forces, in this case the intermolecular halogen bonding described above. The computed energies of **IIb’** are 12–14 kcal mol^–1^ above **IIb**, making the V(V) isomer thermally inaccessible in solution at the reaction temperatures utilized in these studies. Accordingly, the solution structure of **II** is most likely a mixture **IIa** and **IIb**.

### Redox-Active Ligand-Mediated O_2_ Activation and O-Atom Transfer

Ligand-centered redox and covalency in the vanadium–aminophenol bonding in **I** and **II**, as established above, are essential features of the observed O-atom transfer reactivity. The metal center in **II** has significantly less electron density than in **I**, but formulation of **II** as V(IV) implies that O-atom addition to **I** is a redox neutral process at the V center. Accordingly, the net O-atom addition is appropriately described as an oxidative addition at the [(^Ph^ap)(^Ph^isq)V^IV^] core, where ligand-centered redox gives access to a net 2e^–^ reaction that is otherwise impossible at a monometallic d^1^ center.

Spontaneous generation of 2 equiv **II** from O_2_ + 2 equiv **I** establishes a lower limit for the V≡O bond in **II** at one-half the strength of the O=O bond in dioxygen (59.8 kcal mol^–1^).^102^ Indeed, the vanadium–oxo bond in **II** is remarkably weak. Reported V≡O BDEs in vanadyl complexes typically fall in the range of 120–170 kcal mol^–1^.^48^ A Holm-type thermodynamic bracketing^40^ via reactions with O-atom donor and acceptor substrates returns a V≡O BDE of 73 ± 14 kcal mol^–1^ in **II**. The origin of this weakening is not attributable to a destabilization of the vanadyl functionality. The V≡O bond in **II** is on the shorter end of those typical for vanadyls.^85,86,111^ The d_*xy*_^1^ configuration and the shape and ordering of the V≡O (anti)bonding orbitals exactly mirror those elaborated by Ballhausen and Gray for the canonical V≡O triple bonds.^32,112^ Indeed, the V center in **II** is highly electron deficient, which should strengthen the bond to the terminal [O]^2–^ ligand. Rather, the astonishingly weak V≡O bond in **II** is a function of the stability of the reduced product **I**. O-atom transfer from a V(V) or V(IV) oxo complex would typically occur with 2e^–^ reduction of the metal center. Accordingly, the thermodynamic bias for high vanadium +4 and +5 formal oxidation states in classic, redox-innocent Werner complexes significantly raises the energy of the deoxygenated products. Here, the reduced product is energetically stabilized by shifting the locus of the reduction to the redox-active ligands, which permits the net 2e^–^ reduction to occur without a reduction of the V(IV) center. Thus, the intrinsically weak V≡O bond is not only a function of the V–O bonding itself but of the relative stability of the reduced, deoxygenated V product. While two sides of the same coin, this highlights the need to consider the product stability in efforts to generate activated metal–oxo species for applications in small molecule oxidations.

O_2_ homolysis by 2 equiv **I** to make 2 equiv **II** is exothermic by no more than 54 kcal mol^–1^ and, per the computed gas phase V–O BDE of 60 kcal mol^–1^, when the product is **I**•THF, approximately thermoneutral. The relatively large uncertainty in the experimentally determined V–O BDE of 73 ± 14 kcal mol^–1^ in **II** reflects the fact that **I** is unreactive with even weak O atom donors such as pyridine N-oxide or trimethylamine N-oxide, which we ascribe to kinetic rather than thermodynamic reasons. Preliminary data supports a mechanism of bimetallic O_2_ homolysis proceeding via a mononuclear V(O_2_) intermediate, which bears resemblance to our previous report of bimetallic O_2_ homolysis at bis(amidophenolato) and bis(catecholato) oxorhenium(V) centers.^73^ In that study, ligand-centered 1e^–^ redox was shown to lower the barrier to a O_2_ homolysis by giving access to superoxo and peroxo intermediates. Accordingly, the capacity of the redox-active ligand to mediate 1e^–^ reactions with O_2_ was essential for its binding and reduction, thereby lowering the kinetic barrier for O_2_ cleavage.^29,73,77,78^ In this context, the disparate reactivity with O_2_ and amine-N-oxides likely reflect a kinetic bias for odd-electron reactivity with O donors during formation of the V–O bond rather than thermodynamically uphill N→O bond heterolysis.

Separation of the site of bond making and breaking, here the V center, from the locus of oxidation for atom or group transfer is a concept with ample precedent. Brown and coworkers have described so-called ″nonclassical oxygen-atom transfer″, such as oxidations of oxomolybdenum(VI) complexes containing redox-active bis(3,5-di*tert*-butylcatecholate) by pyridine–N-oxide, which afford dioxomolybdenum products in impossibly high formal oxidation states.^74,75^ Heyduk and coworkers have reported redox-active ligand-mediated multielectron nitrene transfer cycling at d^0^ Zr and Ta centers.^57,113,114^ However, subsequent 2e^–^ atom or group transfer to olefins, or C–H oxidations, by the high-valent products of these reactions has proven to be elusive. Indeed, there is a rich history of O-atom transfer to low valent organometallic Group 4 and Group 5 metals,^40^ and such species are notoriously air and moisture sensitive, but the oxido products of these reactions are typically thermodynamic minima and essentially inert. To our knowledge, the capacity of **I** to cleanly generate **II** by O_2_ homolysis, and the ability of **II** to subsequently regenerate **I** via O-atom transfer to small molecule acceptors or C–H dehydrogenation, are without precedent in this part of the periodic table.

## Conclusions

The atypically weak V–O bonds in **II** provide a strong thermodynamic driving force for oxidations of weak O-atom acceptors and strong X–H bonds. The aerobic oxidations described herein establish proof of principle for the utility of **II** in dioxygenase-type redox cycles. Accordingly, the power of the approach elaborated herein is not in the organic oxidation products themselves, but in illuminating a pathway for extending dioxygenase activity to early metals. For example, extensive efforts to functionally mimic enzymatic heme and nonheme oxygenase-type C–H oxidations have yielded copious small molecule Fe catalysts, including some with the capacity to activate and functionalize strong C–H bonds.^12,25,26,42^ However, the most active systems use peroxides as O_2_ surrogates, and synthetic aerobic dioxygenases are still rare, and to our knowledge, entirely unknown at early metals outside this report.

The vanadyl complexes reported herein exhibit reactivity typical of late metal–oxo species. This umpolung can be ascribed to both thermodynamic and kinetic factors, which are a direct result of ligand redox noninnocence and covalency in the bonding between the vanadium and aminophenol-derived ligands. **II** is electrophilic at O and a strong oxidant. However, unlike later 3d metals, where M=O species are typically high energy and activated by, for instance, occupancy of M–O π* antibonding MOs, the exceptionally weak V≡O bonds in **II** result from stabilization of the reduced products. The modest 1e^–^ reduction potential of **II** (E = –1.17 V vs Fc^+^/Fc) belies its potency as an oxidant. Thus, this research highlights an alternative pathway to generating strong O atom donors that are not strong outer-sphere electron acceptors.

The mechanisms of the O_2_ activation and C–H activation are a subject of ongoing investigation, but it seems reasonable to expect that the accessibility of multiple structural and electronic isomers, and the multiconfigurational ground states in **I** and **II**, provide lower energy pathways to traverse in spin forbidden reactions with the small molecule substrates. Extensions to aerobic alkyl C–H hydroxylations and dehydrogenations are a focus of ongoing work in our lab.

## Experimental Section

### General Considerations

Unless otherwise mentioned, all operations were carried out under anaerobic conditions using standard vacuum line techniques or in an inert-atmosphere glovebox under nitrogen. NMR spectra were recorded on a Bruker Avance III 400 MHz instrument, a Bruker Avance III 500 MHz instrument with a Prodigy cryoprobe, or a Varian mercury 300 MHz instrument, and are labeled as such. ^1^H chemical shifts are reported in parts per million (ppm) relative to tetramethylsilane (TMS), with the residual solvent peak as an internal reference. ^31^P chemical shifts (ppm) are reported relative to a pseudointernal standard of phosphoric acid. ^51^V chemical shifts (ppm) are reported relative to an external standard of VOCl_3_. Solution magnetic moments were obtained by Evans’ NMR method.^82^ UV–vis absorption spectra were acquired on an Agilent 8453 DAD spectrophotometer, recorded at ambient temperature in a 1 cm quartz cell. UV–vis-NIR absorption spectra were acquired on a Hitachi 4150 spectrophotometer, recorded at ambient temperature in a 1 cm quartz cell. Cyclic voltammetry experiments were performed inside an N_2_-filled glovebox in acetonitrile with 0.1 M [(^n^Bu)_4_N][PF_6_] as the supporting electrolyte, unless otherwise noted. The voltammograms were recorded with a CH Instruments 620C potentiostat, using a 2.5 mm glassy carbon disk working electrode, Ag wire quasi-reference electrode, and a Pt wire auxiliary electrode, at a scan rate of 0.1 V s^–1^, unless reported otherwise. Reported potentials are referenced to the ferrocenium/ferrocene (Fc^+^/Fc) redox couple, added as an internal standard at the conclusion of each experiment. Elemental analyses were performed by Atlantic Microlab, Inc., Norcross, GA. All analyses were performed in duplicate, and the reported compositions are the averages of the two runs. Full details of X-ray data collection and refinement are provided in the Supporting Information.

### Materials and Methods

Anhydrous acetonitrile (CH_3_CN), dichloromethane (CH_2_Cl_2_), pentane, tetrahydrofuran (THF), toluene, and hexane were purchased from Sigma-Aldrich and further dried by passage through columns of activated alumina, degassed by at least three freeze–pump–thaw cycles, and stored under N_2_ prior to use. Anhydrous benzene (Drisolv) was purchased from EMD Millapore, transferred to a sealable flask, degassed by three freeze–pump–thaw cycles, and stirred over sodium overnight with benzophenone as an indicator. The benzene was then vacuum transferred to a flame-dried sealable flask and stored under N_2_ prior to use. Deuterated solvents were purchased from Cambridge Isotope Laboratories, degassed by three freeze–pump–thaw cycles, and stored over 3Å molecular sieves under N_2_ prior to use. Naphthalene (Sigma-Aldrich) was sublimed under vacuum and stored under N_2_ prior to use. Tetramethylsilane (Acros), pyridine (Drisolv), *cis*-cyclooctene (Fisher Scientific), dimethyl sulfide (Beantown), and dimethyl sulfoxide (Sigma-Aldrich), were degassed by three freeze–pump–thaw cycles and stored over 3Å molecular sieves under N_2_ prior to use. Potassium Hydride (Sigma-Aldrich) was washed with pentane and stored under N_2_ prior to use. Vanadyl Chloride (Strem), sodium (Sigma-Aldrich), triphenylphosphine (Strem), triphenylphosphine oxide (Sigma-Aldrich), triphenylarsine (Sigma-Aldrich), 9,10-dihydroanthracene (Sigma-Aldrich), pyridine N-oxide (Sigma-Aldrich), trimethylamine N-oxide (TCI), tris(4-bromophenyl)aminium hexachloroantimonate (Sigma-Aldrich) were stored under N_2_ prior to use. The ligand 2-anilino-4,6-di*tert*-butylphenol was prepared according to a published procedure.^115^ Catechol (TCI), tert-butanol (Beantown), benzene (Sigma-Aldrich), sulfuric acid (BDH), triethylamine (Alfa Aesar), aniline (Alfa Aesar), heptane (BDH), and hexanes (BDH) for these reactions were used as received.

### Synthesis of [(^Ph^ap)(^Ph^isq)V^IV^Cl] (I)

2-anilino-4,6-di-*tert*-butylphenol (600 mg, 2 mmol), was dissolved in THF (4 mL) and stirred over potassium hydride (480 mg, 12 mmol) for 30 min. This solution was then filtered through a glass microfiber filter and added to a stirring solution of VOCl_3_ (200 mg, 1.1 mmol) in THF (1 mL). The reaction was stirred for 2 h before removal of the solvent in vacuo, then extracted with benzene (80 mL) and filtered through Celite, leaving behind both light and dark insoluble solids. The benzene was evaporated to give **I** as a blue solid (85%). UV–vis (benzene) λ_max_, nm (ε, M^–1^ cm^–1^): 294 (15000), 602 (9300). UV–vis (THF) λ_max_, nm (ε, M^–1^ cm^–1^): 289 (12000), 598 (9900), 923 (2400). Anal. Calc. for C_40_H_50_ClN_2_O_3_V: C, 69.30; H, 7.27; N, 4.04; Found: C, 69.09; H, 7.37; N, 3.93.

### Synthesis of [(^Ph^isq)(^Ph^ibq)V^IV^(O)Cl] (II)

A benzene solution of **I** (289 mg) was stirred overnight in air atmosphere. The solution was decanted, and the solvent was removed to give **II** as a brown solid (99%). UV–vis (benzene) λ_max_, nm (ε, M^–1^ cm^–1^): 273 (16000), 326 (13000), 464 (12000). Satisfactory elemental analysis required the inclusion of 0.5 equiv of benzene, present in the single-crystal X-ray structure. Anal. Calc. for C_43_H_53_ClN_2_O_3_V: C, 70.53; H, 7.30; N, 3.83; Found: C, 70.86; H, 7.22; N, 3.99.

### Reaction of [(^Ph^isq)(^Ph^ibq)V^IV^(O)Cl] with *Tris*-(4-Bromophenyl)aminium Hexachloroantimonate

A glass vial was charged with [(^Ph^isq)(^Ph^ibq)V^IV^(O)Cl] (14 mg, 0.02 mmol), tris(4-bromophenyl)aminium hexachloroantimonate (16 mg, 0.02 mmol), and toluene (2 mL), effecting an immediate color change to deep purple. Crystals of 6,8-di*-tert*-butyl-3H-phenoxazine-3-phenylazanylidinium hexachloroantimonate for X-ray diffraction were grown by slow evaporation from toluene solution.

### Reaction of [(^Ph^isq)(^Ph^ibq)V^IV^(O)Cl] with 9,10-Dihydroanthracene

[(^Ph^isq)(^Ph^ibq)V^IV^(O)Cl] (9.8 mg, 0.014 mmol) and 9,10-dihydroanthracene (2.5 mg, 0.014 mmol) were dissolved in C_6_D_6_ and sealed under N_2_ in a J. Young style NMR tube. The tube was heated to 50 °C in an oil bath for 20 h.

### Reaction of [(^Ph^isq)(^Ph^ibq)V^IV^(O)Cl] with Triphenylphosphine

[(^Ph^isq)(^Ph^ibq)V^IV^(O)Cl] (13.8 mg, 0.02 mmol) and triphenylphosphine (4.6 mg, 0.02 mmol) were dissolved in C_6_D_6_ (600 μL) and sealed under N_2_ in a J. Young style NMR tube. After collecting NMR and UV–vis spectra, the tube was opened to air and allowed to sit at room temperature overnight.

### Reaction of [(^Ph^ap)(^Ph^isq)V^IV^Cl] with Triphenylphosphine Oxide

[(^Ph^ap)(^Ph^isq)V^IV^Cl] (13.4 mg 0.02 mmol) and triphenylphosphine oxide (5.6 mg, 0.02 mmol) were dissolved in C_6_D_6_ (600 μL) and sealed under N_2_ in a J. Young style NMR tube.

### Reaction of [(^Ph^isq)(^Ph^ibq)V^IV^(O)Cl] with *cis*-Cyclooctene

[(^Ph^isq)(^Ph^ibq)V^IV^(O)Cl] (6.9 mg, 0.01 mmol) and *cis*-cyclooctene (9.0 mg, 0.08 mmol) were dissolved in CDCl_3_ (600 μL) and sealed under N_2_ in a J. Young style NMR tube. The tube was heated in an oil bath at 50 °C for 20 h.

### Reaction of [(^Ph^isq)(^Ph^ibq)V^IV^(O)Cl] with Dimethyl Sulfoxide

[(^Ph^isq)(^Ph^ibq)V^IV^(O)Cl] (6.2 mg, 0.009 mmol) and dimethyl sulfoxide (0.70 mg, 0.009 mmol) were dissolved in C_6_D_6_ (600 μL), sealed under N_2_ in a J. Young style NMR tube, and heated in an oil bath at 50 °C for 20 h.

### Reaction of [(^Ph^isq)(^Ph^ibq)V^IV^(O)Cl] with Dimethyl Sulfide

[(^Ph^isq)(^Ph^ibq)V^IV^(O)Cl] (6.9 mg, 0.010 mmol) and dimethyl sulfide (0.73 μL, 0.010 mmol) were dissolved in C_6_D_6_ (600 μL), sealed under N_2_ in a J. Young style NMR tube, and heated in an oil bath at 50 °C for 20 h.

### Computational Studies

DFT calculations were performed using ORCA 4.2.1^116,117^ using the PBE0 functional,^118^ def2-TZVPP^119,120^ basis set, D3 with Becke-Johnson dampening,^121^ and RIJCOSX^122^ approximation (default grid) on the full model. FOD calculations^101^ were performed at the same level of theory at 10,000 K using the output coordinates from the geometry optimization as input. Implicit solvent effects were modeled using the conductor-like polarizable continuum model.^123^ Spin density and molecular orbital plots were generated using IQmol (http://www.iqmol.org/).

## Supporting Information Available

UV–vis–NIR absorption data for reactions of **I** and **II** with O atom donor and acceptor substrates; selected ^1^H, ^13^C, ^31^P, and ^51^V NMR spectra complexes **I** and **II** and reaction with O atom donor and acceptor substrates; complete X-ray structure reports and selected X-ray crystallographic data for **I**, **IIa**, **IIb**, and 6,8-di*tert*-butyl-3H-phenoxazine-3-phenylazanylidinium hexachloroantimonate; cyclic voltammograms of **I**, **II**; computational details, selected DFT orbitals and density plots, and DFT coordinates for **I**, **IIa**, **IIb** (PDF)
