# Supplementary material for: Redox-Active Ligands Permit Multielectron O2 Homolysis and O-Atom Transfer at Exceptionally High-Valent Vanadyl Complexes
Source: J Am Chem Soc. 2025 Apr 9;147(16):13356–69. doi: 10.1021/jacs.4c18305 (PMC12023041; doi:10.1021/jacs.4c18305)
Supplement: Supplementary file 1 — ja4c18305_si_001.pdf [file ja4c18305_si_001.pdf]

Supporting Information for:

Redox-Active Ligands Permit Multielectron O_2 Homolysis and O-Atom Transfer at Exceptionally High-Valent Vanadyl Complexes

Andrew G. Hill,[a] Mariah C. Castillo,[a]
John Bacsa,[a,b] Kaitlyn S. Otte,[a] and Jake D. Soper*,[a]

[a]School of Chemistry and Biochemistry, Georgia Institute of Technology, Atlanta, Georgia 30332-0400, United States

[b]X-ray Crystallography Center, Department of Chemistry, Emory University, 1515 Dickey Drive, Atlanta, Georgia 30322, United States

Email: jake.soper@gatech.edu

Table of Contents

UV-vis Data **S5**

Figure S1 Absorption spectra (benzene) for [(^{Ph}ap)(^{Ph}isq)VCl] (**I**) and [(^{Ph}isq)(^{Ph}ibq)V(O)Cl] (**II**). **S5**

Figure S2 Absorption spectrum (benzene) for the reaction of [(^{Ph}isq)(^{Ph}ibq)V(O)Cl] (**II**) and *tris*-(4-bromophenyl)aminium hexachloroantimonate. **S6**

Figure S3 Absorption spectra (benzene) of 1:1 mixture of complex **II** and triphenylphosphine after 24h at room temperature and a 1:1 mixture of complex **I** and triphenylphosphine oxide after 24h at room temperature. **S7**

Figure S4 Absorption spectrum (benzene) of 1:1 mixture of complex **II** and triphenylarsine in benzene after 24h at room temperature. **S8**

Figure S5 Absorption spectrum (benzene) of 1:1 mixture of complex **II** and dimethylsulfoxide in benzene after 24h at 50 °C. **S9**

Figure S6 Absorption spectrum (benzene) of 1:20 mixture of complex **II** and dimethylsulfide in benzene after 24h at 50 °C. **S10**

Figure S7 Absorption spectra (benzene) for the reaction of [(^{Ph}isq)(^{Ph}ibq)V(O)Cl] (**II**) and 9,10-dihydroanthracene compared to [(^{Ph}ap)(^{Ph}isq)VCl] (**I**) and anthracene. **S11**

NMR Data **S12**

Figure S8 ^{1}H NMR spectrum (500 MHz, C_6D_6) of [(^{Ph}ap)(^{Ph}isq)VCl] (**I**). **S12**

**Figure S9**	Evans' Method ^{1}H NMR spectrum (500 MHz, C_6D_6) of 5.0 mM [(^{Ph}ap)(^{Ph}isq)VCl] (**I**).	**S13**
**Figure S10**	Evans' Method ^{1}H NMR spectrum (500 MHz, THF-d_8) of 18 mM [(^{Ph}ap)(^{Ph}isq)VCl] (**I**).	**S14**
**Figure S11**	Evans' Method ^{1}H NMR spectrum (500 MHz, $CDCl_3$) of 19 mM [(^{Ph}ap)(^{Ph}isq)VCl] (**I**).	**S15**
**Figure S12**	Evans' Method ^{1}H NMR spectrum (500 MHz, $CDCl_3$) of 11 mM [(^{Ph}ap)(^{Ph}isq)VCl] (**I**).	**S16**
**Figure S13**	Evans' Method ^{1}H NMR spectrum (500 MHz, C_6D_6) of 9.9 mM [(^{Ph}ap)(^{Ph}isq)VCl] (**I**).	**S17**
**Figure S14**	Evans' Method ^{1}H NMR spectra (400 MHz, $CDCl_3$) of 26 mM [(^{Ph}ap)(^{Ph}isq)VCl] (**I**) from -50°C to 50°C.	**S18**
**Figure S15**	^{1}H NMR spectrum (500 MHz, C_6D_6) of [(^{Ph}isq)(^{Ph}ibq)V(O)Cl] (**II**).	**S19**
**Figure S16**	^{51}V NMR spectrum (400 MHz, C_6D_6) of [(^{Ph}isq)(^{Ph}ibq)V(O)Cl] (**II**).	**S20**
**Figure S17**	Evans' Method ^{1}H NMR spectrum (500 MHz, C_6D_6) of 5.4 mM [(^{Ph}isq)(^{Ph}ibq)V(O)Cl] (**II**).	**S21**
**Figure S18**	Evans' Method ^{1}H NMR spectrum (400 MHz, toluene-d_8) of 26 mM [(^{Ph}isq)(^{Ph}ibq)V(O)Cl] (**II**).	**S22**
**Figure S19**	Evans' Method ^{1}H NMR spectrum (500 MHz, $CDCl_3$) of 20 mM [(^{Ph}isq)(^{Ph}ibq)V(O)Cl] (**II**).	**S23**
**Figure S20**	Evans' Method ^{1}H NMR spectrum (500 MHz, $CDCl_3$) of 10 mM [(^{Ph}isq)(^{Ph}ibq)V(O)Cl] (**II**).	**S24**
**Figure S21**	Evans' Method ^{1}H NMR spectrum (500 MHz, $CDCl_3$) of 11 mM [(^{Ph}isq)(^{Ph}ibq)V(O)Cl] (**II**).	**S25**
**Figure S22**	Evans' Method ^{1}H NMR spectra (400 MHz, $CDCl_3$) of 11 mM [(^{Ph}isq)(^{Ph}ibq)V(O)Cl] (**II**) from -50°C to 50°C.	**S26**
**Figure S23**	^{31}P NMR spectrum (500 MHz, C_6D_6) of the products of the reaction of complex **II** and triphenylphosphine.	**S27**
**Figure S24**	^{31}P NMR spectrum (500 MHz, C_6D_6) of the products of the reaction of complex **II** and triphenylphosphine after being open to air for 24 hours.	**S28**
**Figure S25**	^{31}P NMR spectrum (500 MHz, C_6D_6) of the products of the reaction of complex **I** and triphenylphosphine oxide.	**S29**
**Figure S26**	^{1}H NMR spectrum (500 MHz, C_6D_6) of the products of the reaction between **II** and one equivalent DMSO.	**S30**
**Figure S27**	^{1}H NMR spectra (500 MHz, C_6D_6) of: the products of the reaction between **II** and one equivalent DMSO, the products of the reaction between **II** and one equivalent DMS, authentic DMS,	**S31**

authentic DMSO, and authentic $DMSO_2$.

Figure S28 ^1H NMR spectra (500 MHz, C_6D_6) of: the products of the reaction between **II** and one equivalent DMSO, authentic complex **I**, and authentic complex **II**. **S32**

Figure S29 ^{13}C NMR spectrum (500 MHz, $CDCl_3$) of the reaction products of **II** and 8 equivalents cis-cyclooctene. **S33**

Figure S30 ^1H NMR spectrum (500 MHz, $CDCl_3$) of the reaction products of **II** and 8 equivalents cis-cyclooctene. **S34**

Figure S31 ^1H NMR spectra (500 MHz, C_6D_6) of 1:1 ratio of complex **II** and 9,10-dihydroanthracene after 20 hours at room temperature and 20 hours at 50°C. **S35**

Figure S32 ^1H NMR spectrum (300 MHz, C_6D_6) of 1:10 ratio of complex **II** and 9,10-dihydroanthracene after 20 hours open to air at 50°C. **S36**

X-Ray Structure Reports **S37**

[(^{Ph}ap)(^{Ph}isq)VCl] (I) **S37**

***Trans*-[(^{Ph}isq)(^{Ph}ibq)V(O)Cl] (IIa)** **S46**

***Cis*-[(^{Ph}isq)(^{Ph}ibq)V(O)Cl] (IIb)** **S56**

6,8-di-tert-butyl-3H-phenoxazine-3-phenylazanylidinium hexachloroantimonate **S74**

Cyclic Voltammetry Data **S83**

Figure S33 Cyclic voltammogram of complex **I** in acetonitrile. **S83**

Figure S34 Cyclic voltammogram of complex **II** in acetonitrile. **S84**

Computational Data **S85**

Table S26 Computed spin on vanadium in crystal and implicit solvent environments and MOS values from the crystal structures and DFT optimized coordinates. **S85**

DFT Orbitals and Density Plots **S86**

Figure S35 Calculated HOMO-1α of S = 0 [(^{Ph}ap)(^{Ph}isq)V^{IV}Cl] (**I**). **S86**

Figure S36 Calculated HOMO-1β of S = 0 [(^{Ph}ap)(^{Ph}isq)V^{IV}Cl] (**I**). **S86**

Figure S37 Calculated HOMOα of S = 0 [(^{Ph}ap)(^{Ph}isq)V^{IV}Cl] (**I**). **S87**

Figure S38 Calculated HOMOβ of S = 0 [(^{Ph}ap)(^{Ph}isq)V^{IV}Cl] (**I**). **S87**

Figure S39 Spin density plot of S = 1 [(^{Ph}ap)(^{Ph}isq)V^{IV}Cl] (I) and Löwdin spin population per atom. **S88**

Figure S40 Calculated HOMO-2α of S = 1 [(^{Ph}ap)(^{Ph}isq)V^{IV}Cl] (**I**). **S89**

Figure S41 Calculated HOMO-2β of S = 1 [(^{Ph}ap)(^{Ph}isq)V^{IV}Cl] (**I**). **S89**

**Figure S42**	Calculated HOMO-1α of S = 1 [(^{Ph}ap)(^{Ph}isq)V^{IV}Cl] (**I**).	**S90**
**Figure S43**	Calculated HOMOα of S = 1 [(^{Ph}ap)(^{Ph}isq)V^{IV}Cl] (**I**).	**S90**
**Figure S44**	Calculated LUMO of S = 1 *cis*-[(^{Ph}isq)(^{Ph}ibq)V(O)Cl] (**IIb**).	**S91**
**Figure S45**	Calculated HOMO of S = 0 *trans*-[^{Ph}isq)(^{Ph}ibq)V(O)Cl] (**IIa**).	**S91**
**Figure S46**	Calculated LUMO of S = 0 *trans*-[^{Ph}isq)(^{Ph}ibq)V(O)Cl] (**IIa**).	**S92**
**Figure S47**	Calculated LUMO+1 of S = 0 *trans*-[^{Ph}isq)(^{Ph}ibq)V(O)Cl] (**IIa**).	**S92**
**Figure S48**	Calculated LUMO+2 of S = 0 *trans*-[^{Ph}isq)(^{Ph}ibq)V(O)Cl] (**IIa**).	**S93**
**Figure S49**	Calculated LUMO+3 of S = 0 *trans*-[^{Ph}isq)(^{Ph}ibq)V(O)Cl] (**IIa**).	**S93**
**Figure S50**	Calculated HOMO of S = 0 *cis*-[(^{Ph}isq)(^{Ph}ibq)V(O)Cl] (**IIb**).	**S94**
**Figure S51**	Calculated LUMO of S = 0 *cis*-[(^{Ph}isq)(^{Ph}ibq)V(O)Cl] (**IIb**).	**S94**
**Figure S52**	Calculated LUMO+1 of S = 0 *cis*-[(^{Ph}isq)(^{Ph}ibq)V(O)Cl] (**IIb**).	**S95**
**Figure S53**	Calculated LUMO+2 of S = 0 *cis*-[(^{Ph}isq)(^{Ph}ibq)V(O)Cl] (**IIb**).	**S95**
**Figure S54**	Calculated LUMO+3 of S = 0 cis-[(^{Ph}isq)(^{Ph}ibq)V(O)Cl] (**IIb**).	**S96**
**Figure S55**	FOD of S = 0 *trans*-[^{Ph}isq)(^{Ph}ibq)V(O)Cl] (**IIa**).	**S96**
**Figure S56**	FOD of S = 1 *cis*-[(^{Ph}isq)(^{Ph}ibq)V(O)Cl] (**IIb**).	**S97**
**DFT Coordinates**		**S97**
**List S1**	[(^{Ph}ap)(^{Ph}isq)VCl] (**I**), PBE0, S = 0	**S97**
**List S2**	[(^{Ph}ap)(^{Ph}isq)VCl] (**I**), PBE0, S = 1	**S99**
**List S3**	[(^{Ph}ap)(^{Ph}isq)VCl] (**I**), PBE0, S = 2	**S102**
**List S4**	[(^{Ph}ap)(^{Ph}isq)VCl] (**I**), PBE0, CPCM (benzene), S = 0	**S104**
**List S5**	[(^{Ph}ap)(^{Ph}isq)VCl] (**I**), PBE0, CPCM (benzene), S = 1	**S106**
**List S6**	[(^{Ph}ap)(^{Ph}isq)VCl] (**I**), PBE0, CPCM (benzene), S = 2	**S108**
**List S7**	*Trans*-[(^{Ph}isq)(^{Ph}ibq)V(O)Cl] (**IIa**), S = 0	**S111**
**List S8**	*Trans*-[(^{Ph}isq)(^{Ph}ibq)V(O)Cl] (**IIa**), S = 1	**S113**
**List S9**	*Trans*-[(^{Ph}isq)(^{Ph}ibq)V(O)Cl] (**IIa**), CPCM (benzene), S = 0	**S115**
**List S10**	*Trans*-[(^{Ph}isq)(^{Ph}ibq)V(O)Cl] (**IIa**), CPCM (benzene), S = 1	**S117**
**List S11**	*Cis*-[(^{Ph}isq)(^{Ph}ibq)V(O)Cl] (**IIb**), S = 0	**S119**
**List S12**	*Cis*-[(^{Ph}isq)(^{Ph}ibq)V(O)Cl] (**IIb**), S = 1	**S121**
**List S13**	*Cis*-[(^{Ph}isq)$_2$V(O)Cl] (**IIb'**), S = 0	**S123**
**List S14**	*Cis*-[(^{Ph}isq)$_2$V(O)Cl] (**IIb'**), S = 1	**S125**
**References**		**S128**

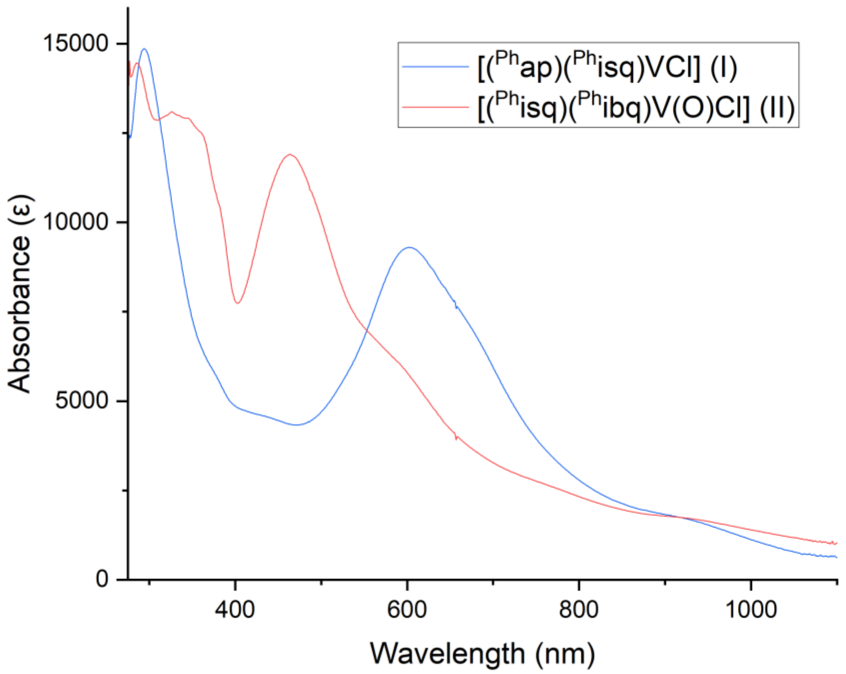

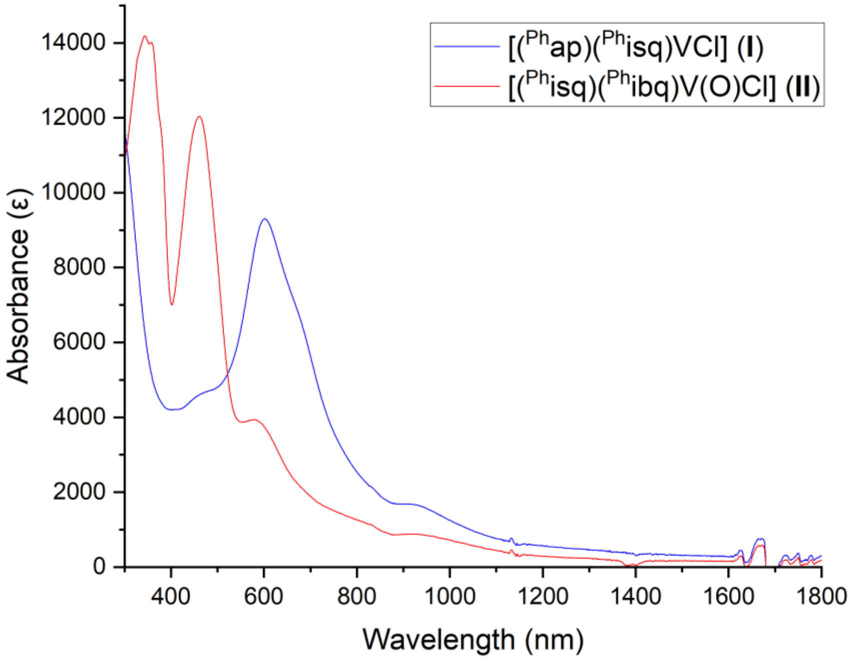

Figure S1. UV–vis and UV–vis–NIR absorption spectra (benzene) of complexes [(^{Ph}ap)(^{Ph}isq)VCl] (**I**) and [(^{Ph}isq)(^{Ph}ibq)V(O)Cl] (**II**). Some samples of **I** and **II** exhibit an absorption feature of varying intensity at 920 nm, which we ascribed to trace quantities of [V(^{Ph}ap)$_2$(^{Ph}isq)].[1]

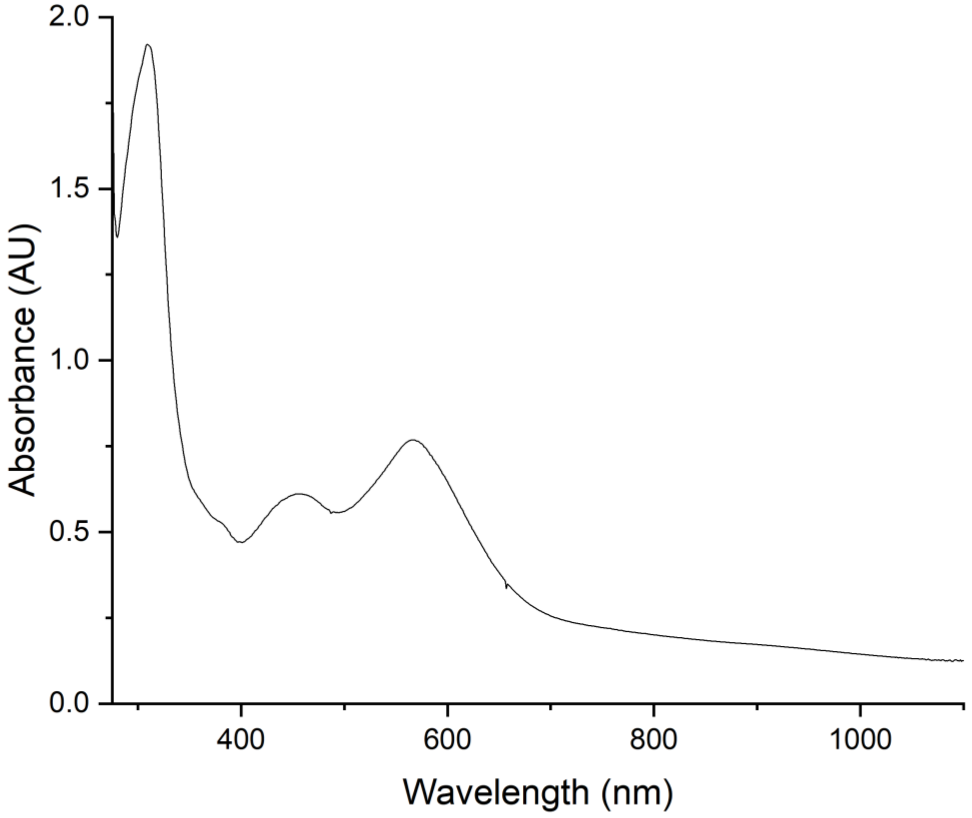

Figure S2: UV–vis spectrum (benzene) of the products of the reaction between complex **II** and tris-(4-bromophenyl)aminium hexachloroantimonate.

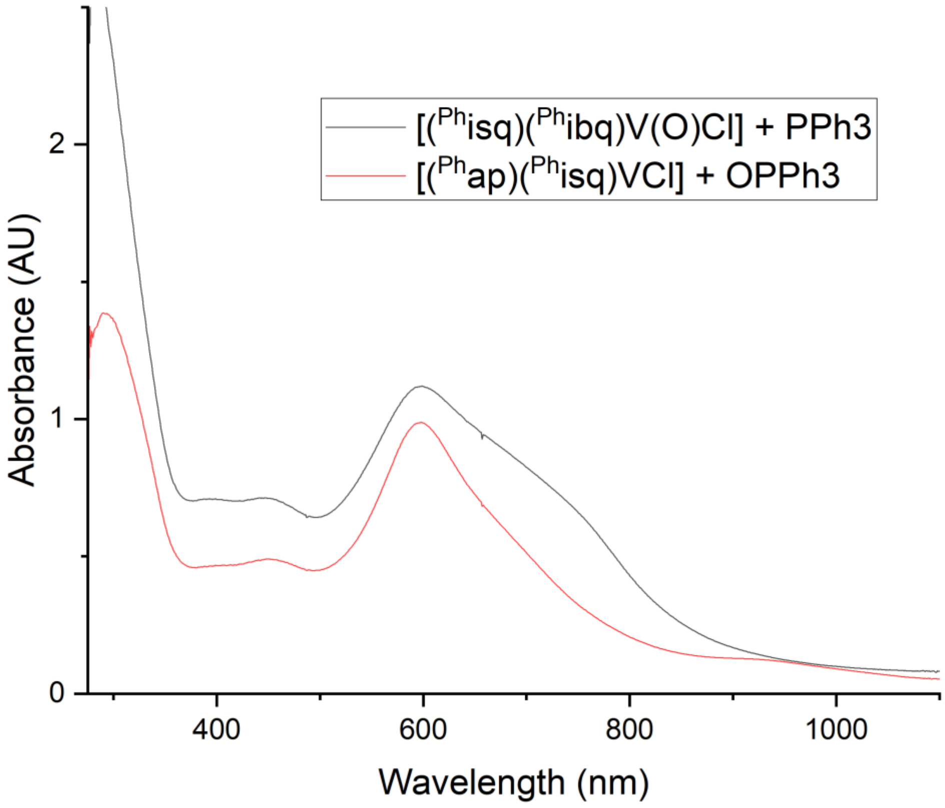

Figure S3: UV–vis spectra (benzene) of 1:1 mixture of complex **II** and triphenylphosphine after 24h at room temperature (black) and a 1:1 mixture of complex **I** and triphenylphosphine oxide after 24h at room temperature (red).

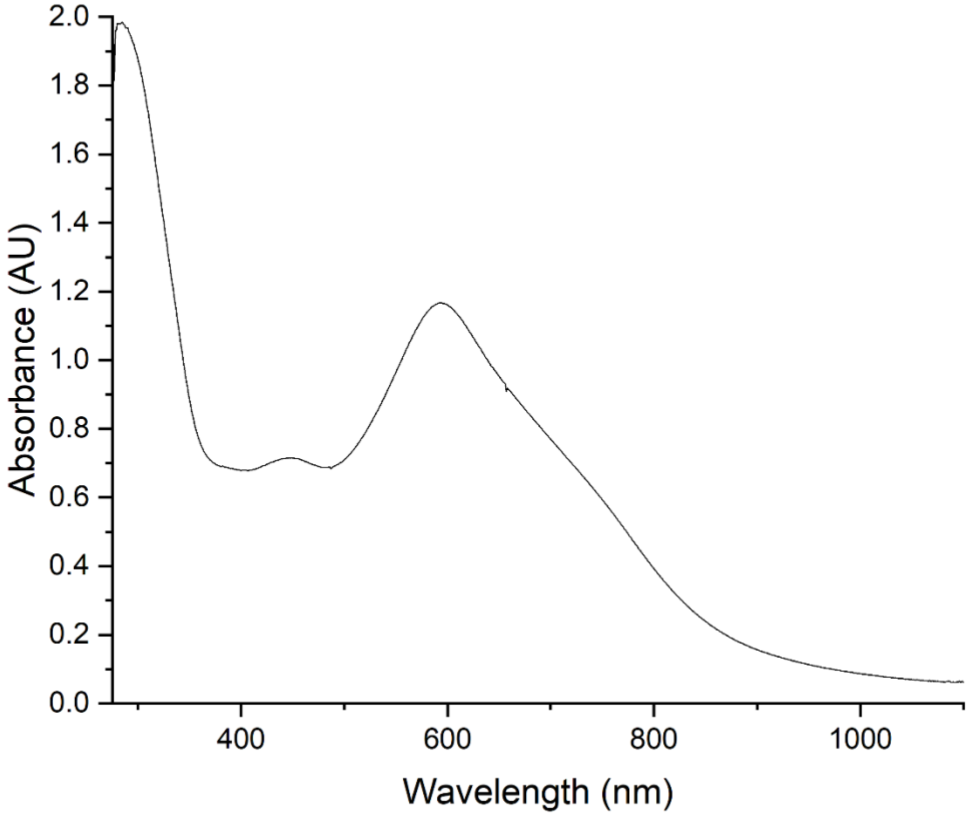

Figure S4: UV–vis spectrum (benzene) of 1:1 mixture of complex **II** and triphenylarsine in benzene after 24h at room temperature.

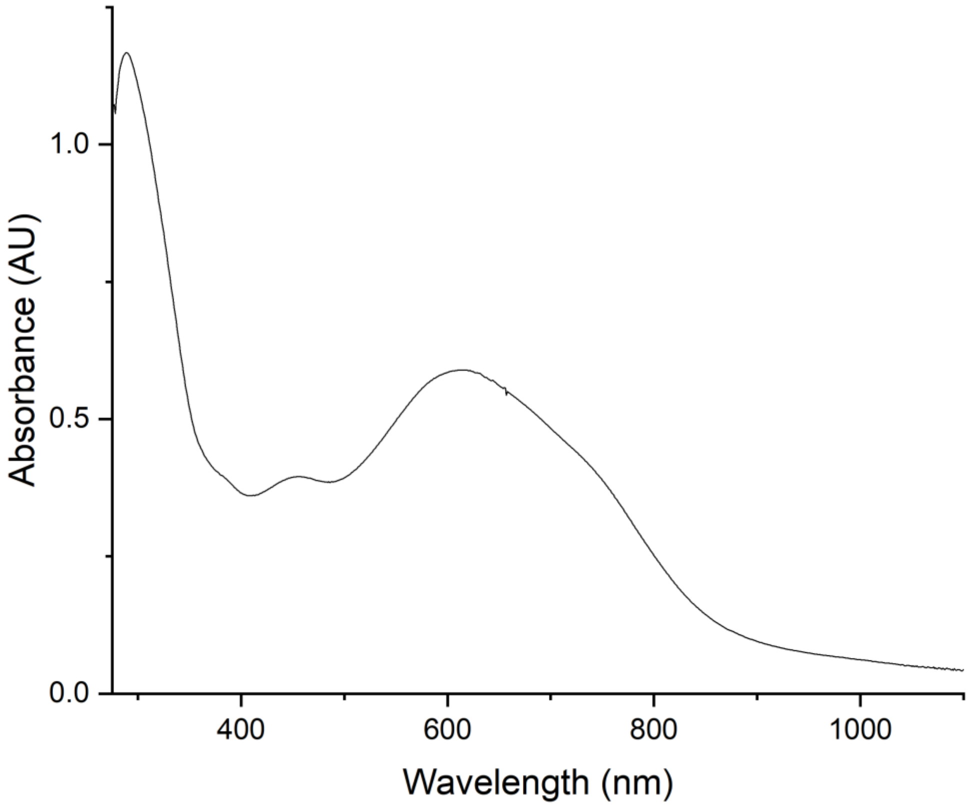

Figure S5: UV–vis spectrum (benzene) of 1:1 mixture of complex **II** and dimethylsulfoxide in benzene after 24h at 50 °C.

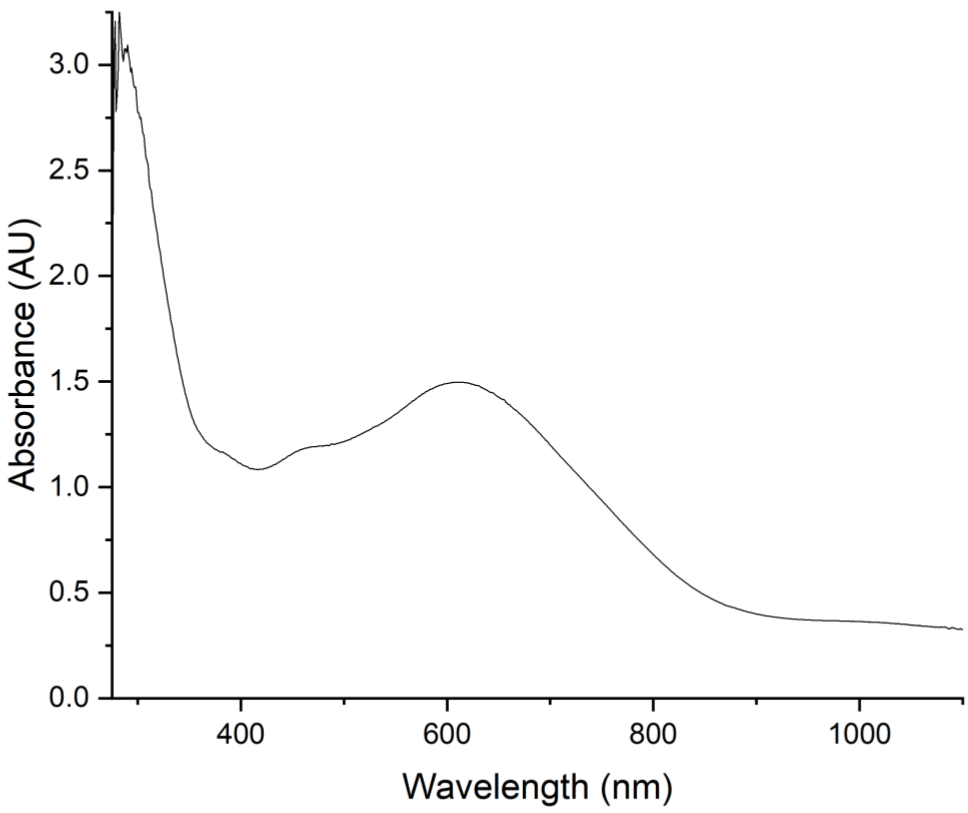

Figure S6: UV–vis spectrum (benzene) of 1:20 mixture of complex **II** and dimethylsulfide in benzene after 24h at 50 °C.

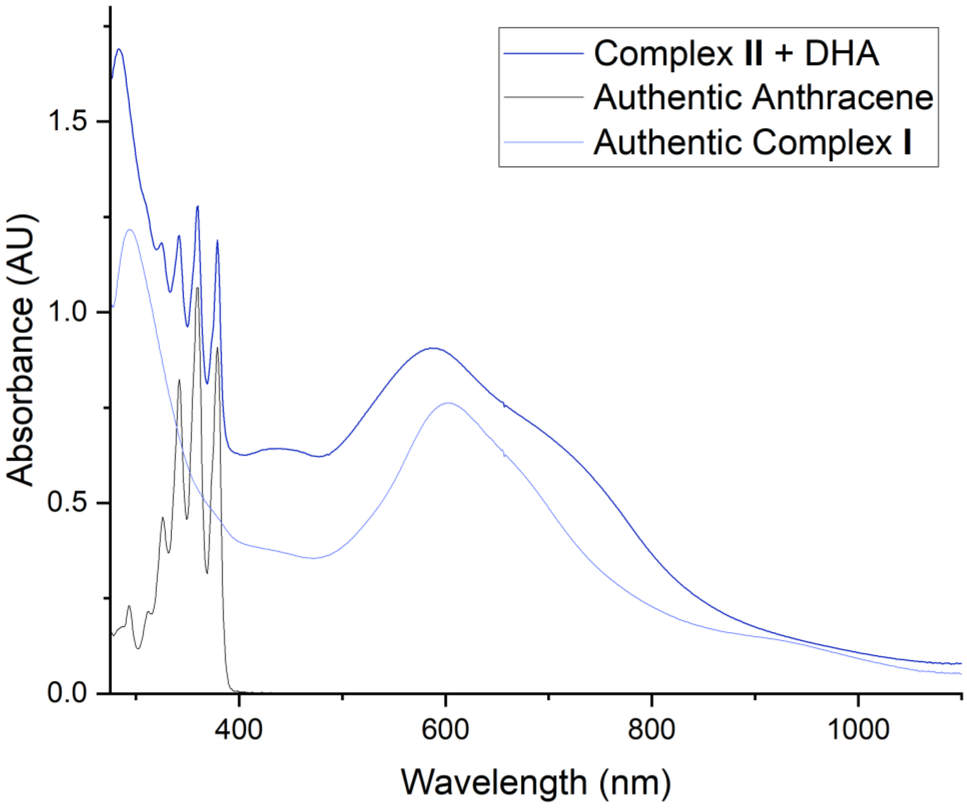

Figure S7. UV–vis absorption spectra (benzene) of the products of the reaction of complex **II** with 9,10-dihydroanthracene compared to authentic samples of complex **I** and anthracene.

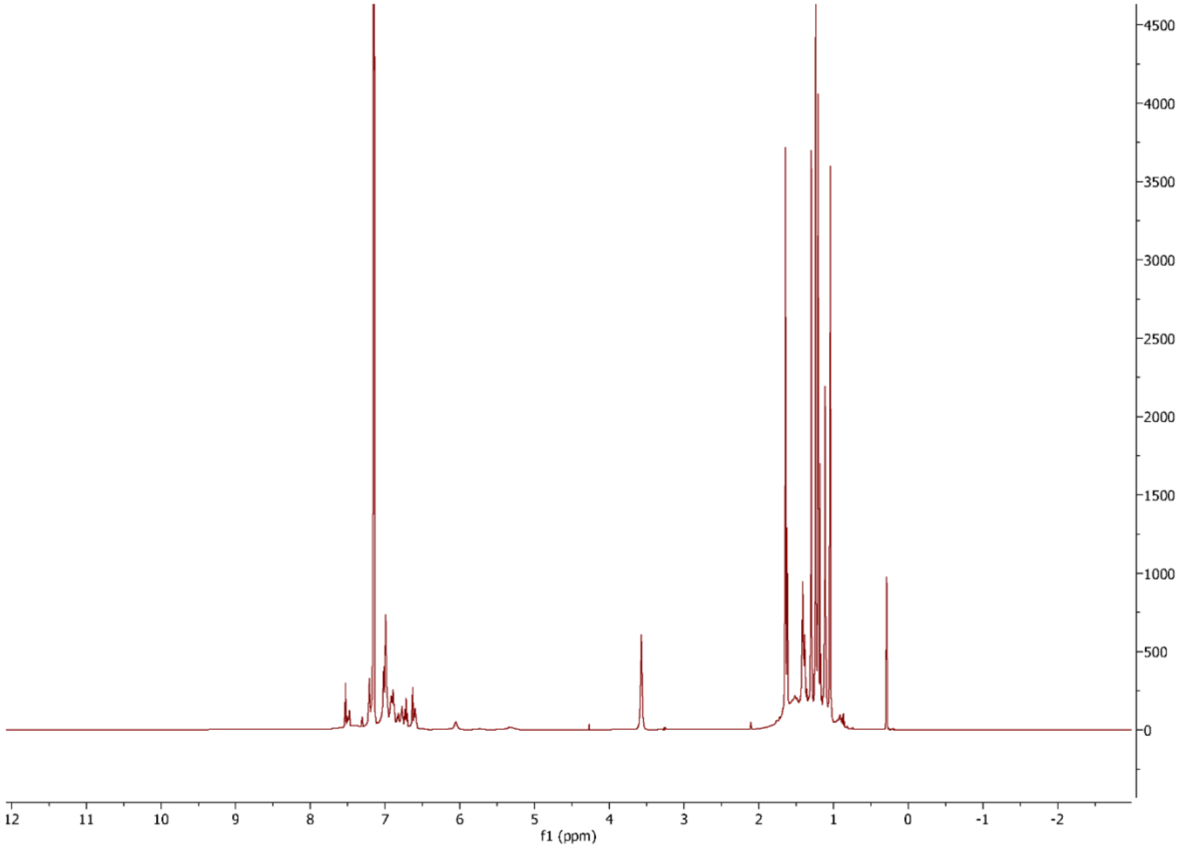

Figure S8: ^{1}H NMR spectrum (500 MHz, C_6D_6) of [(^{Ph}ap)(^{Ph}isq)VCl] (**I**).

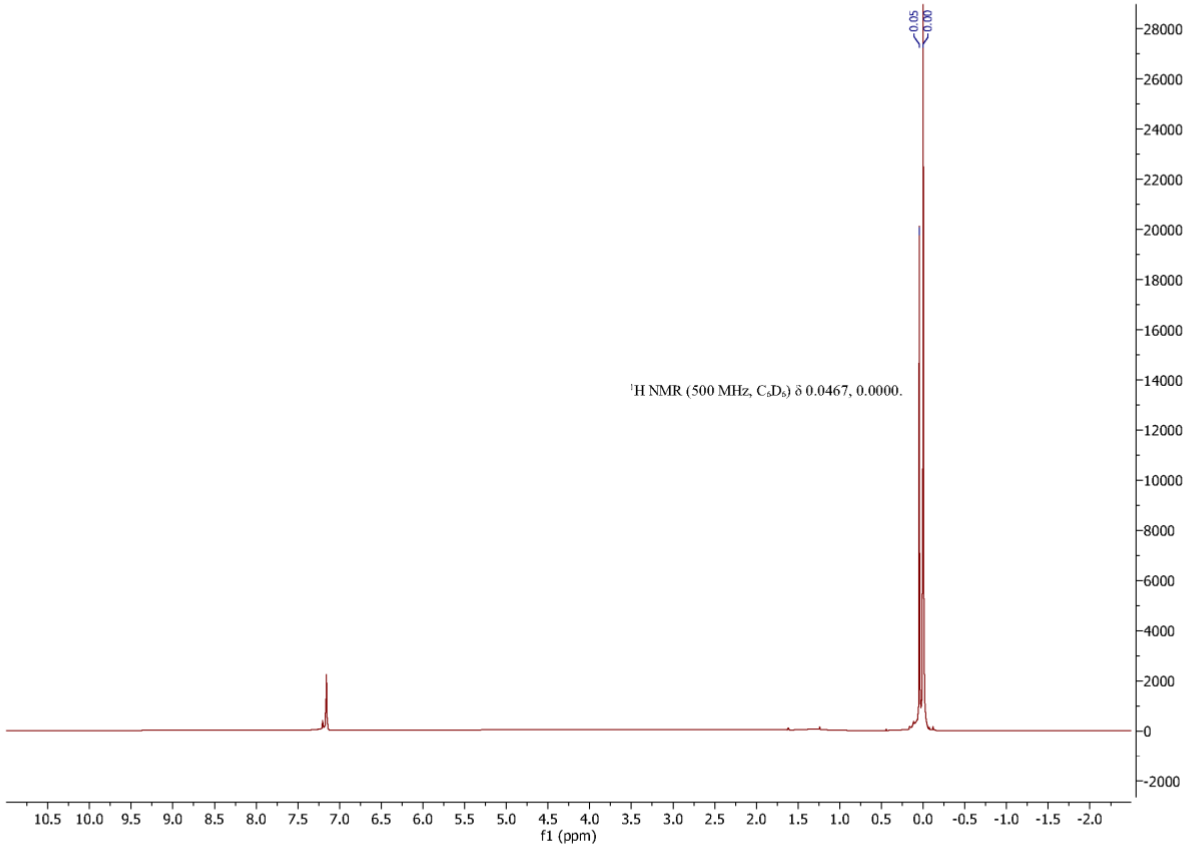

Figure S9: Evans' Method ^{1}H NMR spectrum (500 MHz, C_6D_6) of 4.9 mM [(^{Ph}ap)(^{Ph}isq)VCl] (**I**).

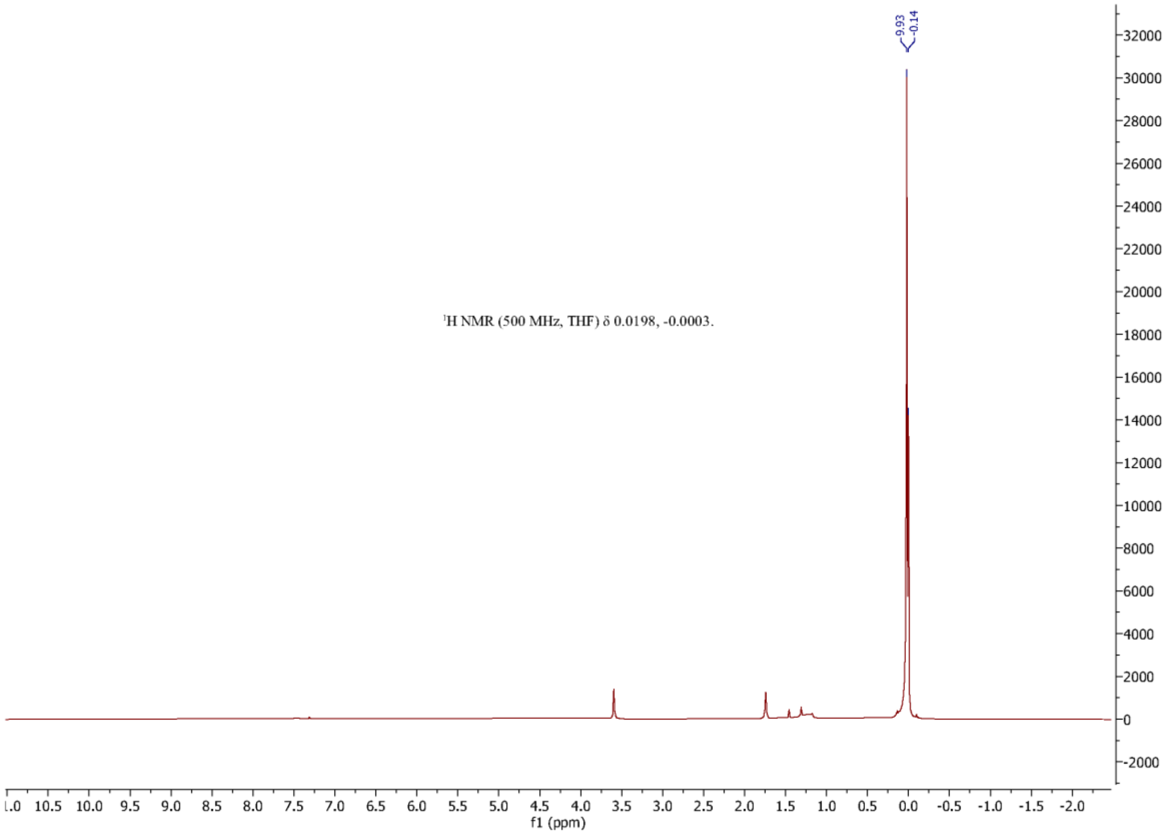

Figure S10: Evans' Method ^{1}H NMR spectrum (500 MHz, THF-d_8) of 18 mM [(^{Ph}ap)(^{Ph}isq)VCl] (**I**).

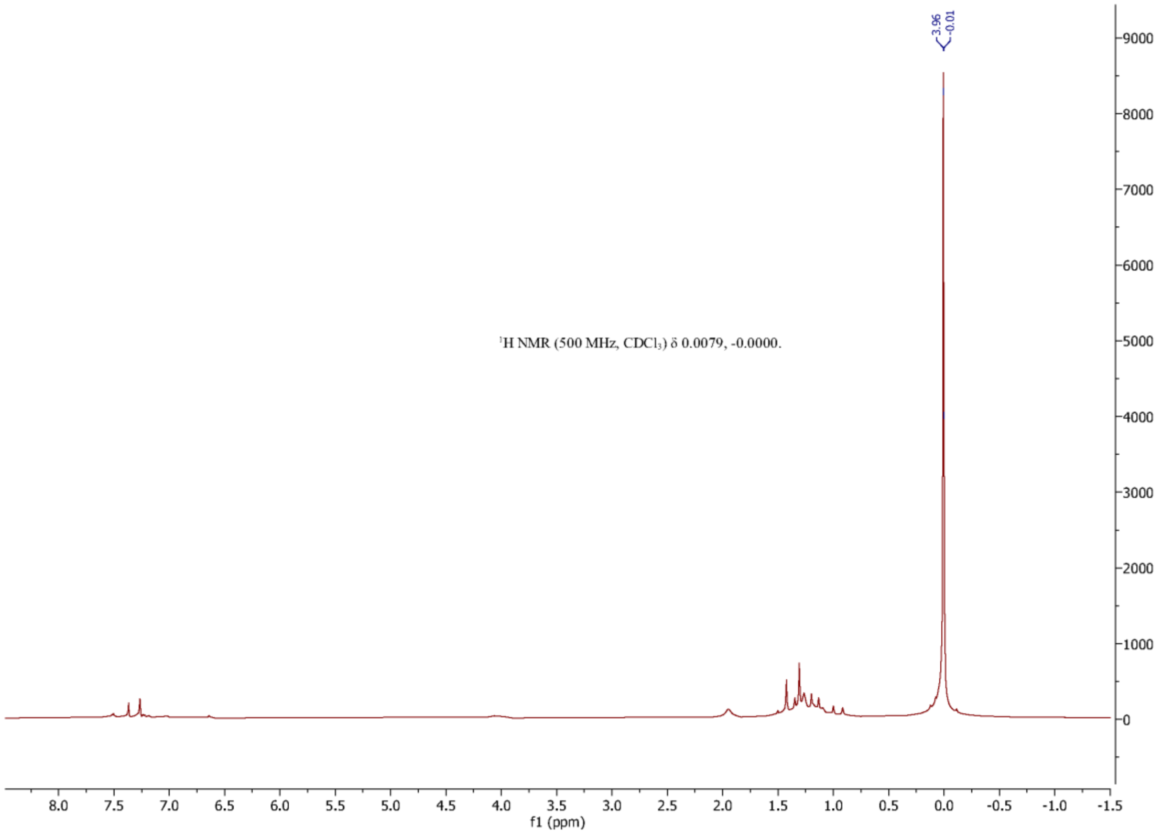

Figure S11: Evans' Method ^{1}H NMR spectrum (500 MHz, $CDCl_3$) of 19 mM [(^{Ph}ap)(^{Ph}isq)VCl] (**I**).

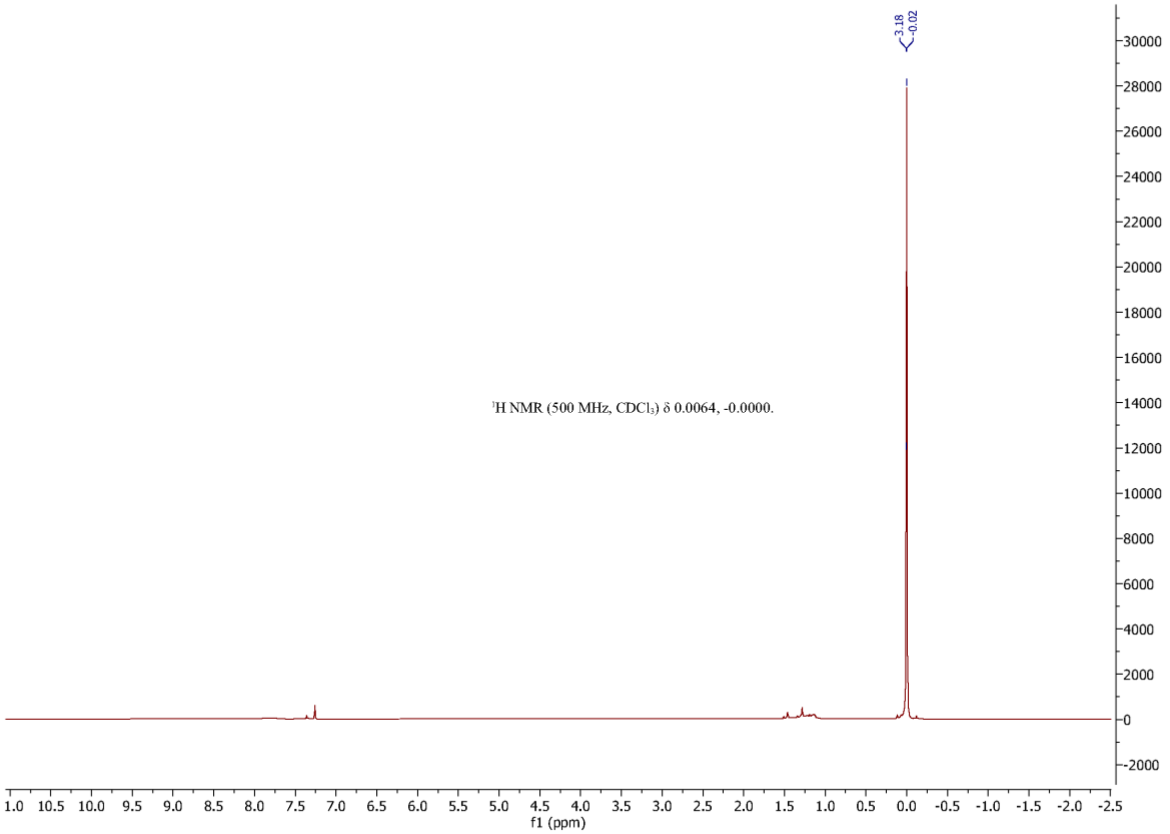

Figure S12: Evans' Method ^{1}H NMR spectrum (500 MHz, $CDCl_3$) of 11 mM [(^{Ph}ap)(^{Ph}isq)VCl] (**I**).

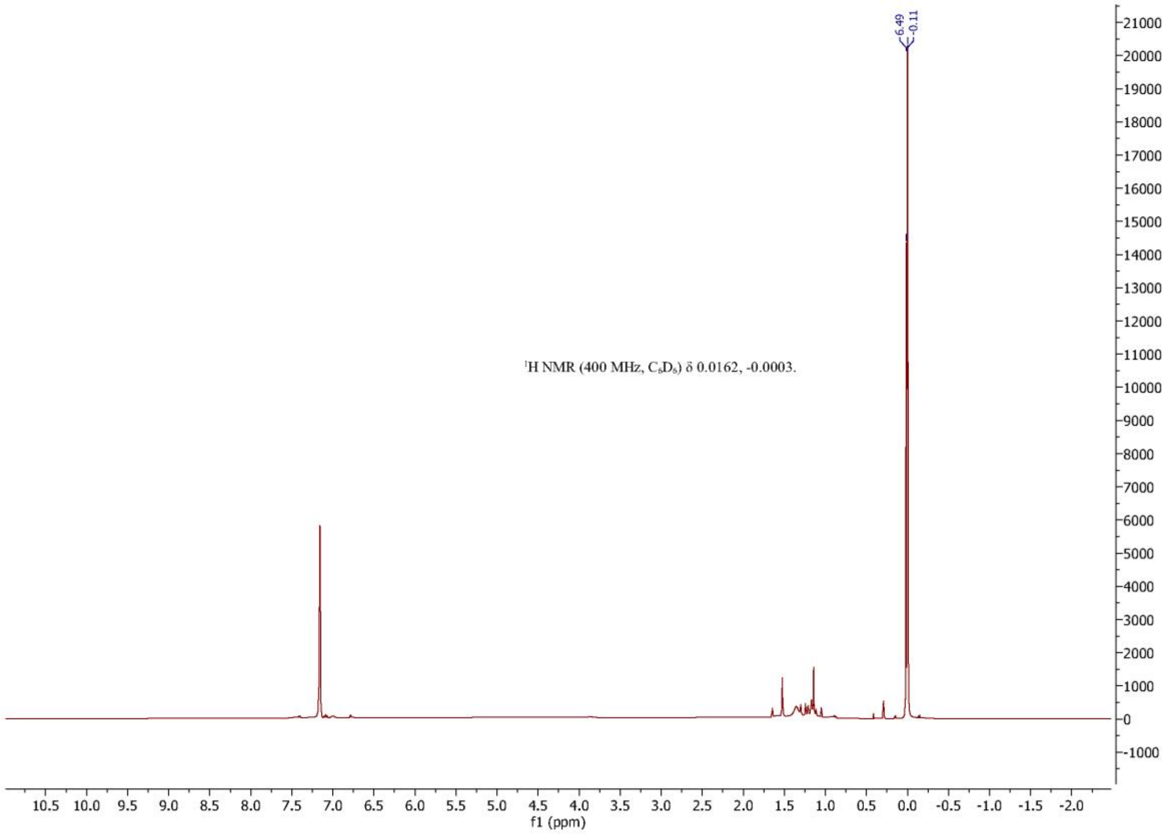

Figure S13: Evans' Method ^{1}H NMR spectrum (500 MHz, C_6D_6) of 9.9 mM [(^{Ph}ap)(^{Ph}isq)VCl] (**I**).

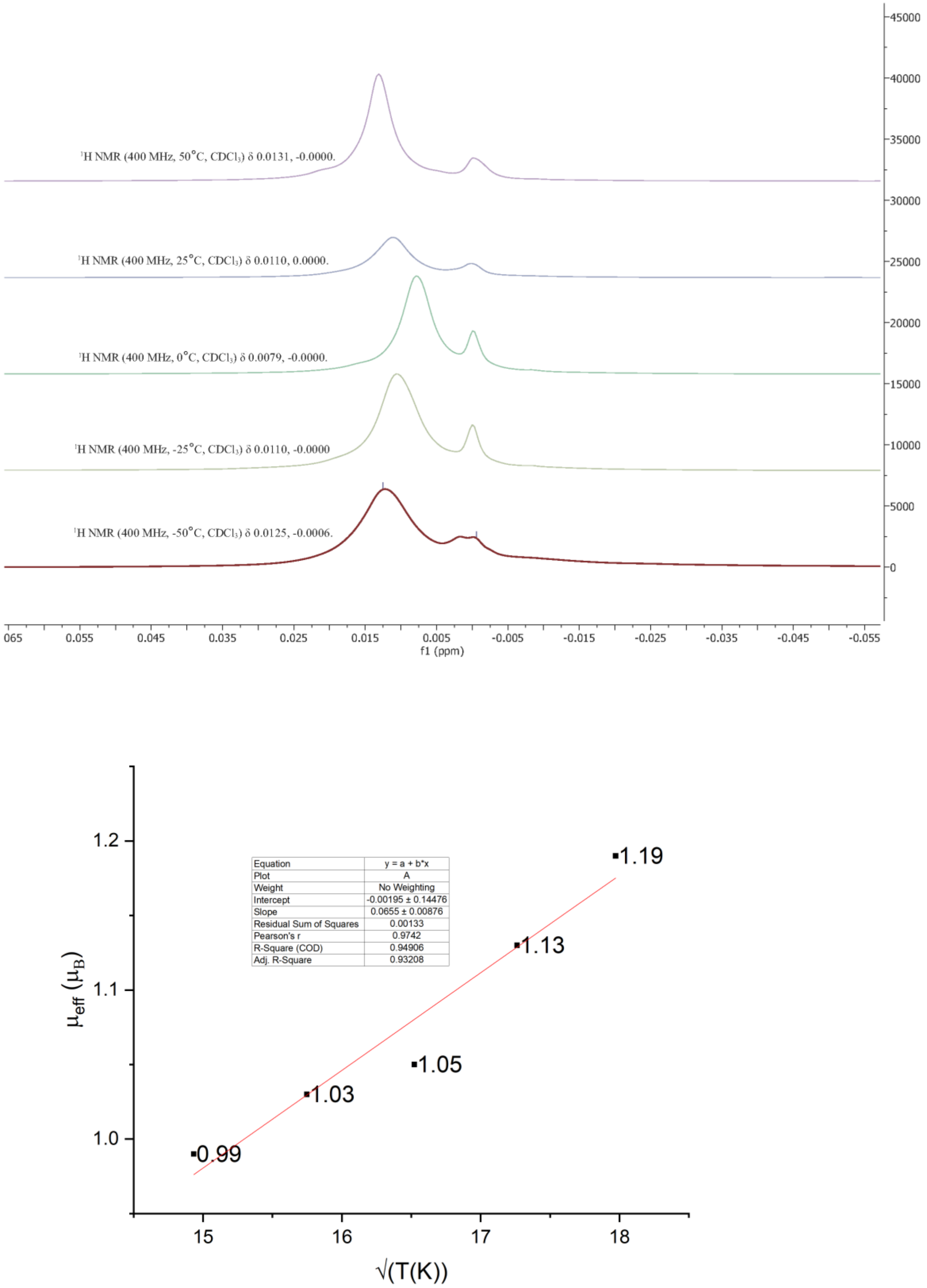

Figure S14: Evans' Method ^{1}H NMR spectra (400 MHz, $CDCl_3$) of 26 mM [(^{Ph}ap)(^{Ph}isq)VCl] (**I**) from -50°C to 50°C (above) and linear fit of calculated μ_{eff} vs. $\sqrt{T}$ (below).

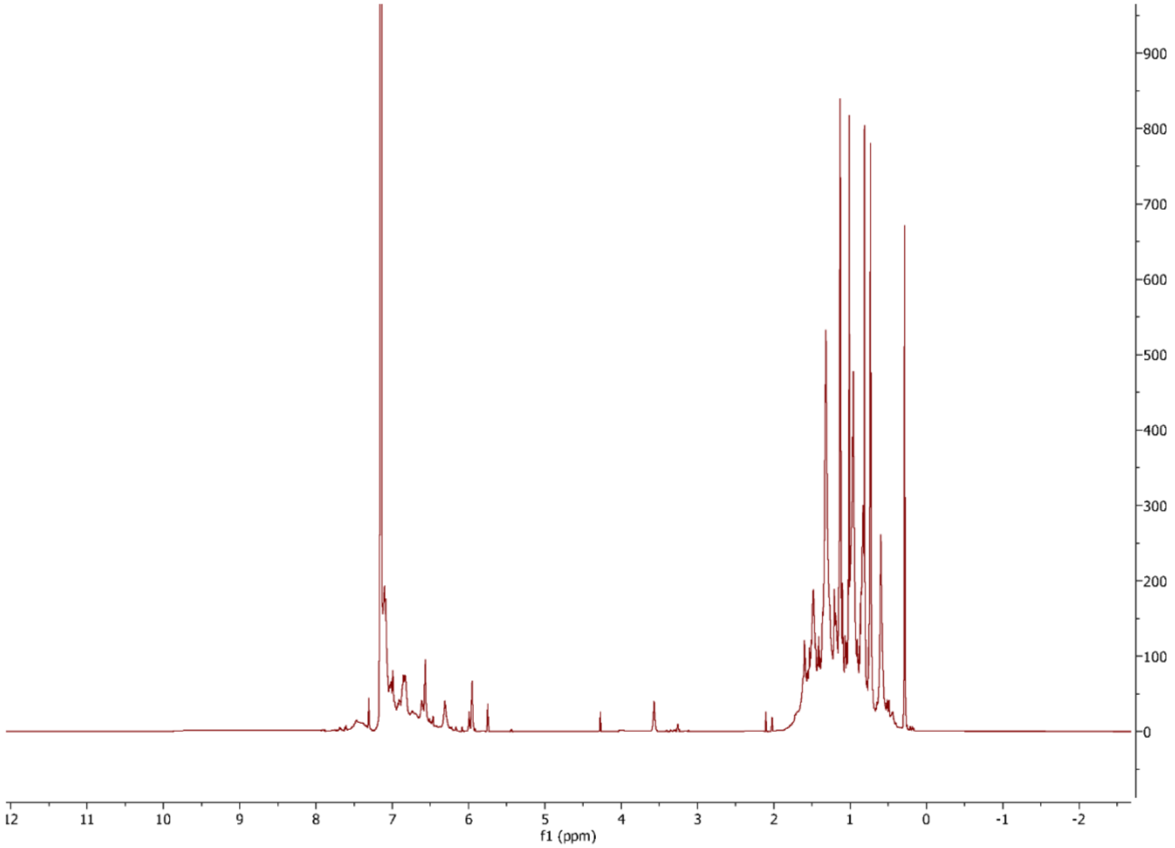

Figure S15: ^{1}H NMR spectrum (500 MHz, C_6D_6) of [(^{Ph}isq)(^{Ph}ibq)V(O)Cl] (**II**).

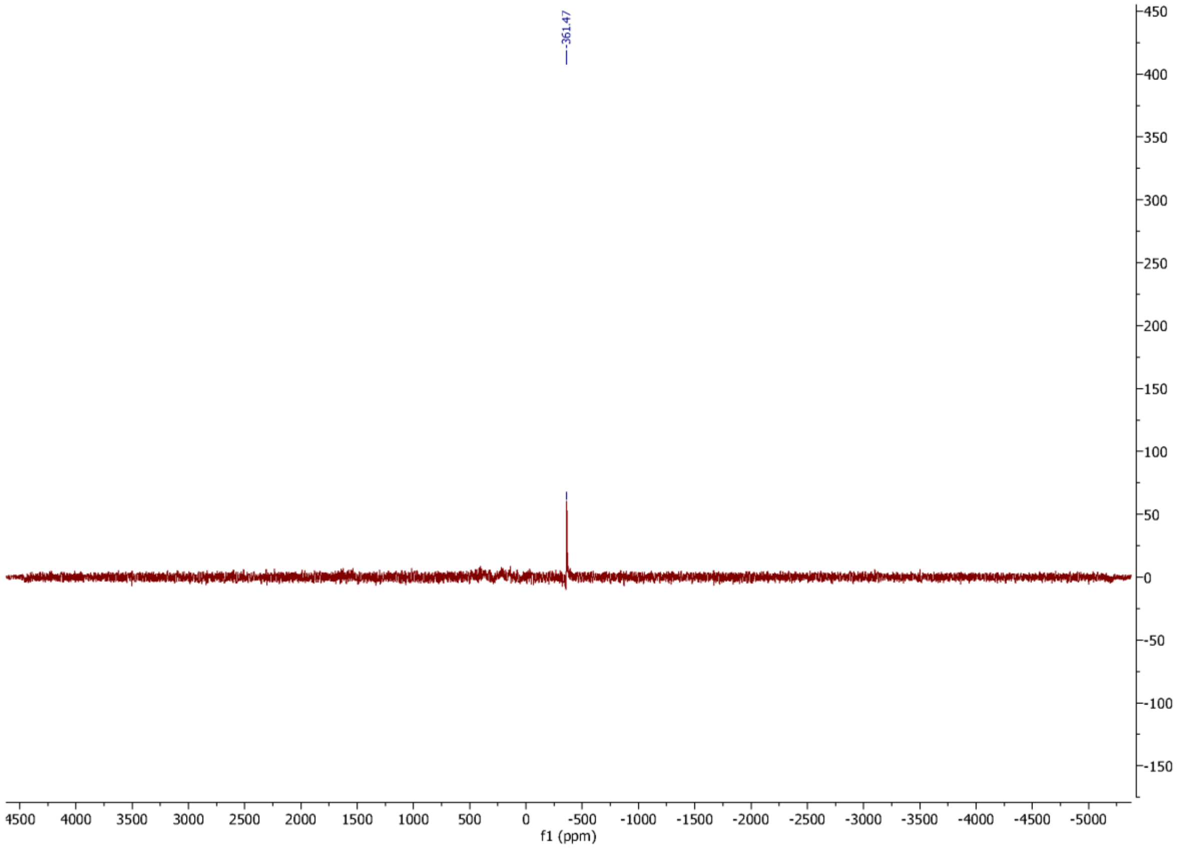

Figure S16: ^{51}V NMR spectrum (400 MHz, C_6D_6) of [(^{Ph}isq)(^{Ph}ibq)V(O)Cl] (**II**).

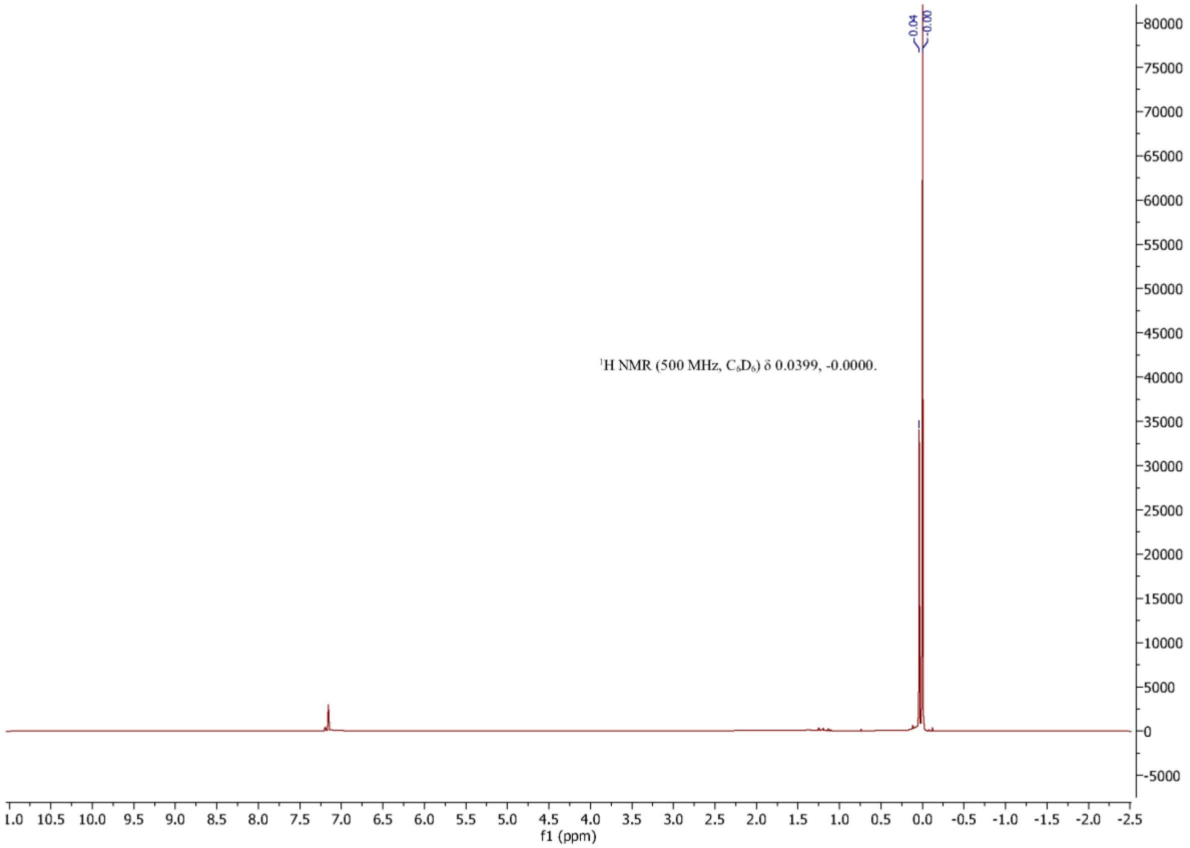

Figure S17: Evans' Method ^{1}H NMR spectrum (500 MHz, C_6D_6) of 5.4 mM [(^{Ph}isq)(^{Ph}ibq)V(O)Cl] (**II**).

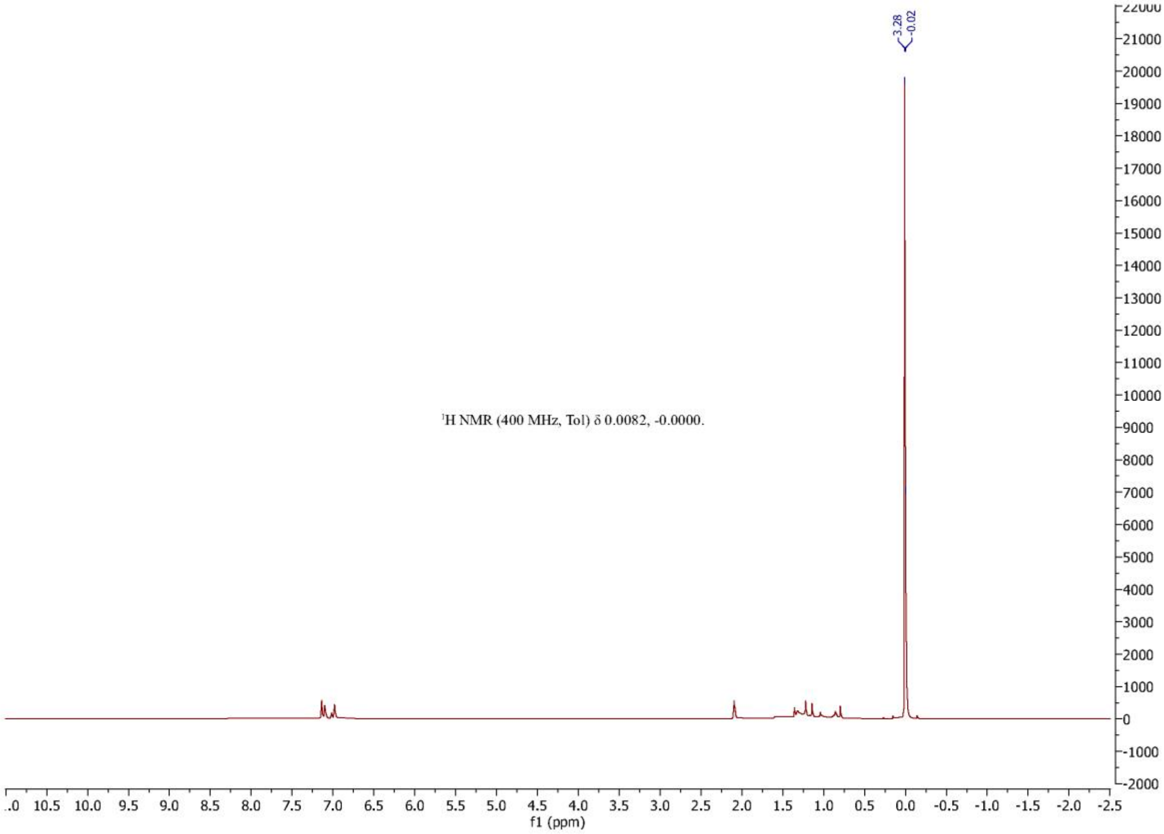

Figure S18: Evans' Method ^{1}H NMR spectrum (400 MHz, toluene-d_8) of 26 mM [(^{Ph}isq)(^{Ph}ibq)V(O)Cl] (**II**).

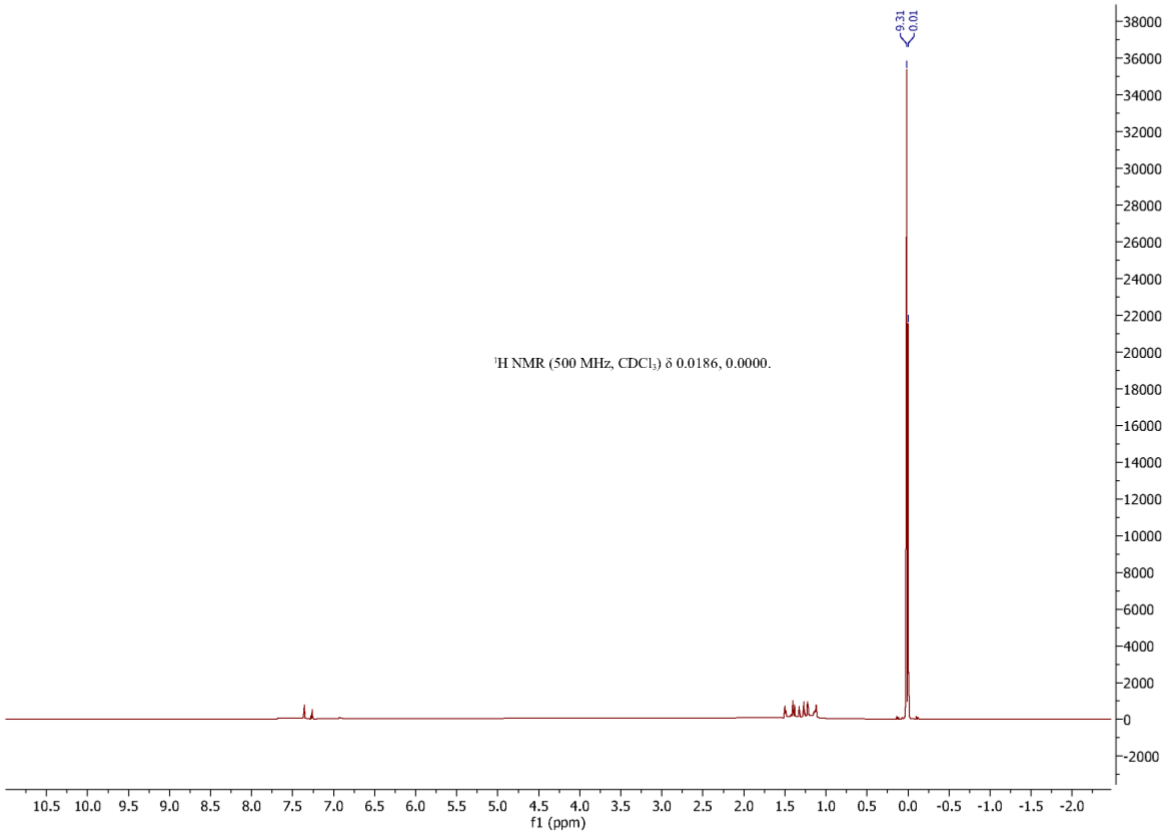

Figure S19: Evans' Method ^{1}H NMR spectrum (500 MHz, $CDCl_3$) of 20 mM [(^{Ph}isq)(^{Ph}ibq)V(O)Cl] (**II**).

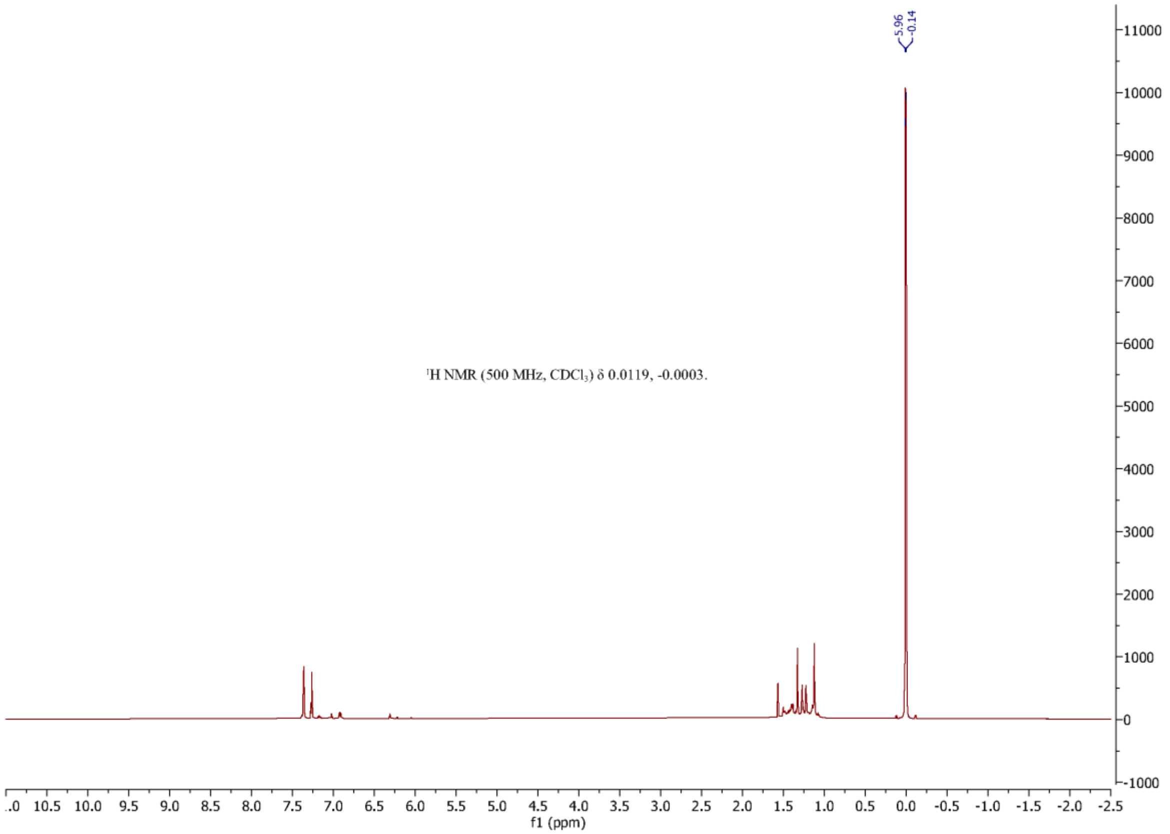

Figure S20: Evans' Method ^{1}H NMR spectrum (500 MHz, $CDCl_3$) of 10 mM [(^{Ph}isq)(^{Ph}ibq)V(O)Cl] (**II**).

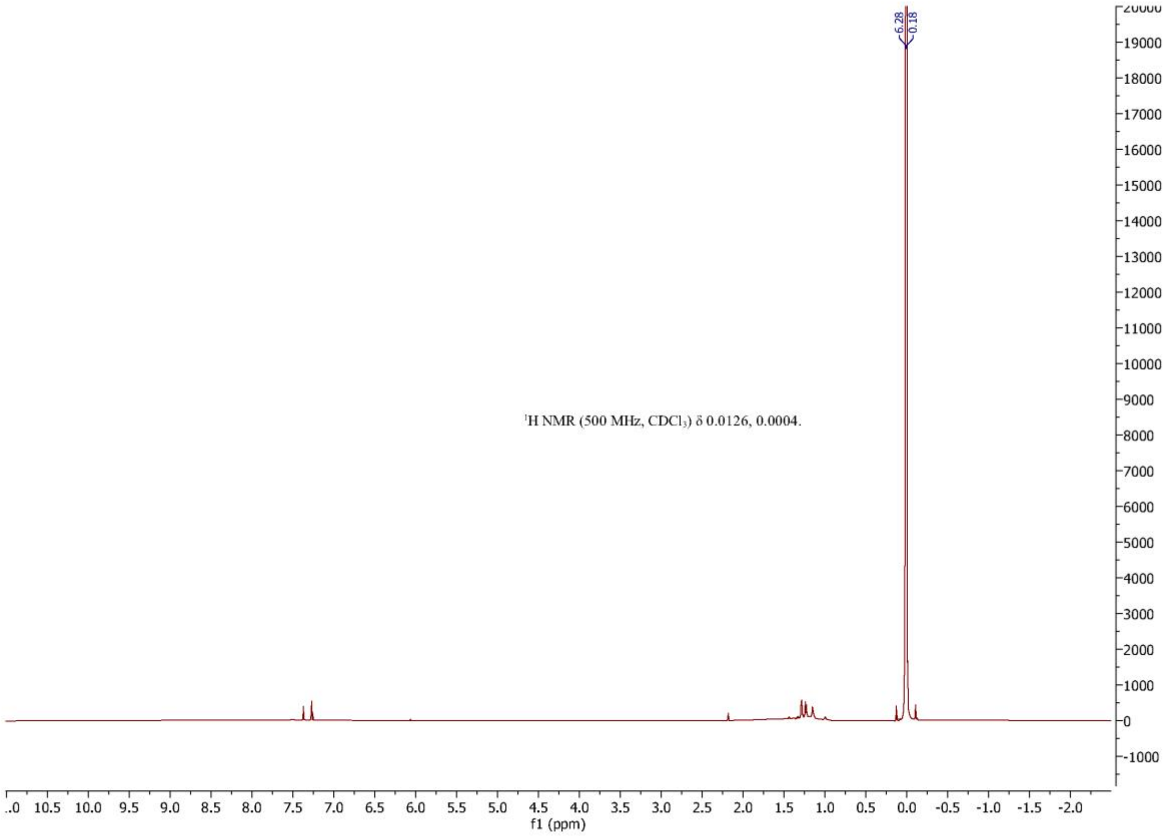

Figure S21: Evans' Method ^{1}H NMR spectrum (500 MHz, C_6D_6) of 11 mM [(^{Ph}isq)(^{Ph}ibq)V(O)Cl] (**II**).

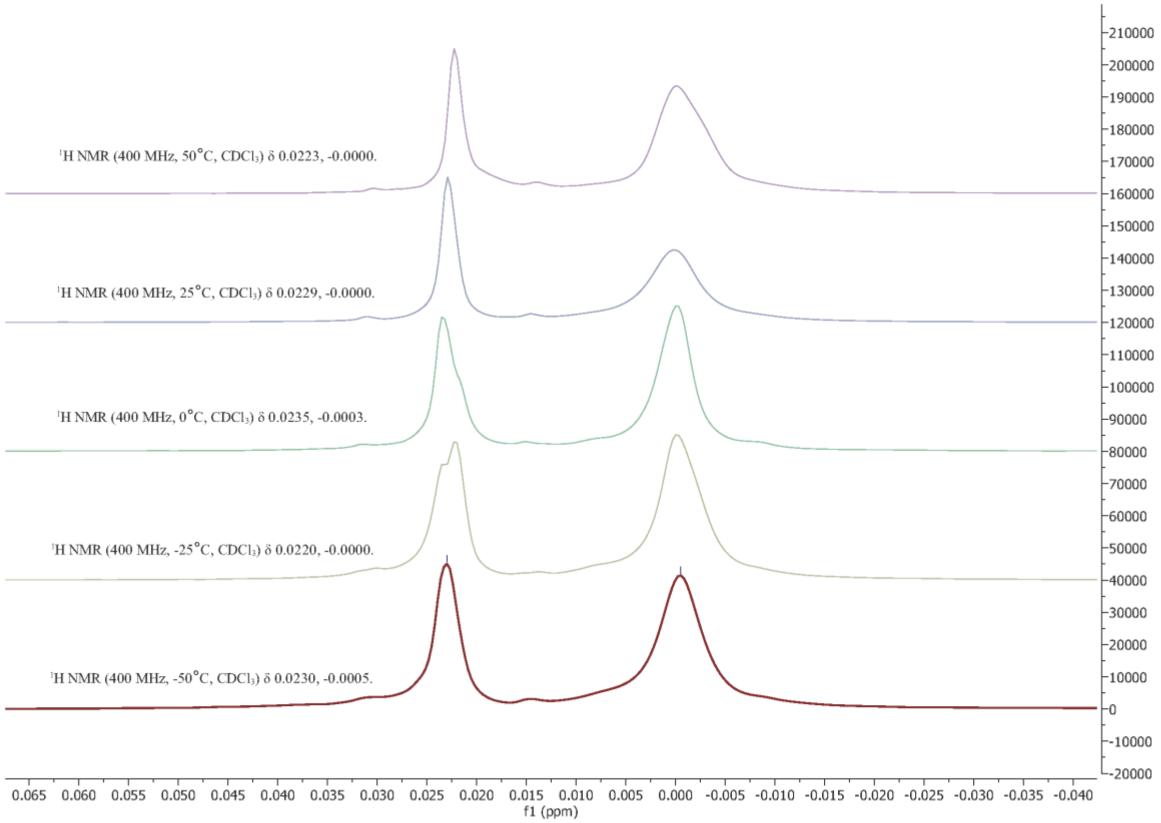

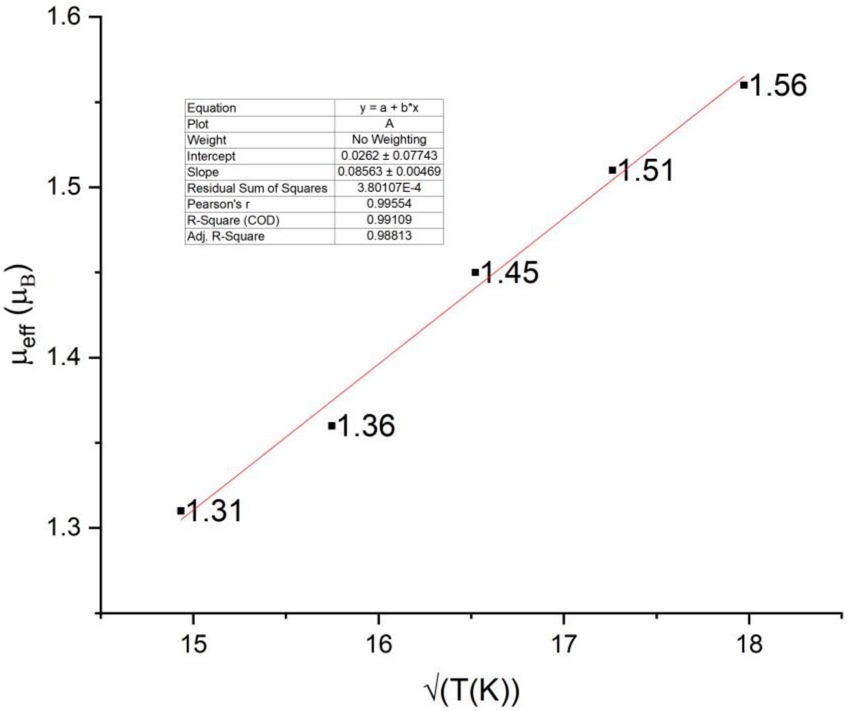

Figure S22: Evans' Method ^{1}H NMR spectrum (400 MHz, $CDCl_3$) of 11 mM [(^{Ph}isq)(^{Ph}ibq)V(O)Cl] (**II**) from -50°C to 50 °C (above) and linear fit of calculated μ_{eff} vs. $\sqrt{T}$ (below).

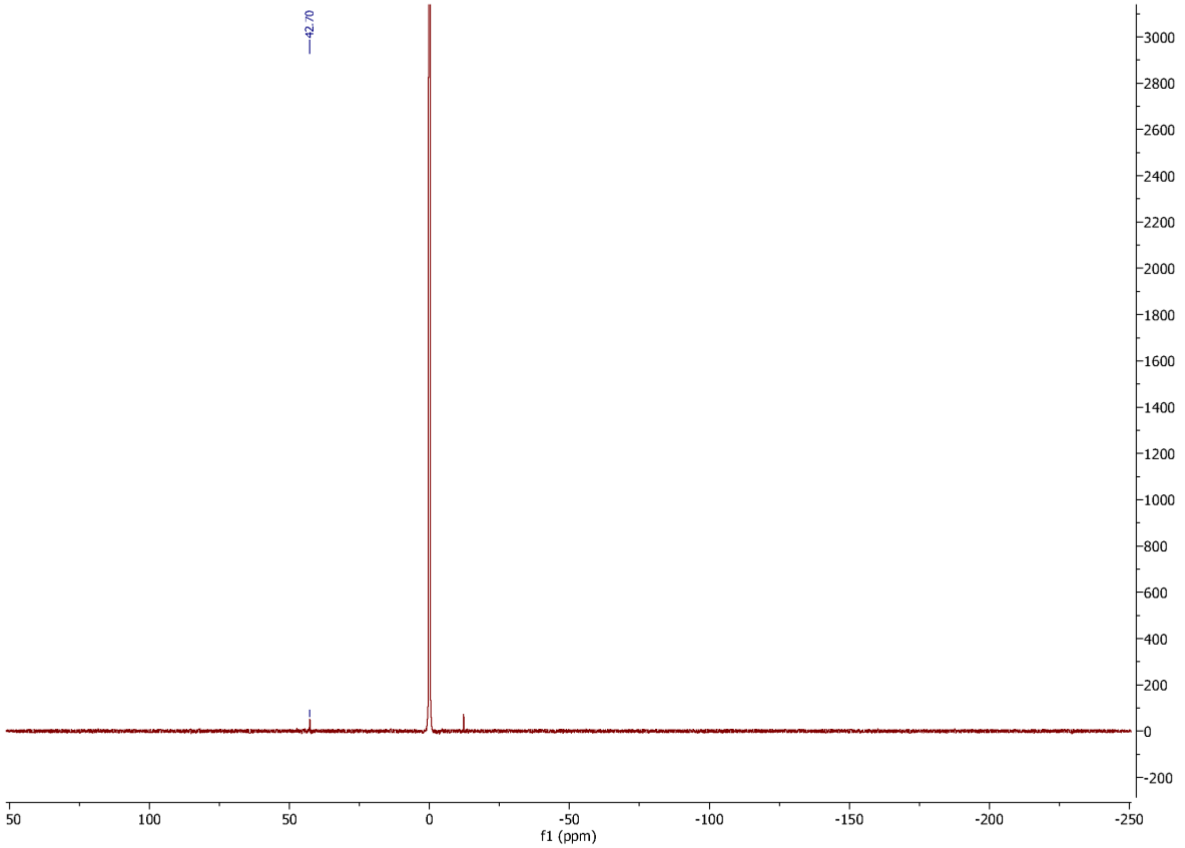

Figure S23: ^{31}P NMR spectrum (500 MHz, C_6D_6) of the products of the reaction of complex **II** and triphenylphosphine. The signal at –12 ppm is an impurity in the phosphoric acid pseudo-internal standard.

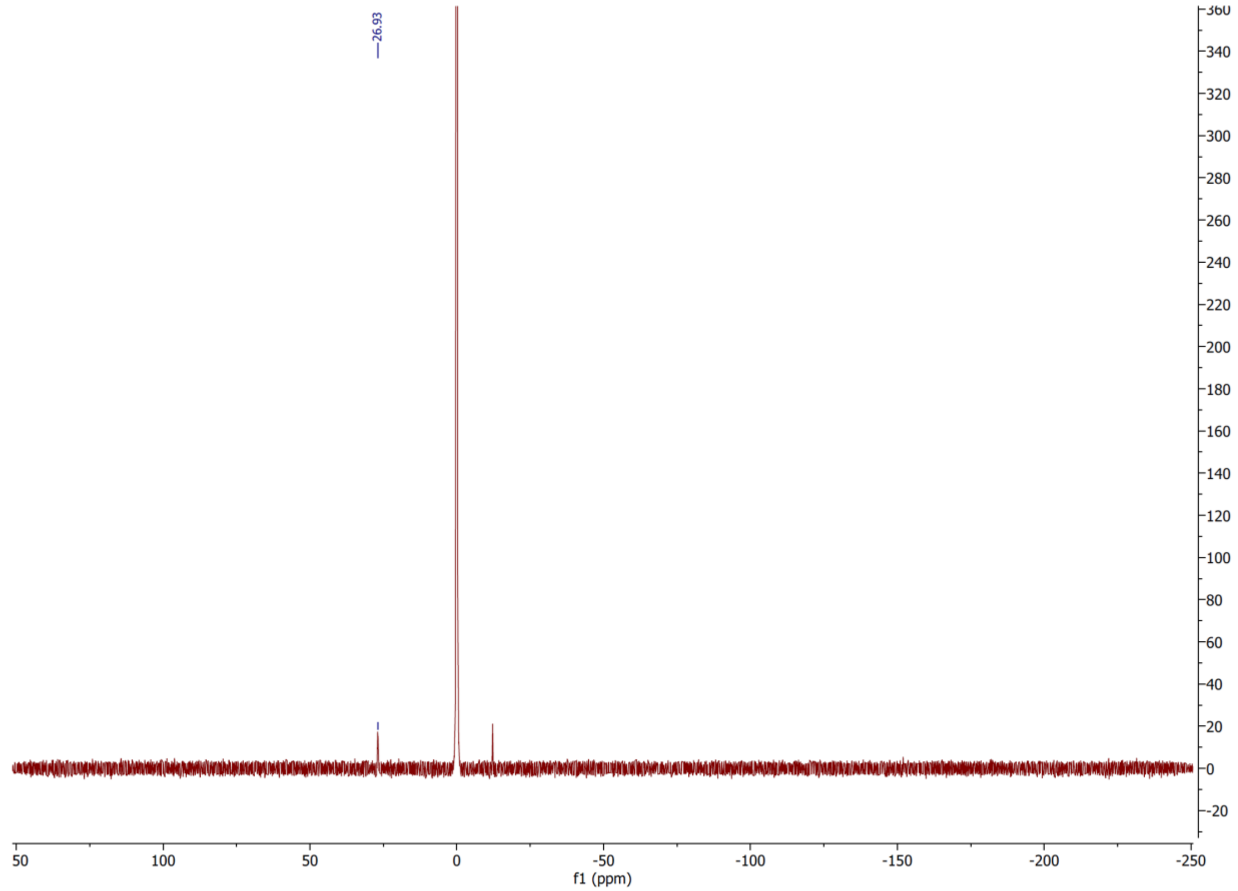

Figure S24: ^{31}P NMR spectrum (500 MHz, C_6D_6) of the products of the reaction of complex **II** and triphenylphosphine after being open to air for 24 hours. The signal at –12 ppm is an impurity in the phosphoric acid pseudo-internal standard.

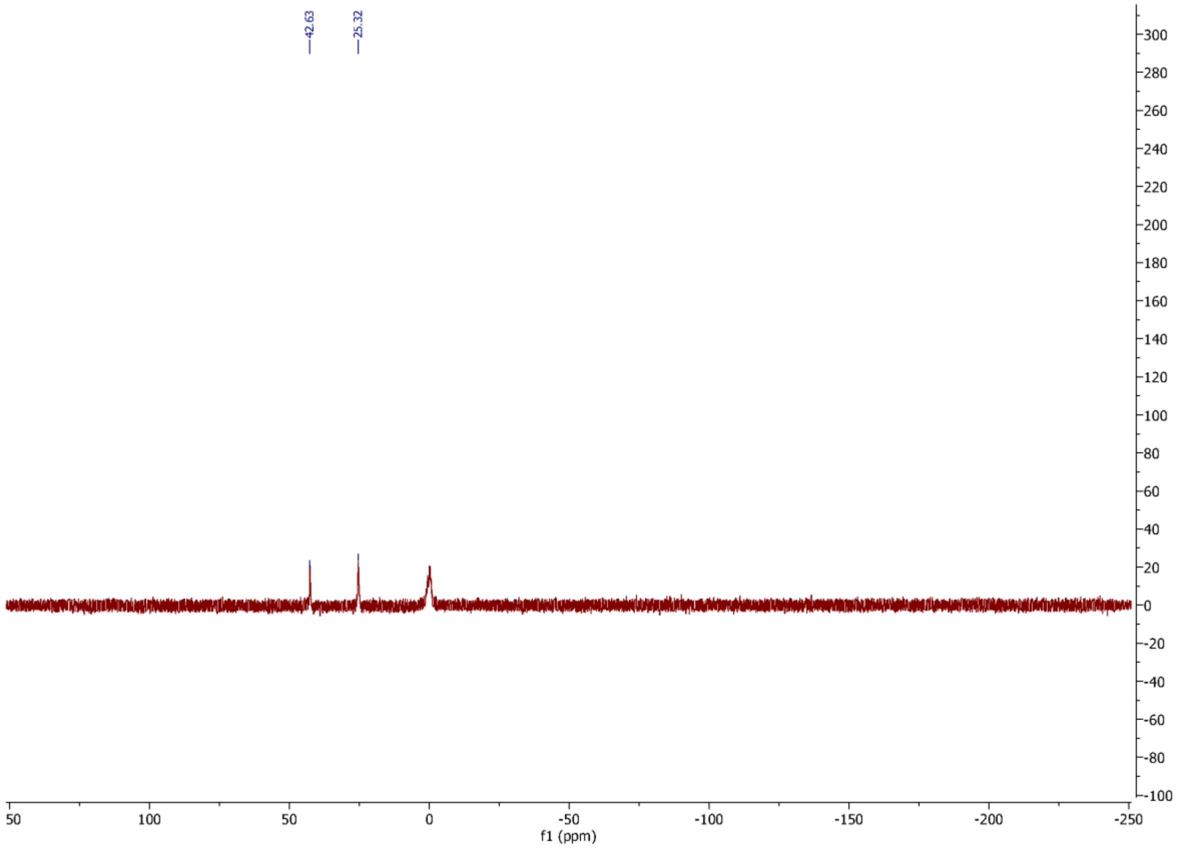

Figure S25: ^{31}P NMR spectrum (500 MHz, C_6D_6) of the products of the reaction of complex **I** and triphenylphosphine oxide.

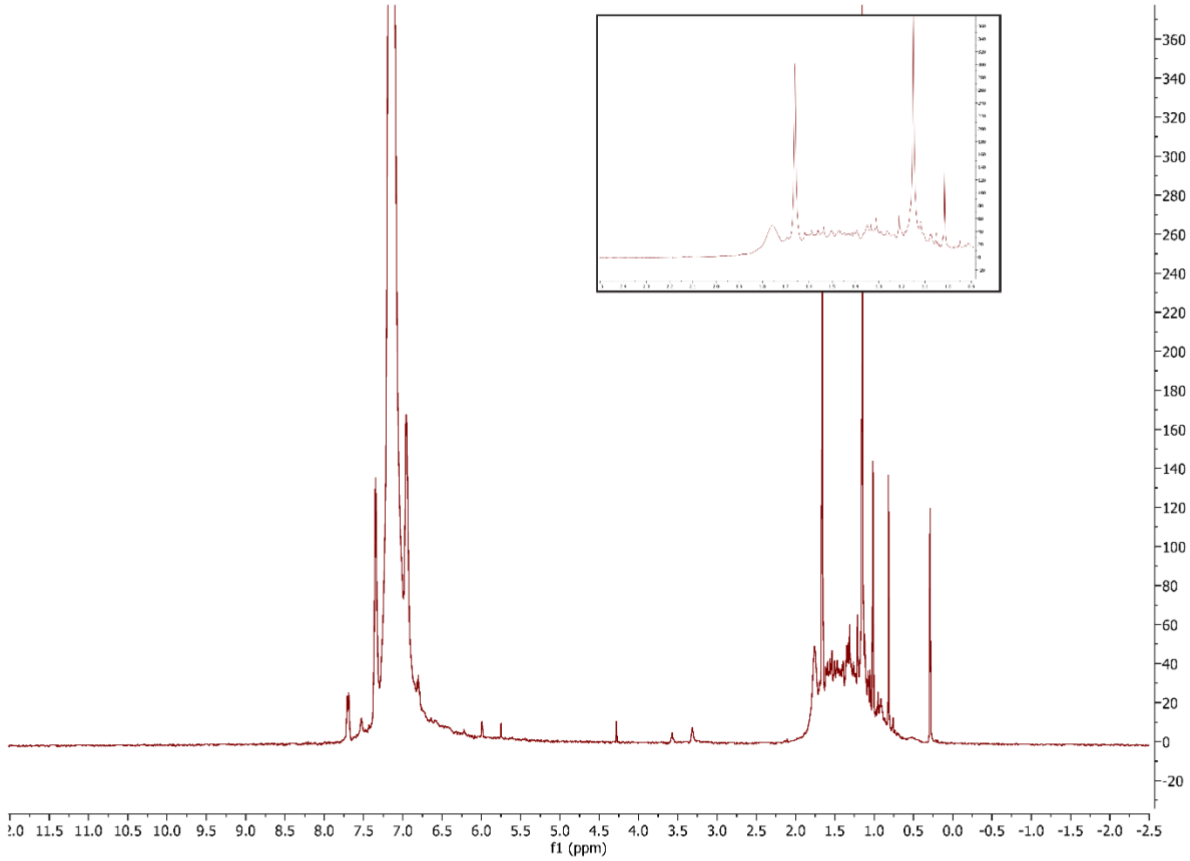

Figure S26: ^{1}H NMR spectrum (500 MHz, C_6D_6) of the products of the reaction between **II** and one equivalent DMSO.

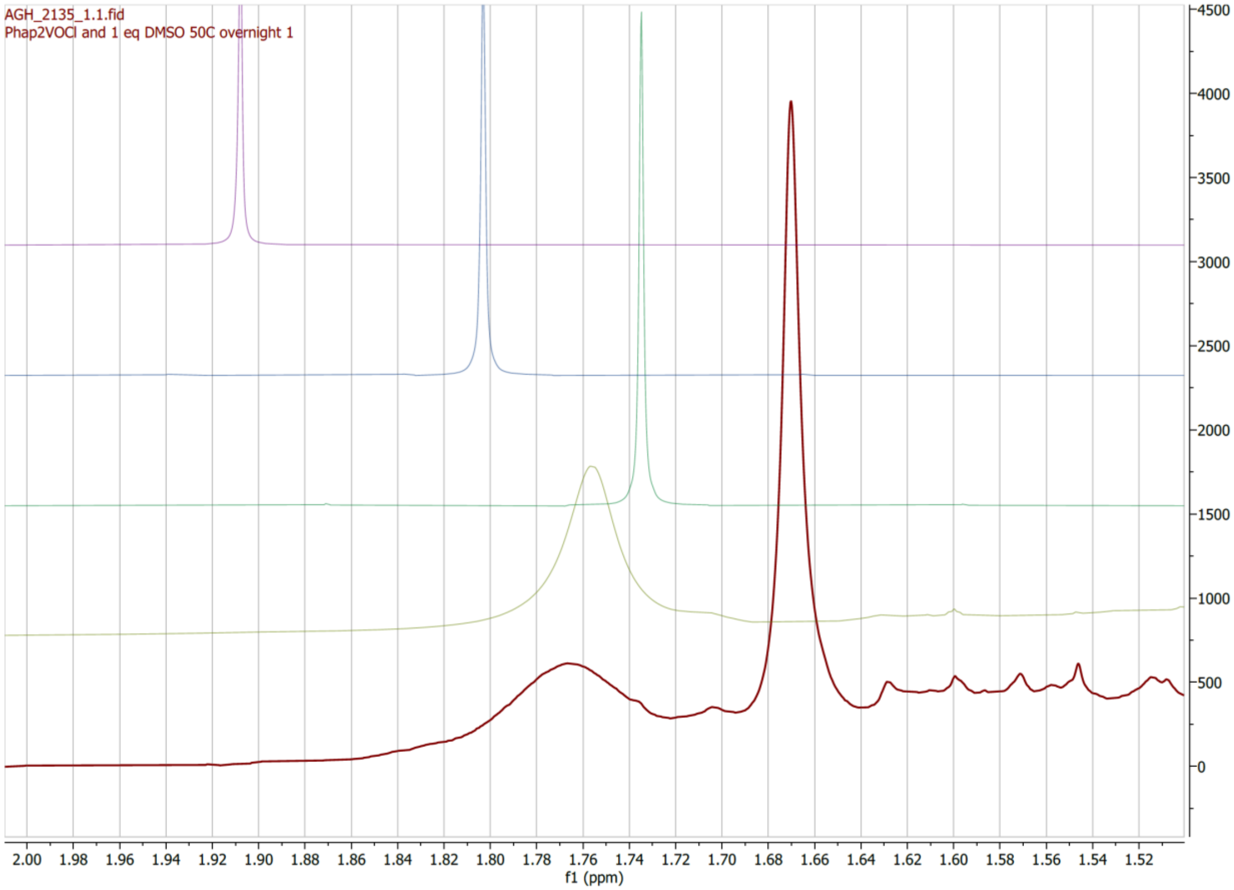

Figure S27: ^{1}H NMR spectra (500 MHz, C_6D_6) of: the products of the reaction between **II** and one equivalent DMSO (red), the products of the reaction between **II** and one equivalent DMS (yellow), authentic DMS (green), authentic DMSO (blue), and authentic $DMSO_2$ (violet).

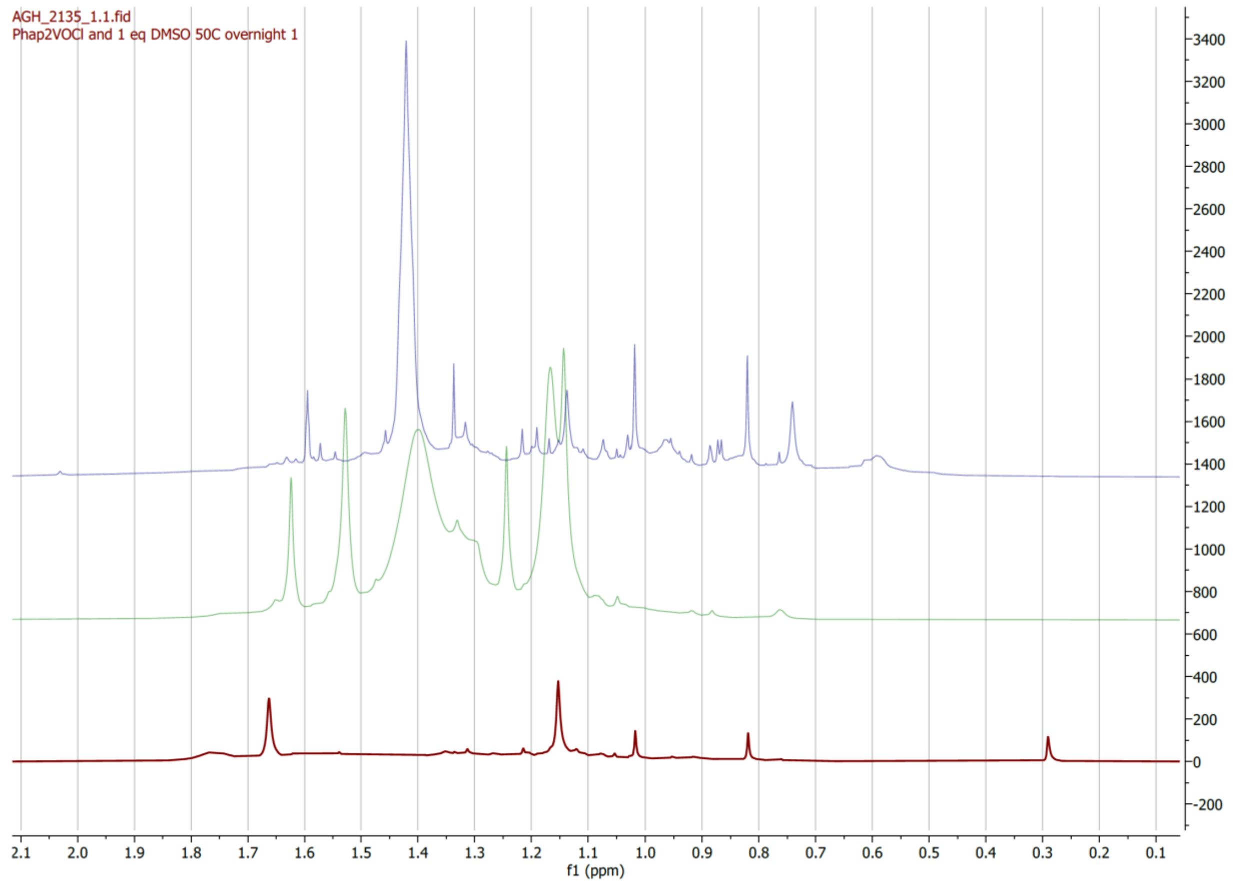

Figure S28: ^{1}H NMR spectra (500 MHz, C_6D_6) of: the products of the reaction between **II** and one equivalent DMSO (red), authentic complex **I** (green), and authentic complex **II** (blue).

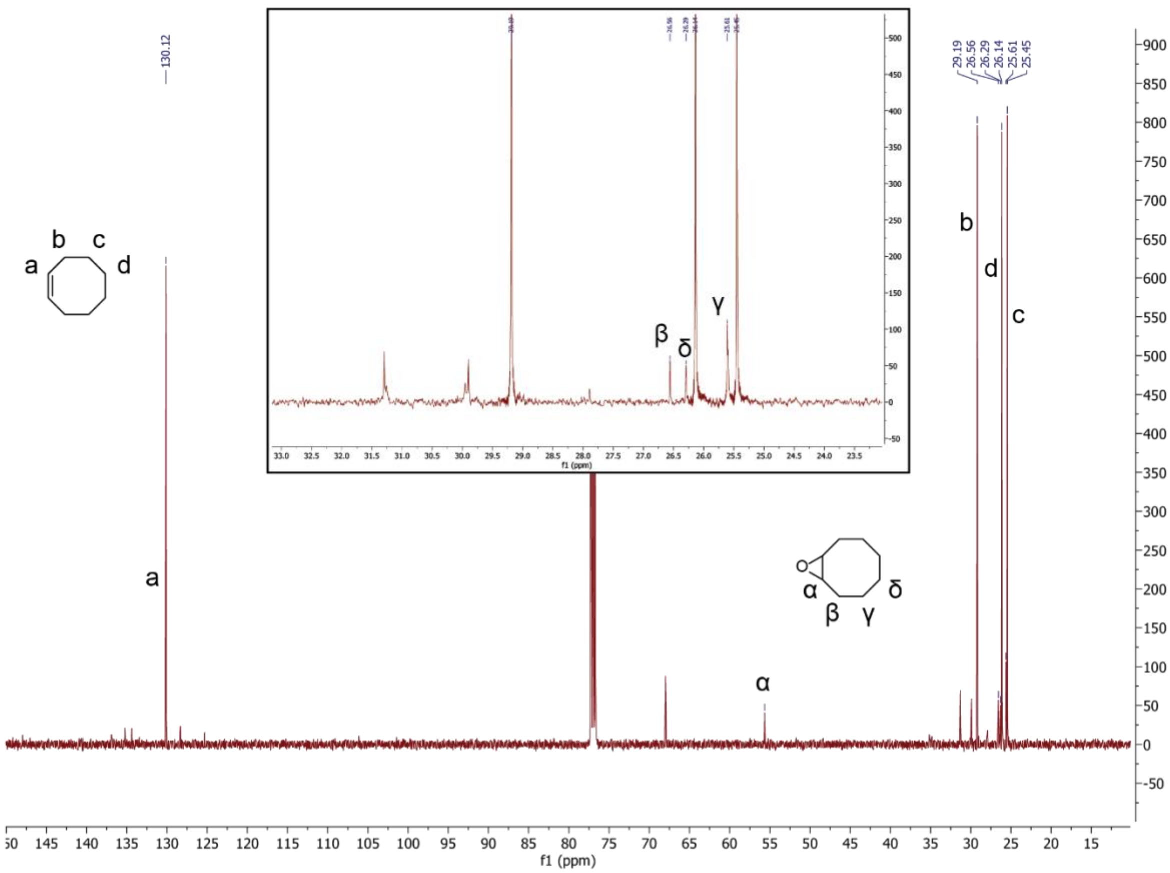

Figure S29: ^{13}C NMR spectrum (500 MHz, $CDCl_3$) of the reaction products of **II** and 8 equivalents cis-cyclooctene.

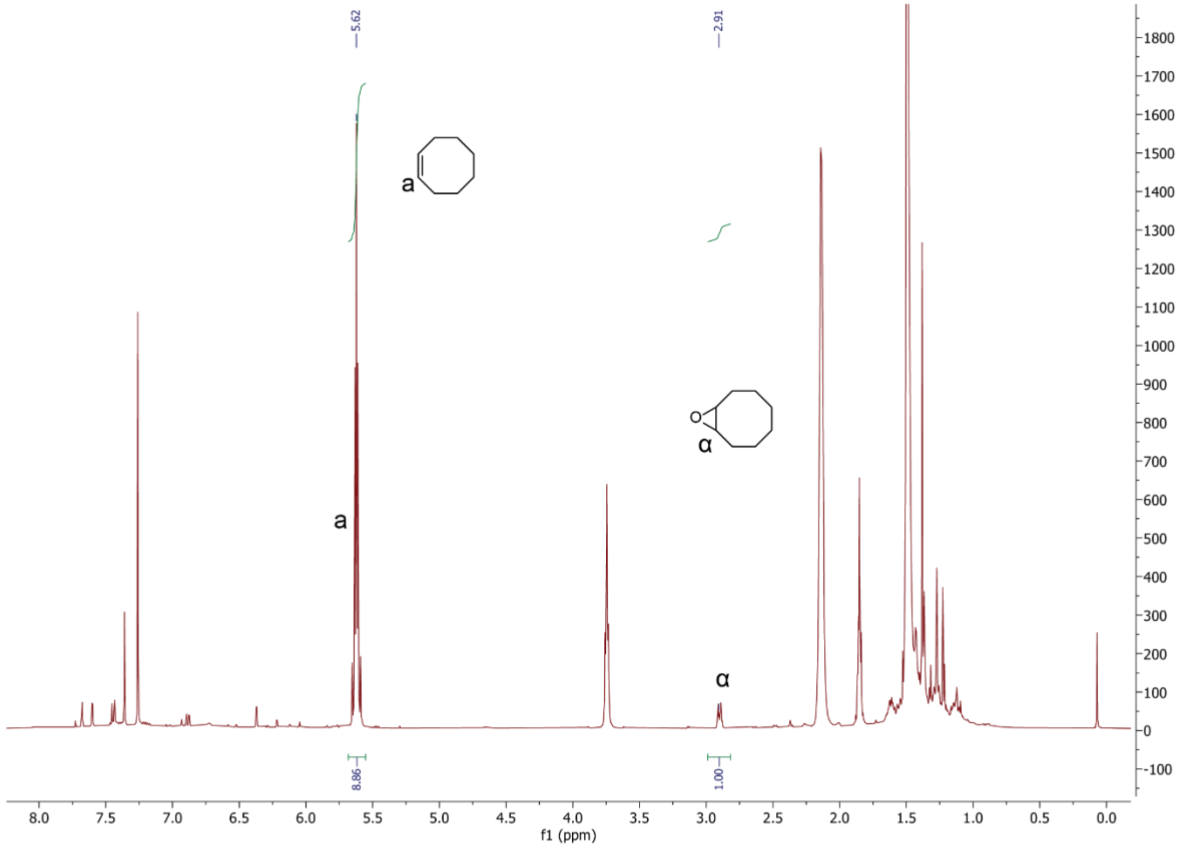

Figure S30: ^{1}H NMR spectrum (500 MHz, $CDCl_3$) of the reaction products of **II** and 8 equivalents cis-cyclooctene.

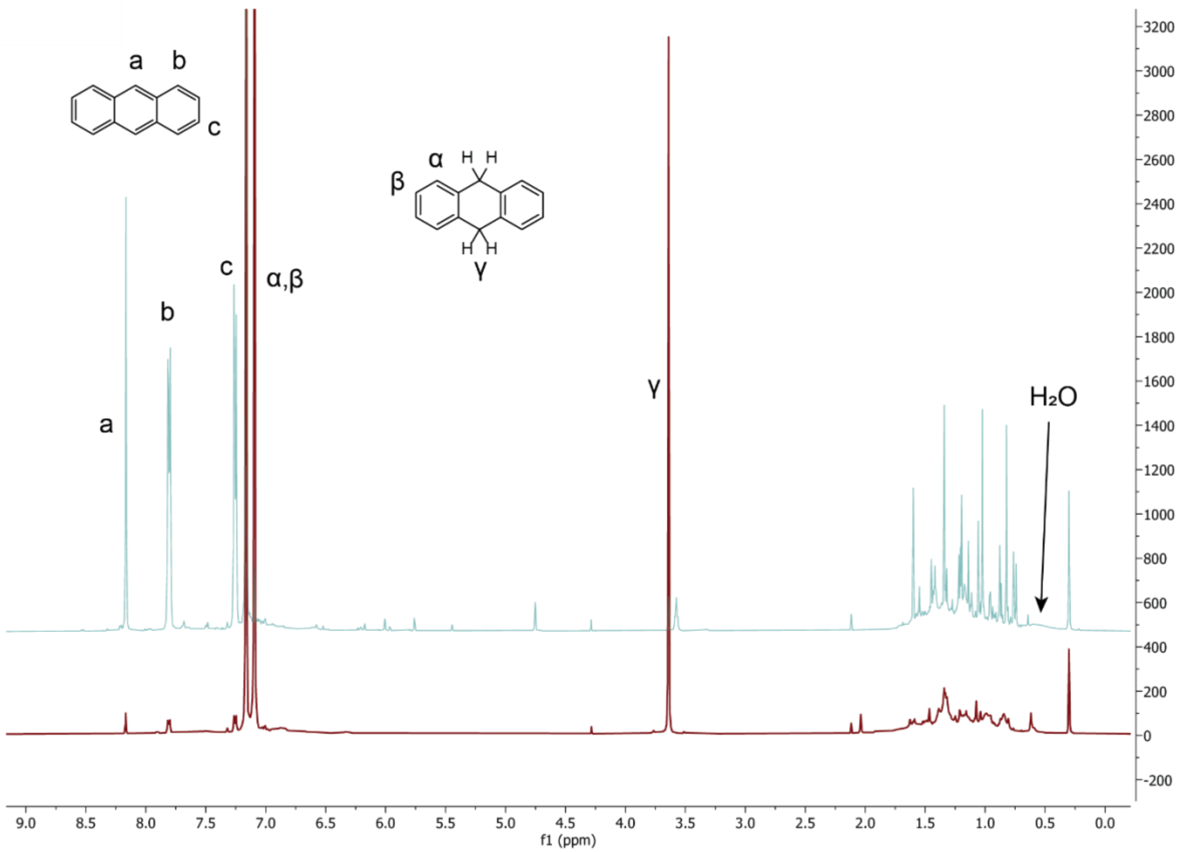

Figure S31: ^{1}H NMR spectra (500 MHz, C_6D_6) of 1:1 ratio of complex **II** and 9,10-dihydroanthracene after 20 hours at room temperature (red) and 20 hours at 50°C (blue).

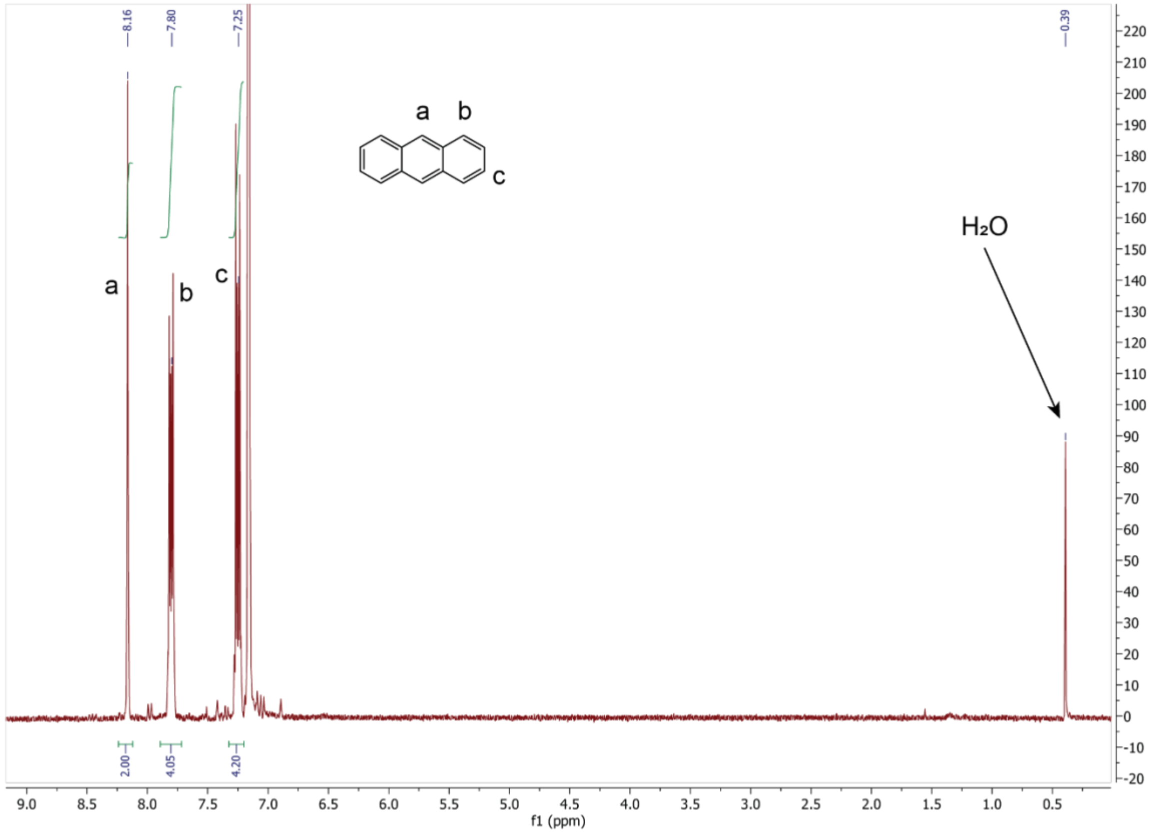

Figure S32: ^{1}H NMR spectrum (300 MHz, C_6D_6) of 1:10 ratio of complex **II** and 9,10-dihydroanthracene after 20 hours open to air at 50°C.

Crystal Data and Experimental for [(^{Ph}ap)(^{Ph}isq)V(THF)Cl] (I)

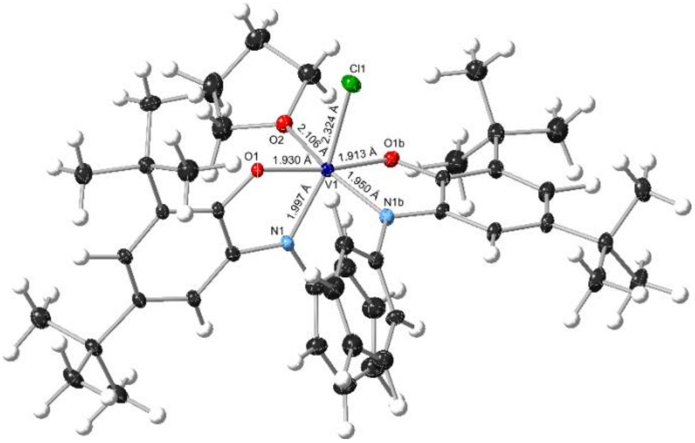

Compound	[(^{Ph}ap)(^{Ph}isq)V(THF)Cl]
Formula	$C_{48}H_{66}ClN_2O_4V$
$D_{calc.}$/ g cm^{-3}	1.237
m/mm^{-1}	0.330
Formula Weight	821.464
Colour	blue
Shape	prism-shaped
Size/mm^3	0.14×0.09×0.06
T/K	100(2)
Crystal System	monoclinic
Space Group	$P2_1/n$
a/Å	10.1847(4)
b/Å	30.5539(11)
c/Å	14.2056(6)
a/°	90
b/°	94.048(2)
g/°	90
V/Å^3	4409.5(3)
Z	4
Z'	1
Wavelength/Å	0.71073
Radiation type	MoK_a
Q_{min}/°	1.96
Q_{max}/°	25.68
Measured Refl's.	45980
Indep't Refl's	8371
Refl's I≥2 *s*(I)	5447
R_{int}	0.1664
Parameters	704
Restraints	226
Largest Peak	0.7429
Deepest Hole	-0.8086
GooF	1.1016
wR_2 (all data)	0.1696
wR_2	0.1362
R_1 (all data)	0.1334
R_1	0.0756

Experimental. A blue prism-shaped-shaped crystal with dimensions 0.14 × 0.09 × 0.06 mm^3 was mounted. Data were collected using a Bruker D8 VENTURE diffractometer operating at *T* = 100(2) K. Data were measured using *f* and *w* scans with a narrow frame width with MoK_a radiation. The diffraction pattern was indexed and the total number of runs and images was based on the strategy calculation from the program APEX2 v2016.5-1.[2] The maximum resolution that was achieved was *Q* = 25.68° (0.82 Å). The unit cell was refined using SAINT v8.37A[3] on 4856 reflections, 11% of the observed reflections. Data reduction, scaling and absorption corrections were performed using SAINT v8.37A.[3] The final completeness is 99.89 % out to 25.68° in *Q*. No absorption correction was performed. The absorption coefficient *m* of this material is 0.330 mm^{-1} at this wavelength (*l* = 0.71073Å) and the minimum and maximum transmissions are 0.599 and 0.745. The structure was solved and the space group $P2_1/n$ (# 14) determined by the ShelXT[4] structure solution program using using dual methods and refined by full matrix least squares minimisation on $\boldsymbol{F^2}$ using version of ShelXL-2014.[5] All non-hydrogen atoms were refined anisotropically. Hydrogen atom positions were calculated geometrically and refined using the riding model.

Refinement using NoSpherA2, an implementation of NOn-SPHERical Atom-form-factors in Olex2.[6] NoSpherA2 implementation of HAR makes use of tailor-made aspherical atomic form factors calculated on-the-fly from a Hirshfeld-partitioned electron density (ED) - not from spherical-atom form factors. The ED is calculated from a gaussian basis set single determinant SCFwavefunction - either Hartree-Fock or DFT using selected funtionals - for a fragment of the crystal. This fragment can be embedded in an electrostatic crystal field by employing cluster charges or modelled using implicit solvation models, depending on the software used. The following options were used: SOFTWARE: ORCA 5.0 PARTITIONING: NoSpherA2 INT ACCURACY: Normal METHOD: PBE BASIS SET: def2-SVP CHARGE: 0 MULTIPLICITY: 1 SOLVATION: THF DATE: 2022-08-03_17-44-06

Table S1: Fractional Atomic Coordinates (×10^4) and Equivalent Isotropic Displacement Parameters (Å^2×10^3) for **[(^{Ph}ap)(^{Ph}isq)V(THF)Cl]**. U_{eq} is defined as 1/3 of the trace of the orthogonalised U_{ij}.

Atom	x	y	z	U_{eq}
V1	1150.0(7)	3763.7(2)	5329.4(5)	11.83(19)
Cl1	-994.2(10)	4001.0(4)	4968.2(8)	20.3(3)
O1	1256(3)	4228.3(9)	6252(2)	12.7(6)
O1b	865(3)	3260.1(9)	4541(2)	15.4(7)
N1	3003(3)	3717.1(10)	5881(2)	11.6(7)
N1b	1807(3)	3981.6(11)	4161(3)	15.0(8)
C1	3357(4)	3984.2(13)	6633(3)	12.4(8)
C1b	1539(4)	3722.8(13)	3388(3)	13.2(9)
C2	2317(4)	4275.0(13)	6810(3)	12.6(8)
C2b	1026(4)	3308.2(14)	3634(3)	15.0(9)
C3	2456(4)	4577.0(13)	7573(3)	12.4(8)
C3b	728(4)	2983.4(14)	2938(3)	16.2(10)
C4	3644(4)	4563.6(13)	8120(3)	13.9(9)
C4b	932(4)	3102.4(14)	2005(3)	17.8(10)
C5	4687(4)	4272.0(13)	7953(3)	13.6(9)
C5b	1406(4)	3520.2(14)	1736(3)	17.5(10)
C6	4543(4)	3985.1(13)	7202(3)	13.4(9)
C6b	1707(4)	3826.1(14)	2446(3)	16.9(9)
C7	3961(4)	3450.0(14)	5474(3)	19.7(10)
C7b	2631(4)	4352.3(13)	4083(3)	15.4(9)
C8	3621(5)	3017.1(15)	5235(4)	24.5(11)
C8b	2243(4)	4757.2(14)	4430(3)	19.2(10)
C9	4519(5)	2749.9(16)	4818(4)	28.2(11)
C9b	3064(5)	5118.9(15)	4353(3)	22.9(11)
C10	5763(5)	2899.8(16)	4655(4)	28.6(11)
C10b	4268(5)	5075.5(15)	3967(3)	23.9(11)
C11	6096(5)	3330.0(16)	4877(4)	25.4(11)
C11b	4663(5)	4675.8(15)	3635(3)	24.6(11)
C12	5206(4)	3605.8(16)	5274(3)	22.2(10)
C12b	3840(4)	4313.0(15)	3686(3)	18.0(10)
C13	1343(4)	4895.2(14)	7763(3)	16.7(9)
C13b	167(4)	2541.1(14)	3213(3)	20.4(10)
C14	76(4)	4648.3(16)	7928(4)	25.1(11)
C14b	-1176(5)	2610.6(16)	3614(4)	25.2(11)
C15	1109(5)	5204.1(15)	6917(4)	25.1(11)
C15b	1115(5)	2312.5(15)	3928(4)	28.2(12)
C16	1694(5)	5175.5(16)	8645(3)	24.0(11)
C16b	-55(5)	2240.4(15)	2357(4)	28.0(12)
C17	5949(4)	4292.2(14)	8611(3)	18.0(9)
C17b	1535(4)	3621.2(15)	690(3)	20.7(10)
C18	6497(5)	4760.1(15)	8655(4)	24.2(11)
C18b	2402(5)	3271.7(17)	251(4)	30.2(12)
C19	5623(5)	4148.5(17)	9605(4)	27.6(11)

Atom	x	y	z	U_{eq}
C19b	162(5)	3617.4(17)	169(4)	27.9(12)
C20	7022(4)	3990.2(16)	8284(4)	24.6(11)
C20b	2155(5)	4070.9(16)	545(3)	26.1(11)
O1s	-4067(5)	3081.3(16)	1600(5)	93(2)
C1s	-2846(6)	3225.1(19)	1337(5)	42.6(15)
C2s	-2429(6)	3596.6(18)	1995(5)	42.4(15)
C3s	-3707(6)	3757.2(19)	2358(5)	41.3(14)
C4s	-4597(6)	3362(2)	2225(5)	49.7(16)
O2	568(3)	3312.0(9)	6340(2)	18.1(7)
C21	952(5)	3332.1(16)	7342(4)	29.0(12)
C22	292(6)	2945.5(18)	7758(4)	35.0(13)
C23	-973(5)	2900.6(18)	7127(4)	33.5(13)
C24	-496(5)	2994.8(16)	6167(4)	26.4(11)

Table S2: Anisotropic Displacement Parameters (×10^4) for **[(^{Ph}ap)(^{Ph}isq)V(THF)Cl]**. The anisotropic displacement factor exponent takes the form: $-2p^2[h^2a^{*2} \times U_{11} + ... + 2hka^* \times b^* \times U_{12}]$

Atom	U_{11}	U_{22}	U_{33}	U_{23}	U_{13}	U_{12}
V1	12.7(4)	11.9(4)	10.9(4)	-1.6(3)	0.7(3)	-0.6(3)
Cl1	15.6(5)	20.9(6)	23.7(6)	1.1(4)	-3.0(5)	-0.9(5)
O1	12.4(13)	16.4(15)	9.3(15)	-0.7(10)	0.4(9)	-1.8(11)
O1b	17.8(15)	13.4(15)	15.3(17)	-2.7(12)	3.4(13)	-1.5(13)
N1	14.3(16)	9.0(15)	11.0(17)	-2.7(10)	-1.6(10)	-0.0(10)
N1b	18.1(19)	14.4(18)	13(2)	-5.5(15)	0.8(15)	-1.8(15)
C1	14.7(16)	9.3(17)	13.0(18)	-4.7(10)	0.3(10)	0.6(11)
C1b	19(2)	11(2)	10(2)	-2.9(17)	5.2(17)	2.0(18)
C2	16.3(16)	11.2(18)	10.0(18)	-2.8(10)	-1.1(10)	-0.7(11)
C2b	18(2)	14(2)	12(2)	-2.9(18)	2.3(18)	0.6(18)
C3	13.8(16)	12.1(18)	11.1(19)	-1.7(10)	-0.0(10)	-1.6(11)
C3b	20(2)	13(2)	16(3)	-1.6(18)	0.8(19)	-2.8(18)
C4	14.0(16)	11.9(19)	15(2)	-2.8(11)	-0.9(11)	-1.5(13)
C4b	26(2)	13(2)	15(3)	-1.8(18)	4.5(19)	-0.4(19)
C5	13.8(17)	14.2(19)	12.5(19)	-1.4(10)	-1.8(10)	-0.8(11)
C5b	24(2)	19(2)	10(2)	0.4(19)	2.4(19)	-0.2(19)
C6	15.4(18)	11.7(19)	13.0(19)	-1.4(12)	-1.3(11)	-0.8(12)
C6b	25(2)	16(2)	11(2)	-5.5(19)	4.8(18)	0.7(18)
C7	19.2(18)	15.1(18)	25(2)	1.8(10)	2.9(12)	-2.9(12)
C7b	20(2)	11(2)	15(2)	-4.4(18)	1.9(19)	-1.6(18)
C8	22(2)	19.4(19)	32(3)	0.6(12)	2.5(16)	-5.7(13)
C8b	22(2)	15(2)	20(3)	-1.3(19)	2(2)	0.1(19)
C9	27(2)	22(2)	36(3)	1.2(13)	6.0(15)	-8.8(16)
C9b	32(3)	11(2)	26(3)	0(2)	6(2)	0(2)
C10	24(2)	27(2)	36(3)	3.4(13)	4.0(16)	-3.1(16)
C10b	33(3)	15(2)	23(3)	-10(2)	2(2)	3(2)
C11	19(2)	28(2)	30(3)	1.9(13)	7.1(15)	-1.4(15)
C11b	27(3)	22(3)	25(3)	-9(2)	5(2)	0(2)
C12	18.4(19)	23(2)	26(3)	1.2(12)	3.6(13)	-0.4(15)
C12b	17(2)	18(2)	20(3)	-4.2(19)	6.9(19)	-2(2)
C13	15.3(18)	16.6(18)	18(2)	1.9(11)	1.3(11)	-3.8(12)
C13b	28(3)	16(2)	17(3)	-1.4(19)	0(2)	-2.2(19)
C14	17(2)	33(3)	27(3)	-3.7(14)	7.1(16)	-6.7(19)
C14b	27(3)	21(3)	28(3)	-8(2)	5(2)	0(2)
C15	28(3)	22(2)	26(2)	5.4(17)	0.5(16)	-0.3(14)
C15b	38(3)	14(2)	32(3)	-2(2)	2(2)	-2(2)
C16	25(2)	27(2)	20(2)	3.6(17)	1.1(16)	-7.1(15)
C16b	42(3)	15(2)	28(3)	-7(2)	6(2)	-3(2)

Atom	U_{11}	U_{22}	U_{33}	U_{23}	U_{13}	U_{12}
C17	14.5(18)	19.9(19)	18(2)	-0.8(11)	-5.2(11)	0.9(12)
C17b	28(3)	21(2)	13(2)	-1(2)	2(2)	-1.5(19)
C18	20(2)	22(2)	29(3)	-5.4(13)	-8.8(19)	0.2(15)
C18b	45(3)	30(3)	17(3)	1(2)	10(2)	-5(2)
C19	24(3)	37(3)	21(2)	0.7(19)	-4.2(14)	5.4(15)
C19b	34(3)	34(3)	15(3)	-8(2)	-3(2)	2(2)
C20	17(2)	26(2)	30(3)	0.6(14)	-5.8(16)	-2.9(18)
C20b	41(3)	25(3)	14(3)	-10(2)	9(2)	1(2)
O1s	90(4)	60(3)	135(5)	-31(3)	65(4)	-46(3)
C1s	42(3)	37(3)	50(4)	5(3)	6(3)	-1(3)
C2s	38(3)	30(3)	60(4)	5(3)	11(3)	1(3)
C3s	42(3)	37(3)	47(4)	5(3)	18(3)	0(3)
C4s	40(4)	59(4)	49(4)	-1(3)	2(3)	-7(3)
O2	21.1(16)	18.9(16)	14.5(17)	-6.2(13)	3.1(13)	1.7(13)
C21	41(3)	25(3)	21(3)	-15(2)	4(2)	7(2)
C22	56(4)	30(3)	20(3)	-12(3)	5(3)	5(2)
C23	35(3)	37(3)	31(3)	-13(2)	14(2)	4(3)
C24	25(3)	25(3)	29(3)	-7(2)	1(2)	6(2)

Table S3: Bond Lengths in Å for **[(^{Ph}ap)(^{Ph}isq)V(THF)Cl]**.

Atom	Atom	Length/Å
V1	Cl1	2.3238(12)
V1	O1	1.930(3)
V1	O1b	1.913(3)
V1	N1	1.997(3)
V1	N1b	1.950(4)
V1	O2	2.107(3)
O1	C2	1.302(5)
O1b	C2b	1.318(5)
N1	C1	1.372(5)
N1	C7	1.425(5)
N1b	C1b	1.365(5)
N1b	C7b	1.418(5)
C1	C2	1.419(6)
C1	C6	1.406(6)
C1b	C2b	1.423(6)
C1b	C6b	1.397(6)
C2	C3	1.423(6)
C2b	C3b	1.419(6)
C3	C4	1.391(6)
C3	C13	1.532(6)
C3b	C4b	1.404(6)
C3b	C13b	1.529(6)
C4	C5	1.420(6)
C4b	C5b	1.426(6)
C5	C6	1.381(6)
C5	C17	1.536(6)
C5b	C6b	1.393(6)
C5b	C17b	1.532(6)
C7	C8	1.403(6)
C7	C12	1.402(6)
C7b	C8b	1.399(6)
C7b	C12b	1.396(6)
C8	C9	1.389(7)
C8b	C9b	1.395(6)
C9	C10	1.382(7)
C9b	C10b	1.384(7)
C10	C11	1.388(7)
C10b	C11b	1.379(7)
C11	C12	1.386(6)
C11b	C12b	1.395(6)
C13	C14	1.527(6)
C13	C15	1.534(6)
C13	C16	1.539(6)
C13b	C14b	1.533(6)
C13b	C15b	1.522(7)
C13b	C16b	1.528(6)
C17	C18	1.534(6)
C17	C19	1.537(7)
C17	C20	1.528(6)
C17b	C18b	1.545(7)
C17b	C19b	1.535(6)
C17b	C20b	1.532(6)
O1s	C1s	1.394(7)
O1s	C4s	1.372(8)
C1s	C2s	1.512(8)
C2s	C3s	1.515(7)
C3s	C4s	1.514(8)
O2	C21	1.450(6)
O2	C24	1.462(5)
C21	C22	1.501(7)
C22	C23	1.524(8)
C23	C24	1.507(7)

Table S4: Bond Angles in ° for **[(^{Ph}ap)(^{Ph}isq)V(THF)Cl]**.

Atom	Atom	Atom	Angle/°
O1	V1	Cl1	85.82(9)
O1b	V1	Cl1	91.17(9)
O1b	V1	O1	171.14(12)
N1	V1	Cl1	163.02(10)
N1	V1	O1	77.30(13)
N1	V1	O1b	105.80(13)
N1b	V1	Cl1	94.54(11)
N1b	V1	O1	108.71(13)
N1b	V1	O1b	79.81(13)
N1b	V1	N1	89.22(14)
O2	V1	Cl1	92.85(9)
O2	V1	O1	91.38(12)
O2	V1	O1b	80.44(12)
O2	V1	N1	89.41(13)
O2	V1	N1b	159.03(13)
C2	O1	V1	119.9(3)
C2b	O1b	V1	117.3(3)
C1	N1	V1	116.8(3)
C7	N1	V1	122.5(3)
C7	N1	C1	120.5(3)
C1b	N1b	V1	115.2(3)
C7b	N1b	V1	125.4(3)
C7b	N1b	C1b	119.1(4)
C2	C1	N1	110.7(3)
C6	C1	N1	128.1(4)
C6	C1	C2	121.2(4)
C2b	C1b	N1b	112.0(4)
C6b	C1b	N1b	127.5(4)
C6b	C1b	C2b	120.6(4)
C1	C2	O1	114.9(4)
C3	C2	O1	124.7(4)
C3	C2	C1	120.4(4)
C1b	C2b	O1b	114.4(4)
C3b	C2b	O1b	124.6(4)
C3b	C2b	C1b	121.0(4)
C4	C3	C2	116.3(4)
C13	C3	C2	120.7(4)
C13	C3	C4	123.0(4)
C4b	C3b	C2b	116.0(4)
C13b	C3b	C2b	120.4(4)
C13b	C3b	C4b	123.6(4)
C5	C4	C3	123.8(4)
C5b	C4b	C3b	124.0(4)
C6	C5	C4	119.2(4)
C17	C5	C4	118.5(4)
C17	C5	C6	122.3(4)
C6b	C5b	C4b	117.9(4)
C17b	C5b	C4b	119.5(4)
C17b	C5b	C6b	122.5(4)
C5	C6	C1	119.1(4)
C5b	C6b	C1b	120.4(4)
C8	C7	N1	118.3(4)

Atom	Atom	Atom	Angle/°
C12	C7	N1	122.9(4)
C12	C7	C8	118.8(4)
C8b	C7b	N1b	119.7(4)
C12b	C7b	N1b	120.4(4)
C12b	C7b	C8b	119.8(4)
C9	C8	C7	119.9(4)
C9b	C8b	C7b	119.1(4)
C10	C9	C8	121.0(5)
C10b	C9b	C8b	120.5(4)
C11	C10	C9	119.2(5)
C11b	C10b	C9b	120.5(4)
C12	C11	C10	120.8(5)
C12b	C11b	C10b	119.7(5)
C11	C12	C7	120.2(4)
C11b	C12b	C7b	120.2(4)
C14	C13	C3	110.9(4)
C15	C13	C3	109.2(4)
C15	C13	C14	109.8(4)
C16	C13	C3	111.4(4)
C16	C13	C14	107.4(4)
C16	C13	C15	108.1(4)
C14b	C13b	C3b	109.4(4)
C15b	C13b	C3b	110.2(4)
C15b	C13b	C14b	110.9(4)
C16b	C13b	C3b	111.4(4)
C16b	C13b	C14b	107.0(4)
C16b	C13b	C15b	107.9(4)
C18	C17	C5	110.4(4)
C19	C17	C5	108.8(4)
C19	C17	C18	109.3(4)
C20	C17	C5	112.1(4)
C20	C17	C18	108.0(4)
C20	C17	C19	108.2(4)
C18b	C17b	C5b	110.0(4)
C19b	C17b	C5b	109.2(4)
C19b	C17b	C18b	109.1(4)
C20b	C17b	C5b	112.1(4)
C20b	C17b	C18b	108.2(4)
C20b	C17b	C19b	108.2(4)
C4s	O1s	C1s	112.1(5)
C2s	C1s	O1s	106.7(5)
C3s	C2s	C1s	104.0(5)
C4s	C3s	C2s	102.8(5)
C3s	C4s	O1s	108.6(5)
C21	O2	V1	124.9(3)
C24	O2	V1	124.1(3)
C24	O2	C21	109.8(3)
C22	C21	O2	104.9(4)
C23	C22	C21	102.8(4)
C24	C23	C22	101.7(4)
C23	C24	O2	104.9(4)

Table S5: Torsion Angles in ° for **[(^{Ph}ap)(^{Ph}isq)V(THF)Cl]**.

Atom	Atom	Atom	Atom	Angle/°
V1	O1	C2	C1	4.0(3)
V1	O1	C2	C3	-173.6(3)
V1	O1b	C2b	C1b	6.8(3)
V1	O1b	C2b	C3b	-173.3(3)
V1	N1	C1	C2	-5.3(3)
V1	N1	C1	C6	173.1(3)
V1	N1	C7	C8	-48.5(4)
V1	N1	C7	C12	129.3(3)
V1	N1b	C1b	C2b	-10.1(3)
V1	N1b	C1b	C6b	169.4(3)
V1	N1b	C7b	C8b	-54.9(4)
V1	N1b	C7b	C12b	123.4(4)
V1	O2	C21	C22	179.7(4)
V1	O2	C24	C23	-154.4(4)
O1	C2	C1	N1	1.0(4)
O1	C2	C1	C6	-177.5(3)
O1	C2	C3	C4	176.9(4)
O1	C2	C3	C13	-3.2(5)
O1b	C2b	C1b	N1b	2.3(4)
O1b	C2b	C1b	C6b	-177.2(3)
O1b	C2b	C3b	C4b	178.2(4)
O1b	C2b	C3b	C13b	0.6(5)
N1	C1	C2	C3	178.7(3)
N1	C1	C6	C5	-177.6(4)
N1	C7	C8	C9	178.6(4)
N1	C7	C12	C11	-180.0(4)
N1b	C1b	C2b	C3b	-177.6(3)
N1b	C1b	C6b	C5b	178.8(4)
N1b	C7b	C8b	C9b	179.7(4)
N1b	C7b	C12b	C11b	-178.2(4)
C1	C2	C3	C4	-0.6(4)
C1	C2	C3	C13	179.4(4)
C1	C6	C5	C4	-1.1(5)
C1	C6	C5	C17	179.8(4)
C1b	C2b	C3b	C4b	-1.9(5)
C1b	C2b	C3b	C13b	-179.5(4)
C1b	C6b	C5b	C4b	-0.2(5)
C1b	C6b	C5b	C17b	178.2(4)
C2	C3	C4	C5	0.2(5)
C2	C3	C13	C14	58.0(4)
C2	C3	C13	C15	-63.1(4)
C2	C3	C13	C16	177.6(4)
C2b	C3b	C4b	C5b	-0.1(5)
C2b	C3b	C13b	C14b	62.8(4)
C2b	C3b	C13b	C15b	-59.3(4)
C2b	C3b	C13b	C16b	-179.0(4)
C3	C4	C5	C6	0.7(5)
C3	C4	C5	C17	179.9(4)
C3b	C4b	C5b	C6b	1.2(5)
C3b	C4b	C5b	C17b	-177.4(4)
C4	C5	C17	C18	-54.1(4)
C4	C5	C17	C19	65.8(4)
C4	C5	C17	C20	-174.5(4)
C4b	C5b	C17b	C18b	-55.2(5)
C4b	C5b	C17b	C19b	64.5(4)
C4b	C5b	C17b	C20b	-175.7(4)
C7	C8	C9	C10	1.7(6)
C7	C12	C11	C10	1.2(6)

Atom	Atom	Atom	Atom	Angle/°
C7b	C8b	C9b	C10b	-2.1(5)
C7b	C12b	C11b	C10b	-1.0(5)
C8	C9	C10	C11	-2.7(6)
C8b	C9b	C10b	C11b	1.3(6)
C9	C10	C11	C12	1.2(6)
C9b	C10b	C11b	C12b	0.3(6)
O1s	C1s	C2s	C3s	-21.8(6)
O1s	C4s	C3s	C2s	-17.8(6)
C1s	C2s	C3s	C4s	23.3(5)
O2	C21	C22	C23	-32.3(4)
O2	C24	C23	C22	-32.6(4)
C21	C22	C23	C24	39.8(5)

Table S6: Hydrogen Fractional Atomic Coordinates (×10[4]) and Equivalent Isotropic Displacement Parameters (Å[2]×10[3]) for **[(^Ph^ap)(^Ph^isq)V(THF)Cl]**. U_{eq} is defined as 1/3 of the trace of the orthogonalised U_{ij}.

Atom	x	y	z	U_{eq}
H4	3740(40)	4800(13)	8730(30)	20.8(13)
H4b	700(40)	2835(14)	1400(30)	26.7(15)
H6	5290(40)	3739(13)	7090(30)	20.2(13)
H6b	2050(40)	4158(14)	2290(30)	25.4(14)
H8	2620(40)	2912(15)	5380(30)	36.7(16)
H8b	1280(40)	4772(14)	4710(30)	28.7(15)
H9	4190(50)	2404(16)	4630(40)	42.2(17)
H9b	2720(40)	5430(15)	4580(30)	34.4(16)
H10	6530(50)	2665(16)	4330(40)	42.9(17)
H10b	4930(40)	5353(15)	3930(30)	35.9(16)
H11	7010(40)	3458(15)	4720(30)	38.1(16)
H11b	5650(50)	4639(15)	3310(30)	37.0(16)
H12	5480(40)	3958(15)	5430(30)	33.3(15)
H12b	4140(40)	3994(14)	3470(30)	27.0(15)
H14a	-260(40)	4493(12)	7272(15)	37.6(16)
H14b	-760(30)	4859(11)	8010(30)	37.6(16)
H14c	190(40)	4436(11)	8536(19)	37.6(16)
H14d	-1550(40)	2309(8)	3890(30)	37.8(17)
H14e	-960(40)	2803(11)	4243(17)	37.8(17)
H14f	-1880(30)	2745(14)	3090(20)	37.8(17)
H15a	330(30)	5415(11)	7130(30)	37.6(16)
H15b	1980(20)	5393(12)	6800(30)	37.6(16)
H15c	830(40)	5032(12)	6270(16)	37.6(16)
H15d	670(40)	2000(7)	4070(30)	42.3(17)
H15e	2102(19)	2272(16)	3710(30)	42.3(17)
H15f	1360(40)	2512(12)	4542(19)	42.3(17)
H16a	2580(20)	5356(12)	8540(30)	36.0(16)
H16b	840(20)	5376(11)	8750(30)	36.0(16)
H16c	1790(40)	4956(11)	9242(19)	36.0(16)
H16d	850(20)	2148(15)	2050(30)	42.0(18)
H16e	-830(30)	2357(14)	1860(20)	42.0(18)
H16f	-440(40)	1938(8)	2620(30)	42.0(18)
H18a	5690(30)	4957(11)	8860(30)	36.4(16)
H18b	7360(20)	4742(14)	9140(20)	36.4(16)
H18c	6680(40)	4897(14)	7976(14)	36.4(16)
H18d	2460(50)	3345(15)	-488(9)	45.3(18)
H18e	3392(18)	3274(16)	580(30)	45.3(18)
H18f	2000(40)	2952(6)	380(30)	45.3(18)
H19a	6540(20)	4143(15)	10040(30)	41.5(17)
H19b	5220(40)	3822(6)	9640(30)	41.5(17)
H19c	4890(30)	4355(12)	9890(30)	41.5(17)
H19d	-460(30)	3858(11)	470(30)	41.9(17)
H19e	310(40)	3699(14)	-554(10)	41.9(17)
H19f	-320(40)	3310(8)	290(40)	41.9(17)
H20a	7890(30)	4035(15)	8760(20)	36.8(16)
H20b	6750(40)	3649(4)	8300(30)	36.8(16)
H20c	7290(40)	4034(14)	7567(11)	36.8(16)
H20d	3134(19)	4112(15)	880(30)	39.1(17)
H20e	1550(30)	4322(10)	830(30)	39.1(17)
H20f	2260(40)	4102(15)	-203(7)	39.1(17)
H2sa	-1860(40)	3854(12)	1700(40)	64(2)
H1sa	-2180(40)	2949(10)	1340(40)	64(2)

Atom	x	y	z	U_{eq}
H1sb	-3060(50)	3356(16)	637(15)	64(2)
H2sb	-1820(40)	3478(16)	2590(20)	64(2)
H3sa	-4140(50)	3992(13)	1860(30)	62(2)
H3sb	-3590(60)	3894(16)	3060(15)	62(2)
H4sa	-5635(14)	3428(19)	2090(40)	75(2)
H24a	30(40)	2708(9)	5960(30)	39.5(17)
H24b	-1280(30)	3166(13)	5770(30)	39.5(17)
H21a	2003(9)	3378(16)	7450(40)	43.6(18)
H21b	500(40)	3638(8)	7520(30)	43.6(18)
H22a	880(40)	2654(9)	7690(40)	53(2)
H22b	50(50)	3008(17)	8473(13)	53(2)
H23a	-1650(40)	3150(11)	7320(40)	50.3(19)
H23b	-1360(40)	2572(6)	7130(40)	50.3(19)
H4sb	-4520(60)	3205(17)	2908(19)	75(2)

Crystal Data and Experimental for *trans*-[(^{Ph}isq)(^{Ph}ibq)V(O)Cl] (IIa)

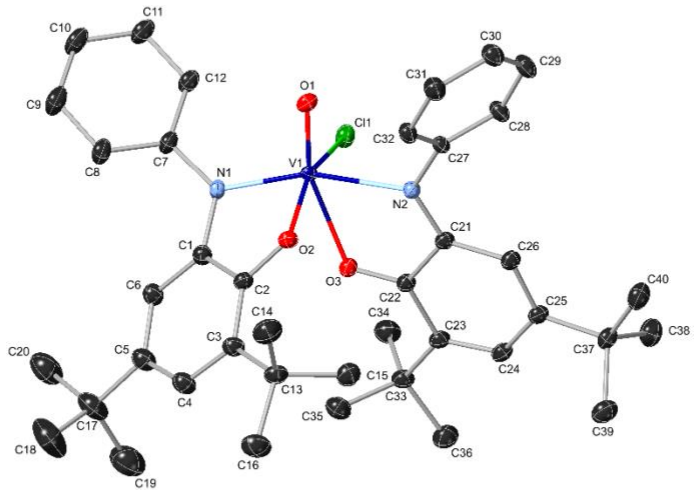

Experimental. A dark green needle-shaped crystal with dimensions 0.14 × 0.02 × 0.02 mm^3 was mounted on a loop with paratone. Data were collected using a XtaLAB Synergy, Dualflex, HyPix diffractometer operating at T = 100.00(10) K. Data were measured using w scans with Cu K_a radiation. The diffraction pattern was indexed and the total number of runs and images was based on the strategy calculation from the program CrysAlisPro 1.171.41.98a (Rigaku OD, 2021). The maximum resolution that was achieved was Q = 72.83˚ (0.81 Å). The unit cell was refined using CrysAlisPro 1.171.41.98a[7] on 2011 reflections, 7% of the observed reflections. Data reduction, scaling and absorption corrections were performed using CrysAlisPro 1.171.41.98a.[7] The final completeness is 99.67 % out to 72.83˚ in Q. A numerical absorption correction based on gaussian integration over a multifaceted crystal model was performed using CrysAlisPro 1.171.41.98.[7] An empirical absorption correction using spherical harmonics, implemented in SCALE3 ABSPACK scaling algorithm was also applied. The absorption coefficient m of this material is 2.682 mm^{-1} at this wavelength (l = 1.54184Å) and the minimum and maximum transmissions are 0.728 and 1.000. The structure was solved and the space group $P2_1/c$ (# 14) determined by the ShelXT[4] structure solution program using dual methods and refined by full matrix least squares minimisation on $\boldsymbol{F^2}$ using version of olex2.refine 1.5-alpha.[8] All atoms, even hydrogens, were refined anisotropically. Hydrogen atom positions were located and refined using the Hirshfeld model. Refinement was by using NoSpherA2, an implementation of non-spherical atom-form-factors.[6] NoSpherA2 implementation of HAR makes use of tailor-made aspherical atomic form factors calculated from a Hirshfeld-partitioned electron density (ED) not from spherical-atom form factors. The ED was calculated from a Gaussian basis set single determinant SCF wavefunction from DFT using selected functionals for a fragment of this crystal. This fragment was embedded in an electrostatic crystal field by employing cluster charges. The following options were used: SOFTWARE: 70.140.189.230_B692-needleHB692-needle.wfn PARTITIONING: NoSpherA2 INT ACCURACY: Normal METHOD: PBE BASIS SET: def2-SVP CHARGE: 0 MULTIPLICITY: 1 SOLVATION: Benzene DATE: 2022-06-04_10-37-16

There is a single molecule of the V-complex and a single toluene in the asymmetric unit. There is also ½-THF molecule for each V-complex, which is represented by the reported sum formula. In other words: Z is 4 and Z' is 1. The moiety formula is C40 H50 Cl N2 O3 V, C6 H6.

**Compound**	***trans*-[(Phisq)(Phibq)V(O)Cl]**
Formula	$C_{48}ClH_{60}N_2O_{3.5}V$
$D_{calc.}$/ g cm^{-3}	1.179
m/mm^{-1}	2.682
Formula Weight	807.417
Colour	dark green
Shape	needle-shaped
Size/mm^3	0.14×0.02×0.02
T/K	100.00(10)
Crystal System	monoclinic
Space Group	$P2_1/c$
a/Å	18.200(1)
b/Å	10.3892(7)
c/Å	24.4554(14)
a/°	90
b/°	100.291(5)
g/°	90
V/Å^3	4549.7(5)
Z	4
Z'	1
Wavelength/Å	1.54184
Radiation type	Cu K_a
Q_{min}/°	3.67
Q_{max}/°	72.83
Measured Refl's.	29456
Indep't Refl's	8707
Refl's I≥2 *s*(I)	5985
R_{int}	0.0970
Parameters	1007
Restraints	888
Largest Peak	1.2455
Deepest Hole	-0.5626
GooF	1.0259
wR_2 (all data)	0.1426
wR_2	0.1275
R_1 (all data)	0.0923
R_1	0.0581

Table S7: Fractional Atomic Coordinates (×10^4) and Equivalent Isotropic Displacement Parameters (Å^2×10^3) for ***trans*-[(^{Ph}isq)(^{Ph}ibq)V(O)Cl]**. U_{eq} is defined as 1/3 of the trace of the orthogonalised U_{ij}.

Atom	x	y	z	U_{eq}
V1	3645.9(6)	5508.7(11)	3212.4(4)	12.2(4)
Cl1	4310.9(4)	7004.7(7)	3832.0(3)	20.43(18)
O2	2788.1(10)	4868.5(19)	2695.6(7)	16.7(4)
O3	2892.9(10)	7284.4(19)	3122.3(7)	16.6(4)
O1	4324.7(11)	4583(2)	3172.2(8)	20.0(5)
N2	3856.6(12)	6668(2)	2505.3(9)	14.0(5)
N1	3132.6(12)	4648(2)	3746.9(9)	15.9(5)
C27	4387.9(14)	6216(3)	2185.6(10)	14.1(6)
C21	3495.1(14)	7741(3)	2377.6(10)	15.6(6)
C32	4298.4(16)	4969(3)	1973.3(11)	17.8(6)
C22	2958.7(14)	8067(3)	2758.8(10)	15.2(6)
C13	1272.0(16)	4951(3)	2041.2(12)	21.6(6)
C30	5415.2(16)	5237(3)	1584.2(13)	23.1(7)
C24	2647.9(16)	9999(3)	2226.6(11)	18.1(6)
C28	4991.3(15)	6979(3)	2107.6(12)	20.8(6)
C7	3439.8(16)	3849(3)	4193.2(11)	18.2(6)
C26	3538.4(15)	8550(3)	1902.8(11)	17.6(6)
C33	2057.8(16)	9696(3)	3089.7(11)	19.3(6)
C1	2372.5(15)	4694(3)	3542.3(11)	16.9(6)
C37	3141.6(16)	10538(3)	1336.6(12)	20.8(6)
C29	5506.8(16)	6483(3)	1800.3(13)	23.9(7)
C25	3123.9(15)	9640(3)	1826.3(11)	17.7(6)
C23	2548.9(15)	9292(3)	2672.5(11)	17.4(6)
C2	2207.2(15)	4822(3)	2952.7(11)	16.9(6)
C8	3052.0(18)	2799(3)	4369.2(12)	23.7(7)
C12	4192.1(17)	4049(3)	4442.5(12)	22.5(7)
C4	912.8(16)	4779(3)	3005.3(12)	23.5(7)
C31	4807.9(16)	4491(3)	1664.3(12)	22.6(6)
C3	1457.2(16)	4847(3)	2673.3(12)	19.9(6)
C9	3406.5(19)	1975(3)	4782.0(13)	29.2(7)
C34	2557.8(18)	9856(4)	3661.4(13)	26.1(7)
C11	4535.2(18)	3226(3)	4859.0(12)	27.3(7)
C5	1058.2(16)	4694(3)	3592.9(12)	23.4(7)
C6	1799.2(16)	4641(3)	3859.5(12)	20.3(6)
C10	4143.6(19)	2195(3)	5029.8(13)	28.6(7)
C15	1593.1(19)	6200(3)	1852.7(14)	27.4(7)
C35	1457.4(17)	8681(3)	3128.4(14)	26.7(7)
C38	3454(2)	11836(3)	1562.2(14)	28.2(7)
C36	1667.4(19)	10991(4)	2926.0(14)	29.4(7)
C17	405.5(18)	4686(4)	3910.4(14)	34.8(8)
C40	3633(2)	10021(3)	937.0(13)	27.9(7)
C39	2336.9(18)	10711(4)	1005.8(13)	28.7(7)
C14	1605(2)	3789(4)	1777.3(14)	30.2(8)
C6S	3524(2)	8163(4)	5025.9(14)	36.4(8)
C1S	2928(2)	7461(4)	4745.7(14)	36.6(8)
C16	427.1(19)	4941(4)	1828.0(15)	35.5(9)
C4S	2844(2)	8628(4)	5756.9(15)	40.0(9)
C5S	3489(2)	8728(4)	5533.5(14)	36.9(8)
C2S	2277(2)	7361(4)	4965.5(15)	43.1(9)
C3S	2235(2)	7933(4)	5475.7(16)	45.5(10)
C20	665(2)	4358(5)	4528.3(15)	44.9(10)
C18	-165(2)	3667(5)	3676.7(18)	52.6(12)
C19	31(2)	5995(5)	3869(2)	52.6(11)

Table S8: Anisotropic Displacement Parameters (×10[4]) for ***trans*-[(^{Ph}isq)(^{Ph}ibq)V(O)Cl]**. The anisotropic displacement factor exponent takes the form: $-2p^2[h^2a^{*2} \times U_{11} + ... +2hka^* \times b^* \times U_{12}]$

Atom	U_{11}	U_{22}	U_{33}	U_{23}	U_{13}	U_{12}
V1	9.4(6)	18.8(9)	8.2(6)	2.1(6)	1.6(5)	2.3(6)
Cl1	23.8(4)	18.3(4)	18.0(3)	-4.0(3)	0.2(3)	1.9(3)
O2	14.4(9)	18.1(11)	16.8(9)	1.4(8)	0.9(7)	1.1(8)
O3	17.8(10)	16.3(11)	16.8(9)	4.0(8)	6.4(7)	2.6(7)
O1	19.1(10)	18.2(11)	22.0(10)	4.0(9)	1.5(8)	4.2(9)
N2	14.5(11)	14.6(12)	13.8(10)	1.8(8)	5.1(7)	0.6(8)
N1	16.7(11)	15.0(13)	15.6(10)	-2.9(8)	2.1(7)	3.6(8)
C27	12.2(12)	15.1(14)	15.7(12)	0.2(8)	4.5(8)	0.2(8)
C21	15.8(13)	18.1(14)	13.2(12)	3.8(8)	3.0(8)	0.7(8)
C32	17.5(14)	14.5(14)	22.7(13)	-1.9(9)	7.1(9)	-0.6(9)
H32	45(9)	30(8)	64(17)	-21(4)	40(6)	-24(5)
C22	16.1(12)	15.0(13)	14.8(12)	2.8(8)	4.1(8)	-0.1(8)
C13	17.3(13)	25.6(15)	19.6(12)	1.6(9)	-2.4(8)	0.7(9)
C30	20.3(14)	19.8(15)	31.0(15)	0.1(9)	9.4(10)	-1.9(10)
H30	41(9)	30(10)	75(18)	-11(4)	39(6)	-21(6)
C24	20.3(14)	14.7(15)	18.8(12)	4.4(10)	2.4(8)	0.9(9)
H24	89(19)	33(6)	54(13)	40(5)	52(8)	26(4)
C28	16.1(13)	18.5(15)	29.7(14)	-1.6(9)	9.7(9)	-0.4(11)
H28	80(17)	52(8)	190(30)	-46(6)	106(12)	-73(8)
C7	24.2(13)	14.4(14)	14.8(12)	-1.0(9)	-0.1(8)	2.4(9)
C26	18.9(14)	18.6(14)	15.5(12)	3.2(9)	4.2(9)	2.2(9)
H26	50(14)	26(12)	28(8)	25(6)	25(5)	19(5)
C33	17.9(13)	20.2(15)	20.9(12)	6.6(9)	6.2(8)	-1.4(9)
C1	17.1(12)	17.5(15)	15.6(12)	-2.5(9)	1.0(7)	-0.4(9)
C37	24.1(14)	17.5(14)	20.3(12)	1.7(9)	2.5(8)	4.5(9)
C29	19.5(15)	19.8(15)	35.8(16)	-1.8(10)	14.7(10)	-2.8(10)
H29	61(10)	49(9)	110(20)	-35(4)	69(8)	-43(7)
C25	17.4(13)	18.1(14)	17.3(12)	3.5(9)	2.3(8)	1.9(8)
C23	16.5(13)	17.9(14)	18.2(12)	5.7(9)	4.1(8)	0.5(8)
C2	15.0(12)	17.4(15)	17.2(12)	1.1(9)	0.4(7)	2.1(9)
C8	29.7(16)	18.9(15)	21.4(14)	-3.9(10)	1.3(10)	4.8(10)
H8	38(5)	40(14)	57(16)	-17(4)	-15(4)	33(8)
C12	25.0(14)	18.1(15)	22.1(13)	-0.8(10)	-2.6(9)	5.0(10)
H12	49(11)	54(10)	110(20)	-30(5)	-47(8)	60(8)
C4	17.3(14)	30.7(19)	21.9(13)	-3.3(11)	2.0(8)	-1.6(10)
H4	18(3)	130(40)	28(9)	-2(2)	0.4(18)	4(5)
C31	24.0(14)	18.5(15)	27.5(14)	0.2(10)	10.4(9)	-2.9(11)
H31	63(14)	27(4)	90(20)	-18(3)	60(9)	-25(4)
C3	15.5(13)	24.7(17)	18.1(12)	-0.5(9)	-0.8(7)	0.2(9)
C9	40.7(16)	20.8(16)	23.5(14)	-3.7(11)	-1.1(10)	6.2(11)
H9	66(10)	80(15)	130(30)	-40(5)	-43(8)	82(10)
C34	24.4(16)	33(2)	21.2(14)	4.0(13)	6.3(10)	-2.9(11)
H34a	30(8)	35(4)	24(9)	6(3)	4(3)	-3(3)
H34b	26(6)	34(5)	37(10)	4(3)	12(3)	-5(3)
H34c	35(7)	47(9)	31(5)	4(3)	15(3)	-6(3)
C11	31.4(16)	22.4(16)	24.6(14)	1.1(11)	-4.2(10)	7.1(10)
H11	39(4)	59(18)	70(20)	-15(4)	-22(4)	42(10)
C5	19.1(13)	29.5(18)	21.7(13)	-2.3(10)	3.8(7)	-3.3(10)
C6	19.8(13)	24.3(17)	16.9(13)	-1.5(9)	4.1(8)	0.8(11)
H6	31(9)	70(30)	18(2)	-6(5)	3.3(13)	3.2(17)
C10	38.3(16)	21.8(17)	21.9(14)	-0.9(10)	-4.7(10)	7.1(11)
H10	56(10)	51(13)	62(18)	-10(5)	-21(6)	39(7)
C15	25.1(17)	29.4(18)	26.3(16)	0.1(12)	0.6(12)	5.3(12)
H15a	35(9)	33(5)	37(8)	2(3)	5(4)	2(3)
H15b	26(3)	32(10)	33(9)	-0.2(15)	0.6(16)	5(4)
H15c	28(9)	39(10)	27(3)	1(4)	1.4(16)	6.2(16)

Atom	U_{11}	U_{22}	U_{33}	U_{23}	U_{13}	U_{12}
C35	21.0(16)	30.6(18)	30.2(17)	2.4(12)	8.9(11)	-1.9(13)
H35a	30(6)	34(10)	34(4)	0(3)	4(2)	-1(2)
H35b	28(8)	32(4)	34(9)	5(2)	11(4)	0(2)
H35c	30(6)	34(9)	38(6)	3(3)	16(3)	-2(3)
C38	35.4(19)	19.9(16)	29.3(17)	-3.0(12)	5.7(13)	0.8(11)
H38a	40(8)	23(10)	36(6)	-6(4)	10(3)	-3(3)
H38b	38(3)	39(10)	39(7)	-2(2)	3(2)	1(3)
H38c	41(10)	24(7)	34(5)	-1(3)	9(3)	4(3)
C36	30.2(18)	28.9(18)	31.9(17)	12.7(12)	13.4(13)	5.1(12)
H36a	36(6)	32(5)	31(9)	9(3)	14(3)	5(3)
H36b	39(7)	41(10)	35(3)	11(3)	9(2)	7(2)
H36c	35(7)	28(9)	35(6)	16(3)	17(3)	8(3)
C17	22.5(14)	52(2)	32.4(15)	-6.1(10)	10.5(9)	-8.2(11)
C40	36.6(19)	22.9(18)	25.8(16)	4.3(13)	10.1(11)	5.0(12)
H40a	38(3)	35(10)	34(8)	6(2)	8.5(18)	4(4)
H40b	40(8)	25(4)	29(8)	4(2)	10(3)	4(2)
H40c	43(10)	31(7)	31(5)	5(3)	12(3)	10(3)
C39	28.6(16)	31(2)	25.1(15)	6.5(12)	1.1(10)	5.0(14)
H39a	38(10)	34(4)	37(10)	3(2)	2(4)	1(2)
H39b	31(7)	40(9)	31(7)	6(3)	4(3)	0(3)
H39c	51(10)	33(8)	28(5)	7(4)	7(3)	6(3)
C14	32.4(18)	31.8(19)	23.6(16)	4.1(13)	-2.5(12)	-3.0(12)
H14a	33(3)	43(10)	38(9)	4.4(17)	-3.9(17)	-1(4)
H14b	46(9)	34(4)	35(9)	2(2)	5(4)	-2(2)
H14c	31(9)	40(10)	25(3)	5(4)	-1.8(16)	-2.5(16)
C6S	49.5(19)	32.5(19)	28.5(15)	7.5(13)	10.6(11)	2.6(11)
H6S	77(8)	190(40)	92(17)	-55(9)	52(6)	-91(14)
C1S	53.3(19)	34(2)	23.8(15)	4.9(13)	9.3(10)	4.6(12)
H1S	71(13)	70(20)	38(8)	-14(7)	23(4)	-18(7)
C16	21.6(15)	51(3)	30.7(18)	-0.2(12)	-4.6(11)	4.0(17)
H16a	33(9)	55(5)	45(10)	-5(3)	-3(4)	8(3)
H16b	28(8)	51(5)	36(9)	2(3)	1(4)	5(3)
H16c	16(9)	53(10)	31(3)	2(4)	-4.5(15)	4(2)
C4S	50.9(19)	39(2)	31.7(17)	-0.3(13)	13.1(11)	-5.0(13)
H4S	71(13)	100(30)	48(8)	-26(8)	29(4)	-35(8)
C5S	48.0(19)	36(2)	26.9(15)	3.7(14)	7.4(11)	1.7(12)
H5S	66(9)	110(30)	60(13)	-31(7)	25(5)	-45(9)
C2S	52(2)	43(2)	35.7(17)	-0.1(14)	12.6(11)	-5.1(13)
H2S	61(7)	90(30)	54(13)	-19(6)	20(5)	-29(9)
C3S	50(2)	49(2)	40.4(18)	-4.8(15)	16.0(12)	-9.6(14)
H3S	97(11)	270(50)	111(18)	-103(12)	73(7)	-125(15)
C20	33(2)	72(3)	32.9(16)	-6.8(19)	13.2(12)	-4.0(14)
H20a	34(7)	73(5)	44(11)	-6(3)	16(4)	-1(3)
H20b	37(6)	76(7)	44(9)	-8(3)	11(3)	-8(3)
H20c	36(4)	89(13)	40(8)	-6(3)	17(3)	-2(4)
C18	43(2)	76(3)	43(2)	-26.5(17)	19.3(15)	-18.0(19)
H18a	41(9)	81(11)	45(3)	-31(4)	18(2)	-16(3)
H18b	45(9)	75(5)	42(10)	-26(3)	25(4)	-15(3)
H18c	47(6)	89(13)	53(9)	-26(4)	26(4)	-17(4)
C19	37(2)	61(3)	65(3)	5.0(14)	21.1(18)	-2.1(16)
H19a	36(7)	63(7)	68(10)	4(3)	25(4)	-7(3)
H19b	39(9)	62(10)	65(4)	5(4)	21(2)	-2(2)
H19c	39(5)	75(12)	70(10)	3(3)	25(4)	-3(4)

Table S9: Bond Lengths in Å for ***trans*-[(^{Ph}isq)(^{Ph}ibq)V(O)Cl]**.

Atom	Atom	Length/Å
V1	Cl1	2.3497(13)
V1	O2	1.942(2)
V1	O3	2.285(2)
V1	O1	1.582(2)
V1	N2	2.197(2)
V1	N1	1.954(3)
O2	C2	1.324(3)
O3	C22	1.226(3)
N2	C27	1.427(3)
N2	C21	1.304(4)
N1	C7	1.405(3)
N1	C1	1.385(3)
C27	C32	1.394(4)
C27	C28	1.395(4)
C21	C22	1.504(4)
C21	C26	1.447(4)
C32	C31	1.388(4)
C22	C23	1.471(4)
C13	C3	1.526(4)
C13	C15	1.528(5)
C13	C14	1.543(5)
C13	C16	1.533(4)
C30	C29	1.397(4)
C30	C31	1.392(4)
C24	C25	1.467(4)
C24	C23	1.353(4)
C28	C29	1.401(4)
C7	C8	1.408(4)
C7	C12	1.411(4)

Atom	Atom	Length/Å
C26	C25	1.355(4)
C33	C23	1.530(4)
C33	C34	1.535(4)
C33	C35	1.533(4)
C33	C36	1.541(4)
C1	C2	1.426(4)
C1	C6	1.408(4)
C37	C25	1.523(4)
C37	C38	1.528(4)
C37	C40	1.534(4)
C37	C39	1.552(4)
C2	C3	1.413(4)
C8	C9	1.391(4)
C12	C11	1.390(4)
C4	C3	1.391(4)
C4	C5	1.416(4)
C9	C10	1.389(5)
C11	C10	1.391(5)
C5	C6	1.390(4)
C5	C17	1.531(4)
C17	C20	1.538(5)
C17	C18	1.521(5)
C17	C19	1.517(6)
C6S	C1S	1.383(5)
C6S	C5S	1.385(5)
C1S	C2S	1.390(5)
C4S	C5S	1.383(5)
C4S	C3S	1.397(5)
C2S	C3S	1.396(5)

Table S10: Bond Angles in ° for ***trans*-[(^{Ph}isq)(^{Ph}ibq)V(O)Cl]**.

Atom	Atom	Atom	Angle/°
O2	V1	Cl1	155.20(8)
O3	V1	Cl1	76.07(6)
O3	V1	O2	79.35(8)
O1	V1	Cl1	97.13(8)
O1	V1	O2	107.28(11)
O1	V1	O3	161.08(11)
N2	V1	Cl1	90.16(7)
N2	V1	O2	84.75(9)
N2	V1	O3	70.58(8)
N2	V1	O1	92.07(10)
N1	V1	Cl1	97.13(8)
N1	V1	O2	81.59(9)
N1	V1	O3	94.81(9)
N1	V1	O1	103.61(12)
N1	V1	N2	161.67(10)
C2	O2	V1	108.72(16)
C22	O3	V1	118.08(18)
C27	N2	V1	118.79(18)
C21	N2	V1	120.38(18)
C21	N2	C27	120.8(2)
C7	N1	V1	128.20(19)
C1	N1	V1	107.97(17)
C1	N1	C7	122.3(2)
C32	C27	N2	117.7(2)

Atom	Atom	Atom	Angle/°
C28	C27	N2	121.0(3)
C28	C27	C32	121.3(3)
C22	C21	N2	113.5(2)
C26	C21	N2	126.2(3)
C26	C21	C22	120.1(2)
C31	C32	C27	119.3(3)
C21	C22	O3	117.3(3)
C23	C22	O3	124.4(3)
C23	C22	C21	118.3(2)
C15	C13	C3	109.8(3)
C14	C13	C3	109.6(3)
C14	C13	C15	109.7(3)
C16	C13	C3	111.8(3)
C16	C13	C15	108.5(3)
C16	C13	C14	107.3(3)
C31	C30	C29	120.5(3)
C23	C24	C25	125.4(3)
C29	C28	C27	118.9(3)
C8	C7	N1	122.9(3)
C12	C7	N1	118.0(3)
C12	C7	C8	118.8(3)
C25	C26	C21	119.1(3)
C34	C33	C23	108.4(2)
C35	C33	C23	111.1(2)

Atom	Atom	Atom	Angle/°
C35	C33	C34	109.4(3)
C36	C33	C23	111.3(2)
C36	C33	C34	108.2(3)
C36	C33	C35	108.4(3)
C2	C1	N1	112.7(2)
C6	C1	N1	126.2(2)
C6	C1	C2	121.2(2)
C38	C37	C25	108.4(2)
C40	C37	C25	112.5(3)
C40	C37	C38	108.5(3)
C39	C37	C25	109.3(2)
C39	C37	C38	109.9(3)
C39	C37	C40	108.1(2)
C28	C29	C30	119.9(3)
C26	C25	C24	120.1(3)
C37	C25	C24	117.9(3)
C37	C25	C26	122.1(3)
C24	C23	C22	116.6(3)
C33	C23	C22	118.9(2)
C33	C23	C24	124.5(3)
C1	C2	O2	116.2(2)
C3	C2	O2	123.6(2)
C3	C2	C1	120.1(3)
C9	C8	C7	120.4(3)
C11	C12	C7	120.0(3)

Atom	Atom	Atom	Angle/°
C5	C4	C3	124.9(3)
C30	C31	C32	120.1(3)
C2	C3	C13	120.7(3)
C4	C3	C13	122.9(2)
C4	C3	C2	116.4(3)
C10	C9	C8	120.1(3)
C10	C11	C12	120.4(3)
C6	C5	C4	117.9(3)
C17	C5	C4	119.6(3)
C17	C5	C6	122.5(3)
C5	C6	C1	119.5(2)
C11	C10	C9	120.1(3)
C20	C17	C5	111.7(3)
C18	C17	C5	110.7(3)
C18	C17	C20	106.5(3)
C19	C17	C5	109.9(3)
C19	C17	C20	108.6(3)
C19	C17	C18	109.3(3)
C5S	C6S	C1S	120.6(4)
C2S	C1S	C6S	119.7(3)
C3S	C4S	C5S	119.9(3)
C4S	C5S	C6S	120.0(4)
C3S	C2S	C1S	120.0(4)
C2S	C3S	C4S	119.7(4)

Table S11: Torsion Angles in ° for ***trans*-[(^{Ph}isq)(^{Ph}ibq)V(O)Cl]**.

Atom	Atom	Atom	Atom	Angle/°
V1	O2	C2	C1	-25.37(19)
V1	O2	C2	C3	156.87(19)
V1	O3	C22	C21	4.09(18)
V1	O3	C22	C23	-176.01(17)
V1	N2	C27	C32	53.2(2)
V1	N2	C27	C28	-124.33(19)
V1	N2	C21	C22	0.77(19)
V1	N2	C21	C26	-175.34(18)
V1	N1	C7	C8	-146.3(3)
V1	N1	C7	C12	28.1(3)
V1	N1	C1	C2	24.5(2)
V1	N1	C1	C6	-154.67(19)
O2	C2	C1	N1	0.4(3)
O2	C2	C1	C6	179.6(3)
O2	C2	C3	C13	-0.9(4)
O2	C2	C3	C4	179.5(3)
O3	C22	C21	N2	-3.3(3)
O3	C22	C21	C26	173.1(2)
O3	C22	C23	C24	-175.3(3)
O3	C22	C23	C33	5.8(3)
N2	C27	C32	C31	-179.4(2)
N2	C27	C28	C29	178.7(3)
N2	C21	C22	C23	176.8(2)
N2	C21	C26	C25	179.8(3)
N1	C7	C8	C9	174.4(3)
N1	C7	C12	C11	-175.5(3)
N1	C1	C2	C3	178.2(2)
N1	C1	C6	C5	-179.8(3)
C27	C32	C31	C30	2.2(3)

Atom	Atom	Atom	Atom	Angle/°
C27	C28	C29	C30	-0.8(3)
C21	C22	C23	C24	4.6(3)
C21	C22	C23	C33	-174.3(2)
C21	C26	C25	C24	1.0(3)
C21	C26	C25	C37	179.9(2)
C32	C31	C30	C29	-1.7(3)
C22	C23	C24	C25	0.3(3)
C22	C23	C33	C34	61.9(3)
C22	C23	C33	C35	-58.3(3)
C22	C23	C33	C36	-179.3(3)
C13	C3	C2	C1	-178.5(3)
C13	C3	C4	C5	-179.3(3)
C24	C25	C37	C38	62.5(3)
C24	C25	C37	C40	-177.5(3)
C24	C25	C37	C39	-57.3(3)
C24	C23	C33	C34	-116.9(3)
C24	C23	C33	C35	122.9(3)
C24	C23	C33	C36	2.0(3)
C7	C8	C9	C10	1.0(4)
C7	C12	C11	C10	0.6(4)
C26	C25	C37	C38	-116.5(3)
C26	C25	C37	C40	3.5(3)
C26	C25	C37	C39	123.7(3)
C1	C2	C3	C4	1.8(3)
C1	C6	C5	C4	1.0(3)
C1	C6	C5	C17	-178.0(3)
C2	C3	C4	C5	0.3(4)
C8	C9	C10	C11	-1.2(4)
C12	C11	C10	C9	0.4(4)
C4	C5	C17	C20	170.3(4)
C4	C5	C17	C18	51.7(4)
C4	C5	C17	C19	-69.1(4)
C6S	C1S	C2S	C3S	-2.0(4)
C6S	C5S	C4S	C3S	1.4(4)
C1S	C2S	C3S	C4S	1.4(5)

Table S12: Hydrogen Fractional Atomic Coordinates (×10⁴) and Equivalent Isotropic Displacement Parameters ($Å^2 \times 10^3$) for ***trans*-[(PhIsq)(PhIbq)V(O)Cl]**. U_{eq} is defined as 1/3 of the trace of the orthogonalised U_{ij}.

Atom	x	y	z	U_{eq}
H32	3847(18)	4420(30)	2065(14)	42(8)
H30	5777(18)	4820(30)	1349(15)	45(9)
H24	2400(20)	10860(30)	2186(14)	54(10)
H28	5090(20)	8020(40)	2300(20)	97(16)
H26	3890(17)	8230(30)	1611(12)	33(8)
H29	5957(19)	7080(40)	1751(17)	68(11)
H8	2512(18)	2630(30)	4181(14)	48(10)
H12	4450(20)	4930(40)	4353(18)	78(13)
H4	313(18)	4810(40)	2797(13)	58(12)
H31	4752(19)	3470(30)	1514(16)	53(10)
H9	3120(20)	1180(40)	4890(20)	100(15)
H34a	2859(16)	9000(30)	3815(12)	30(4)
H34b	2944(17)	10640(30)	3637(13)	32(5)
H34c	2251(17)	10070(30)	3951(12)	37(4)
H11	5160(20)	3420(40)	5048(16)	60(12)
H6	1950(16)	4590(30)	4307(12)	40(9)

Atom	x	y	z	U_{eq}
H10	4419(19)	1510(40)	5362(15)	60(11)
H15a	1406(17)	7030(30)	2043(13)	35(5)
H15b	2200(17)	6200(30)	1935(12)	31(4)
H15c	1500(16)	6310(30)	1410(12)	32(4)
H35a	1082(16)	8590(30)	2747(12)	33(4)
H35b	1710(16)	7760(30)	3242(13)	31(4)
H35c	1111(16)	8950(30)	3409(12)	33(4)
H38a	3142(18)	12270(30)	1893(14)	33(5)
H38b	3982(18)	11750(30)	1771(13)	39(4)
H38c	3466(17)	12450(30)	1220(13)	33(4)
H36a	2061(17)	11750(30)	2948(12)	32(4)
H36b	1318(17)	11030(30)	2534(13)	38(5)
H36c	1333(16)	11210(30)	3243(12)	31(4)
H40a	4189(18)	9910(30)	1121(13)	35(4)
H40b	3419(16)	9120(30)	780(12)	31(4)
H40c	3596(17)	10650(30)	578(13)	34(5)
H39a	2139(18)	9660(30)	855(14)	37(5)
H39b	1982(16)	11130(30)	1253(12)	34(5)
H39c	2376(18)	11350(30)	635(13)	37(5)
H14a	2201(18)	3790(30)	1910(13)	40(4)
H14b	1437(18)	2880(30)	1907(13)	39(5)
H14c	1494(16)	3850(30)	1337(12)	33(4)
H6S	4080(30)	8210(60)	4840(20)	120(20)
H1S	3040(20)	7030(40)	4321(15)	59(11)
H16a	183(18)	4030(40)	1934(14)	45(5)
H16b	169(17)	5790(40)	1962(13)	39(5)
H16c	331(16)	5060(30)	1382(14)	35(4)
H4S	2780(20)	9050(50)	6195(16)	69(13)
H5S	3960(20)	9310(50)	5774(17)	78(14)
H2S	1750(20)	6790(40)	4723(17)	67(13)
H3S	1710(30)	7770(70)	5640(20)	150(20)
H20a	1028(19)	3530(40)	4586(14)	50(5)
H20b	1050(19)	5040(40)	4738(15)	52(5)
H20c	159(18)	4370(40)	4695(14)	54(5)
H18a	-385(19)	3890(40)	3236(15)	54(5)
H18b	120(20)	2750(40)	3697(15)	52(5)
H18c	-650(20)	3710(40)	3930(15)	61(6)
H19a	444(19)	6750(40)	4041(16)	54(5)
H19b	-144(19)	6280(40)	3448(17)	54(5)
H19c	-490(20)	5920(40)	4063(16)	59(6)

Table S13: Solvent masking (PLATON/SQUEEZE) information for ***trans*-[(^{Ph}isq)(^{Ph}ibq)V(O)Cl]**.

No	x	y	z	V	e	Content
1	0.000	0.000	0.500	230.5	42.0	1C4H8O
2	0.000	0.500	0.000	230.5	42.0	1C4H8O

Crystal data and experimental for *cis*-[(^{Ph}isq)(^{Ph}ibq)V(O)Cl] (IIb)

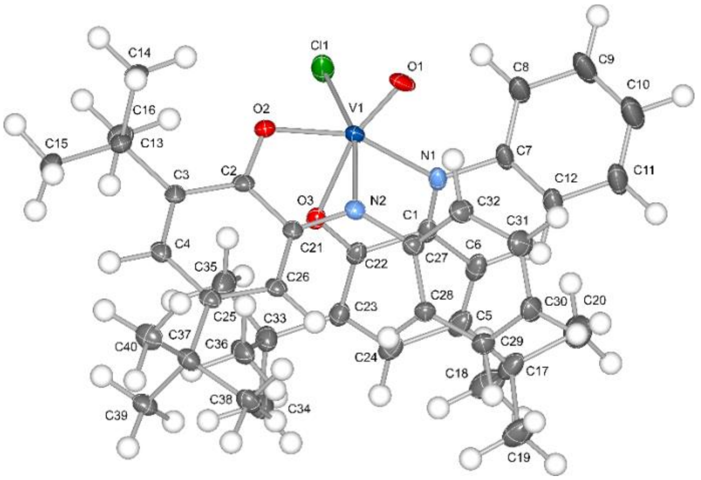

Experimental. A black prism-shaped-shaped crystal with dimensions 0.08 × 0.06 × 0.05 mm^3 was mounted on a loop with paratone. Data were collected using a XtaLAB Synergy, Dualflex, HyPix diffractometer operating at T = 100.00(10) K. Data were measured using *w* scans with Cu K_a radiation. The diffraction pattern was indexed and the total number of runs and images was based on the strategy calculation from the program CrysAlisPro system.[7] The maximum resolution that was achieved was Q = 65.082° (0.85 Å). The unit cell was refined using CrysAlisPro 1.171.43.103a[7] on 6665 reflections, 12% of the observed reflections. Data reduction, scaling and absorption corrections were performed using CrysAlisPro 1.171.43.a.[7] The final completeness is 98.90 % out to 65.082° in Q. A numerical absorption correction based on gaussian integration over a multifaceted crystal model was performed using CrysAlisPro 1.171.42.74a.[7] An empirical absorption correction using spherical harmonics, implemented in SCALE3 ABSPACK scaling algorithm was also applied. The absorption coefficient *m* of this material is 2.968 mm^{-1} at this wavelength (l = 1.54184Å) and the minimum and maximum transmissions are 0.846 and 0.902. The structure was solved and the space group $P2_1/n$ (# 14) determined by the ShelXT[4] structure solution program using using dual methods and refined by full matrix least squares

**Compound**	***cis*-[(^{Ph}isq)(^{Ph}ibq)V(O)Cl]**
Formula	$C_{86}H_{106}Cl_2N_4O_6V_2$
$D_{calc.}$/ g cm^{-3}	1.207
m/mm^{-1}	2.968
Formula Weight	1464.52
Colour	black
Shape	prism-shaped
Size/mm^3	0.08×0.06×0.05
T/K	100.00(10)
Crystal System	monoclinic
Space Group	$P2_1/n$
a/Å	20.9479(11)
b/Å	15.5814(7)
c/Å	25.0491(11)
a/°	90
b/°	99.67(1)
g/°	90
V/Å^3	8059.9(7)
Z	4
Z'	1
Wavelength/Å	1.54184
Radiation type	Cu K_a
Q_{min}/°	3.011
Q_{max}/°	65.082
Measured Refl's.	53557
Indep't Refl's	13612
Refl's I≥2 *s*(I)	8009
R_{int}	0.1466
Parameters	974
Restraints	1506
Largest Peak	0.750
Deepest Hole	-0.854
GooF	1.028
wR_2 (all data)	0.2316
wR_2	0.1994
R_1 (all data)	0.1422
R_1	0.0841

minimisation on F^2 using version 2018/3 of ShelXL.[5] All non-hydrogen atoms were refined anisotropically. Hydrogen atom positions were calculated geometrically and refined using the riding model. There are two [V(O)L2] complexes and one disordered benzene molecule in the asymmetric unit, which is represented by the reported sum formula. In other words: Z is 4 and Z' is 1. The moiety formula is 2[C40H50ClN3O3V], 1[C6H6].

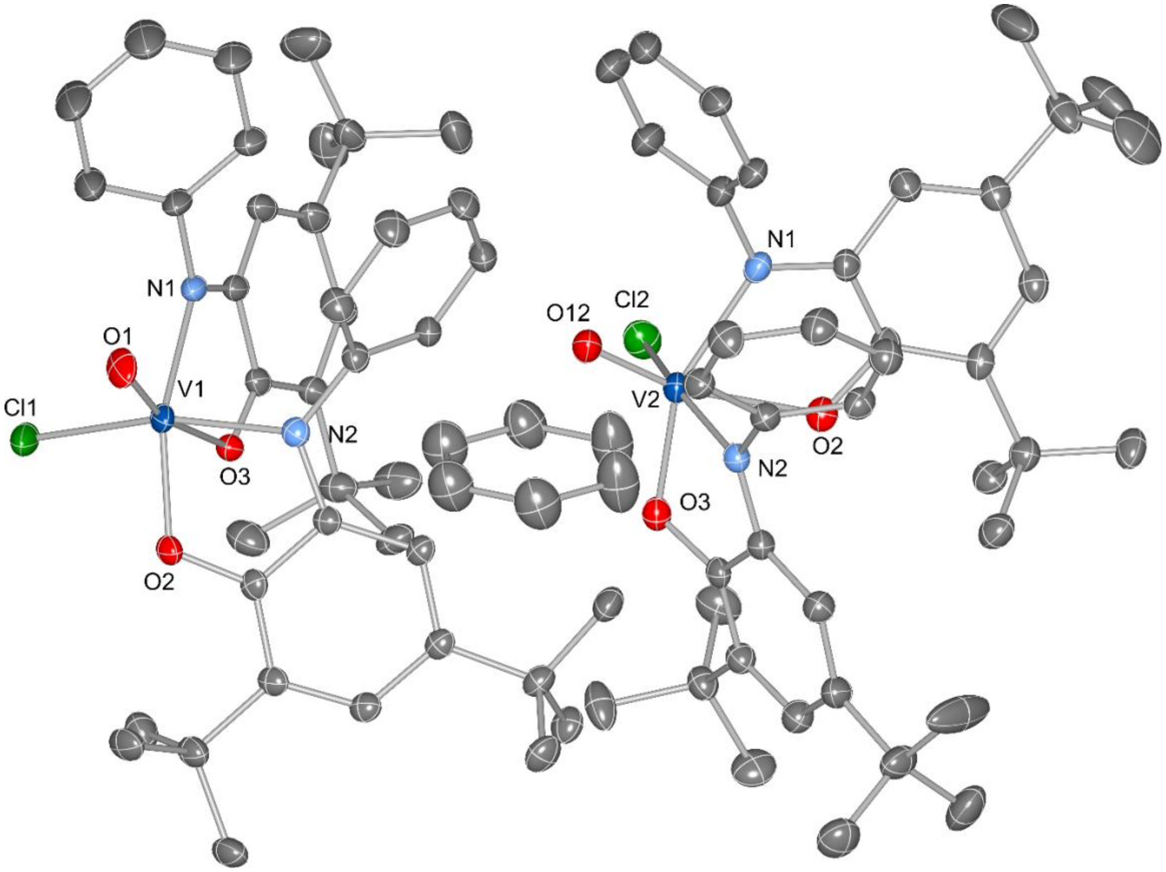

Table S14: Fractional Atomic Coordinates (×10^4) and Equivalent Isotropic Displacement Parameters (Å^2×10^3) for ***cis*-[(^{Ph}isq)(^{Ph}ibq)V(O)Cl]**. U_{eq} is defined as 1/3 of the trace of the orthogonalised U_{ij}.

Atom	x	y	z	U_{eq}
V1	8007.8(4)	6099.5(6)	6514.4(4)	34.9(2)
Cl1	7661.1(8)	5926.4(11)	5584.5(6)	57.7(4)
O1	7335.7(18)	5956(3)	6725.4(17)	54.0(11)
O3	8875.5(17)	6675(2)	6266.2(13)	32.8(8)
O2	8474.6(17)	5030(2)	6531.0(14)	34.8(8)
N2	8603(2)	6193(3)	7233.0(16)	31.6(9)
N1	7881(2)	7463(3)	6492.1(17)	39.0(10)
C25	10125(2)	5011(3)	7694(2)	31.8(10)
C21	9116(2)	5649(3)	7277(2)	29.7(10)
C1	8340(3)	7950(3)	6360(2)	36.8(11)
C22	8918(3)	7468(3)	6249.1(19)	34.6(10)
C2	9026(2)	5006(3)	6873(2)	31.2(10)
C23	9486(3)	7933(3)	6158(2)	37.0(11)

Atom	x	y	z	U_{eq}
C4	10037(2)	4388(3)	7272(2)	33.9(10)
C26	9663(2)	5642(3)	7696(2)	30.2(10)
C3	9500(2)	4356(3)	6866(2)	32.4(10)
C28	9006(3)	7501(3)	7727(2)	33.9(11)
C27	8553(3)	6831(3)	7628.5(19)	32.2(10)
C37	10728(3)	4971(3)	8131(2)	37.0(11)
C6	8347(3)	8878(3)	6315(2)	44.5(12)
C13	9406(3)	3653(3)	6423(2)	41.0(11)
C29	8927(3)	8134(4)	8097(2)	41.6(12)
C39	11323(3)	5133(4)	7864(2)	42.9(13)
C7	7331(3)	7857(4)	6660(2)	48.8(14)
C5	8872(3)	9287(4)	6240(2)	48.6(13)
C32	8021(3)	6819(4)	7889(2)	41.9(12)
C31	7942(3)	7459(4)	8258(2)	50.2(14)
C38	10717(3)	5644(4)	8571(2)	45.3(13)
C14	8782(3)	3137(3)	6470(3)	49.2(15)
C12	7412(3)	8447(4)	7085(2)	48.7(14)
C15	9971(3)	3002(3)	6506(3)	50.2(15)
C36	10336(3)	6870(4)	6549(3)	49.2(14)
C40	10781(3)	4080(4)	8398(2)	46.8(14)
C16	9345(3)	4062(4)	5873(2)	50.9(14)
C17	8916(3)	10264(4)	6238(2)	54.2(14)
C33	10090(3)	7439(4)	6054(2)	46.1(12)
C19	9398(4)	10533(5)	6746(3)	72(2)
C34	10634(4)	8050(4)	5978(4)	69(2)
C8	6723(3)	7595(5)	6427(3)	79(2)
C18	9179(4)	10601(4)	5734(3)	72(2)
C10	6269(4)	8578(6)	6997(4)	98(3)
C20	8266(4)	10697(4)	6224(4)	89(3)
C9	6182(4)	7969(6)	6595(4)	107(3)
C30	8392(3)	8112(4)	8362(2)	46.8(13)
C24	9455(3)	8806(3)	6174(2)	43.9(12)
C35	9900(4)	6902(4)	5539(3)	59.4(17)
C11	6871(3)	8822(5)	7244(3)	70(2)
V12	11094.0(5)	8698.6(6)	7966.3(3)	35.1(2)
Cl12	11214.5(8)	9652.2(9)	7265.0(6)	54.1(4)
O32	11590.3(18)	7869(2)	7639.5(13)	37.3(8)
O22	12086.9(18)	9152(2)	8366.4(13)	39.4(8)
O12	10337.0(19)	8537(2)	7800.8(16)	46.9(10)
N22	11327(2)	7867(2)	8583.1(16)	32.0(9)
N12	10962(2)	9716(3)	8506.4(17)	38.4(10)
C222	12009(2)	7442(3)	7998.5(19)	31.5(10)
C272	11051(3)	7875(3)	9069(2)	35.8(11)
C212	11850(3)	7389(3)	8531.2(19)	33.0(10)
C232	12569(3)	7042(3)	7875(2)	35.8(11)
C1A	12663(2)	9977(3)	9108.3(18)	35.7(10)
C282	10402(3)	7658(3)	9041(2)	40.5(12)
C322	11410(3)	8153(3)	9557(2)	39.1(12)
C42	12587(3)	10643(4)	9452(2)	40.3(12)
C242	12914(3)	6532(3)	8277(2)	41.4(12)
C302	10477(3)	7975(4)	9992(2)	50.2(14)
C82	10141(3)	9777(4)	9099(2)	44.4(12)
C92	9525(3)	9981(4)	9192(3)	50.5(14)
C312	11119(3)	8217(4)	10012(2)	44.9(13)
C292	10120(3)	7700(4)	9505(2)	48.5(13)
C62	11434(3)	10822(4)	9146(2)	43.3(12)
C152	13546(3)	9711(4)	8567(2)	43.3(13)

Atom	x	y	z	U_{eq}
C172	12010(3)	11890(4)	9817(3)	56.5(15)
C372	13157(3)	5846(4)	9217(2)	51.9(14)
C382	13008(4)	4909(4)	9070(3)	65.5(18)
C192	12472(5)	12543(5)	9653(4)	85(3)
C392	13878(4)	5998(5)	9210(3)	77(2)
C202	11345(4)	12313(5)	9769(4)	96(3)
C182	12231(6)	11578(7)	10401(3)	102(3)
C252	12745(3)	6430(4)	8802(2)	42.4(12)
C52	11980(3)	11103(4)	9451(2)	40.7(11)
C1B	12086.8(17)	9770(3)	8763.7(16)	37.2(11)
C262	12225(3)	6862(3)	8925(2)	39.4(11)
C332	12772(3)	7178(3)	7324(2)	39.8(11)
C132	13330(3)	9545(4)	9108(2)	42.5(12)
C1C	11471.7(19)	10093(3)	8799(2)	35.7(11)
C72	10328(3)	9951(4)	8604(2)	43.6(12)
C162	13844(3)	9896(4)	9563(2)	52.1(15)
C142	13268(3)	8580(4)	9193(3)	51.0(15)
C122	9891(3)	10305(4)	8186(3)	58.8(16)
C102	9086(3)	10352(4)	8785(3)	61.6(16)
C112	9269(3)	10519(5)	8294(3)	65.2(17)
C352	12773(4)	8130(4)	7180(3)	60.9(17)
C362	13472(3)	6867(4)	7323(3)	57.3(16)
C342	12317(3)	6673(5)	6904(2)	59.6(18)
C402	13044(6)	6045(7)	9791(3)	118(4)
C1S_2	8804(2)	4403(2)	9866.7(13)	147(4)
C2S_2	8418(2)	5134(2)	9858.9(13)	149(5)
C3S_2	8387(2)	5721(2)	9435.9(14)	144(5)
C4S_2	8740(2)	5578(2)	9020.4(13)	140(6)
C5S_2	9125(2)	4848(2)	9028.1(14)	148(6)
C6S_2	9157(2)	4261(2)	9451.1(14)	157(6)
C1S_1	8399(2)	4518(2)	9332.7(14)	147(4)
C6S_1	8495(2)	5197(2)	8992.8(12)	146(5)
C5S_1	8967(2)	5811(2)	9165.5(14)	133(5)
C4S_1	9344(2)	5747(2)	9678.1(14)	167(7)
C3S_1	9247.6(19)	5069(2)	10017.9(12)	168(6)
C2S_1	8775(2)	4454(2)	9845.3(14)	147(4)

Table S15: Anisotropic Displacement Parameters (×10^4) for ***cis*-[(^{Ph}isq)(^{Ph}ibq)V(O)Cl]**. The anisotropic displacement factor exponent takes the form: $-2p^2[h^2a^{*2} \times U_{11} + ... +2hka^* \times b^* \times U_{12}]$

Atom	U_{11}	U_{22}	U_{33}	U_{23}	U_{13}	U_{12}
V1	21.0(5)	39.8(5)	41.7(5)	-12.8(4)	-1.0(4)	-0.7(4)
Cl1	44.7(9)	74.2(10)	47.7(8)	-25.3(7)	-11.3(6)	19.6(7)
O1	21(2)	74(3)	67(3)	-25(2)	8.9(18)	-12.5(18)
O3	34(2)	30.9(15)	31.8(17)	-3.9(14)	1.1(15)	5.3(14)
O2	27.1(18)	32.4(17)	43.5(19)	-10.3(15)	1.7(14)	-4.6(14)
N2	27(2)	33(2)	35(2)	-2.2(16)	6.2(17)	-3.0(16)
N1	31(2)	45(2)	37(2)	-15.1(19)	-4.8(18)	12.4(16)
C25	26(2)	31(2)	38(2)	5.4(18)	5.4(19)	-3.7(18)
C21	26(2)	28(2)	36(2)	-1.0(18)	7.1(18)	-3.7(17)
C1	37(3)	39(2)	30(2)	-9(2)	-5(2)	12.6(18)
C22	38(3)	34(2)	28(2)	-6(2)	-3(2)	5.4(18)
C2	26(2)	29(2)	38(2)	-0.7(18)	4.2(18)	-8.0(17)
C23	41(3)	33(2)	36(3)	-4(2)	3(2)	5.4(18)

Atom	U_{11}	U_{22}	U_{33}	U_{23}	U_{13}	U_{12}
C4	27(2)	32(2)	44(2)	0.8(19)	8.9(19)	-2(2)
C26	24(2)	30(2)	36(2)	4(2)	5.6(18)	-4.3(18)
C3	27(2)	27(2)	45(2)	-2.9(19)	9.6(19)	-6.3(18)
C28	30(3)	36(2)	34(2)	-5.8(19)	-1(2)	0.2(19)
C27	31(2)	37(2)	29(2)	-3.0(18)	4.3(19)	-1.9(19)
C37	28(2)	40(2)	42(2)	6.1(19)	5.1(19)	2(2)
C6	53(3)	39(2)	36(3)	-8(2)	-7(2)	16(2)
C13	33(3)	34(3)	56(3)	-12(2)	6(2)	-5.1(19)
C29	35(3)	46(3)	41(3)	-12(2)	0(2)	-1(2)
C39	28(3)	54(3)	47(3)	0(3)	7(2)	-5(2)
C7	31(3)	61(3)	50(3)	-21(2)	-4(2)	20(2)
C5	60(3)	35(2)	46(3)	-4(2)	-8(3)	10(2)
C32	40(3)	47(3)	41(3)	-4(2)	13(2)	-5(2)
C31	48(3)	62(3)	43(3)	-11(2)	14(3)	-1(2)
C38	31(3)	57(3)	45(3)	-2(2)	-2(2)	5(3)
C14	37(3)	30(3)	80(4)	-14(3)	7(3)	-10(2)
C12	39(3)	56(3)	48(3)	-14(2)	0(2)	17(3)
C15	42(3)	34(3)	74(4)	-17(3)	8(3)	-1(2)
C36	32(3)	40(3)	74(4)	7(3)	4(3)	6(2)
C40	43(4)	47(3)	47(3)	16(2)	-1(3)	5(3)
C16	50(4)	54(3)	48(3)	-14(2)	7(3)	-6(3)
C17	70(4)	33(2)	52(3)	-8(2)	-13(3)	7(2)
C33	43(3)	36(3)	61(3)	-2(2)	15(3)	3(2)
C19	86(5)	59(4)	60(4)	-18(3)	-19(4)	0(4)
C34	49(4)	54(4)	109(6)	-2(4)	27(4)	-4(3)
C8	36(3)	96(5)	96(5)	-58(4)	-13(3)	21(3)
C18	96(6)	44(4)	69(4)	5(3)	-7(4)	-12(4)
C10	44(3)	132(6)	115(6)	-64(5)	2(4)	28(4)
C20	77(5)	42(4)	144(8)	-7(4)	2(5)	14(3)
C9	34(4)	150(7)	127(6)	-79(5)	-15(4)	33(4)
C30	44(3)	56(3)	40(3)	-18(3)	6(2)	5(2)
C24	53(3)	33(2)	41(3)	-1(2)	-3(3)	2(2)
C35	61(4)	53(4)	70(4)	-16(3)	26(3)	-2(3)
C11	46(3)	87(5)	75(4)	-34(4)	3(3)	26(3)
V12	32.4(5)	36.4(5)	33.9(5)	-2.8(4)	-1.9(4)	-1.1(4)
Cl12	69.0(11)	45.5(8)	46.1(8)	5.8(6)	4.7(7)	-0.8(7)
O32	38(2)	37.5(19)	34.6(17)	0.2(15)	0.6(15)	-3.8(15)
O22	38(2)	43.2(19)	37.3(18)	1.7(15)	6.0(16)	-3.0(16)
O12	41(2)	42(2)	52(2)	-6.5(17)	-7.9(18)	1.6(17)
N22	30(2)	32(2)	33.5(19)	-4.7(17)	2.5(16)	-2.4(16)
N12	29(2)	38(2)	44(2)	0.1(19)	-4.2(17)	1.8(17)
C222	28(2)	31(2)	34(2)	-5.2(18)	1.0(17)	-6.4(18)
C272	33(2)	37(3)	37(2)	-1(2)	4.7(18)	3(2)
C212	31(3)	34(2)	33(2)	-3.1(19)	2.4(19)	-1.2(18)
C232	32(3)	38(3)	38(2)	-4.9(19)	7.4(19)	-2.4(19)
C1A	32(2)	45(3)	30(2)	4.4(19)	3.9(19)	-8(2)
C282	33(2)	39(3)	49(3)	-5(2)	6(2)	0(2)
C322	36(3)	44(3)	36(2)	3(2)	3.9(19)	3(2)
C42	35(3)	49(3)	37(3)	-2(2)	7(2)	-10(2)
C242	37(3)	44(3)	43(2)	-2(2)	5(2)	0(2)
C302	43(3)	61(4)	49(3)	0(3)	13(2)	1(3)
C82	27(3)	45(3)	57(3)	-7(2)	-4(2)	6(2)
C92	30(3)	50(3)	70(3)	-21(3)	3(2)	4(2)
C312	44(3)	50(3)	41(3)	0(2)	8(2)	-2(2)
C292	38(3)	54(3)	55(3)	-2(3)	15(2)	-9(3)
C62	40(3)	45(3)	45(3)	-9(2)	7(2)	-3(2)
C152	35(3)	47(3)	49(3)	-3(2)	11(2)	-1(2)
C172	51(3)	65(3)	58(3)	-27(3)	21(3)	-15(3)
C372	52(3)	58(3)	43(3)	5(2)	0(3)	19(3)

Atom	U_{11}	U_{22}	U_{33}	U_{23}	U_{13}	U_{12}
C382	65(5)	55(3)	73(4)	13(3)	2(4)	9(3)
C192	98(6)	64(4)	107(6)	-45(4)	56(5)	-31(4)
C392	49(3)	79(5)	94(5)	15(4)	-17(4)	7(3)
C202	65(4)	78(5)	146(8)	-66(5)	24(5)	-3(4)
C182	130(9)	128(8)	50(3)	-32(4)	23(4)	2(6)
C252	44(3)	43(3)	38(2)	-5(2)	0(2)	6(2)
C52	39(3)	46(3)	37(3)	-5(2)	10(2)	-8(2)
C1B	35(2)	39(3)	38(2)	3.4(19)	4.4(19)	-8(2)
C262	39(3)	39(3)	39(3)	0(2)	4(2)	1(2)
C332	38(3)	45(3)	38(2)	-7(2)	9(2)	-5(2)
C132	35(3)	45(3)	46(3)	-2(2)	3(2)	-3(2)
C1C	35(2)	38(2)	32(2)	-1.8(19)	-0.1(19)	-1.8(19)
C72	32(2)	43(3)	51(3)	-10(2)	-7(2)	5(2)
C162	31(3)	73(4)	51(3)	-8(3)	2(2)	-5(3)
C142	37(3)	50(3)	64(4)	3(3)	2(3)	2(3)
C122	50(3)	63(4)	57(3)	-10(3)	-11(3)	20(3)
C102	40(3)	67(4)	71(3)	-27(3)	-8(3)	14(3)
C112	47(3)	73(4)	67(3)	-13(3)	-14(3)	24(3)
C352	74(5)	47(3)	65(4)	8(3)	23(4)	-6(3)
C362	51(3)	70(4)	57(4)	2(3)	27(3)	7(3)
C342	50(4)	89(5)	42(3)	-23(3)	16(3)	-26(3)
C402	154(10)	156(9)	43(4)	16(5)	13(5)	111(8)
C1S_2	179(9)	131(7)	128(7)	10(6)	16(7)	-26(7)
C2S_2	178(11)	133(9)	135(9)	5(7)	22(8)	-23(8)
C3S_2	155(9)	138(8)	136(8)	8(7)	22(7)	-10(7)
C4S_2	167(12)	122(11)	129(10)	4(9)	20(9)	-7(10)
C5S_2	176(14)	133(11)	136(11)	20(9)	32(10)	4(10)
C6S_2	188(13)	143(11)	138(10)	27(9)	27(9)	-4(10)
C1S_1	179(9)	131(7)	128(7)	10(6)	16(7)	-26(7)
C6S_1	164(11)	134(9)	136(9)	15(7)	15(8)	-24(8)
C5S_1	164(11)	131(9)	103(8)	0(8)	18(9)	-18(9)
C4S_1	213(14)	147(12)	125(11)	29(10)	-21(11)	-66(11)
C3S_1	219(13)	142(10)	127(10)	38(9)	-22(10)	-72(10)
C2S_1	182(10)	131(8)	126(8)	18(7)	24(8)	-29(8)

Table S16: Bond Lengths in Å for ***cis*-[(^{Ph}isq)(^{Ph}ibq)V(O)Cl]**.

Atom	Atom	Length/Å
V1	Cl1	2.3362(16)
V1	O1	1.600(4)
V1	O3	2.208(4)
V1	O2	1.929(3)
V1	N2	2.016(4)
V1	N1	2.141(4)
O3	C22	1.240(6)
O2	C2	1.318(6)
N2	C21	1.359(6)
N2	C27	1.420(6)
N1	C1	1.310(7)
N1	C7	1.429(7)
C25	C4	1.423(7)
C25	C26	1.380(7)
C25	C37	1.529(7)
C21	C2	1.414(7)
C21	C26	1.418(7)
C1	C22	1.491(7)

Atom	Atom	Length/Å
C1	C6	1.450(8)
C22	C23	1.443(8)
C2	C3	1.420(7)
C23	C33	1.540(8)
C23	C24	1.363(7)
C4	H4	1.0770
C4	C3	1.385(7)
C26	H26	1.0770
C3	C13	1.547(7)
C28	H28	1.0770
C28	C27	1.403(7)
C28	C29	1.384(7)
C27	C32	1.383(8)
C37	C39	1.532(8)
C37	C38	1.524(8)
C37	C40	1.537(7)
C6	H6	1.0770
C6	C5	1.313(9)

Atom	Atom	Length/Å
C13	C14	1.556(8)
C13	C15	1.547(8)
C13	C16	1.504(9)
C29	H29	1.0770
C29	C30	1.396(8)
C39	H39A	1.0880
C39	H39B	1.0880
C39	H39C	1.0880
C7	C12	1.396(8)
C7	C8	1.371(9)
C5	C17	1.525(8)
C5	C24	1.464(9)
C32	H32	1.0770
C32	C31	1.389(8)
C31	H31	1.0770
C31	C30	1.383(9)
C38	H38A	1.0880
C38	H38B	1.0880
C38	H38C	1.0880
C14	H14A	1.0880
C14	H14B	1.0880
C14	H14C	1.0880
C12	H12	1.0770
C12	C11	1.390(9)
C15	H15A	1.0880
C15	H15B	1.0880
C15	H15C	1.0880
C36	H36A	1.0880
C36	H36B	1.0880
C36	H36C	1.0880
C36	C33	1.540(9)
C40	H40A	1.0880
C40	H40B	1.0880
C40	H40C	1.0880
C16	H16A	1.0880
C16	H16B	1.0880
C16	H16C	1.0880
C17	C19	1.543(9)
C17	C18	1.551(10)
C17	C20	1.514(11)
C33	C34	1.523(9)
C33	C35	1.534(9)
C19	H19A	1.0880
C19	H19B	1.0880
C19	H19C	1.0880
C34	H34A	1.0880
C34	H34B	1.0880
C34	H34C	1.0880
C8	H8	1.0770
C8	C9	1.401(10)
C18	H18A	1.0880
C18	H18B	1.0880
C18	H18C	1.0880
C10	H10	1.0770
C10	C9	1.373(11)
C10	C11	1.362(11)
C20	H20A	1.0880
C20	H20B	1.0880
C20	H20C	1.0880

Atom	Atom	Length/Å
C9	H9	1.0770
C30	H30	1.0770
C24	H24	1.0770
C35	H35A	1.0880
C35	H35B	1.0880
C35	H35C	1.0880
C11	H11	1.0770
V12	Cl12	2.3466(17)
V12	O32	1.926(4)
V12	O22	2.265(4)
V12	O12	1.590(4)
V12	N22	2.012(4)
V12	N12	2.133(4)
O32	C222	1.325(6)
O22	C1B	1.385(2)
N22	C272	1.432(7)
N22	C212	1.350(7)
N12	C1C	1.327(6)
N12	C72	1.436(7)
C222	C212	1.431(7)
C222	C232	1.407(7)
C272	C282	1.391(8)
C272	C322	1.392(8)
C212	C262	1.416(7)
C232	C242	1.388(8)
C232	C332	1.525(7)
C1A	C42	1.374(8)
C1A	C1B	1.399(2)
C1A	C132	1.550(8)
C282	H282	1.0770
C282	C292	1.391(8)
C322	H322	1.0770
C322	C312	1.382(8)
C42	H42	1.0770
C42	C52	1.459(8)
C242	H242	1.0770
C242	C252	1.427(8)
C302	H302	1.0770
C302	C312	1.389(9)
C302	C292	1.387(9)
C82	H82	1.0770
C82	C92	1.386(8)
C82	C72	1.389(8)
C92	H92	1.0770
C92	C102	1.381(9)
C312	H312	1.0770
C292	H292	1.0770
C62	H62	1.0770
C62	C52	1.338(8)
C62	C1C	1.440(7)
C152	H15D	1.0880
C152	H15E	1.0880
C152	H15F	1.0880
C152	C132	1.521(8)
C172	C192	1.508(10)
C172	C202	1.527(11)
C172	C182	1.537(11)
C172	C52	1.527(8)
C372	C382	1.525(9)

Atom	Atom	Length/Å
C372	C392	1.533(10)
C372	C252	1.533(8)
C372	C402	1.526(10)
C382	H38D	1.0880
C382	H38E	1.0880
C382	H38F	1.0880
C192	H19D	1.0880
C192	H19E	1.0880
C192	H19F	1.0880
C392	H39D	1.0880
C392	H39E	1.0880
C392	H39F	1.0880
C202	H20D	1.0880
C202	H20E	1.0880
C202	H20F	1.0880
C182	H18D	1.0880
C182	H18E	1.0880
C182	H18F	1.0880
C252	C262	1.359(8)
C1B	C1C	1.400(2)
C262	H262	1.0770
C332	C352	1.528(8)
C332	C362	1.544(9)
C332	C342	1.516(8)
C132	C162	1.532(8)
C132	C142	1.526(8)
C72	C122	1.384(8)
C162	H16D	1.0880
C162	H16E	1.0880
C162	H16F	1.0880
C142	H14D	1.0880
C142	H14E	1.0880
C142	H14F	1.0880
C122	H122	1.0770
C122	C112	1.415(10)
C102	H102	1.0770
C102	C112	1.373(10)

Atom	Atom	Length/Å
C112	H112	1.0770
C352	H35D	1.0880
C352	H35E	1.0880
C352	H35F	1.0880
C362	H36D	1.0880
C362	H36E	1.0880
C362	H36F	1.0880
C342	H34D	1.0880
C342	H34E	1.0880
C342	H34F	1.0880
C402	H40D	1.0880
C402	H40E	1.0880
C402	H40F	1.0880
C1S_2	H1S_2	1.0770
C1S_2	C2S_2	1.3930(9)
C1S_2	C6S_2	1.3930(9)
C2S_2	H2S_2	1.0770
C2S_2	C3S_2	1.3930(9)
C3S_2	H3S_2	1.0770
C3S_2	C4S_2	1.3930(9)
C4S_2	H4S_2	1.0770
C4S_2	C5S_2	1.3930(9)
C5S_2	H5S_2	1.0770
C5S_2	C6S_2	1.3930(9)
C6S_2	H6S_2	1.0770
C1S_1	H1S_1	1.0770
C1S_1	C6S_1	1.3930(9)
C1S_1	C2S_1	1.3930(9)
C6S_1	H6S_1	1.0770
C6S_1	C5S_1	1.3930(9)
C5S_1	H5S_1	1.0770
C5S_1	C4S_1	1.3930(9)
C4S_1	H4S_1	1.0770
C4S_1	C3S_1	1.3930(9)
C3S_1	H3S_1	1.0770
C3S_1	C2S_1	1.3930(9)
C2S_1	H2S_1	1.0770

Table S17: Bond Angles in ° for ***cis*-[(^{Ph}isq)(^{Ph}ibq)V(O)Cl]**.

Atom	Atom	Atom	Angle/°
O1	V1	Cl1	99.77(16)
O1	V1	O3	164.01(18)
O1	V1	O2	110.04(19)
O1	V1	N2	99.31(19)
O1	V1	N1	92.0(2)
O3	V1	Cl1	84.20(10)
O2	V1	Cl1	89.62(11)
O2	V1	O3	85.34(13)
O2	V1	N2	78.91(15)
O2	V1	N1	156.65(17)
N2	V1	Cl1	160.20(13)
N2	V1	O3	78.88(14)
N2	V1	N1	90.40(16)
N1	V1	Cl1	94.14(12)
N1	V1	O3	72.16(15)

Atom	Atom	Atom	Angle/°
C22	O3	V1	118.8(3)
C2	O2	V1	115.1(3)
C21	N2	V1	112.9(3)
C21	N2	C27	122.1(4)
C27	N2	V1	124.5(3)
C1	N1	V1	119.3(3)
C1	N1	C7	119.2(5)
C7	N1	V1	121.4(4)
C4	C25	C37	119.5(4)
C26	C25	C4	119.1(5)
C26	C25	C37	121.4(5)
N2	C21	C2	112.0(4)
N2	C21	C26	126.5(4)
C2	C21	C26	121.3(4)
N1	C1	C22	114.1(5)

Atom	Atom	Atom	Angle/°
N1	C1	C6	127.9(5)
C6	C1	C22	118.0(5)
O3	C22	C1	115.4(5)
O3	C22	C23	124.9(5)
C23	C22	C1	119.6(5)
O2	C2	C21	116.3(4)
O2	C2	C3	123.4(4)
C21	C2	C3	120.3(4)
C22	C23	C33	119.8(4)
C24	C23	C22	116.8(5)
C24	C23	C33	123.3(5)
C25	C4	H4	118.1
C3	C4	C25	123.9(5)
C3	C4	H4	118.1
C25	C26	C21	118.8(4)
C25	C26	H26	120.6
C21	C26	H26	120.6
C2	C3	C13	120.6(5)
C4	C3	C2	116.6(4)
C4	C3	C13	122.8(4)
C27	C28	H28	120.2
C29	C28	H28	120.2
C29	C28	C27	119.7(5)
C28	C27	N2	121.0(5)
C32	C27	N2	118.6(5)
C32	C27	C28	120.2(5)
C25	C37	C39	108.4(4)
C25	C37	C40	109.9(4)
C39	C37	C40	109.6(5)
C38	C37	C25	112.2(4)
C38	C37	C39	108.4(5)
C38	C37	C40	108.4(5)
C1	C6	H6	119.5
C5	C6	C1	121.0(5)
C5	C6	H6	119.5
C3	C13	C14	108.7(5)
C15	C13	C3	111.5(4)
C15	C13	C14	106.6(4)
C16	C13	C3	109.7(4)
C16	C13	C14	110.1(5)
C16	C13	C15	110.2(5)
C28	C29	H29	120.1
C28	C29	C30	119.8(5)
C30	C29	H29	120.1
C37	C39	H39A	109.5
C37	C39	H39B	109.5
C37	C39	H39C	109.5
H39A	C39	H39B	109.5
H39A	C39	H39C	109.5
H39B	C39	H39C	109.5
C12	C7	N1	120.5(5)
C8	C7	N1	119.0(5)
C8	C7	C12	120.3(5)
C6	C5	C17	122.5(6)
C6	C5	C24	120.1(5)
C24	C5	C17	117.3(6)
C27	C32	H32	120.0
C27	C32	C31	120.0(5)
C31	C32	H32	120.0

Atom	Atom	Atom	Angle/°
C32	C31	H31	120.0
C30	C31	C32	120.0(6)
C30	C31	H31	120.0
C37	C38	H38A	109.5
C37	C38	H38B	109.5
C37	C38	H38C	109.5
H38A	C38	H38B	109.5
H38A	C38	H38C	109.5
H38B	C38	H38C	109.5
C13	C14	H14A	109.5
C13	C14	H14B	109.5
C13	C14	H14C	109.5
H14A	C14	H14B	109.5
H14A	C14	H14C	109.5
H14B	C14	H14C	109.5
C7	C12	H12	120.2
C11	C12	C7	119.7(6)
C11	C12	H12	120.2
C13	C15	H15A	109.5
C13	C15	H15B	109.5
C13	C15	H15C	109.5
H15A	C15	H15B	109.5
H15A	C15	H15C	109.5
H15B	C15	H15C	109.5
H36A	C36	H36B	109.5
H36A	C36	H36C	109.5
H36B	C36	H36C	109.5
C33	C36	H36A	109.5
C33	C36	H36B	109.5
C33	C36	H36C	109.5
C37	C40	H40A	109.5
C37	C40	H40B	109.5
C37	C40	H40C	109.5
H40A	C40	H40B	109.5
H40A	C40	H40C	109.5
H40B	C40	H40C	109.5
C13	C16	H16A	109.5
C13	C16	H16B	109.5
C13	C16	H16C	109.5
H16A	C16	H16B	109.5
H16A	C16	H16C	109.5
H16B	C16	H16C	109.5
C5	C17	C19	107.4(5)
C5	C17	C18	111.7(5)
C19	C17	C18	107.9(6)
C20	C17	C5	112.9(6)
C20	C17	C19	111.1(6)
C20	C17	C18	105.7(6)
C23	C33	C36	109.0(5)
C34	C33	C23	111.2(5)
C34	C33	C36	107.7(6)
C34	C33	C35	109.2(5)
C35	C33	C23	108.0(5)
C35	C33	C36	111.6(5)
C17	C19	H19A	109.5
C17	C19	H19B	109.5
C17	C19	H19C	109.5
H19A	C19	H19B	109.5
H19A	C19	H19C	109.5

Atom	Atom	Atom	Angle/°
H19B	C19	H19C	109.5
C33	C34	H34A	109.5
C33	C34	H34B	109.5
C33	C34	H34C	109.5
H34A	C34	H34B	109.5
H34A	C34	H34C	109.5
H34B	C34	H34C	109.5
C7	C8	H8	120.4
C7	C8	C9	119.3(6)
C9	C8	H8	120.4
C17	C18	H18A	109.5
C17	C18	H18B	109.5
C17	C18	H18C	109.5
H18A	C18	H18B	109.5
H18A	C18	H18C	109.5
H18B	C18	H18C	109.5
C9	C10	H10	119.2
C11	C10	H10	119.2
C11	C10	C9	121.5(7)
C17	C20	H20A	109.5
C17	C20	H20B	109.5
C17	C20	H20C	109.5
H20A	C20	H20B	109.5
H20A	C20	H20C	109.5
H20B	C20	H20C	109.5
C8	C9	H9	120.2
C10	C9	C8	119.6(7)
C10	C9	H9	120.2
C29	C30	H30	119.8
C31	C30	C29	120.3(5)
C31	C30	H30	119.8
C23	C24	C5	124.0(6)
C23	C24	H24	118.0
C5	C24	H24	118.0
C33	C35	H35A	109.5
C33	C35	H35B	109.5
C33	C35	H35C	109.5
H35A	C35	H35B	109.5
H35A	C35	H35C	109.5
H35B	C35	H35C	109.5
C12	C11	H11	120.3
C10	C11	C12	119.4(6)
C10	C11	H11	120.3
O32	V12	Cl12	88.48(11)
O32	V12	O22	82.91(14)
O32	V12	N22	79.39(15)
O32	V12	N12	154.96(17)
O22	V12	Cl12	85.84(10)
O12	V12	Cl12	97.64(15)
O12	V12	O32	111.64(18)
O12	V12	O22	165.04(17)
O12	V12	N22	101.57(19)
O12	V12	N12	93.02(18)
N22	V12	Cl12	160.01(14)
N22	V12	O22	77.01(15)
N22	V12	N12	91.83(16)
N12	V12	Cl12	92.64(13)
N12	V12	O22	72.24(14)
C222	O32	V12	113.0(3)

Atom	Atom	Atom	Angle/°
C1B	O22	V12	115.1(3)
C272	N22	V12	124.6(3)
C212	N22	V12	112.2(3)
C212	N22	C272	122.4(4)
C1C	N12	V12	120.0(3)
C1C	N12	C72	118.4(4)
C72	N12	V12	121.3(3)
O32	C222	C212	115.5(4)
O32	C222	C232	123.7(4)
C232	C222	C212	120.8(5)
C282	C272	N22	119.0(5)
C282	C272	C322	120.0(5)
C322	C272	N22	120.9(5)
N22	C212	C222	112.2(4)
N22	C212	C262	128.2(5)
C262	C212	C222	119.6(5)
C222	C232	C332	120.4(5)
C242	C232	C222	116.5(5)
C242	C232	C332	123.1(5)
C42	C1A	C1B	112.4(4)
C42	C1A	C132	121.9(4)
C1B	C1A	C132	125.7(4)
C272	C282	H282	120.2
C272	C282	C292	119.6(5)
C292	C282	H282	120.2
C272	C322	H322	120.1
C312	C322	C272	119.8(5)
C312	C322	H322	120.1
C1A	C42	H42	117.8
C1A	C42	C52	124.3(5)
C52	C42	H42	117.8
C232	C242	H242	118.2
C232	C242	C252	123.5(5)
C252	C242	H242	118.2
C312	C302	H302	120.3
C292	C302	H302	120.3
C292	C302	C312	119.4(6)
C92	C82	H82	119.5
C92	C82	C72	121.0(6)
C72	C82	H82	119.5
C82	C92	H92	120.1
C102	C92	C82	119.7(7)
C102	C92	H92	120.1
C322	C312	C302	120.6(5)
C322	C312	H312	119.7
C302	C312	H312	119.7
C282	C292	H292	119.8
C302	C292	C282	120.4(6)
C302	C292	H292	119.8
C52	C62	H62	120.8
C52	C62	C1C	118.4(5)
C1C	C62	H62	120.8
H15D	C152	H15E	109.5
H15D	C152	H15F	109.5
H15E	C152	H15F	109.5
C132	C152	H15D	109.5
C132	C152	H15E	109.5
C132	C152	H15F	109.5
C192	C172	C202	107.9(7)

Atom	Atom	Atom	Angle/°
C192	C172	C182	111.6(7)
C192	C172	C52	110.0(5)
C202	C172	C182	109.7(7)
C202	C172	C52	110.7(5)
C52	C172	C182	107.0(6)
C382	C372	C392	107.9(6)
C382	C372	C252	109.6(5)
C382	C372	C402	111.4(7)
C252	C372	C392	110.1(6)
C402	C372	C392	106.7(7)
C402	C372	C252	111.0(5)
C372	C382	H38D	109.5
C372	C382	H38E	109.5
C372	C382	H38F	109.5
H38D	C382	H38E	109.5
H38D	C382	H38F	109.5
H38E	C382	H38F	109.5
C172	C192	H19D	109.5
C172	C192	H19E	109.5
C172	C192	H19F	109.5
H19D	C192	H19E	109.5
H19D	C192	H19F	109.5
H19E	C192	H19F	109.5
C372	C392	H39D	109.5
C372	C392	H39E	109.5
C372	C392	H39F	109.5
H39D	C392	H39E	109.5
H39D	C392	H39F	109.5
H39E	C392	H39F	109.5
C172	C202	H20D	109.5
C172	C202	H20E	109.5
C172	C202	H20F	109.5
H20D	C202	H20E	109.5
H20D	C202	H20F	109.5
H20E	C202	H20F	109.5
C172	C182	H18D	109.5
C172	C182	H18E	109.5
C172	C182	H18F	109.5
H18D	C182	H18E	109.5
H18D	C182	H18F	109.5
H18E	C182	H18F	109.5
C242	C252	C372	119.4(5)
C262	C252	C242	119.1(5)
C262	C252	C372	121.5(5)
C42	C52	C172	116.5(5)
C62	C52	C42	120.0(5)
C62	C52	C172	123.5(5)
O22	C1B	C1A	120.0(4)
O22	C1B	C1C	114.0(3)
C1A	C1B	C1C	125.9(4)
C212	C262	H262	119.9
C252	C262	C212	120.1(5)
C252	C262	H262	119.9
C232	C332	C352	111.2(5)
C232	C332	C362	111.6(5)
C352	C332	C362	105.4(5)
C342	C332	C232	108.5(5)
C342	C332	C352	111.4(5)
C342	C332	C362	108.8(5)

Atom	Atom	Atom	Angle/°
C152	C132	C1A	109.2(4)
C152	C132	C162	109.0(5)
C152	C132	C142	109.6(5)
C162	C132	C1A	111.4(5)
C142	C132	C1A	109.3(5)
C142	C132	C162	108.3(5)
N12	C1C	C62	124.2(4)
N12	C1C	C1B	118.1(4)
C1B	C1C	C62	117.7(4)
C82	C72	N12	120.6(5)
C122	C72	N12	119.0(6)
C122	C72	C82	120.3(6)
C132	C162	H16D	109.5
C132	C162	H16E	109.5
C132	C162	H16F	109.5
H16D	C162	H16E	109.5
H16D	C162	H16F	109.5
H16E	C162	H16F	109.5
C132	C142	H14D	109.5
C132	C142	H14E	109.5
C132	C142	H14F	109.5
H14D	C142	H14E	109.5
H14D	C142	H14F	109.5
H14E	C142	H14F	109.5
C72	C122	H122	121.2
C72	C122	C112	117.5(7)
C112	C122	H122	121.2
C92	C102	H102	120.3
C112	C102	C92	119.3(6)
C112	C102	H102	120.3
C122	C112	H112	119.0
C102	C112	C122	122.1(6)
C102	C112	H112	119.0
C332	C352	H35D	109.5
C332	C352	H35E	109.5
C332	C352	H35F	109.5
H35D	C352	H35E	109.5
H35D	C352	H35F	109.5
H35E	C352	H35F	109.5
C332	C362	H36D	109.5
C332	C362	H36E	109.5
C332	C362	H36F	109.5
H36D	C362	H36E	109.5
H36D	C362	H36F	109.5
H36E	C362	H36F	109.5
C332	C342	H34D	109.5
C332	C342	H34E	109.5
C332	C342	H34F	109.5
H34D	C342	H34E	109.5
H34D	C342	H34F	109.5
H34E	C342	H34F	109.5
C372	C402	H40D	109.5
C372	C402	H40E	109.5
C372	C402	H40F	109.5
H40D	C402	H40E	109.5
H40D	C402	H40F	109.5
H40E	C402	H40F	109.5
C2S_2	C1S_2	H1S_2	120.0
C2S_2	C1S_2	C6S_2	119.96(8)

Atom	Atom	Atom	Angle/°
C6S_2	C1S_2	H1S_2	120.0
C1S_2	C2S_2	H2S_2	120.0
C3S_2	C2S_2	C1S_2	120.05(8)
C3S_2	C2S_2	H2S_2	120.0
C2S_2	C3S_2	H3S_2	120.0
C2S_2	C3S_2	C4S_2	119.98(8)
C4S_2	C3S_2	H3S_2	120.0
C3S_2	C4S_2	H4S_2	120.0
C3S_2	C4S_2	C5S_2	119.99(8)
C5S_2	C4S_2	H4S_2	120.0
C4S_2	C5S_2	H5S_2	120.0
C6S_2	C5S_2	C4S_2	120.02(8)
C6S_2	C5S_2	H5S_2	120.0
C1S_2	C6S_2	H6S_2	120.0
C5S_2	C6S_2	C1S_2	120.00(8)
C5S_2	C6S_2	H6S_2	120.0
C6S_1	C1S_1	H1S_1	120.0

Atom	Atom	Atom	Angle/°
C6S_1	C1S_1	C2S_1	120.01(7)
C2S_1	C1S_1	H1S_1	120.0
C1S_1	C6S_1	H6S_1	120.0
C1S_1	C6S_1	C5S_1	120.00(7)
C5S_1	C6S_1	H6S_1	120.0
C6S_1	C5S_1	H5S_1	120.0
C6S_1	C5S_1	C4S_1	120.00(7)
C4S_1	C5S_1	H5S_1	120.0
C5S_1	C4S_1	H4S_1	120.0
C3S_1	C4S_1	C5S_1	119.99(7)
C3S_1	C4S_1	H4S_1	120.0
C4S_1	C3S_1	H3S_1	120.0
C2S_1	C3S_1	C4S_1	120.02(7)
C2S_1	C3S_1	H3S_1	120.0
C1S_1	C2S_1	H2S_1	120.0
C3S_1	C2S_1	C1S_1	119.98(7)
C3S_1	C2S_1	H2S_1	120.0

Table S18: Torsion Angles in ° for ***cis*-[(^Ph^isq)(^Ph^ibq)V(O)Cl]**.

Atom	Atom	Atom	Atom	Angle/°
V1	O3	C22	C1	-4.7(5)
V1	O3	C22	C23	172.2(4)
V1	O2	C2	C21	16.7(5)
V1	O2	C2	C3	-166.4(4)
V1	N2	C21	C2	-15.0(5)
V1	N2	C21	C26	170.6(4)
V1	N2	C27	C28	-107.9(5)
V1	N2	C27	C32	67.4(6)
V1	N1	C1	C22	-1.2(6)
V1	N1	C1	C6	179.2(4)
V1	N1	C7	C12	120.3(6)
V1	N1	C7	C8	-54.5(8)
O3	C22	C23	C33	2.0(8)
O3	C22	C23	C24	-177.4(5)
O2	C2	C3	C4	-176.1(4)
O2	C2	C3	C13	3.5(7)
N2	C21	C2	O2	-0.5(6)
N2	C21	C2	C3	-177.5(4)
N2	C21	C26	C25	176.3(5)
N2	C27	C32	C31	-176.9(5)
N1	C1	C22	O3	3.9(6)
N1	C1	C22	C23	-173.2(4)
N1	C1	C6	C5	172.0(5)
N1	C7	C12	C11	-179.8(6)
N1	C7	C8	C9	179.1(8)
C25	C4	C3	C2	1.7(7)
C25	C4	C3	C13	-177.9(5)
C21	N2	C27	C28	63.3(6)
C21	N2	C27	C32	-121.5(5)
C21	C2	C3	C4	0.7(7)
C21	C2	C3	C13	-179.7(5)
C1	N1	C7	C12	-54.6(8)
C1	N1	C7	C8	130.6(7)
C1	C22	C23	C33	178.8(5)
C1	C22	C23	C24	-0.6(7)

Atom	Atom	Atom	Atom	Angle/˚
C1	C6	C5	C17	-174.7(5)
C1	C6	C5	C24	2.8(8)
C22	C1	C6	C5	-7.6(7)
C22	C23	C33	C36	-60.4(6)
C22	C23	C33	C34	-179.0(5)
C22	C23	C33	C35	61.1(6)
C22	C23	C24	C5	-4.5(8)
C2	C21	C26	C25	2.4(7)
C2	C3	C13	C14	-61.2(6)
C2	C3	C13	C15	-178.3(5)
C2	C3	C13	C16	59.3(6)
C4	C25	C26	C21	0.0(7)
C4	C25	C37	C39	64.7(6)
C4	C25	C37	C38	-175.7(5)
C4	C25	C37	C40	-55.0(6)
C4	C3	C13	C14	118.4(5)
C4	C3	C13	C15	1.2(7)
C4	C3	C13	C16	-121.1(6)
C26	C25	C4	C3	-2.1(8)
C26	C25	C37	C39	-115.0(5)
C26	C25	C37	C38	4.7(7)
C26	C25	C37	C40	125.3(5)
C26	C21	C2	O2	174.3(4)
C26	C21	C2	C3	-2.7(7)
C28	C27	C32	C31	-1.6(8)
C28	C29	C30	C31	0.2(9)
C27	N2	C21	C2	173.0(4)
C27	N2	C21	C26	-1.5(7)
C27	C28	C29	C30	-1.3(8)
C27	C32	C31	C30	0.5(9)
C37	C25	C4	C3	178.3(5)
C37	C25	C26	C21	179.6(4)
C6	C1	C22	O3	-176.5(4)
C6	C1	C22	C23	6.4(7)
C6	C5	C17	C19	111.9(7)
C6	C5	C17	C18	-130.0(7)
C6	C5	C17	C20	-11.0(9)
C6	C5	C24	C23	3.6(9)
C29	C28	C27	N2	177.1(5)
C29	C28	C27	C32	2.0(8)
C7	N1	C1	C22	173.8(4)
C7	N1	C1	C6	-5.8(8)
C7	C12	C11	C10	2.8(12)
C7	C8	C9	C10	-1.3(15)
C32	C31	C30	C29	0.2(9)
C12	C7	C8	C9	4.3(13)
C17	C5	C24	C23	-178.7(5)
C33	C23	C24	C5	176.1(5)
C8	C7	C12	C11	-5.1(11)
C9	C10	C11	C12	0.1(15)
C24	C23	C33	C36	118.9(6)
C24	C23	C33	C34	0.3(8)
C24	C23	C33	C35	-119.6(6)
C24	C5	C17	C19	-65.7(7)
C24	C5	C17	C18	52.4(7)
C24	C5	C17	C20	171.4(6)
C11	C10	C9	C8	-0.9(16)
V12	O32	C222	C212	25.0(5)
V12	O32	C222	C232	-156.7(4)

Atom	Atom	Atom	Atom	Angle/˚
V12	O22	C1B	C1A	167.7(4)
V12	O22	C1B	C1C	-8.2(5)
V12	N22	C272	C282	68.3(6)
V12	N22	C272	C322	-107.1(5)
V12	N22	C212	C222	-14.1(5)
V12	N22	C212	C262	167.1(5)
V12	N12	C1C	C62	175.6(4)
V12	N12	C1C	C1B	-3.6(6)
V12	N12	C72	C82	109.4(5)
V12	N12	C72	C122	-66.7(6)
O32	C222	C212	N22	-6.5(6)
O32	C222	C212	C262	172.5(5)
O32	C222	C232	C242	-172.2(5)
O32	C222	C232	C332	8.1(7)
O22	C1B	C1C	N12	7.8(7)
O22	C1B	C1C	C62	-171.4(4)
N22	C272	C282	C292	-177.1(5)
N22	C272	C322	C312	175.0(5)
N22	C212	C262	C252	-179.2(5)
N12	C72	C122	C112	179.2(6)
C222	C212	C262	C252	2.0(8)
C222	C232	C242	C252	-2.6(8)
C222	C232	C332	C352	49.0(7)
C222	C232	C332	C362	166.3(5)
C222	C232	C332	C342	-73.9(7)
C272	N22	C212	C222	176.1(4)
C272	N22	C212	C262	-2.8(8)
C272	C282	C292	C302	1.6(9)
C272	C322	C312	C302	2.4(8)
C212	N22	C272	C282	-123.2(5)
C212	N22	C272	C322	61.5(7)
C212	C222	C232	C242	6.0(7)
C212	C222	C232	C332	-173.7(5)
C232	C222	C212	N22	175.1(4)
C232	C222	C212	C262	-5.9(7)
C232	C242	C252	C372	179.5(5)
C232	C242	C252	C262	-1.1(9)
C1A	C42	C52	C62	7.8(8)
C1A	C42	C52	C172	-173.1(5)
C1A	C1B	C1C	N12	-167.7(5)
C1A	C1B	C1C	C62	13.1(8)
C282	C272	C322	C312	-0.3(8)
C322	C272	C282	C292	-1.7(8)
C42	C1A	C1B	O22	175.6(4)
C42	C1A	C1B	C1C	-9.0(8)
C42	C1A	C132	C152	-115.0(5)
C42	C1A	C132	C162	5.4(7)
C42	C1A	C132	C142	125.0(5)
C242	C232	C332	C352	-130.7(6)
C242	C232	C332	C362	-13.4(7)
C242	C232	C332	C342	106.4(6)
C242	C252	C262	C212	1.3(8)
C82	C92	C102	C112	-0.6(9)
C82	C72	C122	C112	3.2(9)
C92	C82	C72	N12	-178.3(5)
C92	C82	C72	C122	-2.3(9)
C92	C102	C112	C122	1.6(10)
C312	C302	C292	C282	0.5(9)
C292	C302	C312	C322	-2.6(9)

Atom	Atom	Atom	Atom	Angle/˚
C372	C252	C262	C212	-179.3(5)
C382	C372	C252	C242	-75.2(7)
C382	C372	C252	C262	105.4(7)
C192	C172	C52	C42	60.0(8)
C192	C172	C52	C62	-120.9(7)
C392	C372	C252	C242	43.4(8)
C392	C372	C252	C262	-136.0(6)
C202	C172	C52	C42	179.1(6)
C202	C172	C52	C62	-1.8(9)
C182	C172	C52	C42	-61.4(8)
C182	C172	C52	C62	117.7(7)
C52	C62	C1C	N12	175.0(5)
C52	C62	C1C	C1B	-5.8(8)
C1B	C1A	C42	C52	-1.5(7)
C1B	C1A	C132	C152	63.1(7)
C1B	C1A	C132	C162	-176.5(5)
C1B	C1A	C132	C142	-56.9(7)
C332	C232	C242	C252	177.1(5)
C132	C1A	C42	C52	176.8(5)
C132	C1A	C1B	O22	-2.6(8)
C132	C1A	C1B	C1C	172.8(5)
C1C	N12	C72	C82	-64.4(7)
C1C	N12	C72	C122	119.6(6)
C1C	C62	C52	C42	-3.6(8)
C1C	C62	C52	C172	177.3(5)
C72	N12	C1C	C62	-10.6(8)
C72	N12	C1C	C1B	170.3(5)
C72	C82	C92	C102	1.0(9)
C72	C122	C112	C102	-2.9(10)
C402	C372	C252	C242	161.3(7)
C402	C372	C252	C262	-18.1(10)
C1S_2	C2S_2	C3S_2	C4S_2	0.00(5)
C2S_2	C1S_2	C6S_2	C5S_2	0.00(5)
C2S_2	C3S_2	C4S_2	C5S_2	0.00(10)
C3S_2	C4S_2	C5S_2	C6S_2	0.00(12)
C4S_2	C5S_2	C6S_2	C1S_2	0.00(10)
C6S_2	C1S_2	C2S_2	C3S_2	0.00(2)
C1S_1	C6S_1	C5S_1	C4S_1	0.00(7)
C6S_1	C1S_1	C2S_1	C3S_1	0.01(6)
C6S_1	C5S_1	C4S_1	C3S_1	0.00(8)
C5S_1	C4S_1	C3S_1	C2S_1	0.01(7)
C4S_1	C3S_1	C2S_1	C1S_1	-0.01(7)
C2S_1	C1S_1	C6S_1	C5S_1	0.00(4)

Table S19: Hydrogen Fractional Atomic Coordinates (×10^4) and Equivalent Isotropic Displacement Parameters (Å^2×10^3) for ***cis*-[(^{Ph}isq)(^{Ph}ibq)V(O)Cl]**. U_{eq} is defined as 1/3 of the trace of the orthogonalised U_{ij}.

Atom	x	y	z	U_{eq}
H4	10409.58	3912.7	7269.39	41
H26	9717.85	6121.55	8010.05	36
H28	9411.87	7521.1	7514.42	41
H6	7914.19	9236.49	6344.11	53
H29	9278.19	8642.75	8181	50
H39A	11342.63	4653.44	7550.66	64
H39B	11759.28	5089.83	8167.23	64

Atom	x	y	z	U_{eq}
H39C	11290.74	5769.35	7682.83	64
H32	7667.33	6313.66	7805.24	50
H31	7529.21	7447.33	8463.15	60
H38A	10717.69	6281.41	8394.26	68
H38B	11142.05	5567.73	8882.38	68
H38C	10281.8	5561.91	8749.78	68
H14A	8707.11	2643.32	6158.31	74
H14B	8367.56	3569.01	6415.9	74
H14C	8833.47	2838.07	6867.13	74
H12	7890.12	8611.94	7289.38	58
H15A	10039.31	2755.94	6917.97	75
H15B	10412.84	3319.36	6436.13	75
H15C	9859.82	2473.99	6221.61	75
H36A	10449.61	7269.98	6908.56	74
H36B	10769.91	6529.92	6482.93	74
H36C	9963.54	6408.35	6605.21	74
H40A	10349.32	3956.19	8574.79	70
H40B	11205.84	4059.64	8714.25	70
H40C	10824.42	3593.4	8094.89	70
H16A	9275.78	3565.48	5563.6	76
H16B	9784.41	4420.42	5845.89	76
H16C	8932.32	4495.27	5812.74	76
H19A	9229.47	10295.81	7106.1	108
H19B	9432.41	11229.22	6763.11	108
H19C	9872.41	10262.44	6725.49	108
H34A	10474.64	8462.56	5631.17	103
H34B	11054.49	7681.69	5909.22	103
H34C	10763.59	8441.59	6339.72	103
H8	6662.21	7106.89	6118	94
H18A	9643.03	10301.14	5714.32	108
H18B	9239.75	11293.82	5763.91	108
H18C	8836.93	10444.8	5369.84	108
H10	5850.55	8870.38	7120.11	118
H20A	7927.93	10465.22	5875.76	134
H20B	8322.71	11387.79	6191.69	134
H20C	8080.46	10547.22	6593.37	134
H9	5701.13	7780.15	6410.32	128
H30	8330.27	8606.78	8649.86	56
H24	9884.64	9164.2	6137.22	53
H35A	9521.45	6449.99	5596.43	89
H35B	10320.73	6552.31	5453.96	89
H35C	9721.93	7324.47	5200.42	89
H11	6927.41	9299.54	7558.75	84
H282	10119.28	7460.02	8661.29	49
H322	11913.11	8317.82	9580.28	47
H42	13001.91	10838.51	9739.99	48
H242	13333.02	6194.18	8188.59	50
H302	10258.79	8000.99	10350.95	60
H82	10480.49	9479.33	9416.06	53
H92	9390.21	9851.73	9580.49	61
H312	11392.26	8456.03	10384.43	54
H292	9620.96	7516.43	9486.09	58
H62	10978.16	11132.78	9159.26	52
H15D	13806.12	10316.97	8584.64	65
H15E	13123.33	9740.54	8248.56	65
H15F	13861.99	9193.41	8480.55	65
H38D	13105.3	4785.03	8663.35	98
H38E	12500.64	4777.53	9083.02	98
H38F	13311.36	4495.55	9356.99	98

Atom	x	y	z	U_{eq}
H19D	12330	12695.61	9226.11	128
H19E	12960.51	12280.72	9722.47	128
H19F	12459.5	13122.84	9893.21	128
H39D	14164.67	5681.53	9557.35	116
H39E	13977.92	6683.49	9229.53	116
H39F	14004.66	5736.5	8838.79	116
H20D	11389.95	12918.38	9988.23	143
H20E	11018.21	11890.03	9939.4	143
H20F	11153.2	12431.32	9344.07	143
H18D	12724.46	11337.51	10444.75	153
H18E	11911.98	11066.43	10492.03	153
H18F	12212.31	12108.78	10680.52	153
H262	12097.4	6802.39	9322.44	47
H16D	13687.21	9793.9	9951.34	78
H16E	13908.46	10579.82	9501.95	78
H16F	14299.99	9564.46	9560.38	78
H14D	13741.73	8280.81	9218.98	77
H14E	12937.96	8307.47	8853.6	77
H14F	13081.32	8464.81	9566.24	77
H122	10020.55	10413.54	7792.61	71
H102	8604.4	10507.05	8851.12	74
H112	8928.18	10823.63	7981.13	78
H35D	13023.91	8490.85	7524.36	91
H35E	12276.6	8358.2	7077.75	91
H35F	13019.5	8220.46	6834.68	91
H36D	13612.79	7021.48	6935.44	86
H36E	13497.08	6176.48	7385.82	86
H36F	13797.83	7185.42	7646.58	86
H34D	11821.89	6887.52	6901.36	89
H34E	12354.76	5993.32	7003.5	89
H34F	12447.06	6776.74	6506.72	89
H40D	12572.48	5801.67	9844.73	177
H40E	13060.77	6735.75	9854.35	177
H40F	13419.59	5739.09	10081.52	177
H1S_2	8827.99	3949.61	10193.79	176
H2S_2	8144.91	5244.13	10180.07	179
H3S_2	8088.61	6285.11	9430.05	172
H4S_2	8715.73	6031.49	8693.25	168
H5S_2	9398.88	4737.06	8706.9	177
H6S_2	9455.05	3696.16	9456.94	188
H1S_1	8033.09	4043.11	9199.14	176
H6S_1	8203.7	5245.93	8596.48	175
H5S_1	9041.8	6335.58	8902.66	160
H4S_1	9709.31	6222.31	9811.63	201
H3S_1	9538.57	5019.49	10414.25	202
H2S_1	8700.65	3929.6	10108.08	176

Table S20: Atomic Occupancies for all atoms that are not fully occupied in ***cis*-[(^{Ph}isq)(^{Ph}ibq)V(O)Cl].**

Atom	Occupancy
C1S_2	0.347(9)
H1S_2	0.347(9)
C2S_2	0.347(9)
H2S_2	0.347(9)
C3S_2	0.347(9)
H3S_2	0.347(9)
C4S_2	0.347(9)
H4S_2	0.347(9)
C5S_2	0.347(9)
H5S_2	0.347(9)
C6S_2	0.347(9)
H6S_2	0.347(9)
C1S_1	0.653(9)
H1S_1	0.653(9)
C6S_1	0.653(9)
H6S_1	0.653(9)
C5S_1	0.653(9)
H5S_1	0.653(9)
C4S_1	0.653(9)
H4S_1	0.653(9)
C3S_1	0.653(9)
H3S_1	0.653(9)
C2S_1	0.653(9)
H2S_1	0.653(9)

Crystal data and experimental for $C_{30}H_{37}Cl_6N_2O_2Sb$

A black red, plate-shaped crystal of b368_kso2 was mounted on a nylon loop with NVH oil. Data were collected from a shock-cooled single crystal at 100.00 K on a Bruker D8 VENTURE dual wavelength Mo/Cu three-circle diffractometer with a microfocus sealed X-ray tube using a mirror optics as monochromator and a Bruker PHOTON III detector. The diffractometer was equipped with an Oxford Cryostream 800 low temperature device and used MoK_α radiation (λ = 0.71073 Å). All data were integrated with SAINT and a multi-scan absorption correction using SADABS was applied.[3,9] The structure was solved by direct methods using SHELXT and refined by full-matrix least-squares methods against F^2 by SHELXL using Olex2.[4,5,10] All non-hydrogen atoms were refined with anisotropic displacement parameters. All C-bound hydrogen atoms were refined isotropic on calculated positions using a riding model with their U_{iso} values constrained to 1.5 times the U_{eq} of their pivot atoms for terminal sp^3 carbon atoms and 1.2 times for all other carbon atoms. Disordered moieties were refined using bond lengths restraints and displacement parameter restraints. Crystallographic data for the structures reported here have been deposited with the Cambridge Crystallographic Data Centre.[11] CCDC 2411785 contain the supplementary crystallographic data for this paper. These data can be obtained free of charge from The Cambridge Crystallographic Data Centre via www.ccdc.cam.ac.uk/structures. This report and the CIF file were generated using FinalCif.[12]

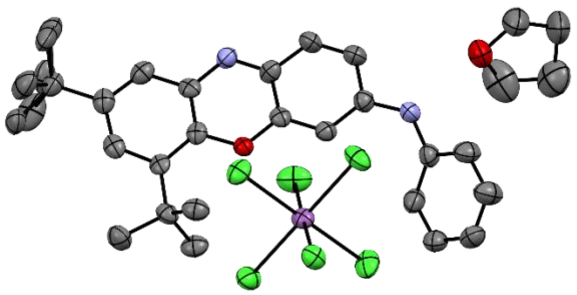

ORTEP plot of $C_{30}H_{37}Cl_6N_2O_2Sb$, ellipsoids drawn at 50% thermal probability.

Crystal data and structure refinement for $C_{30}H_{37}Cl_6N_2O_2Sb$

CCDC number	
Empirical formula	$C_{30}H_{37}Cl_6N_2O_2Sb$
Formula weight	792.06
Temperature [K]	100.00
Crystal system	monoclinic
Space group (number)	$P2_1/c$ (14)
a [Å]	17.833(4)
b [Å]	10.044(2)
c [Å]	20.994(4)
α [°]	90
β [°]	112.017(12)
γ [°]	90
Volume [Å^3]	3486.2(12)
Z	4
ρ_{calc} [gcm^{-3}]	1.509
μ [mm^{-1}]	1.281
F(000)	1600
Crystal size [mm^3]	0.299×0.107×0.06
Crystal colour	black red
Crystal shape	plate
Radiation	MoK_α (λ=0.71073 Å)
2θ range [°]	3.98 to 50.70 (0.83 Å)
Index ranges	$-21 \le h \le 21$<br>$-12 \le k \le 12$<br>$-25 \le l \le 24$
Reflections collected	62513
Independent reflections	6374<br>R_{int} = 0.1411<br>R_{sigma} = 0.0644
Completeness to θ = 25.242°	100.0 %
Data / Restraints / Parameters	6374/3/407
Goodness-of-fit on F^2	1.056
Final R indexes [$I \geq 2\sigma(I)$]	R_1 = 0.0614<br>wR_2 = 0.1157
Final R indexes [all data]	R_1 = 0.1060<br>wR_2 = 0.1391
Largest peak/hole [eÅ^{-3}]	1.59/-1.09

Table S21. Atomic coordinates and U_{eq} [Å^2] for $C_{30}H_{37}Cl_6N_2O_2Sb$

Atom	*x*	*y*	*z*	U_{eq}
Sb1	0.25622(3)	0.27128(4)	0.48844(3)	0.03652(15)
Cl5	0.21770(11)	0.36665(19)	0.57434(10)	0.0460(5)
Cl6	0.22291(12)	0.46887(17)	0.42240(10)	0.0485(5)
Cl2	0.38922(11)	0.35445(18)	0.54714(12)	0.0536(5)
Cl1	0.12416(12)	0.1903(2)	0.43078(10)	0.0521(5)
Cl4	0.29104(13)	0.07192(18)	0.55356(11)	0.0591(6)
Cl3	0.29766(14)	0.1748(2)	0.40474(13)	0.0630(6)
O1	0.2425(3)	0.7495(4)	0.5089(2)	0.0311(10)
N2	0.4917(3)	0.6195(6)	0.6815(3)	0.0404(14)
H2	0.544068	0.615266	0.691148	0.049
O2	0.6497(3)	0.5557(6)	0.7112(3)	0.0653(16)
N1	0.3251(3)	0.7970(6)	0.4213(3)	0.0387(14)
C12	0.1997(4)	0.7931(6)	0.4426(3)	0.0329(15)
C11	0.1169(4)	0.8121(6)	0.4220(4)	0.0331(16)
C1	0.3231(4)	0.7297(6)	0.5309(3)	0.0338(15)
C10	0.0803(4)	0.8609(6)	0.3548(4)	0.0389(18)
H10	0.023617	0.875766	0.338353	0.047
C6	0.3635(4)	0.7548(6)	0.4838(4)	0.0377(16)
C7	0.2432(4)	0.8180(6)	0.4001(4)	0.0345(16)
C23	0.0688(4)	0.7830(7)	0.4669(4)	0.0385(17)
C2	0.3638(4)	0.6877(6)	0.5967(3)	0.0350(16)
H2A	0.336174	0.674437	0.627091	0.042
C8	0.2013(5)	0.8667(7)	0.3328(4)	0.0426(18)
H8	0.230195	0.883433	0.303681	0.051
C4	0.4886(4)	0.6881(7)	0.5723(4)	0.0428(18)
H4	0.545237	0.672711	0.587580	0.051
C5	0.4484(4)	0.7306(7)	0.5090(4)	0.0431(18)
H5	0.476910	0.745468	0.479410	0.052
C3	0.4466(4)	0.6648(7)	0.6185(4)	0.0395(17)
C13	0.4659(4)	0.5777(7)	0.7340(4)	0.0418(18)
C9	0.1199(4)	0.8901(7)	0.3091(4)	0.0418(18)
C24	0.0716(5)	0.6348(7)	0.4830(4)	0.0464(19)
H24A	0.127955	0.607019	0.506811	0.070
H24B	0.047558	0.584412	0.440159	0.070
H24C	0.041143	0.617575	0.512503	0.070
C14	0.3986(5)	0.4960(7)	0.7222(4)	0.050(2)
H14	0.366568	0.468521	0.676821	0.060
C18	0.5134(5)	0.6126(8)	0.8008(4)	0.053(2)
H18	0.560208	0.666011	0.809795	0.063
C15	0.3789(5)	0.4559(8)	0.7759(4)	0.049(2)
H15	0.332612	0.401548	0.767887	0.059
C19	0.0744(5)	0.9477(7)	0.2381(4)	0.0447(19)
C25	0.1036(5)	0.8609(7)	0.5345(4)	0.0473(19)
H25A	0.069440	0.846959	0.561164	0.071
H25B	0.105155	0.955932	0.524563	0.071
H25C	0.158575	0.829438	0.561032	0.071
C16	0.4264(5)	0.4943(8)	0.8423(4)	0.053(2)
H16	0.412191	0.467075	0.879671	0.064
C26	-0.0197(4)	0.8228(8)	0.4311(4)	0.053(2)
H26A	-0.042908	0.776333	0.386930	0.079

H26B	-0.023369	0.919163	0.423228	0.079
H26C	-0.049664	0.798429	0.460082	0.079
C17	0.4926(5)	0.5700(9)	0.8541(5)	0.060(2)
H17	0.525344	0.594219	0.899840	0.073
C27	0.7276(6)	0.6041(10)	0.7540(5)	0.079(3)
H27A	0.724488	0.653664	0.793729	0.095
H27B	0.749883	0.663579	0.727916	0.095
C29	0.7191(7)	0.3783(11)	0.7796(7)	0.097(4)
H29A	0.739225	0.286805	0.778669	0.117
H29B	0.704211	0.389111	0.820267	0.117
C28	0.7790(7)	0.4814(13)	0.7776(7)	0.109(5)
H28A	0.804446	0.456307	0.744751	0.131
H28B	0.821711	0.494710	0.823613	0.131
C30	0.6503(7)	0.4123(11)	0.7143(7)	0.104(4)
H30A	0.658926	0.373616	0.674228	0.125
H30B	0.598575	0.378358	0.715165	0.125
C22	-0.0159(7)	0.9748(14)	0.2254(7)	0.051(4)
H22A	-0.041886	1.019605	0.181095	0.076
H22B	-0.019344	1.031695	0.262179	0.076
H22C	-0.043447	0.890194	0.225050	0.076
C21	0.0785(11)	0.8533(16)	0.1846(7)	0.064(5)
H21A	0.052251	0.769319	0.187935	0.095
H21B	0.135233	0.836422	0.191785	0.095
H21C	0.050753	0.892208	0.138953	0.095
C20	0.1143(9)	1.0829(13)	0.2362(7)	0.063(5)
H20A	0.080893	1.132099	0.195017	0.095
H20B	0.168139	1.068018	0.235053	0.095
H20C	0.119389	1.134449	0.277207	0.095
C22A	0.0328(16)	1.076(2)	0.2432(13)	0.067(8)
H22D	0.002702	1.110727	0.197014	0.101
H22E	0.073440	1.141601	0.269433	0.101
H22F	-0.004612	1.059395	0.266618	0.101
C20A	0.1330(13)	0.988(3)	0.2007(12)	0.065(8)
H20D	0.102081	1.034201	0.157551	0.097
H20E	0.158146	0.908378	0.190826	0.097
H20F	0.175108	1.048040	0.230400	0.097
C21A	0.0215(19)	0.844(2)	0.1925(13)	0.078(10)
H21D	-0.018509	0.814780	0.211168	0.117
H21E	0.054614	0.767988	0.189805	0.117
H21F	-0.006302	0.881231	0.146444	0.117

U_{eq} is defined as 1/3 of the trace of the orthogonalized U_{ij} tensor.

Table S22. Anisotropic displacement parameters [Å^2] for $C_{30}H_{37}Cl_6N_2O_2Sb$. The anisotropic displacement factor exponent takes the form: $-2\pi^2[h^2(a^*)^2U_{11} + k^2(b^*)^2U_{22} + \ldots + 2hka^*b^*U_{12}]$

Atom	U_{11}	U_{22}	U_{33}	U_{23}	U_{13}	U_{12}
Sb1	0.0393(3)	0.0252(2)	0.0455(3)	−0.0041(2)	0.0164(2)	−0.0024(2)
Cl5	0.0449(11)	0.0487(11)	0.0442(11)	−0.0106(9)	0.0165(9)	−0.0039(8)
Cl6	0.0642(13)	0.0299(9)	0.0507(12)	0.0014(8)	0.0208(10)	0.0014(8)
Cl2	0.0357(10)	0.0392(10)	0.0818(15)	−0.0092(10)	0.0174(10)	−0.0033(8)
Cl1	0.0481(11)	0.0583(12)	0.0442(11)	−0.0060(9)	0.0106(9)	−0.0177(9)

Cl4	0.0656(14)	0.0299(10)	0.0675(14)	0.0069(9)	0.0085(11)	−0.0014(9)
Cl3	0.0818(15)	0.0429(11)	0.0837(16)	−0.0201(11)	0.0534(14)	−0.0054(10)
O1	0.031(2)	0.026(2)	0.035(2)	−0.002(2)	0.0111(19)	−0.0013(19)
N2	0.030(3)	0.051(4)	0.039(4)	0.004(3)	0.011(3)	−0.002(3)
O2	0.047(4)	0.074(4)	0.067(4)	0.010(3)	0.012(3)	0.004(3)
N1	0.042(3)	0.039(3)	0.037(4)	0.005(3)	0.017(3)	0.003(3)
C12	0.040(4)	0.019(3)	0.036(4)	−0.005(3)	0.011(3)	0.002(3)
C11	0.038(4)	0.014(3)	0.044(4)	0.000(3)	0.012(3)	0.000(3)
C1	0.035(4)	0.024(3)	0.040(4)	−0.005(3)	0.011(3)	−0.005(3)
C10	0.038(4)	0.023(3)	0.050(5)	−0.007(3)	0.010(4)	0.001(3)
C6	0.045(4)	0.033(4)	0.039(4)	0.001(3)	0.019(3)	0.005(3)
C7	0.037(4)	0.021(3)	0.046(4)	−0.004(3)	0.016(3)	−0.003(3)
C23	0.033(4)	0.033(4)	0.048(4)	−0.004(3)	0.013(3)	−0.004(3)
C2	0.038(4)	0.033(4)	0.033(4)	−0.004(3)	0.012(3)	−0.007(3)
C8	0.055(5)	0.033(4)	0.042(5)	−0.001(3)	0.020(4)	0.000(3)
C4	0.034(4)	0.049(4)	0.048(5)	0.002(4)	0.018(4)	−0.003(3)
C5	0.033(4)	0.049(4)	0.049(5)	0.002(4)	0.017(4)	−0.001(3)
C3	0.038(4)	0.040(4)	0.036(4)	−0.001(3)	0.010(3)	−0.002(3)
C13	0.038(4)	0.045(4)	0.036(4)	0.000(3)	0.006(3)	−0.002(3)
C9	0.046(4)	0.032(4)	0.042(4)	−0.004(3)	0.009(4)	0.002(3)
C24	0.058(5)	0.030(4)	0.058(5)	−0.004(4)	0.029(4)	−0.006(3)
C14	0.053(5)	0.044(4)	0.043(5)	0.006(4)	0.007(4)	−0.005(4)
C18	0.051(5)	0.054(5)	0.056(6)	−0.004(4)	0.023(4)	−0.015(4)
C15	0.048(5)	0.047(5)	0.051(5)	0.004(4)	0.017(4)	−0.009(4)
C19	0.054(5)	0.040(4)	0.036(4)	0.004(3)	0.012(4)	−0.002(3)
C25	0.050(5)	0.039(4)	0.058(5)	−0.008(4)	0.026(4)	−0.007(3)
C16	0.057(5)	0.059(5)	0.048(5)	0.007(4)	0.026(4)	−0.004(4)
C26	0.040(4)	0.058(5)	0.062(6)	0.002(4)	0.021(4)	0.006(4)
C17	0.057(5)	0.074(6)	0.054(6)	−0.010(5)	0.025(5)	−0.019(5)
C27	0.064(6)	0.076(7)	0.085(8)	0.021(6)	0.012(6)	−0.007(5)
C29	0.110(10)	0.072(7)	0.114(10)	0.013(7)	0.047(8)	0.028(7)
C28	0.064(7)	0.130(11)	0.113(10)	0.062(9)	0.009(7)	0.014(7)
C30	0.091(9)	0.088(9)	0.122(11)	−0.044(8)	0.027(8)	0.016(7)
C22	0.045(8)	0.057(9)	0.047(8)	0.016(7)	0.015(6)	0.015(6)
C21	0.072(11)	0.075(11)	0.039(8)	0.000(7)	0.015(8)	0.029(9)
C20	0.068(10)	0.058(9)	0.048(9)	0.022(7)	0.005(7)	−0.009(8)
C22A	0.079(19)	0.060(16)	0.068(17)	0.028(13)	0.035(15)	0.026(14)
C20A	0.077(17)	0.057(16)	0.055(16)	0.022(13)	0.019(13)	0.004(13)
C21A	0.12(3)	0.047(15)	0.059(18)	−0.005(13)	0.019(18)	0.008(17)

Table S23. Bond lengths and angles for $C_{30}H_{37}Cl_6N_2O_2Sb$

Atom–Atom	Length [Å]
Sb1–Cl5	2.3603(19)
Sb1–Cl6	2.3656(19)
Sb1–Cl2	2.3771(19)
Sb1–Cl1	2.3528(19)
Sb1–Cl4	2.372(2)
Sb1–Cl3	2.355(2)
O1–C12	1.385(8)
O1–C1	1.349(7)
N2–H2	0.8800
N2–C3	1.343(9)
N2–C13	1.408(9)
O2–C27	1.427(10)
O2–C30	1.442(12)
N1–C6	1.302(9)
N1–C7	1.374(8)
C12–C11	1.388(9)
C12–C7	1.406(9)
C11–C10	1.401(9)
C11–C23	1.521(10)

C1–C6	1.448(9)
C1–C2	1.364(9)
C10–H10	0.9500
C10–C9	1.420(10)
C6–C5	1.425(9)
C7–C8	1.415(10)
C23–C24	1.523(9)
C23–C25	1.534(10)
C23–C26	1.525(9)
C2–H2A	0.9500
C2–C3	1.393(9)
C8–H8	0.9500
C8–C9	1.368(10)
C4–H4	0.9500
C4–C5	1.323(10)
C4–C3	1.449(10)
C5–H5	0.9500
C13–C14	1.399(10)
C13–C18	1.385(10)
C9–C19	1.518(10)
C24–H24A	0.9800
C24–H24B	0.9800
C24–H24C	0.9800
C14–H14	0.9500
C14–C15	1.361(10)
C18–H18	0.9500
C18–C17	1.371(11)
C15–H15	0.9500
C15–C16	1.388(11)
C19–C22	1.554(13)
C19–C21	1.493(13)
C19–C20	1.541(13)
C19–C22A	1.511(17)
C19–C20A	1.579(18)
C19–C21A	1.488(18)
C25–H25A	0.9800
C25–H25B	0.9800
C25–H25C	0.9800
C16–H16	0.9500
C16–C17	1.348(11)
C26–H26A	0.9800
C26–H26B	0.9800
C26–H26C	0.9800
C17–H17	0.9500
C27–H27A	0.9900
C27–H27B	0.9900
C27–C28	1.504(14)
C29–H29A	0.9900
C29–H29B	0.9900
C29–C28	1.500(15)
C29–C30	1.496(15)
C28–H28A	0.9900
C28–H28B	0.9900

C30–H30A	0.9900
C30–H30B	0.9900
C22–H22A	0.9800
C22–H22B	0.9800
C22–H22C	0.9800
C21–H21A	0.9800
C21–H21B	0.9800
C21–H21C	0.9800
C20–H20A	0.9800
C20–H20B	0.9800
C20–H20C	0.9800
C22A–H22D	0.9800
C22A–H22E	0.9800
C22A–H22F	0.9800
C20A–H20D	0.9800
C20A–H20E	0.9800
C20A–H20F	0.9800
C21A–H21D	0.9800
C21A–H21E	0.9800
C21A–H21F	0.9800
**Atom–Atom–Atom**	**Angle [°]**
Cl5–Sb1–Cl6	91.50(7)
Cl5–Sb1–Cl2	88.91(7)
Cl5–Sb1–Cl4	89.45(8)
Cl6–Sb1–Cl2	89.51(7)
Cl6–Sb1–Cl4	178.99(8)
Cl1–Sb1–Cl5	90.65(7)
Cl1–Sb1–Cl6	90.32(7)
Cl1–Sb1–Cl2	179.53(8)
Cl1–Sb1–Cl4	90.00(7)
Cl1–Sb1–Cl3	90.43(8)
Cl4–Sb1–Cl2	90.18(7)
Cl3–Sb1–Cl5	178.64(8)
Cl3–Sb1–Cl6	89.31(8)
Cl3–Sb1–Cl2	90.01(8)
Cl3–Sb1–Cl4	89.72(8)
C1–O1–C12	120.3(5)
C3–N2–H2	115.8
C3–N2–C13	128.5(6)
C13–N2–H2	115.8
C27–O2–C30	108.8(7)
C6–N1–C7	118.1(6)
O1–C12–C11	118.4(6)
O1–C12–C7	117.9(6)
C11–C12–C7	123.7(6)
C12–C11–C10	113.6(6)
C12–C11–C23	124.2(6)
C10–C11–C23	122.2(6)
O1–C1–C6	118.7(6)
O1–C1–C2	119.1(6)
C2–C1–C6	122.2(6)

C11–C10–H10	116.9
C11–C10–C9	126.3(7)
C9–C10–H10	116.9
N1–C6–C1	122.4(6)
N1–C6–C5	120.9(7)
C5–C6–C1	116.7(6)
N1–C7–C12	122.6(6)
N1–C7–C8	118.4(6)
C12–C7–C8	119.0(6)
C11–C23–C24	110.3(6)
C11–C23–C25	110.4(6)
C11–C23–C26	111.4(6)
C24–C23–C25	108.9(6)
C24–C23–C26	107.9(6)
C26–C23–C25	107.9(6)
C1–C2–H2A	120.6
C1–C2–C3	118.7(7)
C3–C2–H2A	120.6
C7–C8–H8	119.5
C9–C8–C7	120.9(7)
C9–C8–H8	119.5
C5–C4–H4	119.9
C5–C4–C3	120.2(7)
C3–C4–H4	119.9
C6–C5–H5	119.1
C4–C5–C6	121.9(7)
C4–C5–H5	119.1
N2–C3–C2	123.2(7)
N2–C3–C4	116.4(6)
C2–C3–C4	120.3(7)
C14–C13–N2	123.2(7)
C18–C13–N2	117.6(7)
C18–C13–C14	119.0(7)
C10–C9–C19	121.6(7)
C8–C9–C10	116.6(7)
C8–C9–C19	121.8(7)
C23–C24–H24A	109.5
C23–C24–H24B	109.5
C23–C24–H24C	109.5
H24A–C24–H24B	109.5
H24A–C24–H24C	109.5
H24B–C24–H24C	109.5
C13–C14–H14	120.0
C15–C14–C13	119.9(7)
C15–C14–H14	120.0
C13–C18–H18	120.1
C17–C18–C13	119.9(7)
C17–C18–H18	120.1
C14–C15–H15	120.0
C14–C15–C16	120.0(7)
C16–C15–H15	120.0
C9–C19–C22	111.9(7)
C9–C19–C20	106.2(7)

C9–C19–C20A	112.1(10)
C21–C19–C9	109.7(8)
C21–C19–C22	108.8(10)
C21–C19–C20	112.7(11)
C20–C19–C22	107.5(9)
C22A–C19–C9	110.6(11)
C22A–C19–C20A	104.4(14)
C21A–C19–C9	110.0(12)
C21A–C19–C22A	115.7(17)
C21A–C19–C20A	103.8(17)
C23–C25–H25A	109.5
C23–C25–H25B	109.5
C23–C25–H25C	109.5
H25A–C25–H25B	109.5
H25A–C25–H25C	109.5
H25B–C25–H25C	109.5
C15–C16–H16	119.9
C17–C16–C15	120.3(8)
C17–C16–H16	119.9
C23–C26–H26A	109.5
C23–C26–H26B	109.5
C23–C26–H26C	109.5
H26A–C26–H26B	109.5
H26A–C26–H26C	109.5
H26B–C26–H26C	109.5
C18–C17–H17	119.6
C16–C17–C18	120.9(8)
C16–C17–H17	119.6
O2–C27–H27A	110.8
O2–C27–H27B	110.8
O2–C27–C28	104.9(8)
H27A–C27–H27B	108.8
C28–C27–H27A	110.8
C28–C27–H27B	110.8
H29A–C29–H29B	109.6
C28–C29–H29A	111.9
C28–C29–H29B	111.9
C30–C29–H29A	111.9
C30–C29–H29B	111.9
C30–C29–C28	99.5(10)
C27–C28–H28A	111.2
C27–C28–H28B	111.2
C29–C28–C27	102.8(9)
C29–C28–H28A	111.2
C29–C28–H28B	111.2
H28A–C28–H28B	109.1
O2–C30–C29	105.0(9)
O2–C30–H30A	110.7
O2–C30–H30B	110.7
C29–C30–H30A	110.7
C29–C30–H30B	110.7
H30A–C30–H30B	108.8
C19–C22–H22A	109.5

C19–C22–H22B	109.5
C19–C22–H22C	109.5
H22A–C22–H22B	109.5
H22A–C22–H22C	109.5
H22B–C22–H22C	109.5
C19–C21–H21A	109.5
C19–C21–H21B	109.5
C19–C21–H21C	109.5
H21A–C21–H21B	109.5
H21A–C21–H21C	109.5
H21B–C21–H21C	109.5
C19–C20–H20A	109.5
C19–C20–H20B	109.5
C19–C20–H20C	109.5
H20A–C20–H20B	109.5
H20A–C20–H20C	109.5
H20B–C20–H20C	109.5
C19–C22A–H22D	109.5
C19–C22A–H22E	109.5
C19–C22A–H22F	109.5
H22D–C22A–H22E	109.5
H22D–C22A–H22F	109.5
H22E–C22A–H22F	109.5
C19–C20A–H20D	109.5
C19–C20A–H20E	109.5
C19–C20A–H20F	109.5
H20D–C20A–H20E	109.5
H20D–C20A–H20F	109.5
H20E–C20A–H20F	109.5
C19–C21A–H21D	109.5
C19–C21A–H21E	109.5
C19–C21A–H21F	109.5
H21D–C21A–H21E	109.5
H21D–C21A–H21F	109.5
H21E–C21A–H21F	109.5

Table S24. Torsion angles for $C_{30}H_{37}Cl_6N_2O_2Sb$

Atom–Atom–Atom–Atom	Torsion Angle [°]
O1–C12–C11–C10	177.8(5)
O1–C12–C11–C23	−2.4(9)
O1–C12–C7–N1	1.9(9)
O1–C12–C7–C8	−178.0(5)
O1–C1–C6–N1	0.7(10)
O1–C1–C6–C5	−179.1(6)
O1–C1–C2–C3	178.6(6)
N2–C13–C14–C15	177.7(7)
N2–C13–C18–C17	−177.5(7)
O2–C27–C28–C29	−30.8(12)
N1–C6–C5–C4	179.8(7)
N1–C7–C8–C9	−179.6(6)
C12–O1–C1–C6	−0.1(8)
C12–O1–C1–C2	179.5(5)
C12–C11–C10–C9	0.2(9)
C12–C11–C23–C24	−64.0(8)
C12–C11–C23–C25	56.4(8)
C12–C11–C23–C26	176.3(6)
C12–C7–C8–C9	0.3(10)
C11–C12–C7–N1	−179.2(6)
C11–C12–C7–C8	0.9(9)
C11–C10–C9–C8	0.9(10)
C11–C10–C9–C19	−177.6(6)
C1–O1–C12–C11	179.9(5)
C1–O1–C12–C7	−1.2(8)
C1–C6–C5–C4	−0.4(11)
C1–C2–C3–N2	−177.8(6)
C1–C2–C3–C4	1.3(10)
C10–C11–C23–C24	115.8(7)
C10–C11–C23–C25	−123.8(7)
C10–C11–C23–C26	−4.0(9)
C10–C9–C19–C22	2.7(11)
C10–C9–C19–C21	−118.1(11)
C10–C9–C19–C20	119.8(10)
C10–C9–C19–C22A	56.8(14)
C10–C9–C19–C20A	172.8(12)
C10–C9–C19–C21A	−72.3(17)
C6–N1–C7–C12	−1.3(9)
C6–N1–C7–C8	178.6(6)
C6–C1–C2–C3	−1.9(10)
C7–N1–C6–C1	0.0(10)
C7–N1–C6–C5	179.8(6)
C7–C12–C11–C10	−1.0(9)
C7–C12–C11–C23	178.7(6)
C7–C8–C9–C10	−1.1(10)
C7–C8–C9–C19	177.4(6)
C23–C11–C10–C9	−179.6(6)
C2–C1–C6–N1	−178.8(6)
C2–C1–C6–C5	1.4(10)
C8–C9–C19–C22	−175.7(8)
C8–C9–C19–C21	63.4(12)
C8–C9–C19–C20	−58.6(11)
C8–C9–C19–C22A	−121.7(14)
C8–C9–C19–C20A	−5.6(14)

C8–C9–C19–C21A	109.3(17)
C5–C4–C3–N2	178.9(7)
C5–C4–C3–C2	−0.4(11)
C3–N2–C13–C14	43.9(11)
C3–N2–C13–C18	−140.6(8)
C3–C4–C5–C6	−0.1(11)
C13–N2–C3–C2	5.3(12)
C13–N2–C3–C4	−173.9(7)
C13–C14–C15–C16	−1.0(12)
C13–C18–C17–C16	0.0(14)
C14–C13–C18–C17	−1.8(12)
C14–C15–C16–C17	−0.8(13)
C18–C13–C14–C15	2.3(12)
C15–C16–C17–C18	1.3(14)
C27–O2–C30–C29	21.1(12)
C28–C29–C30–O2	−39.0(12)
C30–O2–C27–C28	6.2(12)
C30–C29–C28–C27	42.1(12)

Table S25. Hydrogen bonds for $C_{30}H_{37}Cl_6N_2O_2Sb$

D–H···A [Å]	d(D–H) [Å]	d(H···A) [Å]	d(D···A) [Å]	<(DHA) [°]
N2–H2···O2	0.88	1.87	2.725(8)	164.1

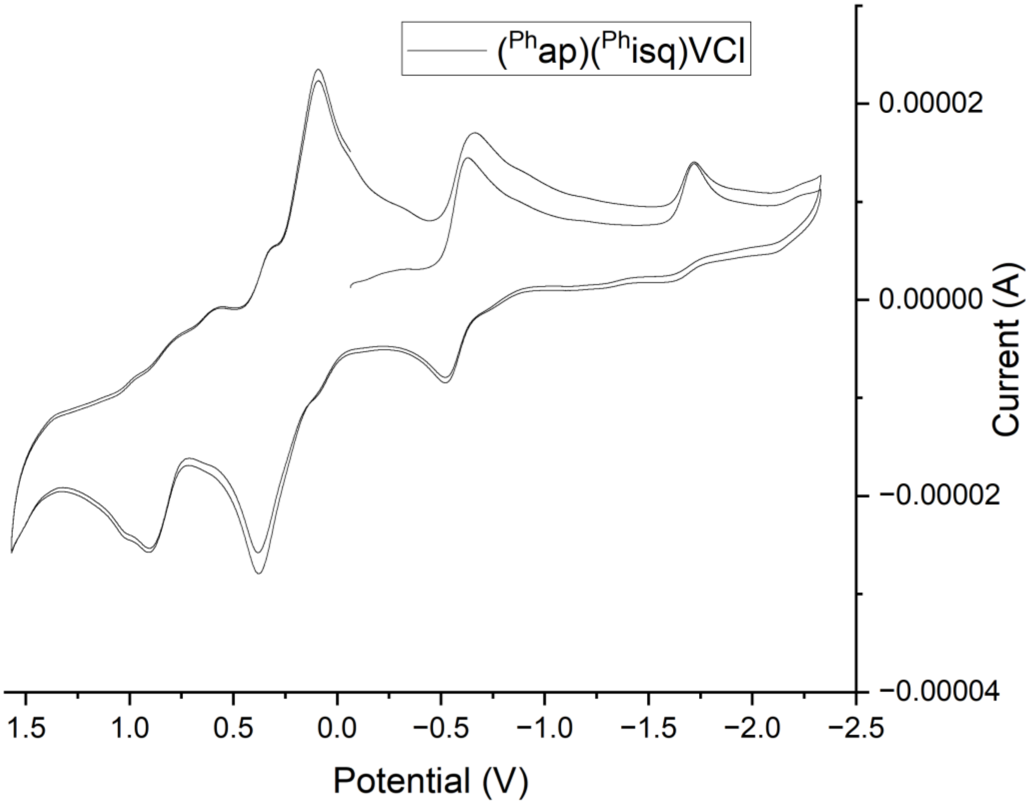

Figure S33: Cyclic voltammogram of complex **I** (1.5 mM) in acetonitrile, 0.1 M tetrabutylammonium hexafluorophosphate as supporting electrolyte.

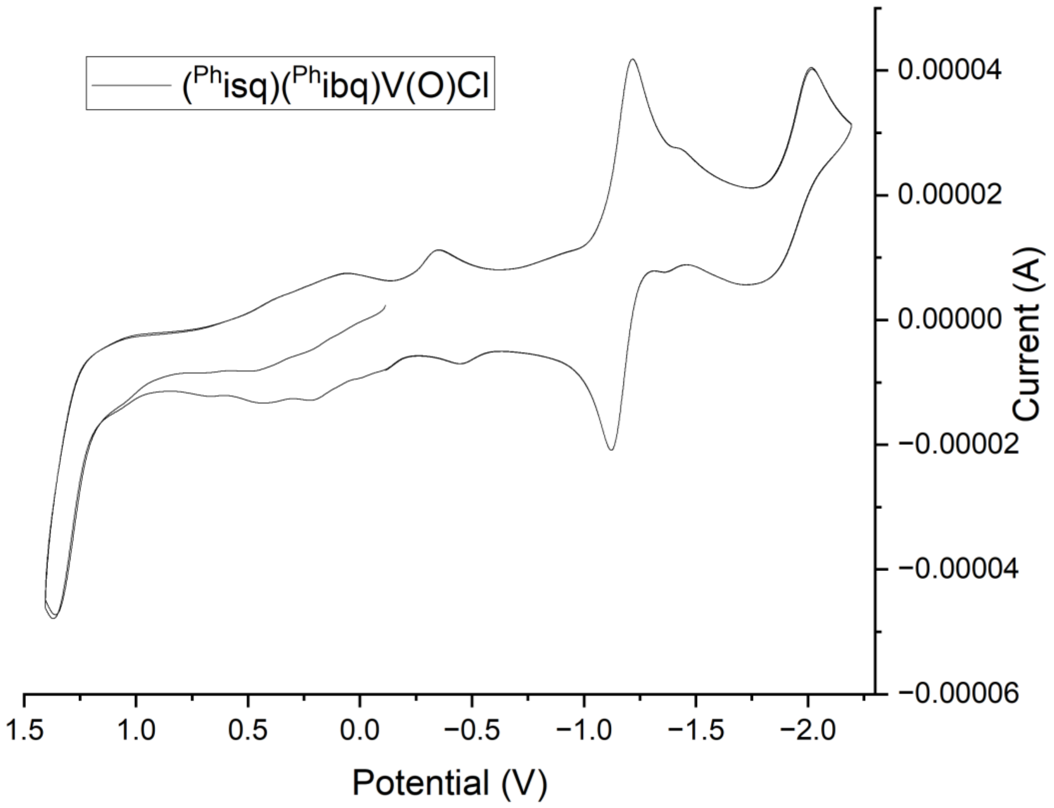

Figure S34: Cyclic voltammogram of complex **II** (1.5 mM) in acetonitrile, 0.1 M tetrabutylammonium hexafluorophosphate as supporting electrolyte.

Computational Data

Table S26. Computed spin on vanadium in crystal and implicit solvent environments and MOS values from the crystal structures and DFT optimized coordinates.

		**I** crystal	**I** solvate	**IIa** crystal	**IIa** solvate	**IIb** crystal	**IIb'** crystal
**S = 0**	**Spin on V (n)**	1.53	1.75	0.00	0.98	0.95	0.47
	**MOS1**	-1.25	-1.05	0.00	-0.05		-1.30
	**MOS2**	-1.35	-1.10	-1.35	-0.90		-1.20
**S = 1**	**Spin on V (n)**	1.68	1.90	0.95	0.97	0.82	0.40
	**MOS 1**		-1.00		-0.05	-0.10	
	**MOS2**		-1.00		-0.85	-1.25	

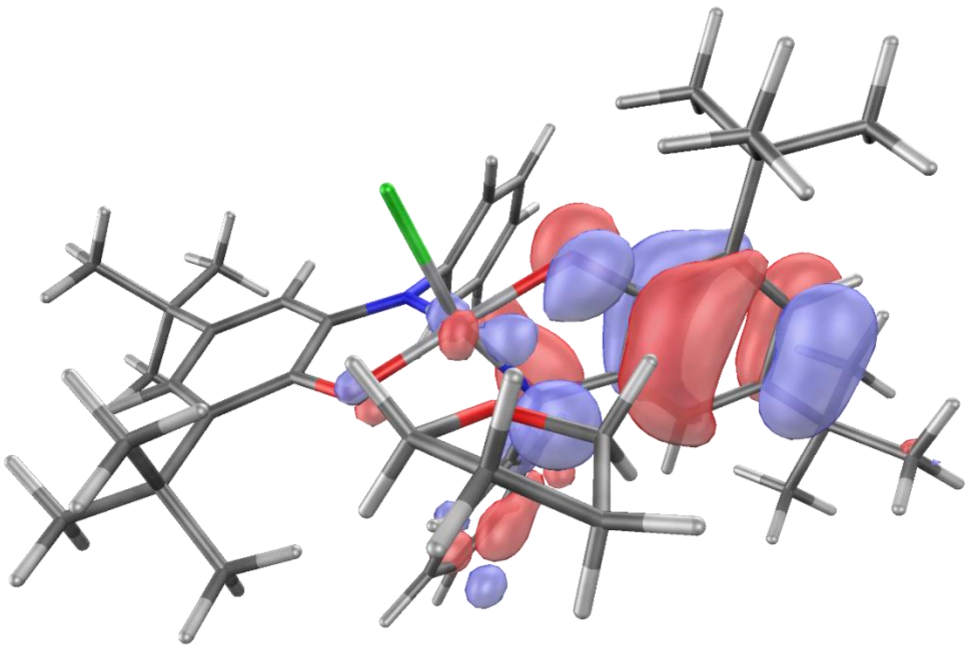

Figure S35. Calculated HOMO-1α of S = 0 [(^{Ph}ap)(^{Ph}isq)V^{IV}Cl] (**I**). Plot generated in IQmol, isosurface value 0.04.

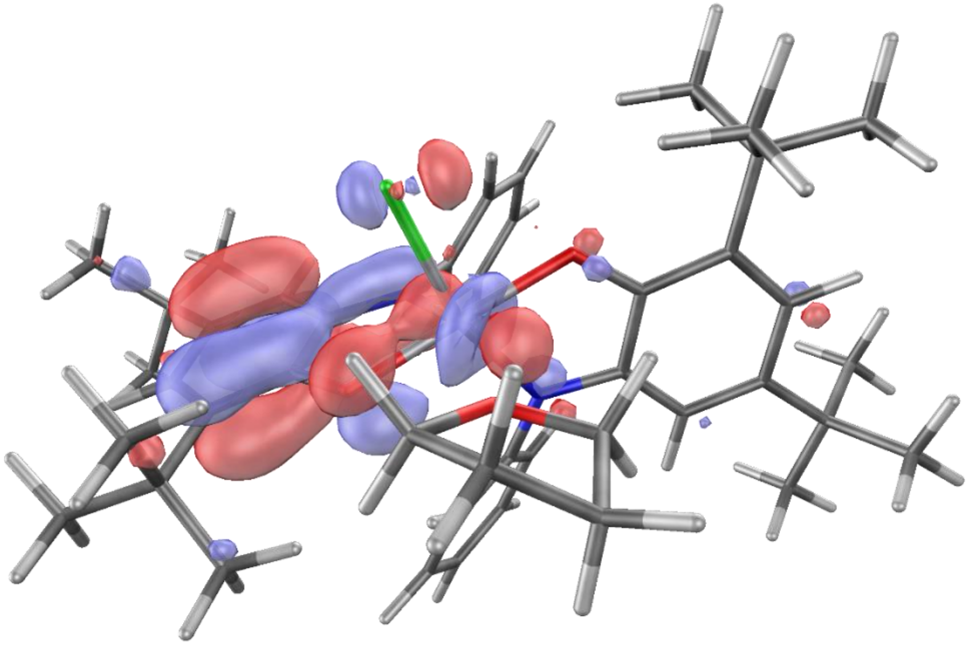

Figure S36. Calculated HOMO-1β of S = 0 [(^{Ph}ap)(^{Ph}isq)V^{IV}Cl] (**I**). Plot generated in IQmol, isosurface value 0.04.

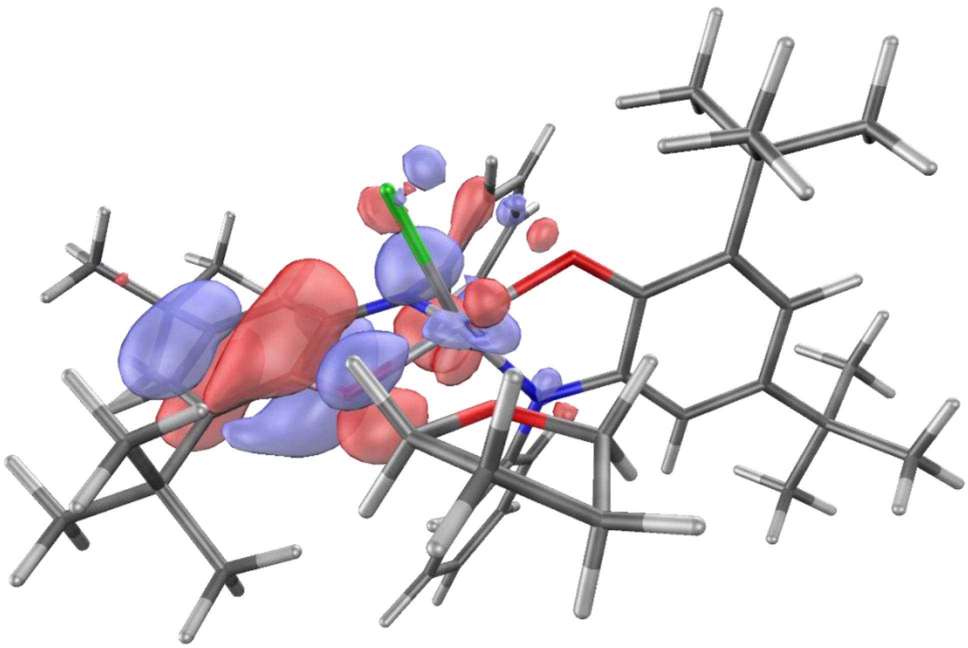

Figure S37. Calculated HOMOα of S = 0 [(^{Ph}ap)(^{Ph}isq)V^{IV}Cl] (**I**). Plot generated in IQmol, isosurface value 0.04.

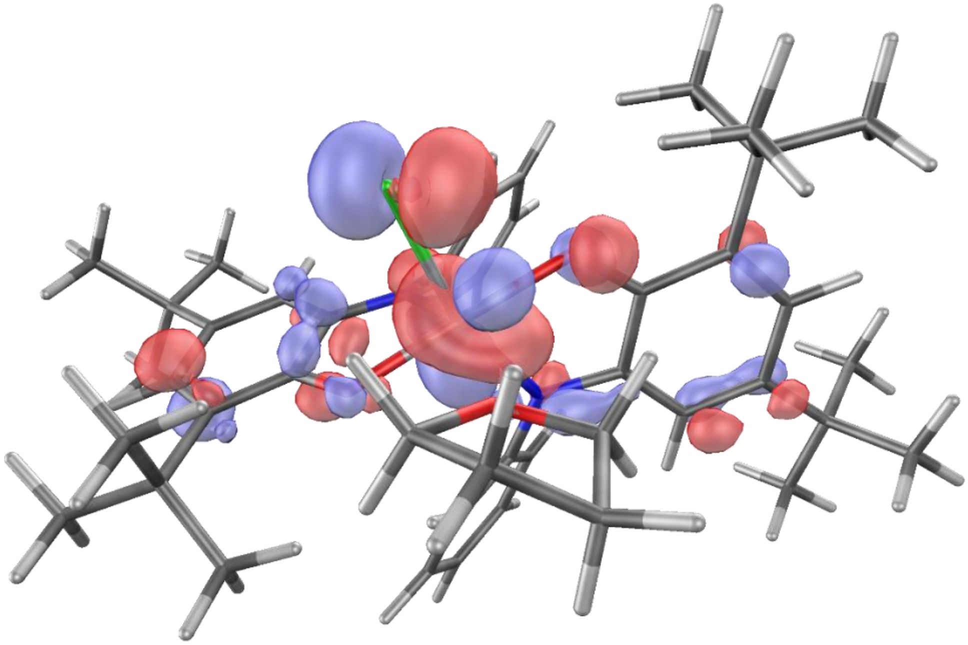

Figure S38. Calculated HOMOβ of S = 0 [(^{Ph}ap)(^{Ph}isq)V^{IV}Cl] (**I**). Plot generated in IQmol, isosurface value 0.04.

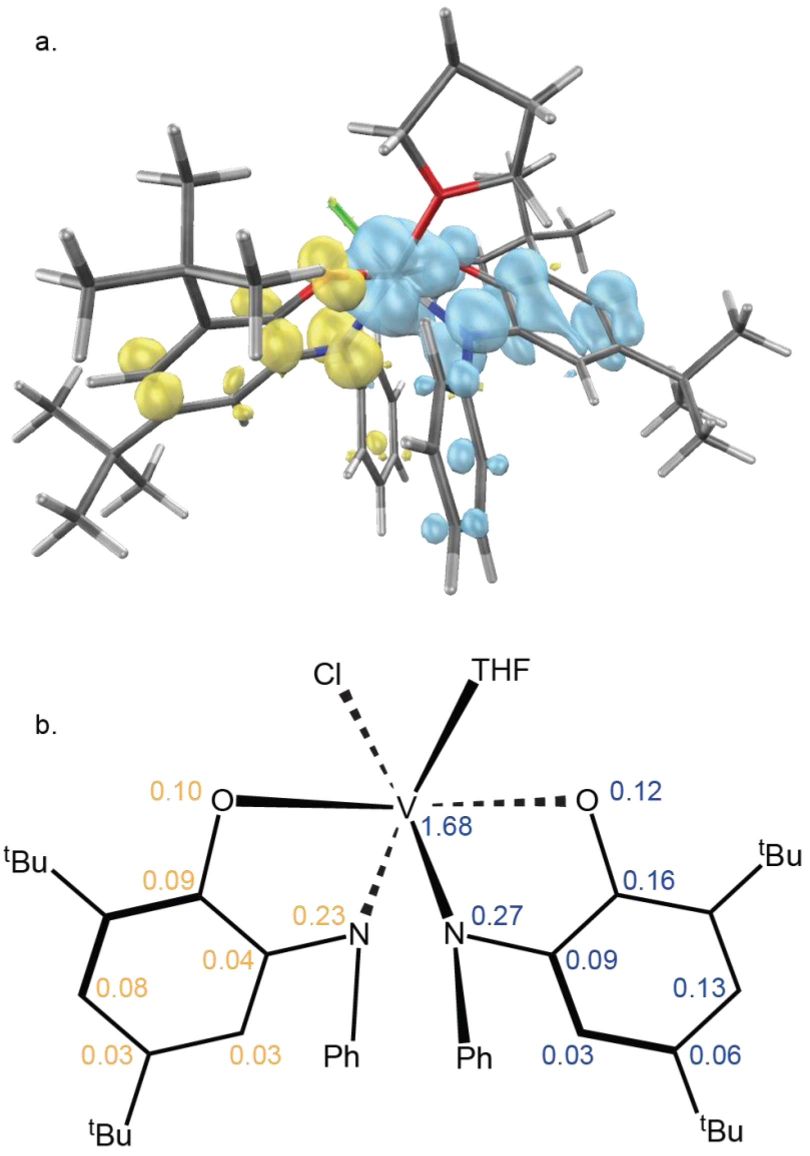

Figure S39. a. Spin density plot of S = 1 THF adduct of [(^{Ph}ap)(^{Ph}isq)V^{IV}Cl] (**I**) generated in IQmol (isosurface value 0.006). **b.** Löwdin spin population per atom (n > 0.02). Spin down density is shown in yellow.

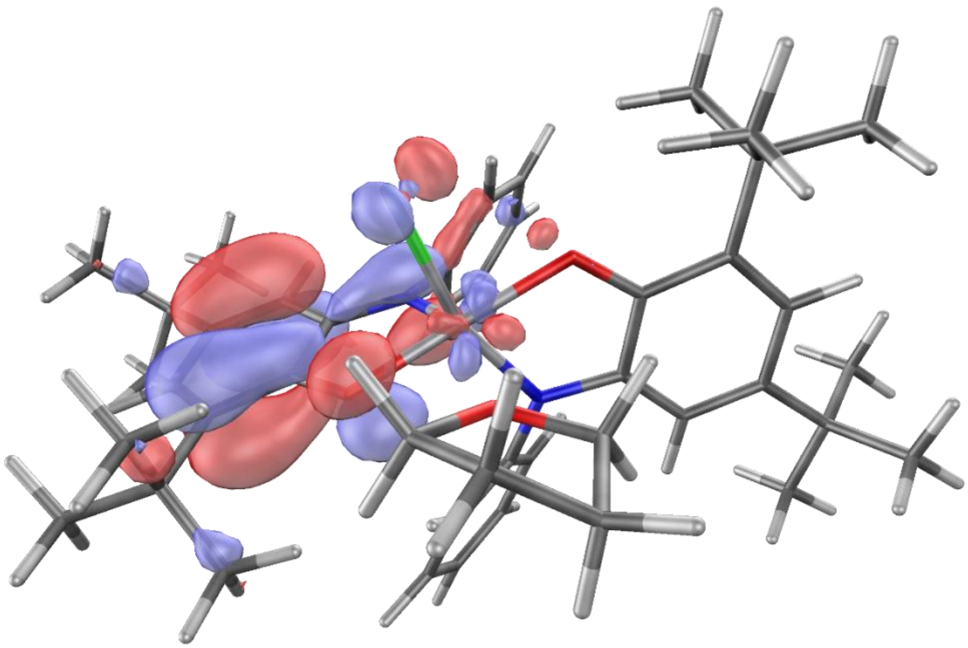

Figure S40. Calculated HOMO-2α of S = 1 [(^{Ph}ap)(^{Ph}isq)V^{IV}Cl] (**I**). Plot generated in IQmol, isosurface value 0.04.

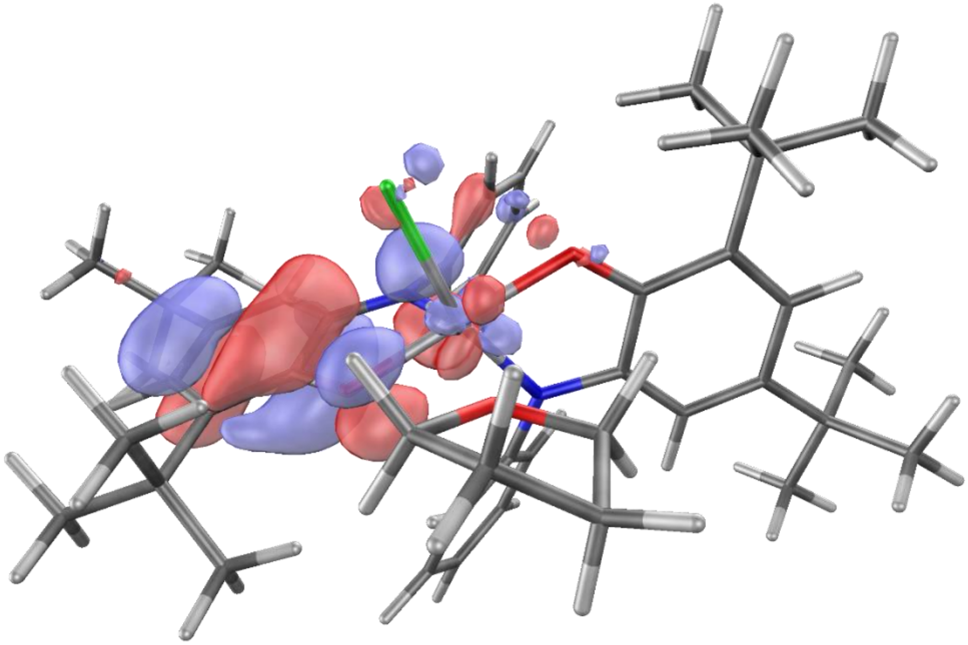

Figure S41. Calculated HOMO-2β of S = 1 [(^{Ph}ap)(^{Ph}isq)V^{IV}Cl] (**I**). Plot generated in IQmol, isosurface value 0.04.

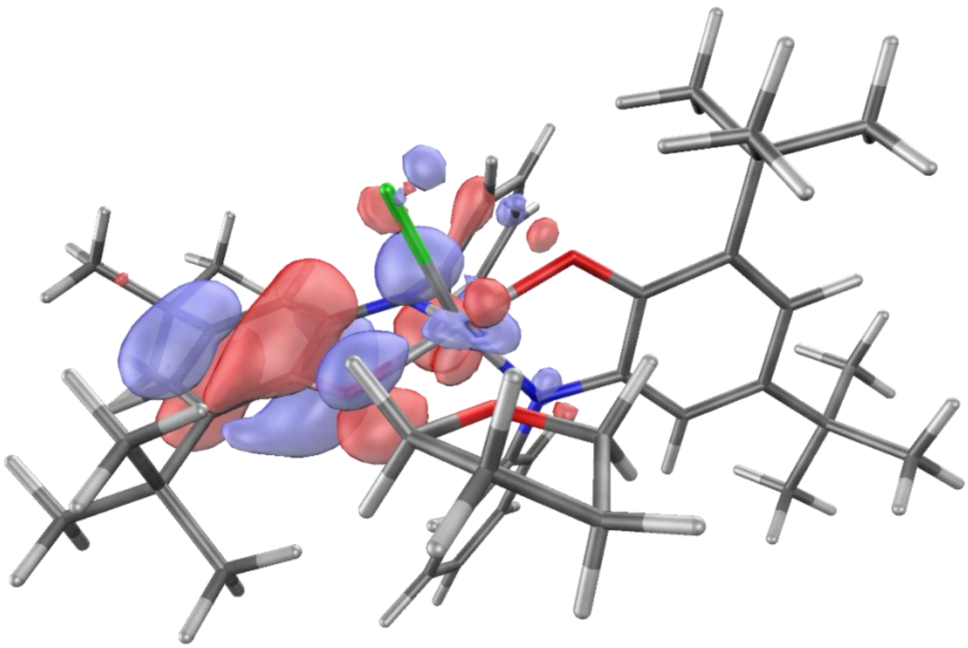

Figure S42: Calculated HOMO-1α of S = 1 [(^{Ph}ap)(^{Ph}isq)V^{IV}Cl] (**I**). Plot generated in IQmol, isosurface value 0.04.

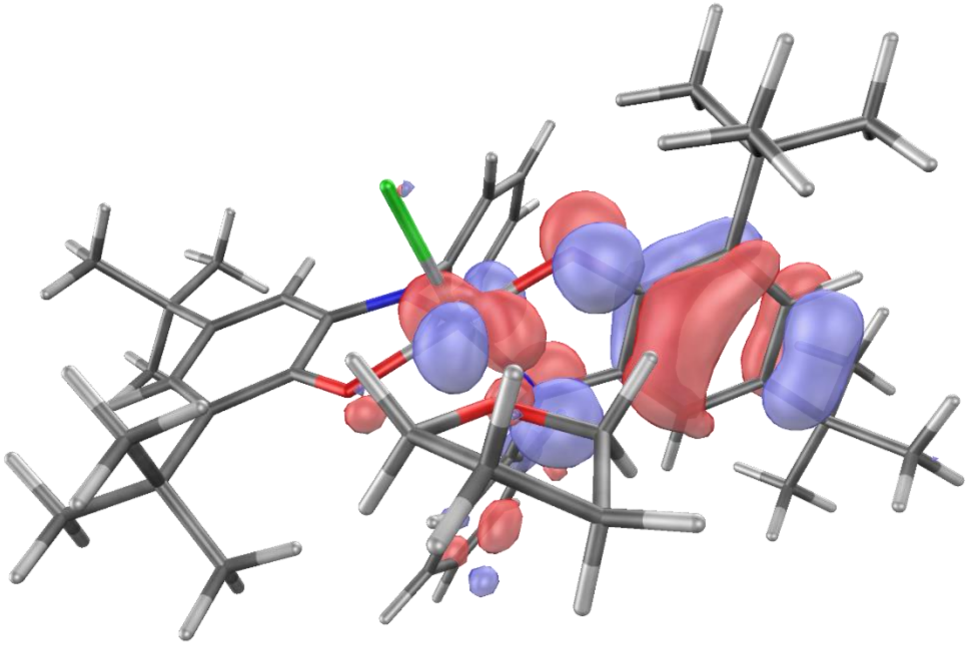

Figure S43. Calculated HOMOα of S = 1 [(^{Ph}ap)(^{Ph}isq)V^{IV}Cl] (**I**). Plot generated in IQmol, isosurface value 0.04.

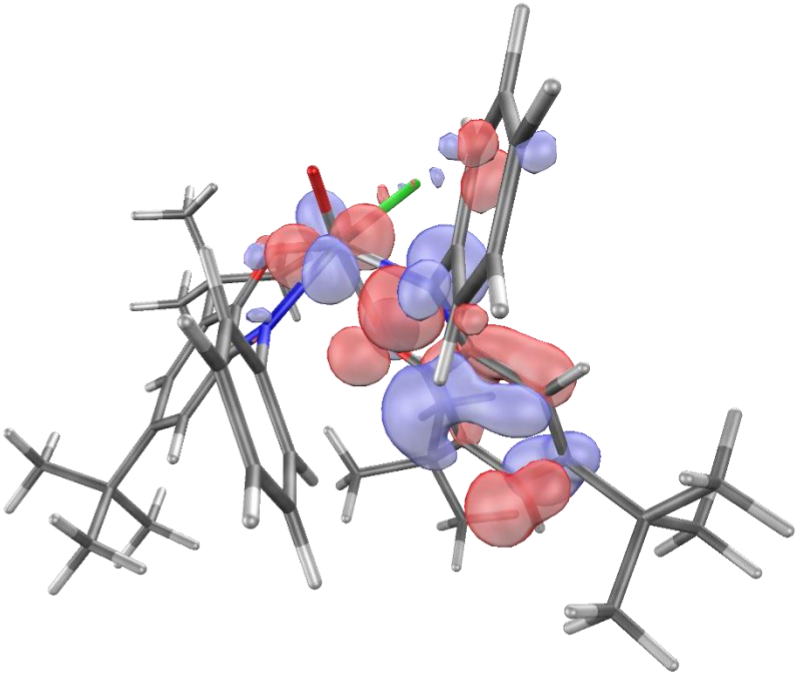

Figure S44: Calculated LUMO of S = 1 *cis*-[(^{Ph}isq)(^{Ph}ibq)V(O)Cl] (**IIb**). Plot generated in IQmol, isosurface value 0.04.

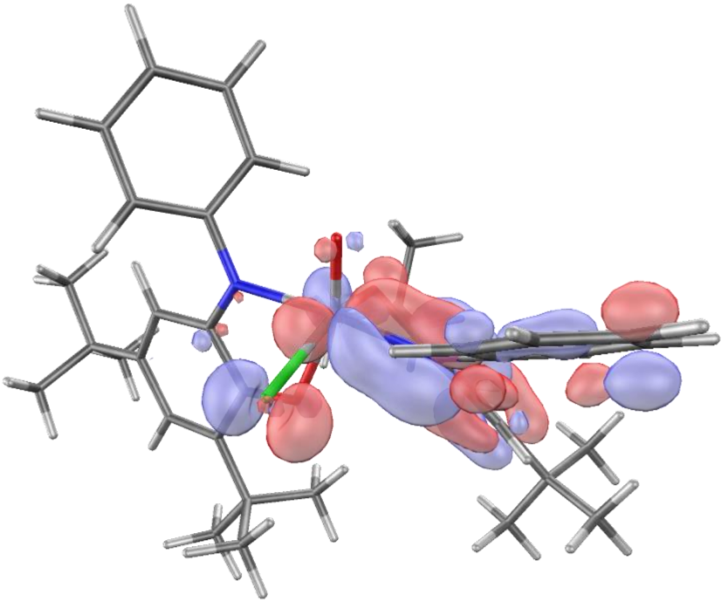

Figure S45: Calculated HOMO of S = 0 *trans*-[^{Ph}isq)(^{Ph}ibq)V(O)Cl] (**IIa**). Plot generated in IQmol, isosurface value 0.04.

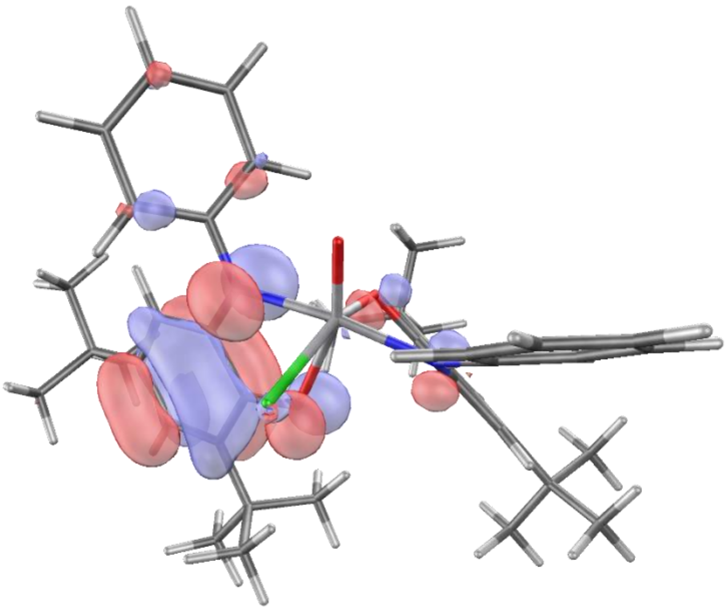

Figure S46: Calculated LUMO of S = 0 *trans*-[^{Ph}isq)(^{Ph}ibq)V(O)Cl] (**IIa**). Plot generated in IQmol, isosurface value 0.04.

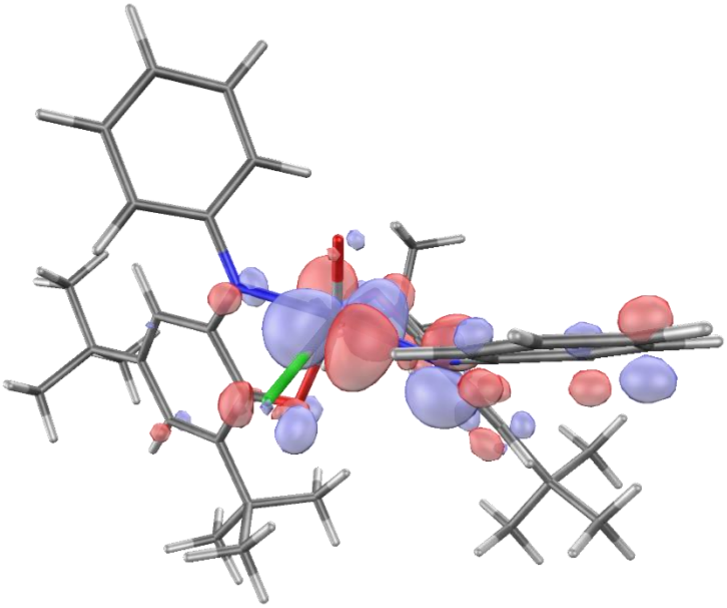

Figure S47: Calculated LUMO+1 of S = 0 *trans*-[^{Ph}isq)(^{Ph}ibq)V(O)Cl] (**IIa**). Plot generated in IQmol, isosurface value 0.04.

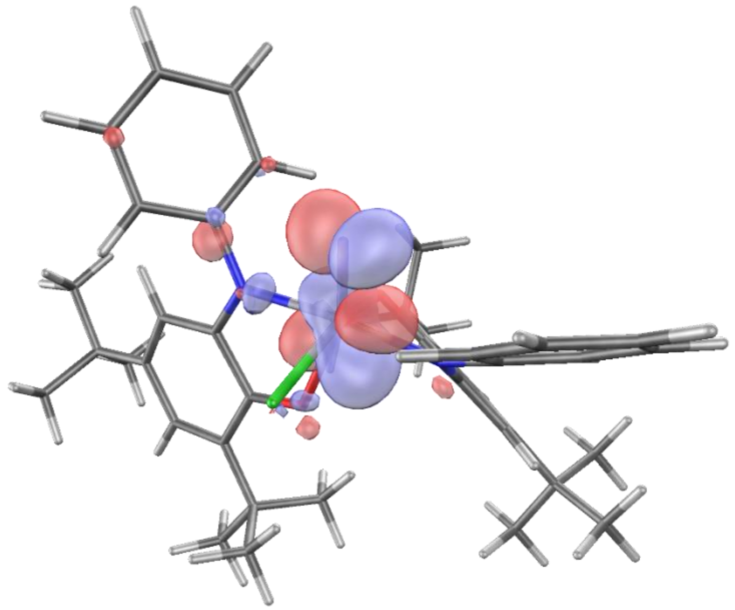

Figure S48: Calculated LUMO+2 of S = 0 *trans*-[^{Ph}isq)(^{Ph}ibq)V(O)Cl] (**IIa**). Plot generated in IQmol, isosurface value 0.04.

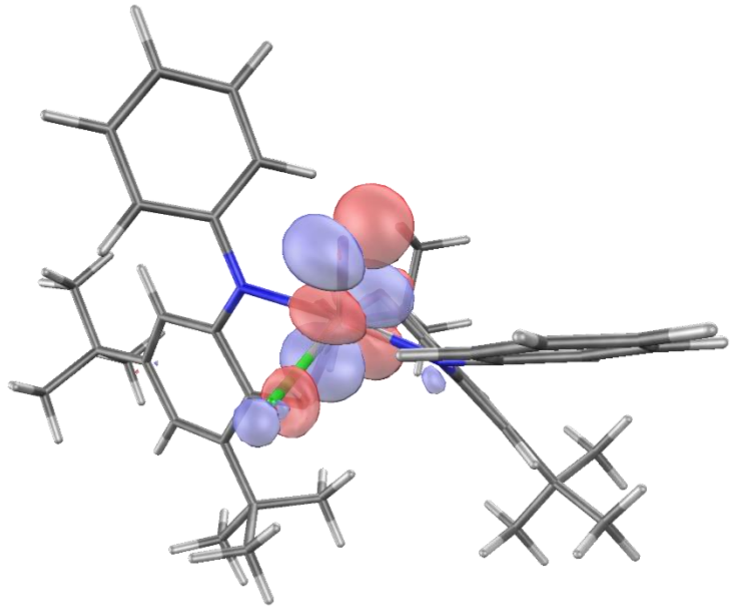

Figure S49: Calculated LUMO+3 of S = 0 *trans*-[^{Ph}isq)(^{Ph}ibq)V(O)Cl] (**IIa**). Plot generated in IQmol, isosurface value 0.04.

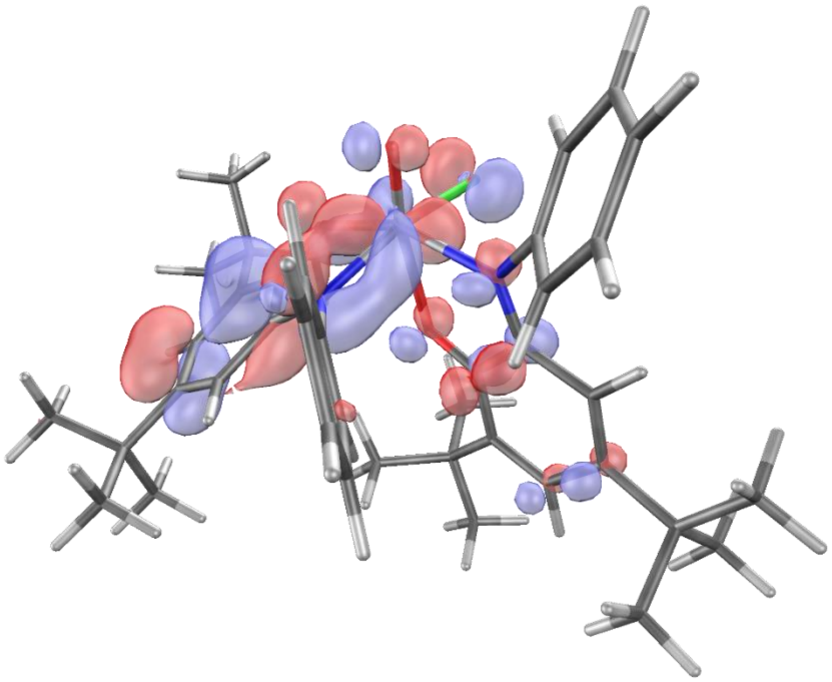

Figure S50: Calculated HOMO of S = 0 *cis*-[(^{Ph}isq)(^{Ph}ibq)V(O)Cl] (**IIb**). Plot generated in IQmol, isosurface value 0.04.

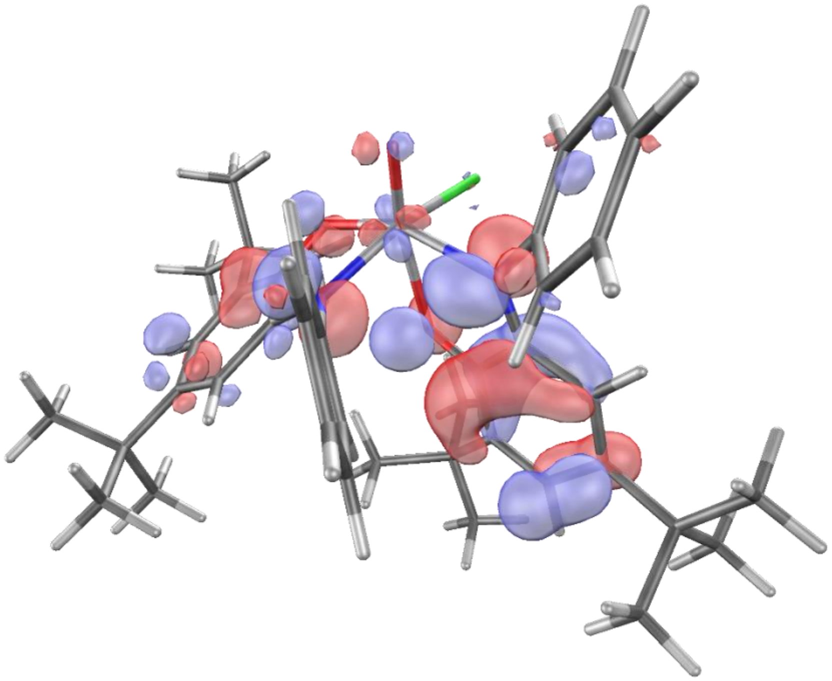

Figure S51: Calculated LUMO of S = 0 *cis*-[(^{Ph}isq)(^{Ph}ibq)V(O)Cl] (**IIb**). Plot generated in IQmol, isosurface value 0.04.

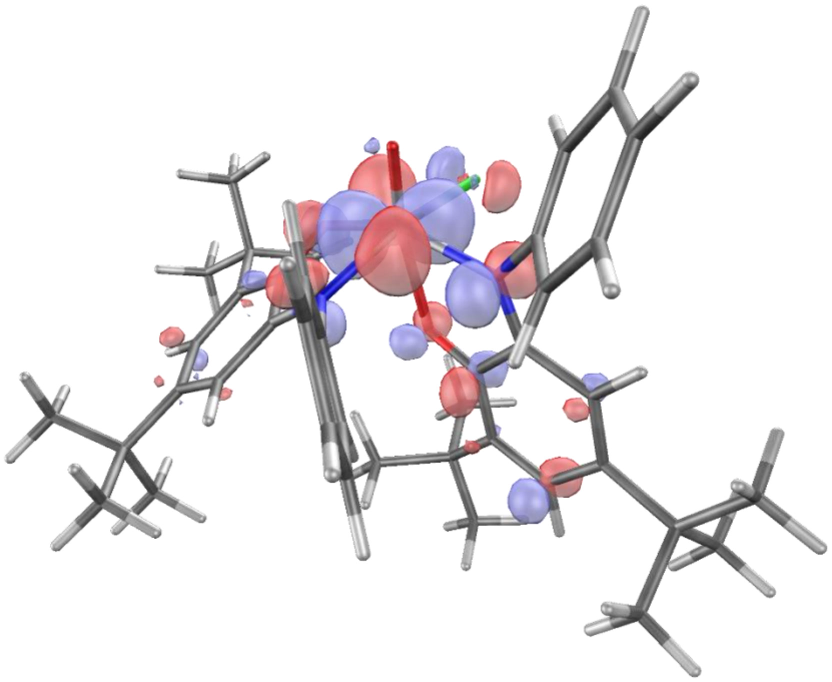

Figure S52: Calculated LUMO+1 of S = 0 *cis*-[(^{Ph}isq)(^{Ph}ibq)V(O)Cl] (**IIb**). Plot generated in IQmol, isosurface value 0.04.

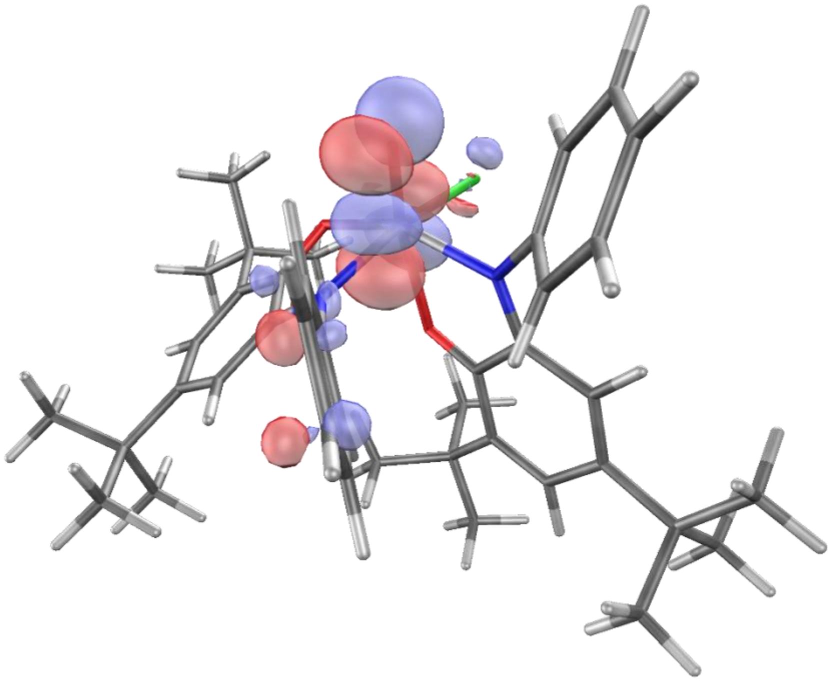

Figure S53: Calculated LUMO+2 of S = 0 *cis*-[(^{Ph}isq)(^{Ph}ibq)V(O)Cl] (**IIb**). Plot generated in IQmol, isosurface value 0.04.

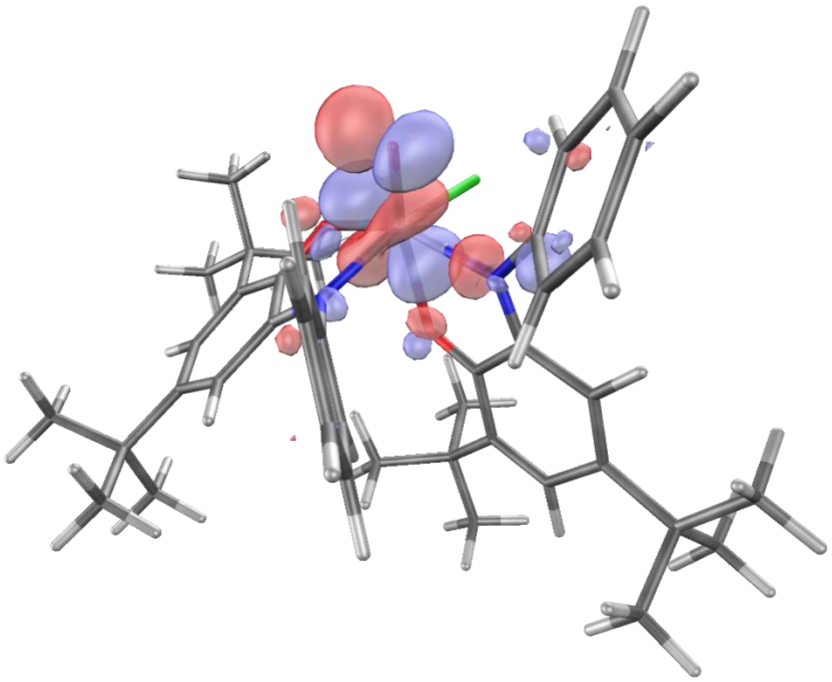

Figure S54: Calculated LUMO+3 of S = 0 *cis*-[(^{Ph}isq)(^{Ph}ibq)V(O)Cl] (**IIb**). Plot generated in IQmol, isosurface value 0.04.

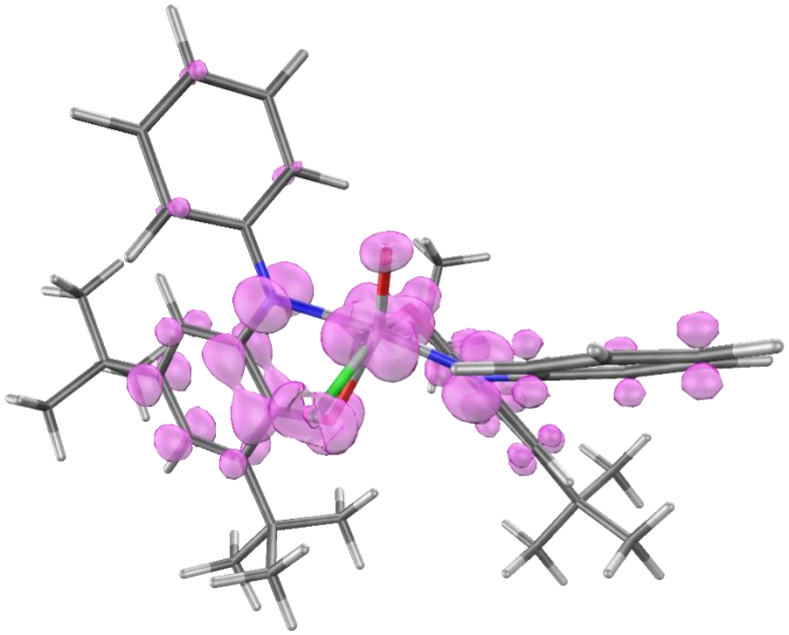

Figure S55: FOD of S = 0 *trans*-[^{Ph}isq)(^{Ph}ibq)V(O)Cl] (**IIa**). Plot generated in IQmol, isosurface value 0.005.

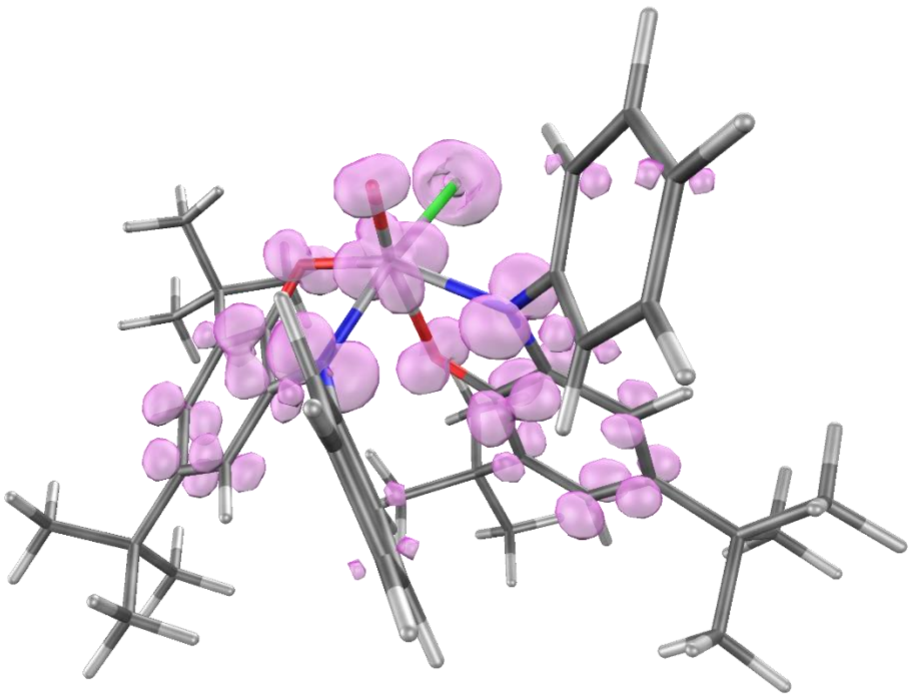

Figure S56: FOD of S = 1 *cis*-[(^{Ph}isq)(^{Ph}ibq)V(O)Cl] (**IIb**). Plot generated in IQmol, isosurface value 0.005.

DFT optimized coordinates

List S1 [(^{Ph}ap)(^{Ph}isq)VCl] (**I**), PBE0, S = 0

V	0.63432	11.49551	7.55454
Cl	-1.51321	12.21907	7.04037
O	0.64811	12.91632	8.86051
O	0.42468	9.95563	6.43871
N	2.46570	11.35473	8.33771
N	1.42159	12.16002	5.89885
C	2.74963	12.17199	9.40273
C	1.22736	11.36814	4.80413
C	1.67208	13.06025	9.65185
C	0.68052	10.10145	5.15353
C	1.73588	13.98406	10.73218
C	0.44802	9.10800	4.16807
C	2.89039	13.94441	11.50821
C	0.75020	9.47043	2.84588
C	3.96999	13.05373	11.27327
C	1.25963	10.74684	2.46385
C	3.89986	12.17606	10.20989
C	1.49380	11.68257	3.46919
C	3.48303	10.53854	7.76257
C	2.26819	13.29293	5.78788
C	3.16157	9.21539	7.42494
C	1.83729	14.53034	6.27804

C	4.11881	8.39885	6.83557
C	2.68074	15.63576	6.16850
C	5.40210	8.85722	6.60513
C	3.94617	15.50320	5.62263
C	5.71814	10.17210	6.91867
C	4.38268	14.28171	5.15370
C	4.77107	11.01489	7.47968
C	3.53982	13.17290	5.22641
C	0.58262	14.95603	10.99959
C	-0.15060	7.75670	4.55861
C	-0.72416	14.20127	11.23312
C	-1.55914	7.96896	5.12556
C	0.42961	15.89859	9.79975
C	0.74276	7.05964	5.57315
C	0.85030	15.81379	12.24876
C	-0.28951	6.83670	3.34644
C	5.18857	13.11690	12.20654
C	1.49691	11.05410	0.98153
C	5.74158	14.54684	12.26791
C	2.42490	9.98606	0.36121
C	4.75599	12.67903	13.61515
C	0.15136	11.04115	0.24225
C	6.31496	12.19425	11.74493
C	2.14242	12.42820	0.77521
H	2.96625	14.61444	12.35159
H	0.56963	8.74442	2.06481
H	4.69375	11.47471	10.00352
H	1.87279	12.66538	3.23132
H	2.16918	8.84535	7.62999
H	0.84630	14.61396	6.70237
H	3.84968	7.38287	6.57234
H	2.33625	16.59896	6.52445
H	6.14447	8.20857	6.15810
H	4.59215	16.37031	5.55175
H	6.70794	10.55809	6.70463
H	5.37179	14.17449	4.72616
H	5.01359	12.04859	7.68734
H	3.87248	12.20692	4.86901
H	-1.04445	13.66804	10.33915
H	-1.50967	14.90743	11.51399
H	-0.61818	13.48375	12.05187
H	-1.99112	7.01035	5.42548
H	-1.54620	8.63182	5.98818
H	-2.20630	8.41970	4.37110
H	-0.33544	16.64840	10.01310
H	1.36763	16.41702	9.58906
H	0.12500	15.34918	8.90933
H	0.32172	6.08666	5.83976
H	1.74065	6.89598	5.16218
H	0.83960	7.65079	6.47967

H	1.77437	16.38922	12.16131
H	0.02947	16.52129	12.37372
H	0.90072	15.19501	13.14815
H	0.67762	6.61899	2.88620
H	-0.94464	7.26860	2.58624
H	-0.73101	5.88938	3.66225
H	5.00089	15.25147	12.64683
H	6.60738	14.58591	12.93293
H	6.05841	14.88372	11.27887
H	2.63688	10.23101	-0.68207
H	3.37115	9.93440	0.90277
H	1.96638	8.99612	0.38867
H	5.60306	12.74519	14.30278
H	4.38176	11.65342	13.61538
H	3.96285	13.32348	14.00128
H	-0.54396	11.74419	0.70461
H	0.28860	11.33543	-0.80182
H	-0.30897	10.05144	0.26235
H	7.16630	12.29312	12.42126
H	6.00749	11.14652	11.75365
H	6.65836	12.44565	10.73910
H	3.10036	12.50567	1.29293
H	1.49595	13.23189	1.13225
H	2.32512	12.59328	-0.28828
O	-0.06024	10.11647	8.98739
C	0.22927	10.17939	10.40741
C	-0.48455	8.99843	10.99749
C	-1.70890	8.85981	10.10255
C	-1.12591	9.14657	8.74239
H	-0.67440	8.26651	8.28260
H	-1.81514	9.61190	8.04101
H	1.31139	10.15795	10.52943
H	-0.15236	11.13029	10.78356
H	0.13757	8.10258	10.92665
H	-0.73453	9.15653	12.04667
H	-2.45354	9.61632	10.35942
H	-2.17578	7.87715	10.15811

List S2 [(^{Ph}ap)(^{Ph}isq)VCl] (**I**), PBE0, S = 1

V	0.63437	11.49549	7.55472
Cl	-1.51312	12.21912	7.04048
O	0.64818	12.91632	8.86066
O	0.42469	9.95560	6.43891
N	2.46572	11.35465	8.33794
N	1.42170	12.15994	5.89904
C	2.74966	12.17192	9.40295
C	1.22747	11.36804	4.80432
C	1.67214	13.06022	9.65203
C	0.68057	10.10139	5.15374
C	1.73594	13.98405	10.73234

C	0.44806	9.10792	4.16829
C	2.89044	13.94437	11.50840
C	0.75028	9.47033	2.84610
C	3.97001	13.05364	11.27350
C	1.25978	10.74670	2.46405
C	3.89987	12.17595	10.21014
C	1.49395	11.68245	3.46939
C	3.48303	10.53842	7.76283
C	2.26835	13.29282	5.78806
C	3.16153	9.21527	7.42522
C	1.83748	14.53025	6.27819
C	4.11875	8.39868	6.83588
C	2.68099	15.63564	6.16865
C	5.40206	8.85699	6.60547
C	3.94642	15.50302	5.62282
C	5.71815	10.17187	6.91900
C	4.38290	14.28150	5.15392
C	4.77110	11.01471	7.47996
C	3.53999	13.17272	5.22663
C	0.58271	14.95607	10.99970
C	-0.15062	7.75666	4.55884
C	-0.72410	14.20137	11.23322
C	-1.55917	7.96898	5.12574
C	0.42978	15.89861	9.79985
C	0.74268	7.05958	5.57340
C	0.85040	15.81384	12.24887
C	-0.28954	6.83665	3.34668
C	5.18857	13.11679	12.20680
C	1.49710	11.05393	0.98173
C	5.74163	14.54670	12.26816
C	2.42507	9.98584	0.36146
C	4.75594	12.67895	13.61541
C	0.15157	11.04102	0.24242
C	6.31493	12.19408	11.74524
C	2.14267	12.42801	0.77541
H	2.96524	14.61318	12.35301
H	0.57029	8.74453	2.06462
H	4.69343	11.47468	10.00323
H	1.87458	12.66466	3.23125
H	2.16705	8.84731	7.62420
H	0.84493	14.61344	6.69919
H	3.84858	7.38335	6.57150
H	2.33619	16.59947	6.52294
H	6.14380	8.20926	6.15596
H	4.59181	16.37051	5.55033
H	6.70709	10.55830	6.70200
H	5.37160	14.17534	4.72486
H	5.01205	12.05013	7.68152
H	3.87122	12.20718	4.86661
H	-1.04139	13.66559	10.33970

H	-1.50947	14.90911	11.50985
H	-0.62019	13.48654	12.05461
H	-1.99048	7.01094	5.42877
H	-1.54528	8.63504	5.98592
H	-2.20674	8.41734	4.37022
H	-0.34224	16.64180	10.01050
H	1.36456	16.42532	9.59540
H	0.13433	15.34725	8.90770
H	0.32155	6.08690	5.84128
H	1.74020	6.89520	5.16173
H	0.83885	7.65295	6.47858
H	1.77414	16.39028	12.16239
H	0.02956	16.52132	12.37274
H	0.89928	15.19595	13.14914
H	0.67757	6.61888	2.88639
H	-0.94423	7.26904	2.58641
H	-0.73151	5.88929	3.66209
H	5.00246	15.25289	12.64749
H	6.60639	14.58381	12.93435
H	6.06070	14.88364	11.27994
H	2.63719	10.23016	-0.68207
H	3.37124	9.93447	0.90318
H	1.96660	8.99593	0.38950
H	5.60364	12.74694	14.30180
H	4.38346	11.65281	13.61663
H	3.96297	13.32273	14.00335
H	-0.54338	11.74436	0.70487
H	0.28826	11.33478	-0.80199
H	-0.30902	10.05148	0.26333
H	7.16585	12.29352	12.42177
H	6.00783	11.14632	11.75448
H	6.65856	12.44554	10.73957
H	3.10069	12.50512	1.29301
H	1.49642	13.23154	1.13315
H	2.32502	12.59357	-0.28814
O	-0.06028	10.11650	8.98758
C	0.22919	10.17944	10.40760
C	-0.48469	8.99851	10.99768
C	-1.70902	8.85992	10.10271
C	-1.12598	9.14663	8.74256
H	-0.67497	8.26649	8.28258
H	-1.81417	9.61315	8.04095
H	1.31131	10.15410	10.53084
H	-0.15238	11.13010	10.78476
H	0.13754	8.10278	10.92491
H	-0.73455	9.15343	12.04745
H	-2.45358	9.61656	10.35963
H	-2.17609	7.87736	10.15853

List S3 [(^{Ph}ap)(^{Ph}isq)VCl] (**I**), PBE0, S = 2

V	0.63432	11.49561	7.55449
Cl	-1.51320	12.21920	7.04033
O	0.64814	12.91643	8.86045
O	0.42465	9.95573	6.43867
N	2.46570	11.35482	8.33765
N	1.42158	12.16010	5.89879
C	2.74965	12.17208	9.40266
C	1.22734	11.36821	4.80407
C	1.67211	13.06035	9.65178
C	0.68049	10.10154	5.15349
C	1.73593	13.98418	10.73210
C	0.44797	9.10807	4.16804
C	2.89045	13.94451	11.50813
C	0.75014	9.47050	2.84585
C	3.97003	13.05381	11.27318
C	1.25959	10.74689	2.46380
C	3.89988	12.17614	10.20981
C	1.49377	11.68264	3.46914
C	3.48301	10.53861	7.76250
C	2.26820	13.29300	5.78780
C	3.16154	9.21546	7.42489
C	1.83731	14.53042	6.27796
C	4.11876	8.39891	6.83552
C	2.68078	15.63583	6.16840
C	5.40205	8.85725	6.60507
C	3.94621	15.50325	5.62252
C	5.71812	10.17213	6.91860
C	4.38270	14.28174	5.15361
C	4.77106	11.01494	7.47960
C	3.53982	13.17295	5.22633
C	0.58268	14.95616	10.99951
C	-0.15066	7.75679	4.55860
C	-0.72411	14.20142	11.23306
C	-1.55920	7.96907	5.12555
C	0.42968	15.89871	9.79967
C	0.74269	7.05973	5.57313
C	0.85038	15.81392	12.24867
C	-0.28959	6.83679	3.34644
C	5.18862	13.11699	12.20645
C	1.49686	11.05414	0.98147
C	5.74164	14.54691	12.26780
C	2.42484	9.98608	0.36116
C	4.75605	12.67913	13.61506
C	0.15130	11.04120	0.24220
C	6.31499	12.19431	11.74484
C	2.14239	12.42824	0.77514
H	2.96499	14.61223	12.35370
H	0.57042	8.74353	2.06530

H	4.69428	11.47587	10.00236
H	1.87281	12.66517	3.23120
H	2.16692	8.84970	7.62843
H	0.84606	14.61036	6.70299
H	3.84995	7.38239	6.57357
H	2.33580	16.59891	6.52414
H	6.14440	8.20898	6.15721
H	4.59280	16.36998	5.55243
H	6.70781	10.55779	6.70307
H	5.37266	14.17469	4.72780
H	5.01236	12.04973	7.68416
H	3.87484	12.20571	4.87392
H	-1.04059	13.66611	10.33888
H	-1.50975	14.90876	11.51037
H	-0.61957	13.48578	12.05363
H	-1.98698	7.01063	5.43117
H	-1.54641	8.63679	5.98443
H	-2.20898	8.41329	4.36937
H	-0.34195	16.64241	10.01034
H	1.36485	16.42499	9.59534
H	0.13358	15.34611	8.90853
H	0.31957	6.08773	5.83917
H	1.74029	6.89363	5.16221
H	0.83830	7.65246	6.47851
H	1.77452	16.38984	12.16247
H	0.02971	16.52171	12.37261
H	0.89908	15.19578	13.14878
H	0.67728	6.61742	2.88587
H	-0.94577	7.26736	2.58603
H	-0.73115	5.89005	3.66318
H	5.00182	15.25294	12.64609
H	6.60622	14.58494	12.93436
H	6.06093	14.88336	11.27943
H	2.63390	10.23247	-0.68213
H	3.37265	9.93555	0.90001
H	1.96801	8.99522	0.38722
H	5.60354	12.74724	14.30184
H	4.38355	11.65299	13.61591
H	3.96270	13.32272	14.00245
H	-0.54318	11.74727	0.70099
H	0.29172	11.33227	-0.80210
H	-0.31166	10.05259	0.26238
H	7.16603	12.29340	12.42142
H	6.00749	11.14669	11.75360
H	6.65841	12.44594	10.73913
H	3.10066	12.50544	1.29208
H	1.49606	13.23195	1.13213
H	2.32414	12.59279	-0.28842
O	-0.06025	10.11659	8.98736
C	0.22926	10.17952	10.40737

C	-0.48457	8.99857	10.99747
C	-1.70891	8.85996	10.10253
C	-1.12593	9.14670	8.74236
H	-0.67601	8.26480	8.28345
H	-1.81427	9.61467	8.04143
H	1.31126	10.15364	10.53265
H	-0.15217	11.12986	10.78605
H	0.13768	8.10274	10.92406
H	-0.73443	9.15247	12.04750
H	-2.45252	9.61774	10.35913
H	-2.17727	7.87793	10.16021

List S4 [(^{Ph}ap)(^{Ph}isq)VCl] (**I**), PBE0, CPCM (benzene), S = 0

V	0.13025647768896	11.33577156980464	7.76885660414326
Cl	-2.06997826934365	12.04534400030727	7.49168759010909
O	0.35652035528877	12.66929216670356	9.16383579675633
O	-0.11305113422798	9.94660572227554	6.39490267719012
N	2.13714034507704	11.06188992047873	8.36120907524009
N	0.96755068564529	12.21014684532152	6.16038233067009
C	2.56723307717239	11.96817284530932	9.26345881732581
C	1.08239971994767	11.42092409399059	5.07830525074719
C	1.52404963259619	12.86069874875502	9.68842597155190
C	0.40400168686160	10.16661268191265	5.22666402342995
C	1.78842726196237	13.86306503542612	10.65347969464969
C	0.32193833464701	9.28608440049933	4.11647000799036
C	3.08511586170515	13.96445846378509	11.09910183886149
C	1.05642738699831	9.62873365441826	2.99967704698157
C	4.13778842268064	13.10613452191289	10.69936586207122
C	1.83226358595687	10.80966640640577	2.87898743919677
C	3.86054471019336	12.08902775590519	9.82091360200147
C	1.79443625193622	11.72012796365081	3.90163004152234
C	2.99555160678156	10.16652941758159	7.71938034135283
C	1.61283736133779	13.46005552657530	6.21903073039438
C	2.52984521479747	8.87646268940631	7.44725220369837
C	0.85238249687171	14.61421115726201	6.37886991778844
C	3.30979438566028	7.97656772297883	6.74455847228756
C	1.48137427146453	15.84566291560548	6.48013420425415
C	4.56825008936731	8.33928460139389	6.28798844403152
C	2.86587944745901	15.93502431679073	6.45087455543989
C	5.04130106674266	9.61700002207377	6.54932057709372
C	3.62513937751456	14.78304656114973	6.30099507168476
C	4.27100960165924	10.51894731289017	7.26102962248639
C	3.00222511261288	13.55353490347536	6.17272813738801
C	0.67765937256154	14.76235427130526	11.17678521237706
C	-0.56088929442384	8.04343570307171	4.15780393348358
C	-0.40256535428271	13.89644012275296	11.83462861435467
C	-2.00695744689466	8.45918097448256	4.46241412834743
C	0.06754338151850	15.57680966169044	10.03424235009285
C	-0.06034155395830	7.06794167807277	5.22738899525630
C	1.19590730514402	15.73311909584482	12.23225978638529

C	-0.58431039652211	7.30857664537292	2.81972791461189
C	5.55122188107372	13.43328104633705	11.16542791535100
C	2.70727925908838	11.00910978150596	1.65004689968561
C	6.00496008884500	14.64668266953902	10.34288554643284
C	3.72865216097462	9.86793955598615	1.59254856980296
C	5.58281568890035	13.79730118390066	12.65206486583118
C	1.87148337035804	10.99565688291746	0.36882722163055
C	6.53374919754357	12.28793751410120	10.94529081618799
C	3.46206095137935	12.33188549315310	1.69872110101826
H	3.31783888398043	14.74106949733168	11.81132951899406
H	1.04055810098960	8.95729862825148	2.15273537352287
H	4.62245581997223	11.37763513849229	9.54430573465733
H	2.31332679531177	12.66423289176312	3.83978646946155
H	1.54094569114494	8.59466295062903	7.78005516769478
H	-0.22662463790762	14.53044098098718	6.42005414057441
H	2.92467328555258	6.98497556440192	6.54333444215078
H	0.88317741152622	16.74157411255347	6.58942200248657
H	5.17381692634809	7.63414917305905	5.73372263203076
H	3.35216837843582	16.89829521932930	6.54406100967489
H	6.01683565889801	9.92062191868165	6.18954622038881
H	4.70716511998241	14.84189340592103	6.27868461394048
H	4.64603712587112	11.51687225253282	7.43645897371824
H	3.58085522126888	12.65110435972630	6.04002305113307
H	-0.86745211875745	13.22813887792819	11.11124037815747
H	-1.18039415028672	14.53405616392331	12.26137361402476
H	0.02925618015734	13.30273328754820	12.64465847082102
H	-2.65325034965828	7.57841716981140	4.43228000395135
H	-2.09610974982312	8.93638504475215	5.43780544635295
H	-2.36585966091103	9.16380408439372	3.70976341820362
H	-0.71421756531703	16.23304168353708	10.42536942241902
H	0.83032432969681	16.19887885687124	9.56016068977266
H	-0.37081466072500	14.93231087474177	9.27516260093270
H	-0.70776958891178	6.18839815009880	5.26090893173027
H	0.95365807038410	6.73334623173311	4.99948808870996
H	-0.05558677017043	7.53297588083737	6.21203088152238
H	1.95019842219314	16.41330932609873	11.82907997099472
H	0.36529505869231	16.34093909683427	12.59602926466591
H	1.62320699374879	15.20755782247240	13.08960521715742
H	0.40194729423267	6.94066008506408	2.52765859298348
H	-0.97302822646187	7.94281071030639	2.01919102010832
H	-1.24350165875705	6.44257584017252	2.90423452676702
H	5.33582861760749	15.49445280150413	10.50157023769652
H	7.01603585601925	14.95149773218678	10.62524307026614
H	5.99571365218771	14.40757045906928	9.27726187638212
H	4.39771604462887	10.00421357377809	0.73928384854519
H	4.33562407716748	9.84238313442172	2.49975825285474
H	3.24248673204842	8.89667179107771	1.48427955986837
H	6.60449054927582	14.04132033689778	12.95209844337474
H	5.23067969784149	12.96615201346450	13.26672768007025
H	4.97006469580491	14.66781761464383	12.88300802316878

H	1.12024637578805	11.78760894022330	0.38645081481259
H	2.52205872144227	11.16256311910468	-0.49392934158313
H	1.35665906111114	10.04400485460322	0.22589526218248
H	7.53109265898067	12.60713526467735	11.25403581767919
H	6.26106034289838	11.41183353071502	11.53799871225247
H	6.60023700329647	11.98370459696933	9.89937086522622
H	4.08908621204134	12.41351657655883	2.58945877667589
H	2.77633697548302	13.18185882571569	1.67878137450281
H	4.11371531749670	12.41034354541107	0.82632886596666
O	-0.35158512690319	9.88389095945705	9.20146127421176
C	0.23335153566230	9.83117562963867	10.52022238253917
C	-0.36880399464937	8.59861283329958	11.15179789561395
C	-1.77168939222059	8.60698106431642	10.56023924794100
C	-1.51328345694689	9.02477040232509	9.12546839208134
H	-1.25606013729852	8.17972589833824	8.48299806571998
H	-2.31340844212202	9.60026198433417	8.66267920916516
H	1.31445905234600	9.79650049911696	10.40762716788455
H	-0.04387326097343	10.74488220312463	11.05065021682844
H	0.17556492402927	7.70625666712617	10.83406240710026
H	-0.35116899565349	8.64338945828396	12.24016698284776
H	-2.38851008760944	9.35449465181822	11.06395046955169
H	-2.27653987584194	7.64423753292611	10.62808443967590

List S5 [(^{Ph}ap)(^{Ph}isq)VCl] (**I**), PBE0, CPCM (benzene), S = 1

V	-0.01042879539518	11.35499264408875	7.80505747450524
Cl	-2.22606311976296	12.08978533798562	7.59474104437547
O	0.28722878001109	12.63843576327089	9.22850676183980
O	-0.28742424080200	10.02141057730915	6.38252965829373
N	2.09477337453014	11.01891289118714	8.35920675734034
N	0.92305232691583	12.22518166445398	6.23369354832896
C	2.54561811645565	12.00536642793705	9.15549305719654
C	1.11555632144544	11.41156202468466	5.18029605092732
C	1.49221659724205	12.85776490455834	9.65726668355221
C	0.35971688187498	10.19444628695903	5.27620095791010
C	1.78274350303315	13.86708214605934	10.60780082669903
C	0.33240372655984	9.30199859840136	4.17211566928755
C	3.10519826398680	14.05763261051476	10.93424871001948
C	1.19605020561752	9.58188878987329	3.13603558726025
C	4.17100551236595	13.28852098443019	10.40883010304436
C	2.03596505411486	10.72287874537426	3.07228058406407
C	3.87912180317012	12.24556061855993	9.57094558493656
C	1.94701109758552	11.65614572364547	4.07065895345660
C	2.90114901938855	10.11223908266038	7.68300761780541
C	1.57854965614755	13.46455564293617	6.34142936359442
C	2.36000239137741	8.84533724231702	7.41512679612136
C	0.81574291178159	14.61253164614542	6.54152974631159
C	3.07386705717266	7.89942524258136	6.70553701709494
C	1.44159861603185	15.83923151792288	6.69836620327090

C	4.34553112523778	8.18536320328075	6.22788215630837
C	2.82735896274024	15.93009026923970	6.69523013224825
C	4.88864903083958	9.43973393419385	6.46580754133210
C	3.58956317362250	14.78501555859967	6.51442986354106
C	4.18432370997681	10.38901437808542	7.18370458551045
C	2.96928330738987	13.56200952464989	6.32443345404427
C	0.66791866265078	14.68909024240384	11.24083770845626
C	-0.65537500072231	8.14273448380141	4.11863964926826
C	-0.30322648637188	13.75495947097651	11.97108098469505
C	-2.07913548264612	8.69735626532530	4.25414550365758
C	-0.08086216096377	15.48555514404947	10.17060671433651
C	-0.40081197349407	7.13564570354707	5.24267687613299
C	1.21244645029261	15.67805487603204	12.26634509778539
C	-0.58633634683741	7.40160203571698	2.78675454700760
C	5.59087418774355	13.67591869740961	10.79295489940733
C	3.00630299512169	10.86021093682232	1.90930511571772
C	5.81760500894709	15.13874302098992	10.38965097787816
C	3.97540406205945	9.67274464490029	1.93641593605679
C	5.76956753511167	13.54876614572737	12.30701007512947
C	2.25632263919328	10.85415630257641	0.57537176746651
C	6.63620434728943	12.81599178765342	10.09195234655374
C	3.80849182807905	12.15268697980838	1.99160629536400
H	3.35273306049518	14.83399729378217	11.64466718782478
H	1.22727993520772	8.90041626290030	2.29792735326706
H	4.66485090180659	11.59011581864393	9.23587052340911
H	2.50395834299752	12.57908272206164	4.03413604292353
H	1.36065993644537	8.62407916748451	7.76273241408206
H	-0.26368195834556	14.52605568615994	6.56985563912932
H	2.62929842890405	6.93088637192674	6.51351514087753
H	0.84091927971168	16.72978793959898	6.83522236356143
H	4.90106065457939	7.44401885295577	5.66779247936361
H	3.31223498139293	16.88826802265880	6.83824845677788
H	5.87030526179027	9.68953419773565	6.08183988540736
H	4.67196283533347	14.84133873276572	6.52193856131784
H	4.62481089345219	11.36376515864948	7.32143911104158
H	3.55556924927318	12.66833863052088	6.17499553439371
H	-0.77814459407881	13.06046708303097	11.27998158526833
H	-1.08390546850273	14.34313741507951	12.45939845698733
H	0.22662649647270	13.18558906266134	12.73953091740344
H	-2.79942736134625	7.88362780863629	4.13502881615038
H	-2.23401378593891	9.17221196554961	5.22252796641362
H	-2.27834246234847	9.43790758510906	3.47666609713608
H	-0.86005188681346	16.08894122284188	10.64278100071245
H	0.60703930640266	16.15604334280717	9.64979237321434
H	-0.54466174744476	14.82850089711985	9.43800744304441
H	-1.11838208333258	6.31452643144484	5.16715178703855

H	0.60406565164200	6.71449865713840	5.17235640189049
H	-0.51242492686826	7.60557769812450	6.21873946884150
H	1.89427270700938	16.40163326345231	11.81154340427872
H	0.37924306902882	16.23418908144543	12.70100498869542
H	1.73683231443001	15.17147147646227	13.08074351964309
H	0.39137733160448	6.94633639184612	2.61168560967971
H	-0.81718437753571	8.06483105712038	1.94943299386183
H	-1.32679633815754	6.59951169398438	2.78932803235517
H	5.12261544605030	15.80585053732926	10.89939239551217
H	6.83176326477200	15.44742953454817	10.65701254637775
H	5.67934803111789	15.27381638658273	9.31429185738554
H	4.73251270620211	9.78965885752266	1.15660507631068
H	4.48418253409669	9.60382991168690	2.90044850090846
H	3.45613700732376	8.72898206389495	1.75972523802574
H	6.78173118287012	13.84691628007639	12.59443491444310
H	5.59790295930945	12.52126590511731	12.63551714723525
H	5.07469186909924	14.19359996428147	12.84606347394626
H	1.54276763375938	11.67917669134577	0.52590403091719
H	2.96950642087048	10.96838708532048	-0.24573802579465
H	1.70989604964595	9.92230652981172	0.41969777136652
H	7.63487942742856	13.15372537274816	10.37577067658573
H	6.55248931097423	11.76244042518145	10.36945329059787
H	6.55569270441315	12.89687709573958	9.00506407524728
H	4.38042205208808	12.22148480842039	2.92015893001268
H	3.15724443798782	13.02698615165814	1.91962271273142
H	4.51665848433950	12.19331069932333	1.16149738708791
O	-0.45177122912689	9.87221650577335	9.19302178762331
C	0.16500832430484	9.76744514478694	10.49729000203800
C	-0.38973395022589	8.48560257372377	11.06874429018956
C	-1.81385443691925	8.51000598022000	10.53072418590622
C	-1.60891166910967	9.00329051495298	9.11217446232484
H	-1.36155070179527	8.19277384277112	8.42417974697070
H	-2.42995788447733	9.58988509890406	8.70355044203511
H	1.24354359185959	9.77613066841227	10.36000307755313
H	-0.13338368995692	10.63980752718787	11.08250690373617
H	0.15426781117092	7.62671095249498	10.66899859342264
H	-0.32951250341715	8.45995508853517	12.15637385481857
H	-2.41701799007085	9.22547664307232	11.09387390169143
H	-2.30676547796030	7.53936744722701	10.56317821166492

List S6 [(^{Ph}ap)(^{Ph}isq)VCl] (**I**), PBE0, CPCM (benzene), S = 2

V	-0.11562672881057	11.32424081416585	7.79276465552983
Cl	-2.30272147077940	12.03941977701149	7.47550059000028
O	0.24411284496450	12.59433703961328	9.26881888124919
O	-0.17206548980502	9.95952498133240	6.38635987954202
N	1.94051084875843	10.92855257730878	8.35843417015232

N	0.81775665512766	12.34244902693911	6.21315011220325
C	2.47366905375254	11.93224933788353	9.06214287532265
C	1.11218194584068	11.51848885238169	5.20772954492230
C	1.46345203142902	12.82407512972693	9.60447742611196
C	0.47473694453278	10.21932788862866	5.29806901625876
C	1.83608838875760	13.84044370912637	10.52429575540698
C	0.55101297721719	9.33048383585282	4.19015439181493
C	3.17708680448631	14.00478408668785	10.75882920391195
C	1.40181022000972	9.68585524331310	3.16887501352895
C	4.19805199717644	13.21323612242801	10.16658733591178
C	2.14196178818051	10.90211993561674	3.11784383528048
C	3.83977997387324	12.16123371349030	9.36768790671386
C	1.94421487644221	11.82397470102489	4.10362641863173
C	2.66928211568032	9.85686823919868	7.83924940705953
C	1.42739721121303	13.60109636010700	6.32667393162120
C	2.16637918296123	8.57165750122644	8.06326453566873
C	0.63463115382219	14.73891928049271	6.45558166014877
C	2.81813504276593	7.45835236463598	7.56735166240719
C	1.22855439673108	15.98378050382276	6.59805477455757
C	3.97424517301471	7.60240359529402	6.81063852003875
C	2.61052553773630	16.10587751157388	6.65097992689448
C	4.46145338562978	8.87535822519236	6.55175056207699
C	3.40217548595697	14.97022974194009	6.55007893194431
C	3.82090734653773	9.99448713029836	7.06119457060571
C	2.81644652650955	13.72864204211624	6.38034290806193
C	0.77847932460624	14.68236066289985	11.22032007340337
C	-0.34619756603386	8.10097329280662	4.10320855311499
C	-0.14677812614101	13.76734296824837	12.03106577335284
C	-1.80594752801350	8.57438411203368	4.11748984501467
C	-0.03168024428203	15.47110416459567	10.18999222859024
C	-0.12065465228663	7.13104516386815	5.26621851110887
C	1.39832190157133	15.67711109723394	12.19366536534621
C	-0.13681581236880	7.33955643443144	2.79655422453692
C	5.63974661986954	13.59833658121310	10.45925394218598
C	3.10689811740056	11.13225396880979	1.96507653632150
C	5.85011186452520	15.06107143459350	10.04621018911114
C	4.13036936856952	9.99163300067933	1.94159064305383
C	5.90811918094037	13.47376083206894	11.96175140130895
C	2.34990912838856	11.15530705240253	0.63429848330201
C	6.63556565401786	12.73134100975075	9.69904942441031
C	3.85117130679729	12.45517159693975	2.10311135328033
H	3.48526175472693	14.78232738985121	11.44349482274631
H	1.50351850377424	9.01300697231998	2.32960668567523
H	4.58798355090431	11.50563720193306	8.94988147736522
H	2.41151875488357	12.79511897195723	4.07089158471157
H	1.24651030518536	8.46642031653434	8.62175017188020

H	-0.44304726841502	14.63162898465951	6.43835263655406
H	2.41030566359949	6.47415169156687	7.76538381256005
H	0.60396633183709	16.86508491775033	6.67640362379606
H	4.48222027558688	6.73240237680584	6.41348975630359
H	3.06890945337901	17.07906931563205	6.77850148021540
H	5.35053653991493	9.00221512916223	5.94594774196581
H	4.48195687008139	15.04838338441277	6.60592811503419
H	4.20519593916669	10.97746088390534	6.83092881645624
H	3.43180662431855	12.84390873419168	6.30443758939714
H	-0.65648805792682	13.05239314230942	11.38779965979241
H	-0.90003057192016	14.36933704119769	12.54546635362460
H	0.42898003446981	13.22223733690179	12.78374872096625
H	-2.46902031360012	7.71621490273634	3.98007903268201
H	-2.05679995512140	9.06987174032502	5.05538569793114
H	-1.98987232275870	9.27563785226768	3.30081360008771
H	-0.77391422688469	16.08804387458403	10.70292701562989
H	0.62738575986182	16.12715421891226	9.61583868230751
H	-0.54643966819918	14.80988422984611	9.49562413568437
H	-0.77818477242624	6.26636067146934	5.14314450708027
H	0.91177960117054	6.77575986892228	5.29375855680085
H	-0.34061695112795	7.60559207396864	6.22022070085671
H	2.05408693411723	16.38845352396387	11.68442958060978
H	0.60084898420409	16.24489232064700	12.67798990973726
H	1.97308067655903	15.17268481651829	12.97513377322268
H	0.88374050035267	6.96415046872635	2.68915490679154
H	-0.37243012246978	7.96134229952199	1.92916612672846
H	-0.80795027690590	6.47925223004050	2.77801877185918
H	5.18994154042129	15.73155715324833	10.59612709112560
H	6.87946192526318	15.36242092438139	10.25800323596811
H	5.65256406046468	15.20036633320610	8.98082966354468
H	4.86461745078609	10.16593629331780	1.15134152663716
H	4.66329613364829	9.92589606418752	2.89274256599937
H	3.65759004218445	9.02673709523290	1.75007752778474
H	6.93192567594533	13.78493303709542	12.18738689391711
H	5.76960893984552	12.44439878090101	12.29944806874298
H	5.23787464032076	14.11045195794272	12.54032782328498
H	1.59490829780347	11.94397293203881	0.63257753830694
H	3.05070097414493	11.35224940373662	-0.18161510843132
H	1.85094849600563	10.20538186476733	0.43256586828595
H	7.65214344870341	13.05965470528980	9.92535411356629
H	6.55668495401385	11.67854367697456	9.98050563894524
H	6.49189137399578	12.81397116400587	8.61881154362191
H	4.40160368877151	12.52039965238941	3.04456041343242
H	3.16623242367721	13.30415831634800	2.04259879422961
H	4.57141740698186	12.55205014549439	1.28843985413147
O	-0.71327325167713	9.93699524385195	9.30716775987926

C	-0.18251593271908	9.93798301690230	10.64384422941446
C	-0.59529488900958	8.59686474325852	11.19855869407271
C	-1.99077397858727	8.45241945347443	10.60390990318300
C	-1.79566482334138	8.98925562337586	9.19581771796718
H	-1.48315001791974	8.21158759437096	8.49566651815496
H	-2.65886148541251	9.51254542417272	8.78681584596699
H	0.89312898901991	10.08859080282897	10.57769657249912
H	-0.62715987502089	10.76576663663827	11.20058391775353
H	0.06688841139081	7.81351312456439	10.82088754605415
H	-0.57479453355073	8.57030466967044	12.28775313727228
H	-2.69481618050862	9.08217064788498	11.15158427830064
H	-2.36504721325358	7.42935199389184	10.60973782830837

List S7 *Trans*-[(^{Ph}isq)(^{Ph}ibq)V(O)Cl] (**IIa**), S = 0

V	5.239142	5.727748	7.734210
Cl	6.175924	7.283979	9.224771
O	3.905676	5.060586	6.489698
O	3.907100	7.571475	7.514077
O	6.492872	4.766755	7.639893
N	5.932805	6.931036	6.032503
N	4.070457	4.833978	9.019752
C	7.040684	6.461458	5.265021
C	5.330303	8.045111	5.723464
C	6.972016	5.165396	4.755307
H	6.088967	4.569505	4.939134
C	4.186181	8.383902	6.639005
C	1.434126	5.143192	4.912010
C	9.175533	5.444406	3.821460
H	10.014525	5.039772	3.268571
C	3.853267	10.389675	5.356148
H	3.348054	11.327688	5.172107
C	8.172657	7.254720	5.077959
H	8.225477	8.237123	5.526987
C	4.433825	4.005122	10.094847
C	5.617370	8.884722	4.580566
H	6.381881	8.570153	3.889051
C	2.399904	10.075866	7.431476
C	2.777022	4.880454	8.525834
C	5.142847	10.948506	3.215674
C	9.246350	6.739438	4.340317
H	10.139650	7.334883	4.200166
C	4.895887	10.016494	4.394557
C	3.477464	9.655910	6.429310
C	2.735400	5.012081	7.106959
C	3.651343	2.914141	10.518410
H	2.708846	2.707978	10.029919
C	5.693232	4.214304	10.696045
H	6.299486	5.045679	10.366472
C	0.356489	4.965958	7.230695

H	-0.605529	4.988513	6.737840
C	8.035531	4.668804	4.013546
H	7.985330	3.665423	3.610818
C	1.493268	5.036612	6.433143
C	4.115545	2.059306	11.513050
H	3.510991	1.213365	11.816359
C	3.058361	10.243816	8.807747
H	3.489651	9.310955	9.167569
H	3.850208	10.995180	8.775347
H	2.306282	10.579883	9.525750
C	6.135063	3.360502	11.699558
H	7.100810	3.535464	12.156532
C	0.362747	4.878974	8.644666
C	1.594157	4.825326	9.287696
H	1.672713	4.782592	10.364068
C	5.347932	2.289237	12.110587
H	5.707391	1.622253	12.885057
C	2.100578	6.440818	4.458034
H	1.650301	7.300062	4.958501
H	3.165733	6.432811	4.682878
H	1.970203	6.570404	3.379940
C	1.290848	9.020711	7.524240
H	0.778135	8.901898	6.566563
H	1.674783	8.050363	7.829801
H	0.552010	9.340954	8.261545
C	5.611311	12.297842	3.757811
H	4.878425	12.748635	4.429126
H	6.550418	12.199156	4.305473
H	5.769055	12.990638	2.927563
C	1.760485	11.420476	7.035557
H	2.498272	12.226766	7.044595
H	1.286991	11.379039	6.051978
H	0.990651	11.669528	7.765556
C	-0.964790	4.870501	9.407039
C	6.213293	10.411194	2.255949
H	7.181711	10.316135	2.751705
H	5.937675	9.439527	1.842523
H	6.332871	11.107597	1.425453
C	3.823662	11.126621	2.417931
H	3.476524	10.167090	2.030972
H	3.031100	11.559528	3.028796
H	3.999055	11.795229	1.573146
C	2.157049	3.935856	4.279021
H	3.207258	3.907054	4.563342
H	1.688908	3.001771	4.595942
H	2.083124	3.993085	3.189306
C	-0.009815	5.131369	4.397275
H	-0.517196	4.196052	4.642219
H	-0.590142	5.959450	4.809481
H	-0.008342	5.242777	3.310505

C	-0.764033	4.531253	10.894390
H	-0.301738	3.550907	11.025281
H	-0.147547	5.274386	11.402337
H	-1.735891	4.515544	11.390669
C	-1.899621	3.810699	8.844567
H	-2.134879	3.975305	7.792037
H	-1.457522	2.816014	8.937074
H	-2.842817	3.811284	9.395401
C	-1.629075	6.229919	9.305346
H	-0.977003	7.012483	9.698591
H	-1.863599	6.483364	8.268694
H	-2.561951	6.243728	9.874066

List S8 *Trans*-[(^{Ph}isq)(^{Ph}ibq)V(O)Cl] (**IIa**), S = 1

V	5.239135	5.727847	7.734466
Cl	6.175863	7.284189	9.224946
O	3.905697	5.060578	6.489982
O	3.907046	7.571527	7.514216
O	6.492891	4.766882	7.640215
N	5.932781	6.931055	6.032696
N	4.070463	4.834120	9.020050
C	7.040679	6.461463	5.265250
C	5.330252	8.045096	5.723587
C	6.972049	5.165369	4.755610
H	6.090046	4.569775	4.943504
C	4.186114	8.383910	6.639099
C	1.434159	5.143028	4.912268
C	9.175567	5.444383	3.821766
H	10.014576	5.039906	3.268617
C	3.853158	10.389601	5.356124
H	3.346925	11.326816	5.170351
C	8.172633	7.254744	5.078152
H	8.224893	8.237011	5.527512
C	4.433844	4.005336	10.095195
C	5.617307	8.884650	4.580643
H	6.381283	8.569185	3.888780
C	2.399786	10.075873	7.431457
C	2.777031	4.880534	8.526118
C	5.142741	10.948342	3.215628
C	9.246346	6.739448	4.340550
H	10.139887	7.334649	4.200106
C	4.895796	10.016391	4.394563
C	3.477365	9.655887	6.429325
C	2.735418	5.012078	7.107235
C	3.651386	2.914359	10.518814
H	2.713774	2.702284	10.023050
C	5.693239	4.214585	10.696393
H	6.297369	5.048100	10.365109

C	0.356507	4.965900	7.230954
H	-0.605741	4.997702	6.738703
C	8.035584	4.668762	4.013887
H	7.986393	3.664880	3.612005
C	1.493290	5.036538	6.433407
C	4.115603	2.059593	11.513508
H	3.513701	1.210272	11.811969
C	3.058226	10.243920	8.807725
H	3.495002	9.312314	9.164362
H	3.847397	10.997949	8.774736
H	2.306034	10.576602	9.527465
C	6.135084	3.360853	11.699958
H	7.100697	3.537005	12.156223
C	0.362755	4.878997	8.644929
C	1.594160	4.825419	9.287973
H	1.671488	4.782905	10.364318
C	5.347979	2.289591	12.111042
H	5.707941	1.620994	12.883773
C	2.100581	6.440646	4.458223
H	1.648173	7.301055	4.954688
H	3.164544	6.434301	4.686590
H	1.971915	6.566102	3.379656
C	1.290756	9.020694	7.524273
H	0.777478	8.902826	6.566660
H	1.678772	8.051252	7.827637
H	0.552324	9.338942	8.263114
C	5.611165	12.297721	3.757692
H	4.877903	12.747744	4.429121
H	6.549661	12.198114	4.306259
H	5.769934	12.991434	2.928249
C	1.760334	11.420443	7.035455
H	2.498856	12.225907	7.043970
H	1.287299	11.379088	6.051655
H	0.990570	11.670668	7.765375
C	-0.964788	4.870533	9.407292
C	6.213209	10.411003	2.255943
H	7.181268	10.315283	2.752208
H	5.937556	9.439189	1.842891
H	6.333307	11.107363	1.425356
C	3.823559	11.126376	2.417864
H	3.476736	10.166378	2.031607
H	3.031225	11.558735	3.029412
H	3.997713	11.794793	1.572521
C	2.157119	3.935675	4.279354
H	3.207574	3.908925	4.562414
H	1.689899	3.000527	4.595166
H	2.081741	3.994110	3.190001
C	-0.009778	5.131138	4.397520
H	-0.518278	4.195359	4.639641
H	-0.590771	5.959633	4.808552

H	-0.006816	5.243984	3.311162
C	-0.764036	4.531376	10.894664
H	-0.303256	3.550369	11.026096
H	-0.147655	5.274386	11.402863
H	-1.736023	4.516899	11.390300
C	-1.899587	3.810674	8.844873
H	-2.135913	3.973903	7.792219
H	-1.459600	2.815200	8.939126
H	-2.842410	3.814061	9.395741
C	-1.629109	6.229928	9.305515
H	-0.978759	7.012767	9.700685
H	-1.863683	6.485199	8.269248
H	-2.562629	6.241319	9.872762

List S9 *Trans*-[(^{Ph}isq)(^{Ph}ibq)V(O)Cl] (**IIa**), CPCM (benzene), S = 0

V	5.80454287177348	5.25395279320373	7.44822259022712
Cl	7.37728009777445	6.01300930612050	8.99637020343480
O	4.10282564167576	5.20138610850803	6.39779493398954
O	4.95649211643513	7.32658707700439	7.90329144189354
O	6.53928540406342	3.94059892294634	6.99564944803560
N	6.37089597316640	6.58541443460724	5.87055087734472
N	4.43692194244470	4.71077330581385	8.87263024702722
C	7.15611672443919	6.10818752235070	4.79584284030838
C	5.81483324205051	7.75631517096340	5.78200787827002
C	6.75172914295151	4.95696420286091	4.12740840248737
H	5.85249703358302	4.44790329896281	4.44674944625207
C	5.01358883412318	8.13973807259024	6.98901708394438
C	1.48703246655284	5.71679413842684	5.08935383951420
C	8.65664738866058	5.15240617346957	2.67129031176569
H	9.24360910208132	4.77508417817949	1.84320485125928
C	4.37284486257068	10.13115622982974	5.85044264702603
H	3.83408128052756	11.06485890966038	5.80618504867600
C	8.32022469642292	6.77115752852677	4.42049641287561
H	8.63768238561640	7.64689725279660	4.97290213398966
C	4.71305001806959	4.37615234152595	10.21519055396474
C	5.80566032596569	8.60594944770667	4.62848390687125
H	6.32739301837277	8.27660301945724	3.74422023008669
C	3.52983582855466	9.86258078415960	8.21663493753691
C	3.16644648722465	4.81368645470103	8.48166031119189
C	4.84095471670095	10.59473611317115	3.40435311800170
C	9.06718092235557	6.28690964395724	3.35730011496911
H	9.97782410805705	6.79622327195266	3.06711868088836
C	5.06749813647436	9.73904509247158	4.63731662867152
C	4.32200392026026	9.42109130811831	6.99784606075225
C	3.00960235240017	5.12797292036197	7.08137198305604
C	5.05196778661930	3.07081616591174	10.54079519368283
H	5.11314353752605	2.33176661514127	9.75148350651343

C	4.65157106365469	5.35126385409443	11.20399951875122
H	4.40534876864849	6.36831295239886	10.92648057506361
C	0.65448994155565	5.14398925773108	7.42101480759145
H	-0.34843218701707	5.28373773521785	7.04265828513641
C	7.49493108901853	4.49481374520036	3.05546973800396
H	7.16802530985229	3.60835154686914	2.52651746200902
C	1.70712316282182	5.33066184238181	6.55084649631463
C	5.30673436985625	2.73427128640526	11.86190649651515
H	5.56835044979039	1.71391178269995	12.11416401754783
C	4.43346173396318	9.91618587480020	9.45369761685043
H	4.85627818902986	8.94282860764388	9.69592001291443
H	5.25381635054833	10.62033217731515	9.29844411144530
H	3.84689783086606	10.26670718317640	10.30626132393994
C	4.91870044491278	5.01061684549819	12.51938718962826
H	4.87940342336717	5.77352759191892	13.28729263765122
C	0.78378926421602	4.76973883638883	8.78807234674369
C	2.03772208276687	4.62071563762955	9.30845348503403
H	2.19767855875572	4.34704249535488	10.34062742368507
C	5.24075089524448	3.70227116588742	12.85432705991784
H	5.45062766374815	3.44049162538049	13.88406831884017
C	2.38227030302849	6.89512012103296	4.69016347021438
H	2.21220990897158	7.75881994833772	5.33560528535126
H	3.43331405181880	6.62412187474974	4.74499565904059
H	2.15879334405897	7.19337252174459	3.66305369729679
C	2.37542031808391	8.88036702585736	8.45552588948798
H	1.73169132565656	8.81410641209899	7.57604530678742
H	2.73843399092434	7.88184806967632	8.69117409780254
H	1.76734104668861	9.23024017761718	9.29264836218021
C	4.98348810502833	12.08586492776831	3.73324023026738
H	4.23440346170745	12.43342280503881	4.44507124203925
H	5.97220891565565	12.31335562851906	4.13750463441135
H	4.84213990552090	12.66361480730323	2.81775977880170
C	2.95177293233074	11.25989820964775	8.01757210228305
H	3.74238537508639	12.00248217552064	7.88749426793041
H	2.27266225061214	11.31527564193905	7.16353402245550
H	2.38282916028631	11.53725779459138	8.90571419786177
C	-0.47521040185227	4.52474279249278	9.60486721707116
C	5.81594761487726	10.24862877127285	2.28619782123468
H	6.85148683214463	10.37447483543988	2.61062036818540
H	5.68293788336754	9.22383846435688	1.93302391338642
H	5.64586024383151	10.91144601330337	1.43608419993292
C	3.40519376632971	10.33585263678600	2.91858775960457
H	3.26715702269648	9.29176323332420	2.63108213106583
H	2.67151764885484	10.57584507977944	3.68995375574651
H	3.19597874800855	10.96399002456305	2.04993312590118
C	1.81371137726084	4.52322398909871	4.18603969284471

H	2.85293382903174	4.21199827703623	4.30170212339046
H	1.17040987149050	3.67188220554662	4.41842041900659
H	1.64795813637899	4.79746899371110	3.14054377561492
C	0.03518621926148	6.11918342208078	4.83430724451971
H	-0.64623648480525	5.27461866792104	4.95452693780569
H	-0.28580990718489	6.92055800763274	5.50394872524199
H	-0.06709229549380	6.47942077320555	3.80825450557978
C	-0.16944893769710	4.20270479803042	11.06346429999692
H	0.46686854743703	3.32140874566089	11.16168719879569
H	0.31856139827099	5.03880071601097	11.56816203427018
H	-1.10448352145311	3.99028246031015	11.58632697756533
C	-1.22173838388743	3.32362197500479	9.01166961251266
H	-1.47272018130655	3.47350819208797	7.96016281686532
H	-0.61002792929261	2.42271688361262	9.09335090844445
H	-2.15225059921895	3.15388426617250	9.55874005172986
C	-1.36528190232032	5.77091361106275	9.56419862872694
H	-0.83361061782541	6.63661459072113	9.96502889014499
H	-1.68980521545867	6.01438601815419	8.55142029397908
H	-2.25931792799939	5.60729356472529	10.17020753853150

List S10 *Trans*-[(^{Ph}isq)(^{Ph}ibq)V(O)Cl] (**IIa**), CPCM (benzene), S = 1

V	5.72730245762326	5.43451795799524	7.52650344058252
Cl	7.29699165584188	6.16090408217386	9.07592198688985
O	3.99805960157690	5.46117974358384	6.50099587147780
O	4.95102419666763	7.53414434075536	7.90694377543743
O	6.40618411787634	4.09593776768399	7.06712452295364
N	6.34546449948894	6.66461321286956	5.90482704014788
N	4.36236528484471	4.83728221990180	8.95507993948202
C	7.10650561912159	6.12172785723965	4.84231502985701
C	5.81357614916907	7.84204316854658	5.76395554612252
C	6.65163158310385	4.97569493913987	4.19905395581543
H	5.73058683314925	4.51518501092919	4.52761651378616
C	5.05270869820912	8.31250155218615	6.96606175315251
C	1.39702794123467	5.71067208606478	5.16982763447547
C	8.56816392099732	5.04621296156653	2.74755412891224
H	9.14001455317790	4.62263275976652	1.93138344627001
C	4.48810414565893	10.28425015851902	5.75228118143512
H	3.99150473858930	11.23999017900580	5.67969639960466
C	8.30071029446447	6.72098311747815	4.45593065565230
H	8.65626342476618	7.59410798489236	4.98886667020068
C	4.64920388854624	4.42347679212824	10.27414199790877
C	5.80780636862445	8.63193533518802	4.56923900333779
H	6.29590362353284	8.23652346368585	3.69241145840381
C	3.72849688387945	10.17845336282166	8.15977754683871
C	3.09561812982063	4.82345057288241	8.54520092086293
C	4.91719026529777	10.61078285497746	3.27403649352151

C	9.02892323063604	6.17593191484558	3.40897217768637
H	9.96247930597826	6.63682234509719	3.11082895176889
C	5.12382340277854	9.79841749287421	4.54013232224598
C	4.44358867458803	9.63455429349948	6.93531666328997
C	2.92148241332644	5.20252715770058	7.16226915087767
C	4.97178336509860	3.09341024388481	10.50943907611179
H	5.02893180458645	2.41038719886231	9.67080598506816
C	4.59617151561889	5.32624055822696	11.32818923221216
H	4.36154481934975	6.36339202523451	11.12426855352099
C	0.57159186265482	4.94862606714693	7.45407307320374
H	-0.43461259057764	5.00056306975097	7.06127896840274
C	7.37749047351330	4.45136101390642	3.14377539129968
H	7.01202091200382	3.56785460182319	2.63545170262320
C	1.61239275150134	5.27778057567094	6.61528546457042
C	5.21175176366694	2.66107288556563	11.80495010347400
H	5.45874194501491	1.62168592788364	11.98470988077971
C	4.67155452030013	10.18157543418308	9.36877696257296
H	5.03931650550066	9.18576266714944	9.60765001275121
H	5.52889949123616	10.83247695431320	9.18310388626635
H	4.13527071592376	10.57229701803044	10.23651079052527
C	4.84484413111171	4.88845410804737	12.62017270148210
H	4.80293628109039	5.59414454745366	13.44074589960368
C	0.72111418905181	4.54021381355597	8.81110474951645
C	1.97737403664459	4.48154584611938	9.34185978183691
H	2.14964401112292	4.17770570998792	10.36385124762816
C	5.14665288941624	3.55599032439979	12.86383894406017
H	5.33904773362103	3.21657044850776	13.87415022642558
C	1.93009433699779	7.13591291704555	4.97265070469583
H	1.40276190558907	7.83750218680185	5.62195970368654
H	2.99474072371298	7.19793049268464	5.19272612708462
H	1.77051369262613	7.44938894991409	3.93739287307586
C	2.49992058584347	9.30930674480823	8.45672623481209
H	1.82144424057864	9.29544503193394	7.60132001755129
H	2.78102518121157	8.28458531192804	8.69460097877349
H	1.95939639388343	9.72730839492652	9.30876805670662
C	5.15675075841636	12.10285654536299	3.53484079937981
H	4.43613425952798	12.52695254651934	4.23460789952395
H	6.16091474489607	12.28651078310763	3.92315406576214
H	5.04571939075437	12.64665181504163	2.59503850835108
C	3.26824754157377	11.61598426254989	7.93783098850287
H	4.11517866572313	12.28003242702960	7.75176826626305
H	2.56275794106647	11.70837456720348	7.10901270971378
H	2.76157546418914	11.96796547667089	8.83763297029993
C	-0.52504375227176	4.19729427170716	9.61211899988557
C	5.83973992402820	10.15890618911644	2.14885582856948
H	6.88997554204753	10.24612719582650	2.43670773232951

H	5.64489976891753	9.12663053396969	1.85106207104347
H	5.67710615347377	10.78760789830124	1.27184535283989
C	3.45877484775896	10.41901884225937	2.82836901106440
H	3.26157586983867	9.37727882049186	2.56789334664554
H	2.75579616111764	10.71462395384209	3.60897878845670
H	3.26283546340931	11.03751839524077	1.94966461651576
C	2.11959090191612	4.74348557794356	4.22426447571523
H	3.19123126961511	4.72858758847814	4.41631173327963
H	1.73393446749928	3.72864957590777	4.34543971985067
H	1.95275676540588	5.04580442134089	3.18702469625858
C	-0.08209247221254	5.71959665402814	4.79356739904716
H	-0.52221814874403	4.72096661097888	4.84297616316459
H	-0.65892937838006	6.38438071980961	5.43877494838142
H	-0.19190996570302	6.08274941945012	3.76878840655588
C	-0.20394538511516	3.79515248666679	11.04833170773455
H	0.45889660384620	2.92879026120088	11.09186208225077
H	0.26183303433445	4.61221293200149	11.60227417654190
H	-1.13110268365869	3.52522044076599	11.55865407356802
C	-1.25160980214128	3.02062612263792	8.95077930486667
H	-1.53307265800378	3.23679149329947	7.91880576652412
H	-0.61693564793039	2.13228202401673	8.95450591087797
H	-2.16522515325182	2.78903631877059	9.50372684578973
C	-1.44306788445423	5.42449341352011	9.65026180208085
H	-0.93336812446496	6.27058846798580	10.11575410990163
H	-1.76225960708256	5.73095959485256	8.65266695030443
H	-2.33951096640618	5.20207359635624	10.23385426283814

List S11 *Cis*-[(^{Ph}isq)(^{Ph}ibq)V(O)Cl] (**IIb**), S = 0

V	-6.916217	9.504892	16.088748
Cl	-7.252268	9.234850	13.792677
O	-8.412721	9.281631	16.610378
O	-4.994259	10.401199	15.474999
O	-5.945611	7.838312	16.129670
N	-5.970959	9.650727	17.862855
N	-7.172135	11.629454	16.033426
C	-2.976446	7.808716	19.000430
C	-4.914936	8.802945	17.971258
C	-6.155073	12.388045	15.706723
C	-4.897855	11.636776	15.432536
C	-4.934075	7.800901	16.973813
C	-3.669671	12.361070	15.207001
C	-2.983847	6.837822	17.958526
H	-2.207133	6.087585	17.966964
C	-3.944926	8.792063	19.005562
H	-3.988009	9.546863	19.774189
C	-3.938375	6.787947	16.956336
C	-5.333750	11.688880	19.082138

H	-4.389617	11.707157	18.552542
C	-6.241518	10.645025	18.839418
C	-1.896698	7.746393	20.079142
C	-6.121291	13.833981	15.595348
H	-7.059869	14.360833	15.686997
C	-3.949545	5.692395	15.862604
C	-5.654354	12.675385	19.995751
H	-4.953789	13.476904	20.195908
C	-0.538211	7.998483	19.419289
H	-0.350706	7.291072	18.610375
H	0.261897	7.877674	20.155584
H	-0.483517	9.006950	19.005432
C	-8.394633	12.243631	16.448374
C	-4.989946	14.471047	15.409624
C	-7.465274	10.626641	19.483133
H	-8.166617	9.838622	19.241647
C	-7.785457	11.624055	20.394273
H	-8.747251	11.608081	20.891314
C	-2.104228	8.795239	21.165563
H	-2.032360	9.806698	20.758001
H	-1.327850	8.690339	21.926256
H	-3.071130	8.684742	21.660530
C	-5.276542	4.888629	15.979289
H	-5.257030	4.060178	15.267102
H	-6.135701	5.517644	15.753568
H	-5.391988	4.475508	16.983009
C	-8.403177	13.163108	17.497693
H	-7.473513	13.412443	17.991901
C	-2.800983	4.677895	16.067301
H	-2.856860	4.195744	17.044767
H	-1.823920	5.157822	15.960129
H	-2.878881	3.902360	15.303350
C	-2.053462	10.704672	16.172193
H	-1.939328	11.318661	17.069353
H	-1.117472	10.163042	16.013974
H	-2.837203	9.971538	16.351365
C	-1.897959	6.358204	20.738696
H	-2.877297	6.126667	21.161871
H	-1.161930	6.325828	21.545960
H	-1.638433	5.574402	20.025825
C	-3.846404	6.329421	14.504309
H	-3.908647	5.562124	13.727245
H	-2.894625	6.853818	14.385765
H	-4.653530	7.040688	14.331608
C	-4.896683	15.993331	15.404393
C	-2.360890	11.591096	14.949837
C	-4.100122	16.412554	16.658459
H	-4.631923	16.125164	17.568238
H	-3.958920	17.495718	16.671596
H	-3.115716	15.944016	16.680461

C	-1.189276	12.542906	14.761575
H	-1.333143	13.214339	13.912489
H	-0.287943	11.958199	14.566329
H	-1.006221	13.144354	15.655425
C	-9.570551	11.835484	15.873516
H	-9.551423	11.079248	15.100242
C	-4.134157	16.518089	14.159464
H	-3.111515	16.143972	14.102212
H	-4.085906	17.607480	14.204379
H	-4.651037	16.235610	13.240648
C	-10.760554	13.367562	17.281228
H	-11.696933	13.813488	17.600093
C	-6.252317	16.668214	15.370200
H	-6.838735	16.326101	14.515119
H	-6.129107	17.747809	15.271928
H	-6.835285	16.481039	16.275744
C	-10.774244	12.418498	16.288712
H	-11.702239	12.107321	15.827305
C	-6.886288	12.641414	20.650581
H	-7.141262	13.419279	21.360395
C	-3.741097	13.721348	15.246309
H	-2.839058	14.294741	15.091896
C	-2.542904	10.754192	13.678325
H	-3.360583	10.043425	13.776958
H	-1.627897	10.194504	13.467512
H	-2.748432	11.404756	12.825621
C	-9.603088	13.747668	17.890667
H	-9.606866	14.466529	18.700091

List S12 *Cis*-[(^{Ph}isq)(^{Ph}ibq)V(O)Cl] (**IIb**), S = 1

V	-6.916120	9.504952	16.088779
Cl	-7.252299	9.234933	13.792724
O	-8.412588	9.281652	16.610493
O	-4.994216	10.401308	15.474928
O	-5.945475	7.838392	16.129622
N	-5.970761	9.650784	17.862832
N	-7.172086	11.629509	16.033499
C	-2.976142	7.808821	19.000207
C	-4.914713	8.803023	17.971162
C	-6.155059	12.388126	15.706747
C	-4.897842	11.636888	15.432476
C	-4.933890	7.800991	16.973705
C	-3.669687	12.361211	15.206879
C	-2.983583	6.837941	17.958290
H	-2.210303	6.084092	17.969837
C	-3.944642	8.792148	19.005409
H	-3.995871	9.539438	19.780820
C	-3.938169	6.788059	16.956156
C	-5.333524	11.688934	19.082104

H	-4.392348	11.709382	18.546842
C	-6.241284	10.645063	18.839424
C	-1.896330	7.746507	20.078855
C	-6.121315	13.834064	15.595389
H	-7.059921	14.360605	15.687485
C	-3.949380	5.692521	15.862410
C	-5.654096	12.675420	19.995749
H	-4.955828	13.479799	20.193130
C	-0.537886	7.998635	19.418925
H	-0.349006	7.291694	18.609818
H	0.260721	7.876557	20.156295
H	-0.482126	9.007542	19.006472
C	-8.394573	12.243654	16.448527
C	-4.989995	14.471157	15.409606
C	-7.465002	10.626644	19.483210
H	-8.167440	9.841056	19.236501
C	-7.785153	11.624040	20.394382
H	-8.748535	11.610394	20.888392
C	-2.103818	8.795334	21.165302
H	-2.033544	9.806716	20.757573
H	-1.326166	8.690958	21.924611
H	-3.069718	8.683809	21.661806
C	-5.276352	4.888725	15.979162
H	-5.254033	4.058188	15.269618
H	-6.134321	5.517603	15.749023
H	-5.394488	4.477974	16.983684
C	-8.403075	13.163118	17.497858
H	-7.473648	13.411279	17.993262
C	-2.800784	4.678042	16.067026
H	-2.857239	4.192956	17.043202
H	-1.823469	5.157974	15.961114
H	-2.878060	3.904200	15.301459
C	-2.053386	10.704834	16.171954
H	-1.934119	11.319006	17.068324
H	-1.120189	10.159304	16.010838
H	-2.839511	9.974842	16.353047
C	-1.897522	6.358309	20.738391
H	-2.876135	6.126185	21.162852
H	-1.161217	6.328921	21.545169
H	-1.636561	5.573476	20.027164
C	-3.846331	6.329567	14.504118
H	-3.916732	5.562272	13.727944
H	-2.890952	6.846866	14.382431
H	-4.650214	7.045640	14.335483
C	-4.896764	15.993443	15.404391
C	-2.360904	11.591268	14.949628
C	-4.100139	16.412666	16.658416
H	-4.632189	16.126245	17.568326
H	-3.958859	17.495741	16.670430
H	-3.115480	15.944542	16.681233

C	-1.189321	12.543105	14.761310
H	-1.333052	13.214572	13.912255
H	-0.288207	11.958270	14.565644
H	-1.005477	13.144267	15.655239
C	-9.570516	11.835490	15.873732
H	-9.550288	11.074710	15.104760
C	-4.134323	16.518234	14.159424
H	-3.112087	16.142932	14.100717
H	-4.084344	17.607404	14.205728
H	-4.652384	16.237799	13.240663
C	-10.760470	13.367524	17.281534
H	-11.696862	13.812842	17.601325
C	-6.252414	16.668298	15.370286
H	-6.838868	16.326296	14.515218
H	-6.128936	17.747825	15.272021
H	-6.835381	16.481174	16.275795
C	-10.774197	12.418472	16.289007
H	-11.702103	12.106300	15.828133
C	-6.885991	12.641414	20.650651
H	-7.142086	13.420902	21.358311
C	-3.741139	13.721487	15.246209
H	-2.837514	14.294566	15.099464
C	-2.542975	10.754377	13.678115
H	-3.363723	10.046806	13.774368
H	-1.629101	10.191745	13.470560
H	-2.743441	11.405295	12.824523
C	-9.602976	13.747647	17.890911
H	-9.606871	14.466177	18.700731

List S13 *Cis*-[(^{Ph}isq)$_2$V(O)Cl] (**IIb'**), S = 0

V	-1.062495	13.552824	19.671892
Cl	-0.514180	15.038343	17.940163
O	0.114123	12.259614	18.865139
O	0.849321	14.258451	20.660137
O	-2.578700	13.301701	19.262998
N	-0.834643	12.257032	21.195069
N	-1.565639	15.138359	21.005453
C	0.839788	11.593998	19.751764
C	-1.617335	12.269899	22.394812
C	0.282435	11.511726	21.067115
C	2.064568	10.970166	19.447011
C	1.744417	15.543577	22.492227
C	-2.965229	11.932402	22.325486
H	-3.387400	11.642459	21.372202
C	-1.070536	12.702868	23.599916
H	-0.029935	12.998372	23.634985
C	1.441005	16.581470	23.340850
H	2.198428	16.943964	24.020049
C	2.617688	10.175304	20.439806

H	3.552355	9.676375	20.230151
C	-3.208212	12.426539	24.673780
H	-3.824586	12.485230	25.562009
C	-3.534872	15.234358	22.468487
H	-2.944043	14.773089	23.248725
C	-4.864270	15.552845	22.697927
H	-5.304863	15.325380	23.660712
C	-1.871607	12.803000	24.723353
H	-1.442829	13.163776	25.650703
C	-3.751249	11.998248	23.471147
H	-4.796570	11.722555	23.413633
C	-0.845355	16.861394	22.584879
H	-1.813107	17.337355	22.567875
C	3.821785	15.128113	21.155892
H	3.972907	16.196161	20.984822
H	3.221931	14.731597	20.337383
H	4.798156	14.638636	21.137900
C	0.079549	18.525139	24.241879
C	2.730545	9.106467	22.761046
C	2.479632	7.646586	22.398078
H	2.799354	7.436898	21.375388
H	1.420934	7.393245	22.488485
H	3.041284	6.990737	23.067395
C	1.116844	19.542109	23.837021
H	0.971900	19.859246	22.802095
H	2.134510	19.156602	23.930303
H	1.044851	20.427921	24.472936
C	4.243936	9.342604	22.743981
H	4.712242	8.726701	23.514735
H	4.486950	10.386395	22.954257
H	4.699733	9.067168	21.793447
C	-1.292982	19.184852	24.123128
H	-1.331477	20.068806	24.761978
H	-2.091216	18.511066	24.444311
H	-1.500022	19.504890	23.100435
C	0.296443	18.038949	25.684031
H	1.296839	17.631413	25.837373
H	-0.428947	17.264596	25.937263
H	0.158813	18.870424	26.380217
C	2.042603	10.016689	21.736137
C	0.170213	17.298801	23.338162
C	0.682305	15.221498	21.641143
C	0.901836	10.690343	22.039660
H	0.452744	10.629096	23.019338
C	2.721857	11.181720	18.086490
C	3.141459	14.869828	22.491722
C	-0.620827	15.725377	21.728099
C	-2.934657	15.505154	21.246235
C	4.026892	15.416370	23.615381
H	3.579827	15.256377	24.599113

H	4.239834	16.480477	23.491552
H	4.980174	14.886762	23.592706
C	2.975111	13.366303	22.701648
H	3.956013	12.885482	22.708521
H	2.386424	12.924874	21.902017
H	2.490519	13.149317	23.656438
C	-3.673872	16.057030	20.213923
H	-3.223513	16.230911	19.246166
C	-5.612287	16.131216	21.692773
H	-6.650407	16.391541	21.859294
C	-5.022139	16.391107	20.480399
H	-5.603790	16.840715	19.684562
C	2.785247	12.665027	17.730849
H	3.305211	13.225975	18.508928
H	1.801489	13.108238	17.594380
H	3.346567	12.791662	16.801745
C	4.188405	10.696470	18.084257
H	4.627025	10.913744	17.109640
H	4.277194	9.619327	18.247199
H	4.775800	11.213001	18.845423
C	1.945161	10.395173	17.049277
H	0.897539	10.697231	17.027150
H	1.995773	9.323105	17.254479
H	2.363334	10.569808	16.054064
C	2.252347	9.416813	24.178372
H	1.193843	9.188842	24.320690
H	2.425064	10.465371	24.428688
H	2.808182	8.803887	24.890086

List S14 *Cis*-[(^{Ph}isq)$_2$V(O)Cl] (**IIb’**), S = 1

V	-1.062292	13.552583	19.671937
Cl	-0.514070	15.038105	17.940180
O	0.114368	12.259413	18.865179
O	0.849510	14.258303	20.660140
O	-2.578493	13.301390	19.263071
N	-0.834359	12.256819	21.195125
N	-1.565481	15.138112	21.005487
C	0.840076	11.593839	19.751799
C	-1.617032	12.269666	22.394881
C	0.282749	11.511559	21.067160
C	2.064877	10.970056	19.447033
C	1.744582	15.543489	22.492200
C	-2.964912	11.932111	22.325582
H	-3.388557	11.643203	21.372616
C	-1.070230	12.702673	23.599970
H	-0.032539	13.009262	23.630741
C	1.441141	16.581379	23.340816
H	2.195367	16.940035	24.025472
C	2.618048	10.175228	20.439827

H	3.550053	9.672185	20.228132
C	-3.207876	12.426265	24.673874
H	-3.825270	12.488337	25.561147
C	-3.534693	15.234044	22.468554
H	-2.942403	14.774655	23.248691
C	-4.864102	15.552477	22.698012
H	-5.304952	15.327056	23.661440
C	-1.871286	12.802784	24.723420
H	-1.443513	13.168100	25.649452
C	-3.750916	11.997936	23.471255
H	-4.797198	11.726101	23.413177
C	-0.845244	16.861196	22.584881
H	-1.814814	17.333418	22.573199
C	3.821946	15.128098	21.155835
H	3.975904	16.196119	20.987456
H	3.217544	14.736569	20.338118
H	4.796877	14.635473	21.134863
C	0.079618	18.525000	24.241845
C	2.730990	9.106423	22.761078
C	2.480133	7.646528	22.398132
H	2.798829	7.434937	21.375401
H	1.422210	7.391414	22.491484
H	3.044762	6.993743	23.067469
C	1.116862	19.542010	23.836958
H	0.971939	19.858418	22.801776
H	2.134130	19.155734	23.930179
H	1.044554	20.428250	24.472394
C	4.244371	9.342625	22.743985
H	4.710900	8.727252	23.515923
H	4.487863	10.386314	22.953816
H	4.701486	9.065529	21.794496
C	-1.292944	19.184653	24.123110
H	-1.331353	20.069367	24.761097
H	-2.090992	18.511023	24.445026
H	-1.500498	19.503089	23.100050
C	0.296557	18.038836	25.683999
H	1.297462	17.632774	25.836942
H	-0.427492	17.262746	25.935878
H	0.156564	18.869414	26.380962
C	2.042992	10.016605	21.736170
C	0.170318	17.298655	23.338141
C	0.682471	15.221354	21.641138
C	0.902201	10.690213	22.039705
H	0.447508	10.622877	23.016269
C	2.722134	11.181622	18.086498
C	3.141653	14.869800	22.491679
C	-0.620682	15.725178	21.728110
C	-2.934510	15.504852	21.246288
C	4.027082	15.416392	23.615317
H	3.579165	15.257109	24.598661

H	4.239349	16.480385	23.491110
H	4.980703	14.886919	23.593728
C	2.975373	13.366269	22.701626
H	3.956022	12.884219	22.704538
H	2.382998	12.929009	21.902658
H	2.493240	13.149523	23.657772
C	-3.673766	16.056684	20.213982
H	-3.223570	16.232793	19.246624
C	-5.612160	16.130805	21.692864
H	-6.650001	16.392244	21.859746
C	-5.022043	16.390706	20.480478
H	-5.604695	16.840492	19.685252
C	2.785455	12.664927	17.730838
H	3.304084	13.226709	18.509043
H	1.801987	13.107994	17.593159
H	3.347652	12.790188	16.802250
C	4.188703	10.696434	18.084246
H	4.626942	10.914281	17.109741
H	4.278362	9.619141	18.246259
H	4.776000	11.213335	18.845224
C	1.945454	10.395029	17.049307
H	0.898042	10.697555	17.025278
H	1.995280	9.322781	17.253960
H	2.364912	10.569442	16.054734
C	2.252803	9.416766	24.178409
H	1.194748	9.187507	24.321519
H	2.424679	10.465401	24.428505
H	2.809820	8.804348	24.889389

References

1. Chun, H.; Verani, C. N.; Chaudhuri, P.; Bothe, E.; Bill, E.; Weyhermüller, T.; Wieghardt, K. Molecular and Electronic Structure of Octahedral o-Aminophenolato and o-Iminobenzosemiquinonato Complexes of V(V), Cr(III), Fe(III), and Co(III). Experimental Determination of Oxidation Levels of Ligands and Metal Ions. *Inorg. Chem.* **2001**, *40*, 4157-4166.
2. *APEX2 suite for crystallographic software*, Bruker axs: Madison, WI, 2014.
3. *SAINT - Software for the Integration of CCD Detector System Bruker Analytical X-Ray Systems*, Bruker axs: Madison, WI, 2013.
4. Sheldrick, G. M. SHELXT-Integrated space-group and crystal-structure determination. *Acta Crystallogr., Sect. A* **2015**, *71*, 3-8.
5. Sheldrick, G. M. Crystal structure refinement with SHELXL. *Acta Crystallogr., Sect. C* **2015**, *71*, 3-8.
6. Kleemiss, F.; Dolomanov, O. V.; Bodensteiner, M.; Peyerimhoff, N.; Midgley, L.; Bourhis, L. J.; Genoni, A.; Malaspina, L. A.; Jaytilaka, D.; Spencer, J. L. Accurate crystal structures and chemical properties from NoSpherA2. *Chem. Sci.* **2021**, *12*, 1675-1692.
7. *CrysAlisPro*, V1.171.41.98a; Rigaku Oxford Diffraction: Yarnton, England, 2021.
8. Bourhis, L. J.; Dolomanov, O. V.; Gildea, R. J.; Howard, J. A. K.; Puschmann, H. The anatomy of a comprehensive constrained, restrained, refinement program for the modern computing environment - Olex2 Dissected. *Acta Crystallogr., Sect. A* **2015**, *A71*, 59-71.
9. Krause, L.; Herbst-Irmer, R.; Sheldrick, G. M.; Stalke, D. Comparison of silver and molybdenum microfocus X-ray sources for single-crystal structure determination. *J. Appl. Cryst.* **2015**, *48*, 3-10.
10. Dolomanov, O. V.; Bourhis, L. J.; Gildea, R. J.; Howard, J. A. K.; Puschmann, H. OLEX2: a complete structure solution, refinement, and analysis program. *J. Appl. Cryst.* **2009**, *42*, 339-341.
11. Groom, C. R.; Bruno, I. J.; Lightfoot, M. P.; Ward, S. C. The Cambridge Structural Database. *Acta Crystallogr., Sect. B* **2016**, *72*, 171-179.
12. Kratzert, D. *FinalCif*.
